# Mechanistic Characterisation of a Diterpene Synthase for Chryseojoostenes A–E from *Chryseobacterium Joostei*

**DOI:** 10.1002/anie.202513149

**Published:** 2025-09-08

**Authors:** Georges B. Tabekoueng, Heng Li, Kexin Yang, Lukas Lauterbach, Bernd Goldfuss, Jeroen S. Dickschat

**Affiliations:** ^1^ Kekulé Institute of Organic Chemistry and Biochemistry University of Bonn Gerhard‐Domagk‐Straße 1 53121 Bonn Germany; ^2^ Department of Chemistry and Biochemistry University of Cologne Greinstraße 4 50939 Cologne Germany

**Keywords:** Biosynthesis, Enzymes, Hydrocarbons, Substrate analogs, Terpenoids

## Abstract

A diterpene synthase from *Chryseobacterium joostei* was characterised and produces the five unique compounds chryseojoostenes A–E. Chryseojoostenes D and E were produced in too low amounts for isolation from the wildtype enzyme, but extensive site‐directed mutagenesis resulted in an enzyme variant in which the production of these compounds was enhanced. The biosynthesis of the enzyme products was investigated in detail through a combined experimental and computational approach, indicating a complex hydrogen scrambling during terpene cyclisation and a long‐range proton shift towards chryseojoostene E. Density functional theory (DFT) calculations revealed that a similar long range hydrogen shift is involved in the formation of an even fragment ion (*m*/*z* 216), characterising the unique chemistry of the chryseojoostene skeleton. Further insights into the cyclisation mechanism were obtained by enzymatic conversion of two substrate analogs with reduced reactivity.

## Graphical abstract

The bacterial diterpene synthase CjCS for chryseojoostenes was mechanistically characterised through labelling experiments, density functional theory (DFT) calculations, and conversion of substrate analogs with blocked reactivity. Site‐directed mutagenesis gave access to minor enzyme products, including chryseojoostene E, whose biosynthesis involves a long‐range hydrogen shift that is also relevant to its EIMS fragmentation.

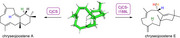

## Introduction

Terpenes and consequently also terpene synthases (TSs) occur in all kingdoms of life. The first enzymes have been reported from land plants and fungi, including the famous examples of 5‐*epi*‐aristolochene synthase from *Nicotiana tabacum*,^[^ ^1^ ^]^ the taxa‐4,11‐diene synthase from *Taxus brevifolia*,^[^ ^2^ ^]^ trichodiene synthase from *Fusarium sporotrichioides*,^[^ ^3^ ^]^ and aristolochene synthase from *Penicillium roqueforti*.^[^ ^4^ ^]^ Also, bacteria are rich in TSs as exemplified by pentalenene synthase from *Streptomyces exfoliatus* and *epi*‐isozizaene synthase from *Streptomyces coelicolor*.^[^ ^5^, ^6^ ^]^ Only recently have animals been identified as carriers of TSs,^[^ ^7^, ^8^ ^]^ while protists,^[^ ^9^ ^]^ marine algae,^[^ ^10^ ^]^ and viruses^[^ ^11^ ^]^ belong to the exotic sources. Despite the fact that today hundreds of TSs have been functionally characterised, these fascinating enzymes continue to be of high interest, especially because of the intriguing structures of their products constructed from comparably simple acyclic precursors and the unusually complex mechanisms leading to these compounds. Each newly discovered enzyme provides a mechanistic riddle and allows for access to a structurally complex molecule in a single enzymatic step that would be very difficult to make by any other means such as total synthesis. Recent research has demonstrated that TSs are also very useful catalysts in the conversion of substrate analogs,^[^ ^12^ ^]^ allowing for the rapid access to derivatives of their natural product. Moreover, the structural changes in the substrate can sometimes be associated with a change in reactivity that leads to surprising products with novel skeletons.^[^ ^13^ ^]^ Another possibility to diversify the products of terpene synthases is targeting of active site residues through site‐directed mutagenesis (SDM).^[^ ^14^, ^15^ ^]^ Here, we present the functional and mechanistic characterisation of a diterpene synthases (DTS) from *C. joostei* DSM 16927, a representative bacterium from a biologically well described genus with largely unexplored secondary metabolism,^[^ ^16^, ^17^, ^18^, ^19^, ^20^, ^21^, ^22^ ^]^ application of the DTS in the conversion of substrate analogs and broadening of its product profile using SDM.

## Results and Discussion

**Scheme 1:**
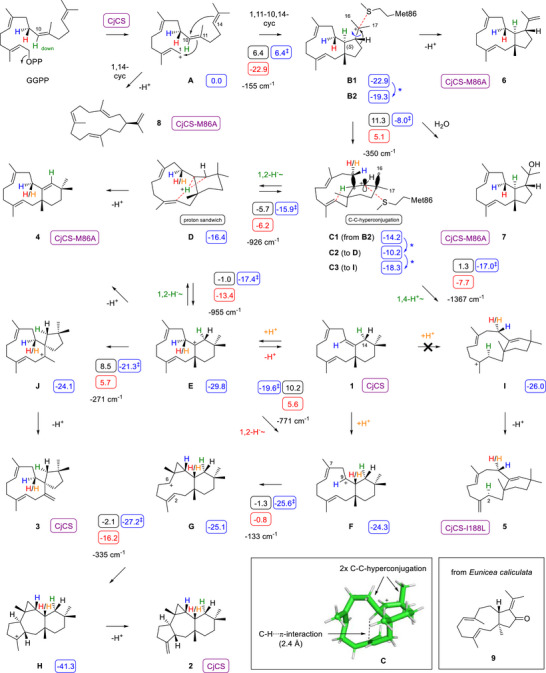
Cyclisation mechanism from GGPP to **1**–**8** by CjCS and its enzyme variants. Blue boxes: free (Gibbs) energies relative to **A** (set to 0.0 kcal mol^−1^; ‡ denotes free energies of transition states), black boxes: free energies of activation (Gibbs reaction barriers), red boxes: free reaction energies (kcal mol^−1^, mPW1PW91/6–311 + G(d,p)//B97D3/6–31G(d,p), 298.15 K, 1 bar). Blue asterisks and arrows indicate required minor conformational changes between computed structures. Imaginary frequencies of transition states are given in cm^−1^. Purple boxes next to isolated compounds indicate the source enzyme. Large boxes: computed structure of intermediate **C** and structure of compound **9** known from *E. caliculata*.

The gene for a TS homolog from *C. joostei* DSM 16927 (accession number WP_076356785) was cloned into the expression vector pYE‐Express through homologous recombination in yeast and expressed in *E. coli* BL21 (DE3). The encoded enzyme represents a larger branch in a phylogenetic tree constructed from 5000 bacterial type I TSs, but no closely related enzymes have been characterised to date (Figure S1). The main aim of the approach to select candidate terpene synthases that are phylogenetically distant to all characterised enzymes is to enable the discovery of new enzyme functions, which turned out to be a successful strategy in several of our previous studies. The nearest relatives of the TS homolog from *C. joostei* are the wanjudiene synthase from *Chryseobacterium wanjuense* ^[^ ^19^ ^]^ and the polytrichastrene synthase from *Chryseobacterium polytrichastri*,^[^ ^21^ ^]^ with amino acid sequence identities of only 27% and 26%, respectively. The predicted amino acid sequence shows the presence of all highly conserved motifs of bacterial type I TSs (Figure S2), suggesting functionality of the enzyme. The purified protein (Figure S3) was incubated with different oligoprenyl diphosphates, showing efficient conversion of geranylgeranyl diphosphate (GGPP) into a mixture of diterpenes, while geranylfarnesyl diphosphate (GFPP) was not accepted, and geranyl diphosphate (GPP) and farnesyl diphosphate (FPP) were mostly converted into simple acyclic lysis and hydrolysis products (Figures S4–S6, Table S1). The three main compounds from GGPP were isolated and their structures elucidated as the new diterpenes chryseojoostene A (**1**), B (**2**) and C (**3**) (Scheme 1, Figures S7–S38, Tables S2–S5), while three side products (**4**–**6**) were produced in too low amounts for compound isolation, especially considering the challenges of chromatographic separation of several hydrocarbon products. Significant line broadening was observed in the NMR spectra of **3** recorded at 298 K, a well‐known phenomenon in the NMR spectroscopy of terpenes,^[^ ^23^, ^24^ ^]^ but structure elucidation could be completed with spectra recorded at 343 K showing sharper signals. Thus, the enzyme was identified as *C. joostei* Chryseojoostene Synthase (CjCS).

The absolute configurations of compounds **1**–**3** were determined through stereoselective labelling experiments (all labelling experiments are summarised in Table S6). Our chemical correlation strategy makes use of four stereoselectively labelled isotopomers of isopentenyl diphosphate (IPP), including (*R*)‐ and (*S*)‐(1–^13^C,1–^2^H)IPP,^[^ ^25^ ^]^ and (*E*)‐ and (*Z*)‐(4–^13^C,4–^2^H)IPP.^[^ ^26^ ^]^ Their conversion with oligoprenyl diphosphate synthases, in the present case with GGPP synthase from *Streptomyces cyaneofuscatus*,^[^ ^27^ ^]^ and eventually isopentenyl diphosphate isomerase (IDI) from *E. coli*,^[^ ^28^ ^]^ proceeds with a known stereochemical course^[^ ^29^ ^]^ and installs stereogenic centres of known configuration in all methylene groups of GGPP. Conversion with CjCS introduces these stereogenic centres into **1**–**3**, turning the enantiotopic hydrogens in GGPP into diastereotopic hydrogens that can be discriminated by HSQC spectroscopy (additional ^13^C‐labels at the deuterated carbons enhance the relevant signals and omit the need of compound purification). The NOESY based assignments of the diastereotopic hydrogens then allows to conclude on the absolute configurations of the CjCS products (Figures S39–S43).

The cyclisation mechanism from GGPP to **1**–**3** starts with the substrate ionisation to **A**, a 1,11‐cyclisation and 10,14‐cyclisation to **B** (Scheme 1; intermediates **B1** and **B2**, and **C1**–**C3** represent slightly different conformers identified in the DFT calculations discussed below). A ring expansion can lead to the secondary cation **C** that undergoes a 1,2‐hydride migration to **E** (the relevance of **D** will be explained later in this article), whose deprotonation results in the main product **1**. Its reprotonation generates the homoallyl cation **F** that can alternatively be formed from **E** by 1,2‐hydride migration, bypassing main product **1**. Subsequently, **F** can react in a 7,9‐cyclisation to the cyclopropylcarbinyl cation **G**, followed by a 2,6‐cyclisation to **H**, the precursor of **2** by deprotonation. Compound **3** is explainable assuming a side branch starting from **E** through a ring contraction to spirocyclic **J** and deprotonation.

This mechanistic proposal was investigated in depth through a series of isotopic labelling experiments. The introduction of single ^13^C‐labels into the individual carbon positions from the 20 isotopomers of (^13^C)GGPP (these compounds were synthesised or enzymatically prepared with GGPPS from synthetic precursors)^[^ ^27^, ^30^, ^31^ ^]^ confirmed the biosynthetic origin of the carbon frameworks of **1**–**3** and in particular supported the skeletal rearrangements from **B** to **C** and from **E** to **J** (Figures S44–S49). For the main compound **1** these experiments revealed a minor distribution of the labelling from C16 and C17 over the geminal Me groups, explainable by a rotation of the *i*Pr group in **B**. This phenomenon has previously been observed with other TSs,^[^ ^32^, ^33^ ^]^ but often a tight control for the stereoselectivity at the carbon carrying the geminal Me groups is observed.^[^ ^34^, ^35^ ^]^ The 1,2‐hydride shift from **C** to **E** was investigated using (2–^2^H)GPP,^[^ ^36^ ^]^ a compound that was synthesised through a known method.^[^ ^37^, ^38^ ^]^ Its GGPPS catalysed elongation with IPP and cyclisation with CjCS was followed by product isolation and NMR spectroscopy, revealing substitution of H14α by deuterium in **1** (Figure S50). Because 1,2‐hydride shifts are necessarily suprafacial processes, this finding allowed to conclude on a 10*Re*,14*Re*‐cyclisation in **A** leading to a 10*S* configuration in **B**. The labelling experiments with (*R*)‐(1–^13^C,1–^2^H)IPP (blue hydrogen in Scheme 1 = ^2^H) and product analysis by GC/MS indicated that the terminal deprotonation from **E** to **1** proceeds mainly with retainment of the 9‐*pro*‐*R*, but also a minor loss of this proton was observed (ca. 12%; Figure S51B). Accordingly, with (*S*)‐(1–^13^C,1–^2^H)IPP (red H = ^2^H) a major loss of the 9‐*pro*‐*S* hydrogen, but also its minor retainment was detected (34%, the differences in the percentage point to a kinetic isotope effect that favours retainment of deuterium). The incubation of GGPP with CjCS in a D_2_O buffer resulted in the minor uptake of deuterium into **1** that is explainable by a reprotonation of **1** at C9 to **E** and subsequent deprotonation with loss of the other hydrogen from C9 (Figure S51C). The following series of experiments indicated that cation **F** is partially formed through a 1,2‐hydride shift from **E**, but also partially through deprotonation to **1** and reprotonation to **F**. GC/MS analysis of the product **2** from (*R*)‐(1–^13^C,1–^2^H)IPP showed an almost full retainment of all four deuterium atoms, but also a minor loss of deuterium is observed for the material formed through **1** (5%, Figure S52B). The experiment using (*S*)‐(1–^13^C,1–^2^H)IPP revealed a major loss of one deuterium for the product **2**, but also partial retainment (37%). The formation of **F** by protonation of **1** was confirmed by an incubation of GGPP in D_2_O, resulting in partially deuterated **2** (Figure S52C). To locate the incorporated deuterium atom in **2** expected to reside at C10, (2–^13^C)GPP,^[^ ^39^ ^]^ and IPP were incubated with GGPPS and CjCS, and the product was analysed by ^13^C‐NMR spectroscopy. The idea of this approach is that the direct ^13^C─^2^H bond will result in a triplet signal due to ^13^C─^2^H spin coupling (with a coupling constant of ^1^ *J*_C,H_ ≈ 20 Hz) that is moderately deshielded because of an isotope effect of deuterium (Δ*δ* ≈ −0.5 ppm). However, this experiment failed, because **2** is a minor product and the signal in the ^13^C‐NMR was too weak. The problem is that the nuclear quadrupole moment of deuterium causes a slow spin–spin relaxation and leads to weak signals. Therefore, an alternative approach was used, placing a ^13^C‐label at the neighbouring carbon C9 whose chemical shift will likewise be influenced by deuterium (expected chemical shift difference: Δ*δ* ≈ −0.1 ppm). The incubation of (1–^13^C)GPP and IPP with GGPPS and CjCS revealed two signals, one at the original chemical shift for C9 (no deuterium incorporation), and one slightly upfield shifted (Δ*δ* = −0.12 ppm, deuterium incorporation at C10; Figure S53).

The cyclisation mechanism of CjCS was further investigated through DFT calculations (Table S7, Figure S54). According to the results, the 1,11–10,14‐cyclisation from **A** to **B** proceeds in a single highly exergonic step through a low barrier. The ring expansion to the secondary cation **C** has a moderate activation barrier (11.3 kcal mol^−1^) and is only slightly endergonic, because **C** is well stabilised by hyperconjugation with two adjacent C─C bonds (relevant orbitals are shown at the structure of **C** and the computed structure of **C1** is shown in the box of Scheme 1). Notably, H10 shows a C─H∙∙∙π interaction with the C2═C3 double bond, leading in the next barrierless and exergonic step to the proton sandwich **D**.^[^ ^40^ ^]^ Breaking up the proton sandwich leads to **E** that is again a barrierless step. At this stage, the deprotonation to the main product **1** is the preferred reaction, while two branches to the minor products **2** and **3** can be entered through passing of moderate reaction barriers. The first option is a 1,2‐hydride shift to **F** (10.2 kcal mol^−1^, Δ*G* = 5.6 kcal mol^−1^), with the two subsequent cyclisations to **G** and **H** proceeding without activation barrier. The second possibility is the skeletal rearrangement from **E** to **J** that is associated with a reaction barrier of 8.5 kcal mol^−1^.

**Figure 1:**
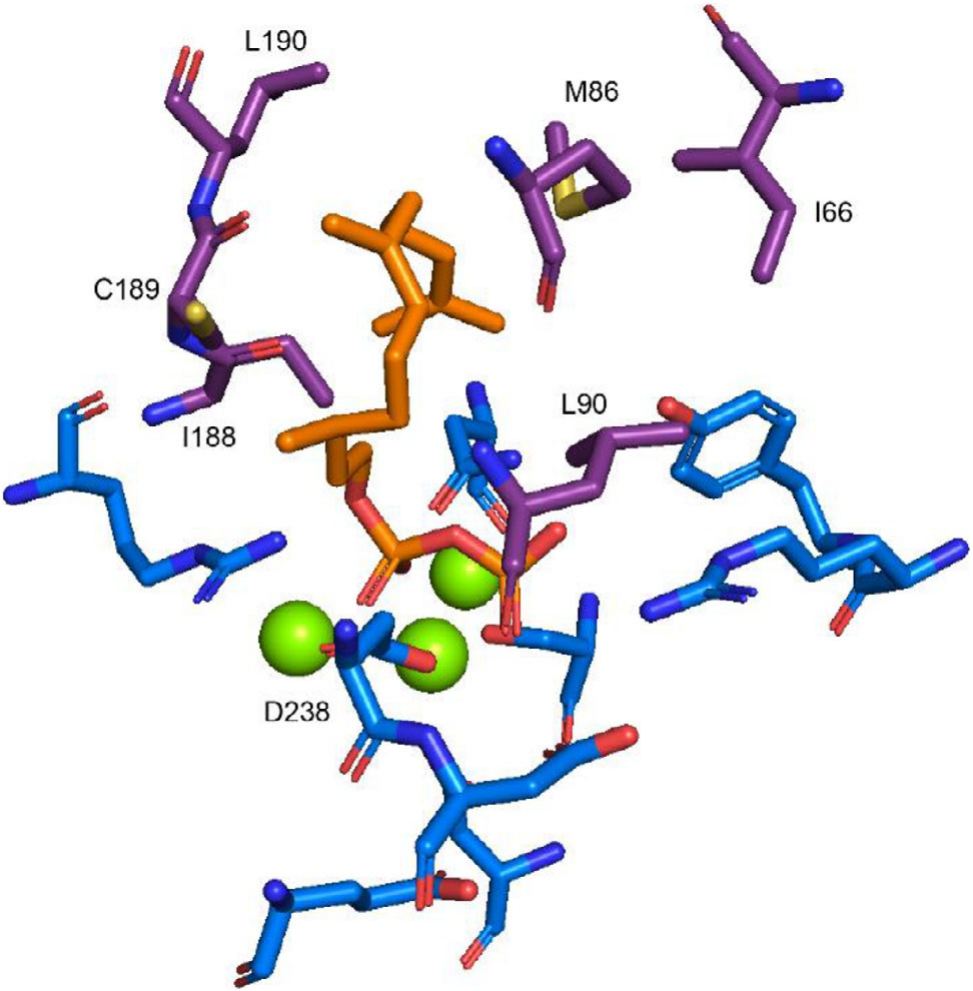
AlphaFold2 model showing the active site residues of CjCS. Residues involved in binding of the substrate and the Mg^2+^ cofactor are shown in blue, residues contouring the hydrophobic cavity that were targeted by site‐directed mutagenesis are shown in purple. The trinuclear Mg^2+^ cluster (green spheres) and the substrate analog 2,3‐dihydro‐FPP (orange) were taken from the crystal structure of selina‐4(15),7(11)‐diene synthase (PDB: 4OKZ).

**Figure 2:**
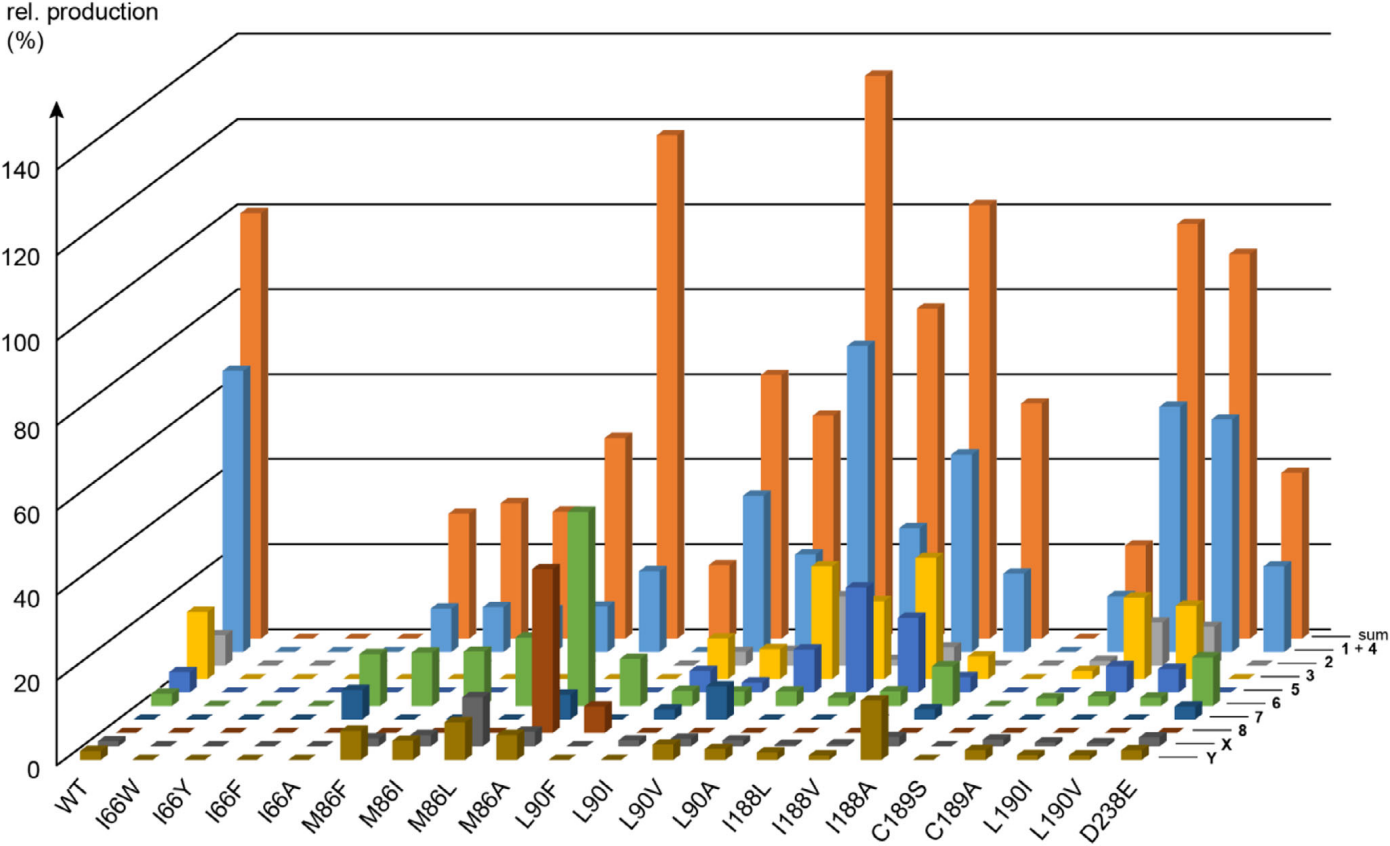
Relative compound production by CjCS wildtype and its enzyme variants constructed by site‐directed mutagenesis. The bars represent means from triplicates, standard deviations are given in Table S9. The sum of **1**–**8** and the unknown compounds **X** and **Y** produced by the wildtype was set to 100%.

Site‐directed mutagenesis is an effective approach to alter the product profiles of terpene synthases and to potentially enrich side products that are observed in small amounts in the wildtype in an enzyme variant.^[^ ^15^ ^]^ In the present case, the aim was to get hands on the minor products **4**–**6** of CjCS and eventually newly formed compounds. For this purpose, an AlphaFold2 model of CjCS was generated to identify key residues of the hydrophobic cavity that determine the substrate fold in CjCS (Figure 1). The model showed the same α‐helical fold as observed in avian FPP synthase^[^ ^41^ ^]^ and aligned well with the crystal structure of selina‐4(15),7(11)‐diene synthase in complex with three Mg^2+^ and the substrate surrogate 2,3‐dihydro‐FPP^[^ ^42^ ^]^ (with a root mean square deviation of 1.086 Å; the predicted local distance difference test plot is shown in Figure S55). The overlayed structures of the CjCS model and of SdS showed a reasonable positioning of all highly conserved residues involved in substrate and Mg^2+^ cofactor binding. While such enzyme models should be taken with care, these facts gave us some confidence that the model can be used qualitatively for the design of enzyme variants. From the residues lining the hydrophobic cavity of CjCS six residues were selected for mutation, in addition to D238 from the NSE triad, because this position is usually occupied by Glu. In total, 21 enzyme variants with altered active site residues have been generated (Figures 2, S2 and S56–S59, Tables S8–S10). The position I66 attracted our attention, because this position is often filled with an aromatic residue, and previous engineering work on polytrichastrene synthase proved to be very successful for the corresponding I66F variant that resulted in the formation of several new diterpenes.^[^ ^21^ ^]^ However, for CjCS all three enzyme variants I66W, I66Y, and I66F were inactive, while the I66A variant showed a reduced productivity (29 ± 3% of wildtype level) and lost the ability to generate **2** and **3**. Similarly, the enzyme variants M86F (32 ± 1%), M86I (30 ± 6%) and M86L (37 ± 6%) gave a reduced compound production, but for the M86A variant a slight increase was observed (118 ± 9%), along with a dramatic shift towards compounds **6**–**8**. For all four M86 enzyme variants the production of **1** was strongly reduced, showing the critical function of this residue for the formation of the CjCS main product. From the M86A variant the four compounds chryseojoostene D (**4**), 12‐*epi*‐dolabella‐3,7,18‐triene (**6**), 12‐*epi*‐18‐hydroxy‐dolabella‐3,7‐diene (**7**) and (+)‐cembrene A (**8**) were isolated (Scheme 1, Figures S60–S83, Tables S11 – S14), demonstrating the success of the mutational approach to isolate trace compounds detected in the wildtype and newly observed compounds from enzyme variants. Met86 may be involved in cation stabilisation^[^ ^43^ ^]^ of **B** and eventually **C** (Scheme 1), and lack of this stabilisation may result in the derailment products **6**–**8**. However, the effect of the M86A exchange could also be of steric nature, as the smaller Ala residue may lead to less strict substrate/intermediate control and widen the cavity for the entry of water explaining the formation of **7**. This view is also supported by the fact that other residues in this position incapable of cation stabilisation do not show a similarly pronounced production of **6**–**8**. The M86L variant was also the best producer of the two compounds **X** and **Y**, but their isolation failed, because they notoriously coeluted in case of all chromatographic separation methods tried (column chromatography on normal and AgNO_3_ activated silica gel, HPLC using various column types) (Figure 2).

Also the L90F (17 ± 1%), L90I (62 ± 5%), and L90V (53 ± 1%) variants exhibited a reduced productivity, with no formation of chryseojoostenes, but only **6** and **8** by the L90F variant. Compound production was increased for the L90A variant (132 ± 1%), but no interesting new compound was observed. In contrast, the I188L variant (78 ± 10%) revealed the highest production of **5** among all tested enzyme variants, which is–considering the small structural change introduced here–a remarkable result. This compound was isolated and identified as chryseojoostene E (Scheme 1, Figures S84–S91, Table S15). Also, the I188V exchange (102 ± 6%) gave a good production of **5**, but not I188A (55 ± 2%). The C189S variant was inactive and also the C189A variant showed a poor productivity (22 ± 2%), demonstrating the critical role of this residue for CjCS catalysis. Both exchanges of L190I (98 ± 3%) and L190V (90 ± 6%) showed a similar product pattern and yield as observed for the wildtype. Finally, the D238E variant exhibited a reduced productivity, with no formation of **2**, **3,** and **5**, but instead increased amounts of **6** were observed.

The absolute configurations of the newly identified compounds **4** and **5** were assigned through stereoselective deuteration experiments using the same stereoselectively deuterated substrates as described above with the CjCS‐M188L variant (Figures S92–S95). Analogous experiments were performed with the CjCS‐M86A variant to assign the absolute configuration of **6** (Figures S96 and S97). Unfortunately, the production of **7** was too low in these experiments and its absolute configuration was tentatively assigned assuming a common biosynthesis of **6** and **7** from the same intermediate **B** (Scheme 1). The hydrocarbon **6** represents a stereoisomer of known natural products from diverse organisms^[^ ^44^, ^45^, ^46^ ^]^ or obtained with other characterised TSs and engineered TS variants.^[^ ^47^, ^48^, ^49^ ^]^ The enantiomer of **7** has been reported from the brown seaweed *Dictyota dichotoma* for which the absolute configuration has been assigned^[^ ^50^ ^]^ using Horeau's method.^[^ ^51^ ^]^ This assignment is now challenged by the same sign of optical rotation for **7** from the CjCS‐M86A variant ([*α*]_D_^25^ = +22.2, *c* 0.09, CH_2_Cl_2_) and the marine natural product ([*α*]_D_ = +27.5, *c* 1, CHCl_3_),^[^ ^50^ ^]^ showing that both materials should be represented by the structure of **7** (Scheme 1). Corey's first enantioselective synthesis of the dolabellane diterpene **9** ^[^ ^52^ ^]^ from the octocoral *Eunicea calyculata* ^[^ ^53^ ^]^ resulted in a revision of its absolute configuration that was previously assigned using Mosher's method.^[^ ^54^ ^]^ Consequently, **7** from CjCS‐M86A and the brown alga, and **9** from the octocoral represent different enantiomeric series. The enantiomeric nature of diterpenes from bacteria and octocorals is now also established for **8** through a comparison of the optical rotations for the CjCS‐M86A product ([*α*]_D_^25^ = +5.5, *c* 0.04, CH_2_Cl_2_) and the ErTC2 product from *Eleutherobia rubra* ([*α*]_D_^25^ = −28.0, *c* 0.02, CHCl_3_).^[^ ^8^ ^]^ Notably, **8** is the only compound that requires a switch in the cyclisation mode from a 1,11–10,14‐cyclisation to a 1,14‐cyclisation in the CjCS‐M86A variant.

In contrast, the formation of **4** and **5** is well understandable from intermediates of the CjCS cyclisation cascade: **4** is an alternative deprotonation product of **E**, and **5** showing an unusual double bond position (C3═C15) is explainable from **C** through a 1,4‐proton shift to **I** and deprotonation. The above described C─H∙∙∙π contact in the computed structure of **C** supports this hypothesis, and further DFT calculations revealed a low reaction barrier of only 1.3 kcal mol^−1^ for the proton transfer. The conversion of (2–^2^H)GPP and (2–^13^C)IPP with GGPPS and CjCS‐I188L confirmed the proton shift experimentally and revealed the stereoselective deuterium incorporation into the 2‐*pro*‐*R* position of **5**, as predicted computationally (Figure S98). Significant deuterium incorporation was observed into **3**–**5** after incubation of GGPP in D_2_O buffer, suggesting similar deprotonation/reprotonation sequences as discussed above for **1** and **2** (Figures S99–S101). Specifically, **1** may be reprotonated at C9 to yield **E**, at C10 to form **F**, or at C2 towards **I**, to explain the uptake of deuterium from D_2_O buffer for all enzyme products. However, the incubation of (2–^13^C)GGPP with CjCS‐I188L in D_2_O buffer and ^13^C‐NMR analysis of the products showed an unchanged singlet for C2 of **5**, but no triplet, excluding reprotonation at C2 for a direct formation of **I** (Figure S102). Instead, deuterated **5** must also be formed from **1** by reprotonation to **E**, followed by back reactions to **D** and **C** from which the branch to **I** can be entered.

**Scheme 2:**
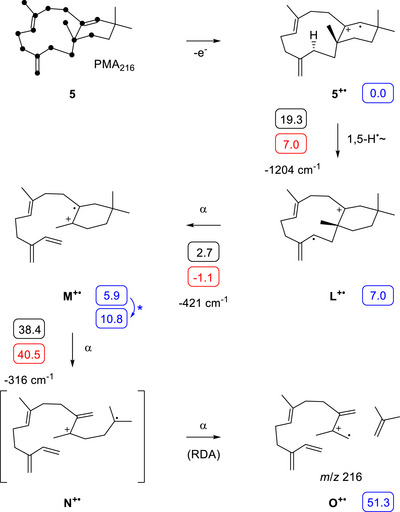
The EI‐MS fragmentation of **5** involving a long‐range hydrogen migration in the formation of the fragment ion *m*/*z* 216. The position‐specific mass shift analysis for this fragment ion, based on individual ^13^C‐labellings of the 20 carbons of **5**, is indicated by the black dots at the structure of **5**. For explanations regarding computational data (small boxes) cf. legend of Scheme 1.

The long range hydrogen migration in the biosynthesis of **5** may also be relevant for its EIMS fragmentation, similar to our recently described findings for the EIMS fragmentation of taxa‐4,11‐diene that proceeds with a long range hydrogen shift^[^ ^55^ ^]^ that also takes place in its biosynthesis.^[^ ^56^, ^57^ ^]^ The individual labelling of all 20 carbons of a diterpene as performed in this study allows for the identification of the molecular portion from which its fragment ions are formed (position‐specific mass shift analysis = PMA). The findings are summarised for the fragment ion *m*/*z* 216 of **5** in Scheme 2 (PMA_216_), which are based on a comparison of the mass spectra of unlabelled **5** and its 20 ^13^C‐labelled isotopomers (Figures S6B, S103, and S104). The carbons contributing to the formation of the fragment ion at *m*/*z* 216 identified through this analysis are marked with black dots at the structure of **5**. After the ionisation of **5** to **5^+•^**, a long range 1,5‐hydrogen migration may lead to **L^+•^** and may be followed by a series of three α‐cleavages through **M^+•^** and **N^+•^** to **O^+•^**, explaining the neutral loss of isobutene. DFT calculations (Table S16, Figure S105) revealed a reaction barrier of 19.3 kcal mol^−1^ for the initial 1,5‐hydrogen migration, which can easily be overcome, because after the ionisation to **5^+•^** with an electron energy of 70 eV the molecule is in a highly excited state. A subsequent α‐fragmentation with a surprisingly low activation barrier (2.7 kcal mol^−1^) leads to **M^+•^** that can undergo a retro‐Diels–Alder (RDA) reaction, realised by two concerted but asynchronous α‐cleavages, to **O^+•^**. The RDA reaction has a substantially higher reaction barrier and is strongly endergonic, which is to be expected for a reaction in which C─C single bonds are cleaved in favour of C═C double bond formation.

**Scheme 3:**
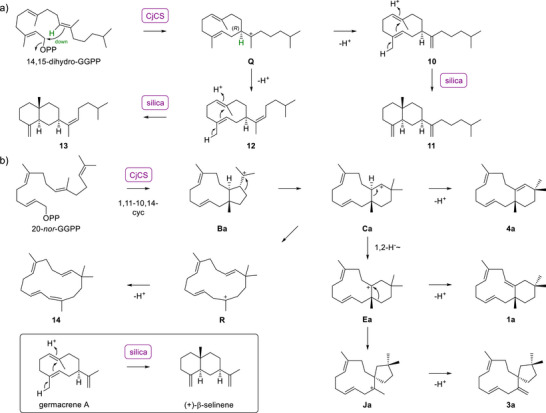
Conversion of substrate analogs with CjCS. a) Conversion of 14,15‐dihydro‐GGPP into the hypothetical products **10** and **12**. These compounds were not isolated, but instead the purification on silica gel resulted in pure **11** and **13** that were not observed in crude enzyme extracts (this is analogous to the behaviour of germacrene A as shown in the box). b) Conversion of 20‐*nor*‐GGPP into the chryseojoostene desmethyl analogs **1a**, **3a** and **4a**, and macrojoostene (**14**). Compounds and intermediates denoted with a small letter “a” (**1a**, **Ba**, etc.) are the desmethyl analogs of those with corresponding name (**1**, **B**, etc.) in Scheme 1.

Many recent examples have shown that terpene synthases can catalyse the conversion of various substrate analogs,^[^ ^12^ ^]^ including inter alia fluorinated,^[^ ^58^ ^]^ hydroxylated,^[^ ^59^ ^]^ or methoxy‐substituted compounds,^[^ ^60^ ^]^ compounds with heteroatoms inserted into the chain,^[^ ^61^ ^]^ with shifted double bonds,^[^ ^13^ ^]^ or modified methylation patterns.^[^ ^62^ ^]^ Such modifications can often lead to a surprising change in reactivity that opens the path towards non‐natural terpenoid skeletons. The saturation of single double bonds of the oligoprenyl diphosphate precursors can give additional insights into the terpene cyclisation cascades that may be interrupted at certain stages, leading to derailment products that represent cationic intermediates.^[^ ^63^, ^64^ ^]^ The incubation of 6,7‐dihydro‐GPP and IPP with GGPPS gave 14,15‐dihydro‐GGPP that was further converted with CjCS into the germacrene A analogs **10** and **12** (Scheme 3a). These compounds were likely detected by GC/MS as their (coeluting) Cope rearrangement products (Figure S106). As described for the acid‐sensitive compound germacrene A,^[^ ^65^ ^]^ after column chromatography on silica gel the bicyclic derivatives 13‐isopentenyl‐β‐selinene (**11**) and (*Z*)‐12‐isopentenyl‐β‐selinene (**13**) were isolated (Figures S107–S122, Tables S17 and S18). Their absolute configurations were assigned based on the following arguments: First, the starting conformation of 14,15‐dihydro‐GGPP in the cyclisation by CjCS should be similar to that of GGPP, with H10 pointing down (compare Schemes 1 and 3a). The changed cyclisation mode of a 1,10‐cyclisation with 14,15‐dihydro‐GGPP will then lead to a 10*R*‐configuration in **10** and **12**. Second, the optical rotation of **11** ([*α*]_D_^25^ = +17.5, *c* 0.063, CH_2_Cl_2_) shows the same sign as that of β‐selinene ([*α*]_D_ = +31.7, CHCl_3_).^[^ ^66^ ^]^ With 20‐*nor*‐GGPP, synthesised as shown in Scheme S1, the three desmethyl analogs 20‐*nor*‐chryseojoostene A (**1a**), 20‐*nor*‐chryseojoostene C (**3a**), and 20‐*nor*‐chryseojoostene D (**4a**) were obtained (Figures S123–S146, Tables S19–S21). Their biosynthesis follows the same logic as for the corresponding natural products from GGPP (Scheme 3b), and it is well understandable that no desmethyl analogs of **2** and **5** were found, because their formation would require secondary cations corresponding to **H** and **I** as intermediates. Instead, a fourth compound was isolated that was structurally characterised as macrojoostene (**14**) (Figures S147–S154, Table S22). Its biosynthesis is explainable by ring opening of **Ca** to **R** and deprotonation.

## Conclusion

In summary, we have identified a diterpene synthase (CjCS) from *C. joostei* that produces cryseojoostenes A–E (**1**–**5**). Two of these compounds were produced in too low amounts by the wildtype enzyme for isolation, but a site‐directed mutagenesis strategy targeting active site residues has made enzyme variants accessible that gave access to sufficient amounts. In this context the M86A variant turned out to be particularly useful, because it gave access to two more compounds, 12‐*epi*‐dolabella‐3,7,18‐triene (**6**) and 12‐*epi*‐18‐hydroxy‐dolabella‐3,7‐diene (**7**), revealing the 10*S* configuration of the bicyclic intermediate **B** that is hidden in the other enzyme products.

The biosynthetic mechanism of CjCS was investigated through isotopic labelling experiments in conjunction with DFT calculations, and is characterised by an unusually complex hydrogen scrambling resulting from a deprotonation–reprotonation sequence, as demonstrated by incubation experiments in deuterium oxide buffer. A similar deuterium uptake has been demonstrated for caryolan‐1‐ol synthase from *Streptomyces griseus*, enabling the incorporation of up to three deuterium atoms.^[^ ^67^ ^]^ The record is held by the non‐canonical C17 sesquiterpene synthase for chlororaphens A and B that can catalyse up to 14 H‐D exchanges.^[^ ^68^ ^]^ Another interesting biosynthetic feature is a long‐range proton shift that is also seen in the biosynthesis of taxa‐4,11‐diene^[^ ^55^, ^56^, ^57^ ^]^ and asperfumene,^[^ ^69^ ^]^ while similar long‐range proton shifts have been proposed based on computational data for trichodiene^[^ ^70^ ^]^ and fusicocca‐2,10(14)‐diene.^[^ ^71^ ^]^ Notably, a hydrogen shift in reverse direction may also be involved in the EIMS fragmentation of **5**.

Capturing the volatiles emitted by *C. joostei* using a closed‐loop stripping apparatus^[^ ^72^ ^]^ and GC/MS analysis revealed no diterpene production, suggesting the gene for CjCS is not expressed under laboratory culture conditions. Another explanation could be rapid conversion by other enzymes, but the coding gene for CjCS is not part of a biosynthetic gene cluster, questioning this hypothesis.

Additional insights into the biosynthesis of **1**–**5** have been obtained with substrates of reduced reactivity. Saturation of the C14═C15 double bond in 14,15‐dihydro‐GGPP blocked the 10,14‐ and shifted the 1,11‐ to a 1,10‐cyclisation to avoid a secondary cation. This demonstrates that the frequently observed 1,11–10,14‐cyclisation in the biosynthesis of di‐ and sesterterpenes^[^ ^73^ ^]^ is only possible as a concerted, albeit likely highly asynchronous process; the initial 1,11‐cyclisation is likely never independent of the second cyclisation event. The substrate analog 20‐*nor*‐GGPP was converted into the corresponding desmethyl analogs of **1**, **3** and **4**, but not of **2** and **5**, which require the presence of the Me group in the substrate to stabilise an intermediate tertiary cation. These examples demonstrate how small structural changes can lead to a dramatically changed reactivity, a strategy by which eventually cyclisation paths to completely new carbon skeletons may be opened.^[^ ^13^ ^]^ We will continue to use these and other strategies to be newly developed to interrogate complex terpene biosynthesis pathways.

## Supporting Information

Additional supporting information can be found online in the Supporting Information section at the end of this article.

## Conflict of Interests

The authors declare no conflict of interest.

## Supporting information



Supporting information

## Data Availability

The data that support the findings of this study are available in the supplementary material of this article.
