# Supplementary material for: Mechanistic Characterisation of a Diterpene Synthase for Chryseojoostenes A–E from Chryseobacterium Joostei
Source: Angew Chem Int Ed Engl. 2025 Sep 8;64(43):e202513149. doi: 10.1002/anie.202513149 (PMC12535395; doi:10.1002/anie.202513149)
Supplement: Supplementary file 1 — Supporting information [file ANIE-64-e202513149-s001.pdf]

Supporting Information for

Mechanistic Characterisation of a Diterpene Synthase for Chryseojoostenes A – E from *Chryseobacterium joostei*

Georges B. Tabekoueng,[a] Heng Li,[a] Kexin Yang,[a] Lukas Lauterbach,[a] Bernd Goldfuss[b] and Jeroen S. Dickschat*[a]

[a] Dr. Georges B. Tabekoueng, Heng Li, Kexin Yang, Dr. Lukas Lauterbach, Prof. Dr. Jeroen S. Dickschat
Kekulé Institute of Organic Chemistry and Biochemistry
University of Bonn
Gerhard-Domagk-Straße 1, 53121 Bonn, Germany
E-mail: dickschat@uni-bonn.de

[b] Prof. Dr. Bernd Goldfuss
Department of Chemistry and Biochemistry
University of Cologne
Greinstraße 4, 50939 Cologne, Germany

Table of Contents

Phylogenetic tree constructed from 5000 bacterial TS homologs	3
Experimental methods	4
GC/MS analysis of CjCS products	8
EI mass spectra of compounds produced from GGPP with CjCS	9
Products obtained from GPP and FPP with CjCS	11
Spectroscopic and physical data of compounds **1**, **2** and **3**	12
Structure elucidation and NMR data of compound **1**	13
Structure elucidation and NMR data of compound **2**	21
Structure elucidation and NMR data of compound **3** at 298 K and 343 K	29
List of incubation experiments with labelled substrates	45
The absolute configuration of **1**, **2** and **3**	46
Results of labelling experiments with CjCS	51
Computational methods and results for the CjCS cyclisation mechanism	61
AlphaFold2 model and site-directed mutagenesis of CjCS	64
SDS-PAGE gels and activity of the purified CjCS variants	70
GC/MS analysis of products from CjCS and its variants	71
Isolation of compounds from CjCS variants	75
Spectroscopic and physical data of compounds **4**, **5**, **6**, **7** and **8**	75
Structure elucidation and NMR data of compound **4**	77
Structure elucidation and NMR data of compound **6**	85
Structure elucidation and NMR data of compound **7**	93
Comparison of measured and previously published ^{13}C-NMR data of **8**	101
Structure elucidation and NMR data of compound **5**	102
The absolute configuration of **4**, **5**, and **6**	110
The long range hydrogen migration in the formation of **5**	116
The deprotonation to **1** and its reprotonation by CjCS, leading to **3**, **4** and **5**	117
The reprotonation of **1** in the biosynthesis of **5**	120
EI mass spectra of the twenty isotopomers of (^{13}C)-**5** obtained with CjCS	121
Computational analysis of the formation of the fragment ion *m*/*z* 216 in **5**	123
Enzymatic conversion of substrate analogs with CjCS and compound isolation	124
GCMS analysis of products obtained from 14,15-dihydro-GGPP with CjCS	126
Structure elucidation and NMR data of compound **11**	127
Structure elucidation and NMR data of compound **13**	135
Synthesis of 20-*nor*-GGPP	143
Structure elucidation and NMR data of compound **1a**	147
Structure elucidation and NMR data of compound **3a**	155
Structure elucidation and NMR data of compound **4a**	163
Structure elucidation and NMR data of compound **14**	171
Cartesian coordinates of computed structures	179
References	217

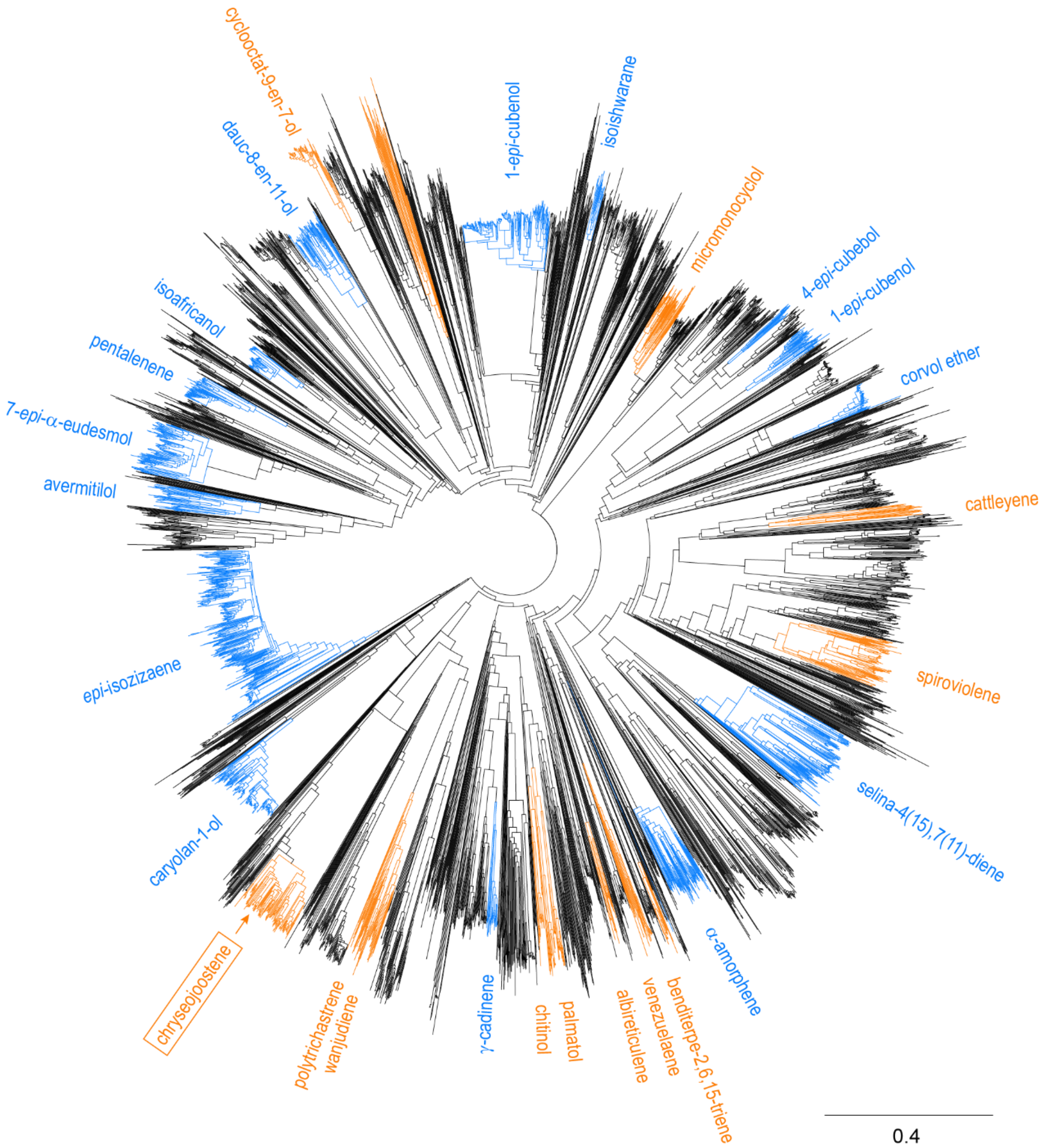

Figure S1. Phylogenetic tree constructed from the amino acid sequences of 5000 bacterial TS homologs. Clades of closely related enzymes with at least one characterised sesquiterpene synthase member are shown in light blue, those with one characterised DTS member are shown in orange (please note that phylogenetic proximity is not necessarily a sign for same enzyme function). The orange box highlights the enzyme newly characterised in this study. The scale bar represents substitutions per site.

Construction of phylogenetic tree

Continuous BLAST searches during the course of our ongoing studies using the amino acid sequences of diverse characterised terpene synthases as probes returned the amino acid sequences of 5000 bacterial terpene synthase homologs. These sequences were all verified by individual inspection for the presence of the highly conserved motifs in type I terpene synthases and used to construct the phylogenetic tree of Figure S1, using the tree builder function of Geneious (alignment type: global alignment with free end gaps, cost matrix: Blosum45, genetic distance model: Jukes-Cantor, tree build method: neighbor-joining, gap open panelty: 8, gap extension penalty: 2).

MNTENFSTLELLYKEFRYPFPTLKNPYAEQLQEITENQWIDGEYLWLYEQNPDLRKKYKKTKTAHIAA
QWFPTATPERFRPICRLMLWTLYN**DDLYE**ESEPEDIGYVHAQSVAVLKGELSGEDSGIPLGKMLATLR
VELLQFIPEESISRFTQMISKYFLGLETELKYKKNKTYPSVSECIAL**R**ENSICLYPFLQLTEVETGVT
LPPEIHAHPVIQRLQALACHLVTFF**NEVQSVIKD**EATGSIYYNIVKVIQNEHQMSLKEACLEDLRLHN
KDLKEFLELQASLPDFGIWQEAVVNWVHYMSMVLSGWKNISTKLD**RY**NMMEFPNVAELKEKLNKI

Figure S2. Amino acid sequence of CjCS from *C. joostei* (accession number WP_076356785, gene locus tag CPIN_RS20360). Highly conserved motifs including the aspartate-rich motif (DDXXD), the pyrophosphate sensor (Arg), the NSE triad (ND(L,I,V)XSXXXE) and the RY pair are highlighted in bold.

Strains and culture conditions
Chryseobacterium joostei DSM 16927 was obtained from the Leibniz Institute DSMZ – German Collection of Microorganisms and Cell Cultures GmbH. The strain was cultivated in medium 1 (5.00 g peptone, 3.00 g meat extract, dissolved in 1 L of distilled water, pH 7.0) at 28 °C. For agar plates, agar (15.0 g L^{-1}) was added to the medium.

Isolation of genomic DNA
C. joostei DSM 16927 cells from a culture in 1. GYM liquid medium (100 mL) were harvested by centrifugation, resuspended in SET buffer (5 mL, 75 mM NaCl, 25 mM EDTA, 20 mM Tris/HCl, pH 8.0) and incubated with lysozyme solution (1 mg/mL) for 30 min at 37 °C. Proteinase K solution (100 µL, 1 mg/mL) was added and the solution was mixed. After addition of 10% SDS (600 µL) and mixing by inversion the mixture was incubated for 1 h at 55 °C. Phenol/chloroform (5 mL) was added and the solution was mixed by inversion before centrifugation for 5 min at 14,000 g. The aqueous layer was transferred to a fresh tube and ice-cold ethanol (70% vol.) was added for precipitation of DNA. After centrifugation and washing with 70% ethanol the DNA was redissolved in TE buffer (100 µL, 10 mM Tris/HCl, 1 mM EDTA, pH 8.0).

Gene cloning
The target gene coding for CjCS (accession number WP_076356785) from *C. joostei* DSM 16927 was amplified from gDNA by PCR using Q5 High-fidelity DNA polymerase (New England Biolabs, Ipswich, MA, USA) and the primer pair WP_076356785-Fw (5’-**GGCAGCCATATGGCTAGCATGACTGGTGGA**<u>ATGAATACAGAAAATTTTAGT</u>-3’) and WP_076356785-Rv (5’-**TCTCAGTGGTGGTGGTGGTGGTGCTCGAGTG**<u>TTATATTTTATT-CAGCTTTTC</u>-3’; end sequences homologous to the linearised pYE-Express vector[1] for homologous recombination are shown in bold, priming sequences are underlined). Yeast homologous recombination of the PCR product with the linearised pYE-Express shuttle vector (digestion with HindIII and EcoRI) was carried out through the standard protocol using LiOAc, polyethylene glycol and salmon sperm DNA.[2] After yeast transformation cultures were grown on SM-URA agar (425 mg yeast nitrogen base, 1.25 g ammonium sulfate, 5 g glucose, 192.5 mg nutritional supplement minus uracil, 5 g agar, 250 mL water) at 28 °C for 3 days. The recombinant plasmid was isolated from grown yeast colonies using the Zymoprep Yeast Plasmid Miniprep II kit (Zymo Research, Irvine, CA, USA) and subsequently used for transformation of *Escherichia coli* BL21 (DE3) electrocompetent cells. Cells were plated on LB agar plates amended with kanamycin sulfate (50 µg mL^{-1}) followed by incubation at 37 °C overnight. Single colonies were selected and used to inoculate LB medium (6 mL) liquid cultures with kanamycin sulfate (6 µL; 50 mg mL^{-1}). After 24 h growth plasmid DNA was isolated and checked for correct insertion of the desired gene by PCR amplification of the inserted DNA sequence using the T7 primer pair and by sequencing. The obtained plasmid was named pYE-CjCS.

Gene expression and protein purification
A starter culture of *E. coli* BL21 (DE3) transformed with pYE-CjCS was grown in LB medium with kanamycin sulfate (50 µg mL^{-1}) overnight with shaking at 37 °C. The gene expression culture was inoculated with the starter culture (2/100) and grown in LB medium containing kanamycin sulfate (50 µg mL^{-1}) with shaking at 37 °C until OD_{600} = 0.4 – 0.6 was reached. After cooling the culture to 18 °C, enzyme expression was induced by the addition of an aqueous IPTG solution (400 mM, 1/1000, v/v). The culture was shaken at 18 °C overnight. The cells were harvested through centrifugation (10,000 g, 5 min, 4 °C), resuspended in cold binding

buffer (10 mL L^{-1} culture; 20 mM Na_2HPO_4, 500 mM NaCl, 20 mM imidazole, 1 mM $MgCl_2$, pH 7.4, 4 °C) and lysed by ultrasonication (8 x 1 min) on ice. The cell debris was removed by centrifugation (14,600 g, 10 min, 4 °C) and the soluble protein fraction was loaded onto a Ni^{2+}-NTA superflow affinity chromatography column (Qiagen, Venlo, Netherlands) equilibrated with binding buffer. The column was washed with washing buffer (2 x 10 mL L^{-1} culture; 20 mM Na_2HPO_4, 500 mM NaCl, 50 mM imidazole, 1 mM $MgCl_2$, pH 7.4, 4 °C) and the desired protein was eluted with elution buffer (2 x 6.25 mL L^{-1} culture; 20 mM Na_2HPO_4, 500 mM NaCl, 500 mM imidazole, 1 mM $MgCl_2$, pH 7.4, 4 °C). The protein concentration of purified CjCS (Figure S3) was determined by Bradford assay (1.5 mg mL^{-1}).[3]

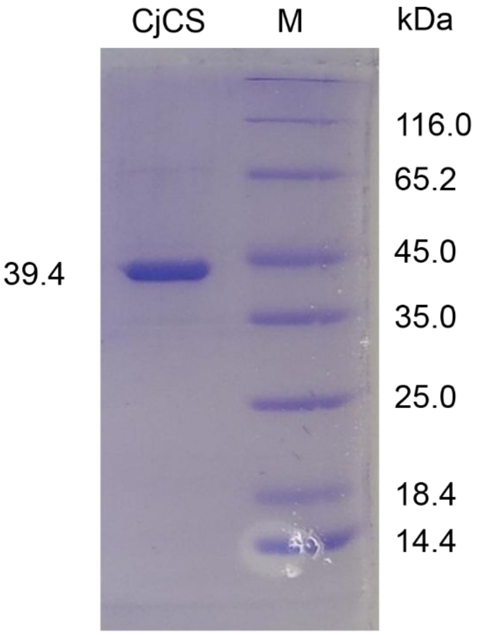

Figure S3. SDS-PAGE analysis of purified recombinant CjCS from *Chryseobacterium joostei* DSM 16927.

Incubation experiments with recombinant CjCS

Test incubations were carried out to identify the substrate scope of recombinant CjCS with the trisammonium salts of GPP, FPP, GGPP or GFPP (1 mg) dissolved in substrate buffer (1.0 mL; 25 mM NH_4HCO_3) and diluted with binding buffer (1.5 mL) and incubation buffer (3.0 mL; 50 mM Tris/HCl, 10 mM $MgCl_2$, 20% glycerol, pH = 8.2). A protein preparation (0.5 mL, from 400 mL expression culture, protein concentration 1.1 mg mL^{-1}) was added to each substrate, followed by incubation with shaking at 28 °C overnight. The products were extracted with hexane (100 μL), the extracts were dried with $MgSO_4$ and analysed by GC/MS.

General methods

Chemicals were purchased from Sigma Aldrich Chemie GmbH (Steinheim, Germany), Carbolution Chemicals GmbH (St. Ingbert, Germany), or Carl Roth (Karlsruhe, Germany) and used without purification. Solvents for column chromatography were purchased in p.a. grade and purified by distillation. Thin-layer chromatography (TLC) was performed with 0.2 mm precoated plastic sheets Polygram Sil G/UV254 purchased from Machery-Nagel (Düren, Germany). Column chromatography was performed using silica gel 60 purchased from Merck (Darmstadt, Germany).

GC/MS

GC/MS analyses were performed on a 5977A GC/MSD system (Agilent, Santa Clara, CA, USA) with a 7890B GC and a 5977A mass selective detector. The GC was equipped with a HP5-MS fused silica capillary column (30 m, 0.25 mm i. d., 0.50 μm film). Specific GC settings were 1) inlet pressure: 77.1 kPa, He at 23.3 mL min^{-1}, 2) injection volume: 1 μL, 3) temperature program: 5 min at 50 °C increasing at 10 °C min^{-1} to 320 °C, 4) 60 s valve time, and 5) carrier gas: He at 1.2 mL min^{-1}. MS settings were 1) source: 230 °C, 2) transfer line: 250 °C, 3) quadrupole: 150 °C and 4) electron energy: 70 eV. Retention indices (*I*) were determined from retention times in comparison to the retention times of *n*-alkanes (C_7-C_{40}).

HRMS

High resolution mass spectra were recorded with LTQ Orbitrap XL (Thermo Fisher Scientific, Waltham, Massachusetts, USA).

HPLC

Analytical scale HPLC seperation was carried out using a PLATINblue series HPLC system (Knauer, Berlin, Germany), equipped with PAD-1 photodiode array detector (190–1000 nm) and a KNAUER Eurospher II 100^{-3} C18 column (3.0 μm; 2.0 mm × 100 mm). The UV-Vis absorption was monitored at 190 – 600 nm. Preparative scale HPLC purification was performed on an Azura series HPLC system (Knauer, Berlin, Germany) with a multi wavelength detector MWL 2.1L (190 – 700 nm) using a Knauer Eurospher II 100^{-5} C18 column (5 μm; 8 x 250 mm).

NMR spectroscopy

NMR spectra were recorded on a Bruker (Billerica, MA, USA) Avance I (300 MHz), Avance I (400 MHz), Avance I (500 MHz), Avance III HD Prodigy (500 MHz) or an Avance III HD Cryo (700 MHz) NMR spectrometer. Spectra were measured in C6D6 and referenced against solvent signals (^{1}H-NMR, residual proton signal: δ = 7.16; ^{13}C-NMR: δ = 128.06).[4]

IR spectroscopy

IR spectra were recorded on a Bruker α infrared spectrometer with a diamond ATR probehead. Peak intensities are given as s (strong), m (medium), w (weak) and br (broad).

Optical rotations

Optical rotations were recorded on a Modular Compact Polarimeter MCP 100 (Anton Paar, Graz, Austria). The temperature setting was 25 °C; the wavelength of the light used was 589 nm (sodium D line); the path-length was 10 cm; the compound concentrations *c* are given in g 100 mL^{-1}.

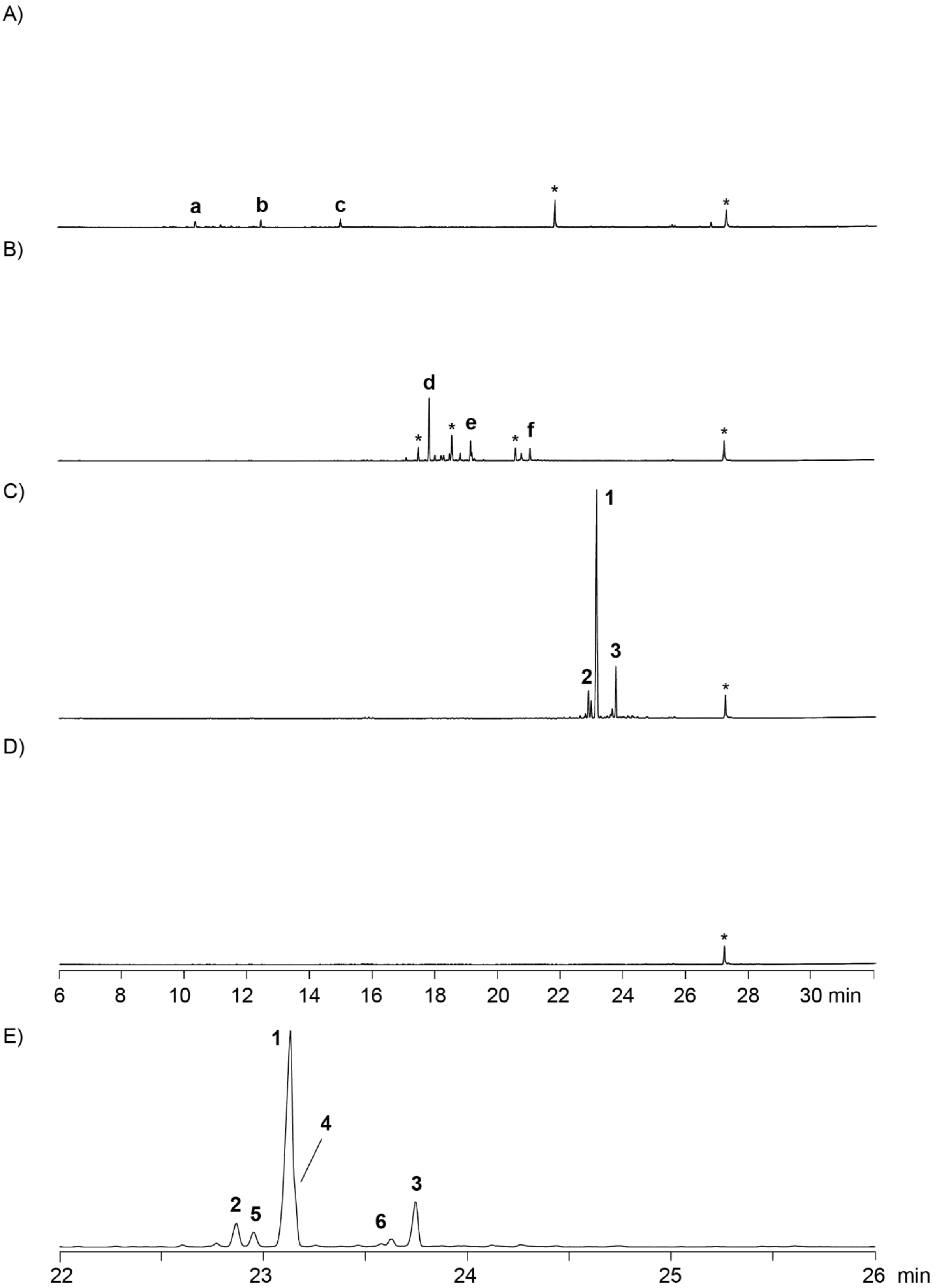

Figure S4. Total ion chromatogram of the products obtained with CjCS from A) GPP, B) FPP, C) GGPP and D) GFPP. The products obtained from GPP and FPP are listed in Table S1. E) Expansion of the chromatogram shown in C) detailing the products obtained from GGPP. Compounds **4** – **6** were isolated from the CjCS-M86A and the CjCS-I188L variant, respectively (vide infra).

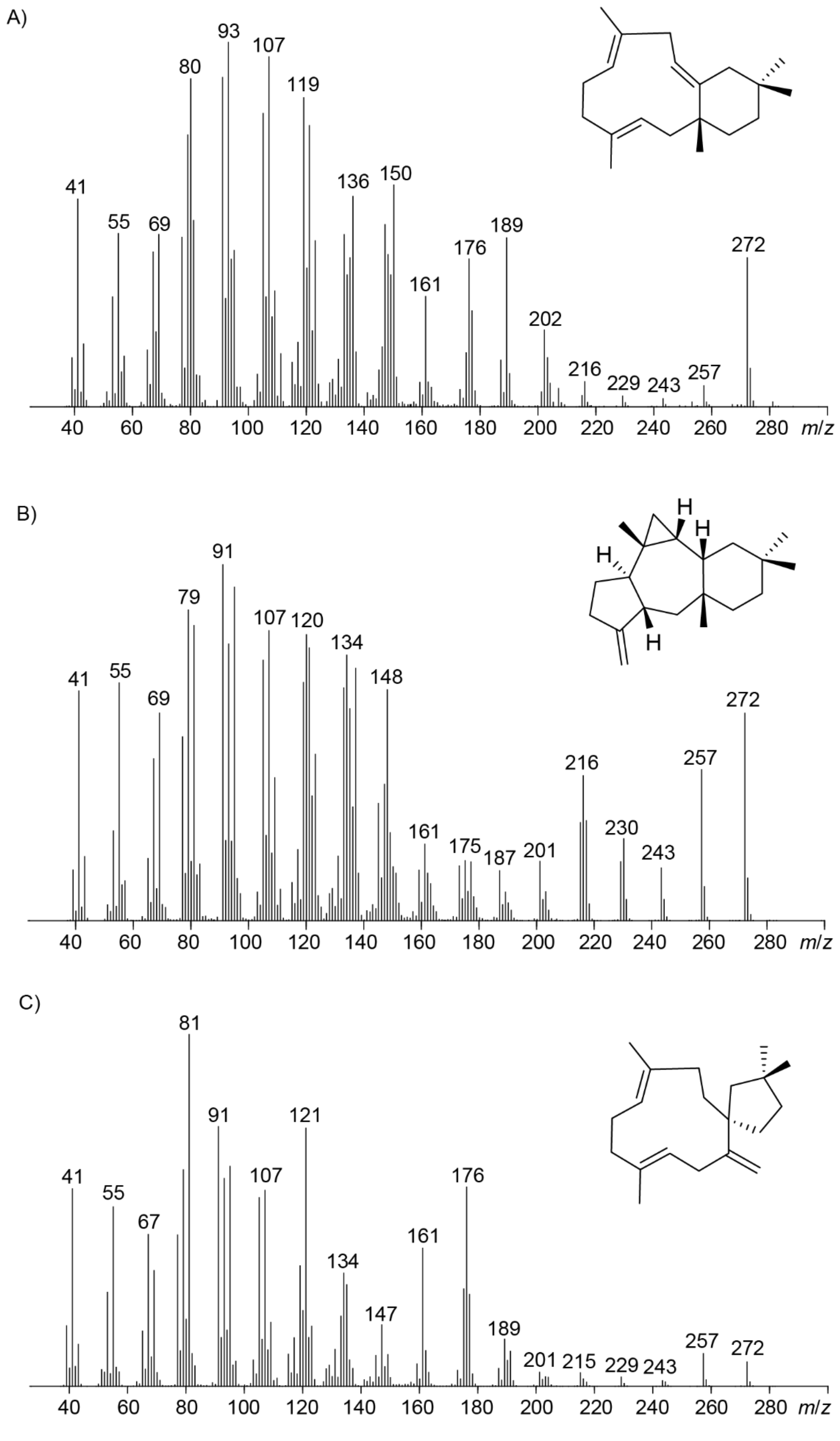

Figure S5. EI mass spectra of A) chryseojoostene A (**1**), B) chryseojoostene B (**2**), and C) chryseojoostene C (**3**).

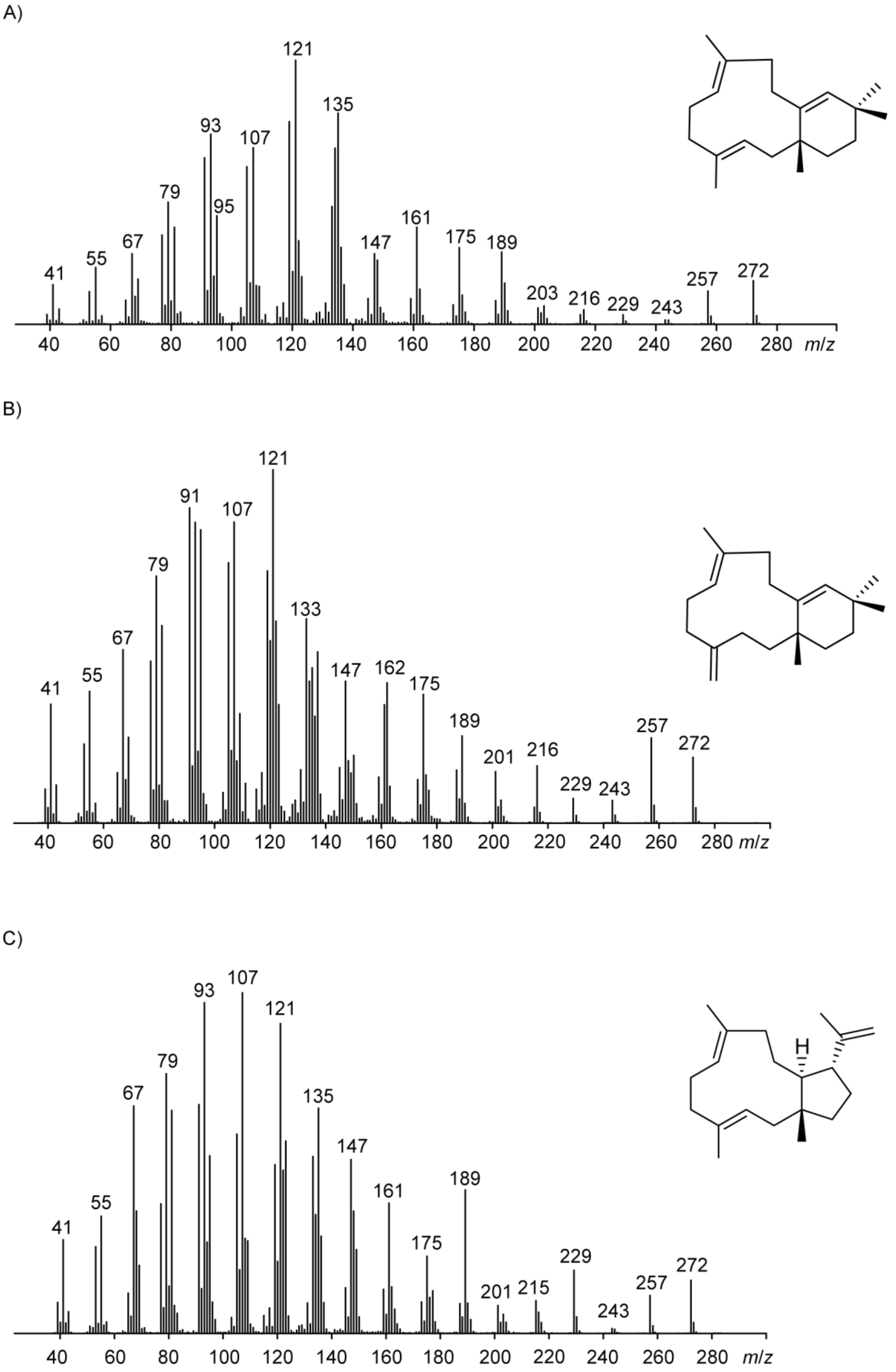

Figure S6. EI mass spectra of A) chryseojoostene D (**4**), B) chryseojoostene E (**5**), and C) 12-*epi*-dolabella-3,7,18-triene (**6**). These three minor compounds were not isolated from wildtype CjCS, but from the CjCS-M86A and CjCS-I188L variant, respectively (vide infra).

Table S1. Products obtained from GPP and FPP with CjCS.

Compound	*I*[a]	*I*(lit.)[b]	MS match[c]
myrcene (**a**)	989	988[5]	919
linalool (**b**)	1099	1095[5]	932
geraniol (**c**)	1255	1249[5]	934
(*E*)-β-farnesene (**d**)	1461	1454[5]	945
elemol (**e**)	1566	1548[5]	960
farnesol (**f**)	1728	1742[5]	940

[a] Retention index on a HP5-MS column. [b] Retention index literature data for the same or a similar GC column. [c] MS match factor (a match factor of 1000 indicates identical EI mass spectra).

Preparative scale incubation of GGPP with recombinant CjCS and compound isolation
For a preparative scale incubation, GGPP (trisammonium salt, 80 mg, 0.16 mmol) in NH_4HCO_3 (25 mM, 20 mL) and an enzyme preparation of CjCS (40 mL; from 8 L expression culture, 2.6 mg mL^{-1}) were added to incubation buffer (100 mL). The reaction mixture was stirred overnight at 28 °C and then extracted with n-hexane (3 x 200 mL). The combined extracts were dried with $MgSO_4$ and concentrated under reduced pressure. For column chromatogray with $AgNO_3$ coated silica gel, a saturated solution of $AgNO_3$ in MeOH was prepared. Silica gel (50 g) was mixed with this solution (150 mL), followed by evaporation of MeOH. The crude product mixture obtained from the enzymatic reaction as described above was purified via silica gel chromatography, followed by further chromatographic steps. The first fraction of the silica gel chromatography with elution of 100% *n*-pentane was further purified via silica gel coated with $AgNO_3$ with elution of *n*-pentane : Et_2O (100 : 1) to afford pure chryseojoostene A (2.7 mg, 9.9 µmol, 6.2%), chryseojoostene B (0.8 mg, 2.9 µmol, 1.9%) and chryseojoostene C (1.3 mg, 4.7 µmol, 2.9%).

Chryseojoostene A (1). TLC (pentane): R_f = 0.60. GC (HP-5MS): *I* = 1913. MS (EI, 70 eV): *m*/*z* (%) = 272 (72), 257 (11), 243 (3), 229 (5), 216 (7), 202 (32), 189 (62), 17 6(51), 163 (37), 150 (77), 136 (76), 119 (100), 106 (97), 93 (92), 80 (83), 67 (47), 55 (48), 41 (50). IR (diamond ATR): ṽ = 2958 (s), 2924 (s), 2852 (s), 1664 (w), 1645 (w), 1454 (m), 1384 (m), 1362 (m), 1192 (w), 1139 (w), 1071 (w), 874 (m), 827 (m) cm^{-1}. ESI-(+)-HR-MS: calc. $[C_{20}H_{33}]^+$ *m*/*z* = 273.2577; found: *m*/*z* = 273.2577. Optical rotation: $[\alpha]_D^{25}$ = +123.1 (*c* 0.27, CH_2Cl_2). NMR data are given in Table S2.

Chryseojoostene B (2). TLC (pentane): R_f = 0.63. GC (HP-5MS): *I* = 1890. MS (EI, 70 eV): *m*/*z* (%) = 272 (62), 257 (51), 243 (16), 230 (25), 216 (44), 201 (18), 188 (15), 175 (18), 161 (23), 148 (70), 134 (76), 120 (86), 107 (83), 91 (100), 82 (92), 69 (59), 55 (66), 41 (64). IR (diamond ATR): ṽ = 2945 (s), 2928 (s), 2865 (s), 1738 (w), 1462 (m), 1366 (m), 1259 (w), 1216 (w), 1090 (w), 1024 (w), 875 (w) cm^{-1}. ESI-(+)-HR-MS: calc. $[C_{20}H_{33}]^+$ *m*/*z* = 273.2577; found: *m*/*z* = 273.2577. Optical rotation: $[\alpha]_D^{25}$ = +72.8 (*c* 0.08, CH_2Cl_2). NMR data are given in Table S3.

Chryseojoostene C (3). TLC (pentane): R_f = 0.60. GC (HP-5MS): *I* = 1977. MS (EI, 70 eV): *m*/*z* (%) = 272 (5), 257 (8), 243 (2), 229 (3), 215 (4), 202 (4), 191 (13), 176 (53), 161 (37), 146 (16), 135 (29), 121 (72), 106 (55), 95 (60), 91 (70), 81 (100), 65 (40), 55 (51), 41 (56). IR (diamond ATR): ṽ = 2948 (s), 2923 (s), 2859 (s), 1621 (w), 1450 (m), 1382 (w), 1363 (w), 1260 (w), 1108 (w), 1086 (w), 883 (m), 839 (w), 806 (w) cm^{-1}. ESI-(+)-HR-MS: calc. $[C_{20}H_{33}]^+$ *m*/*z* = 273.2577; found: *m*/*z* = 273.2577. Optical rotation: $[\alpha]_D^{25}$ = +1.5 (*c* 0.13, CH_2Cl_2). NMR data are given in Table S4.

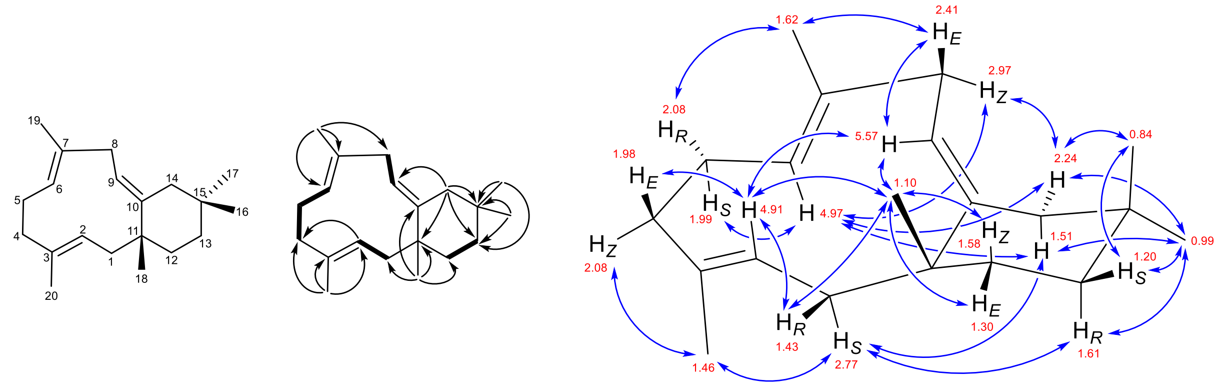

Figure S7. Structure elucidation of chryseojoostene A (**1**). Bold: ^{1}H,^{1}H-COSY correlations, single headed arrows: HMBC correlations, and blue double headed arrows: NOESY correlations. Carbon numbering follows GGPP numbering to indicate the origin of each carbon. The hydrogen labels H_R, H_S, H_E and H_Z indicate the results of stereoselective labelling experiments (Figures S39 and S40).

Table S2. NMR data of chryseojoostene A (**1**) in C_6D_6 recorded at 298 K.

C[a]	type	^{13}C[b]	^{1}H[b]
1	CH_2	36.06	2.77 (dd, *J* = 13.5, 12.0)<br>1.43 (dm, *J* = 13.5)
2	CH	125.17	4.91 (dm, *J* = 12.0)
3	C	132.21	–
4	CH_2	40.43	2.08 (m)<br>1.98 (m)
5	CH_2	23.97	2.08 (m)<br>1.99 (m)
6	CH	125.83	4.97 (m)
7	C	138.81	–
8	CH_2	35.67	2.97 (dd, *J* = 13.1, 10.5)<br>2.41 (dd, *J* = 13.0, 6.1)
9	CH	125.22	5.57 (ddd, *J* = 10.5, 6.1, 2.0)
10	C	140.65	–
11	C	39.88	–
12	CH_2	36.50	1.58 (m)<br>1.30 (m)
13	CH_2	35.54	1.61 (m)<br>1.20 (m)
14	CH_2	39.68	2.24 (dd, *J* = 13.8, 2.4)<br>1.51 (d, *J* = 14.0)
15	C	32.04	–
16	CH_3	24.81	0.84 (s)
17	CH_3	33.07	0.99 (s)
18	CH_3	26.17	1.10 (s)
19	CH_3	18.18	1.62 (s)
20	CH_3	15.13	1.46 (s)

[a] Carbon numbering as shown in Figure S7 indicates the origin of each carbon from GGPP by same number. [b] Chemical shifts δ in ppm, multiplicity: s = singlet, d = doublet, m = multiplet, coupling constants *J* are given in Hertz.

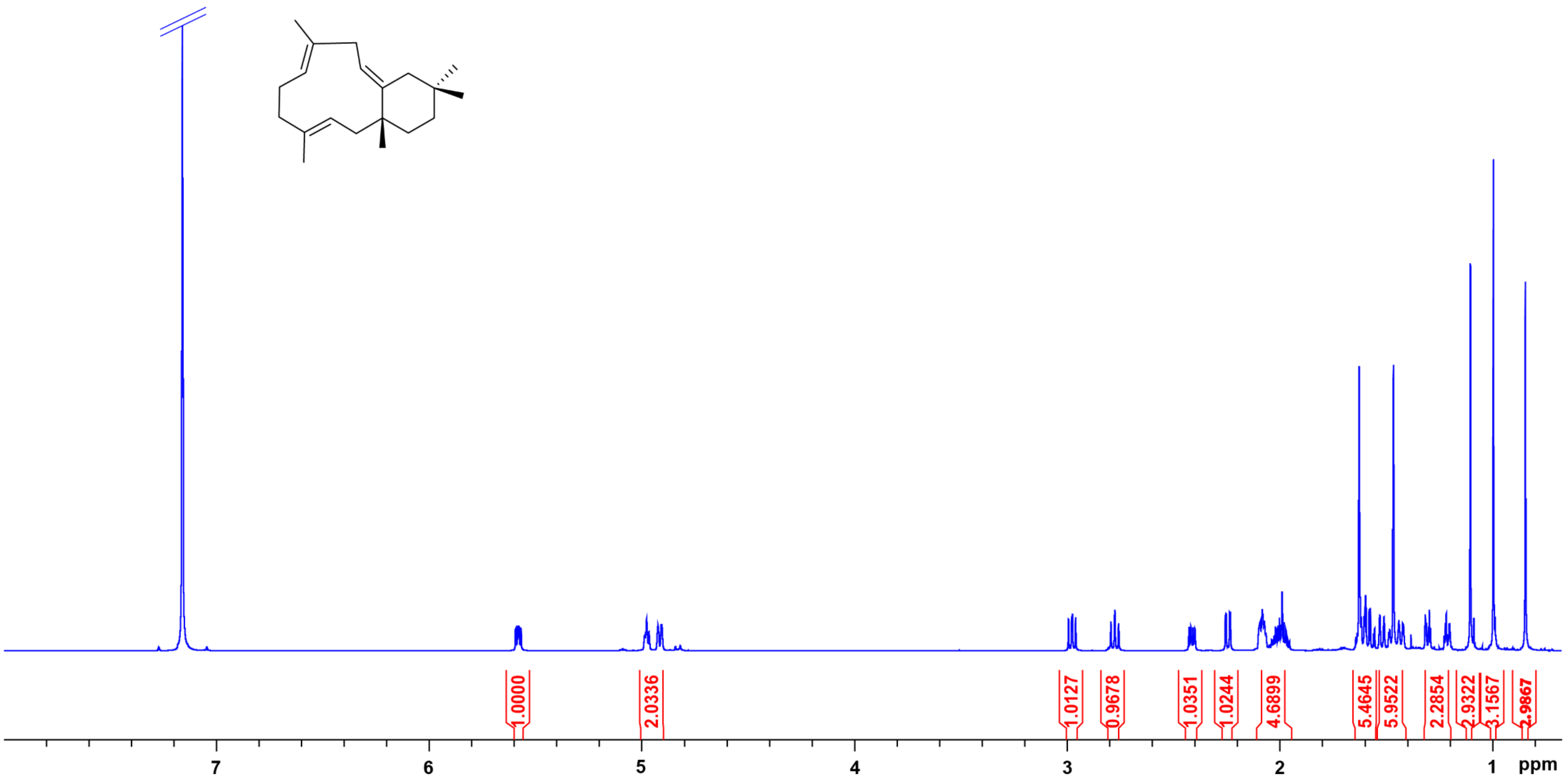

Figure S8. ^{1}H-NMR spectrum of **1** (700 MHz, C_6D_6).

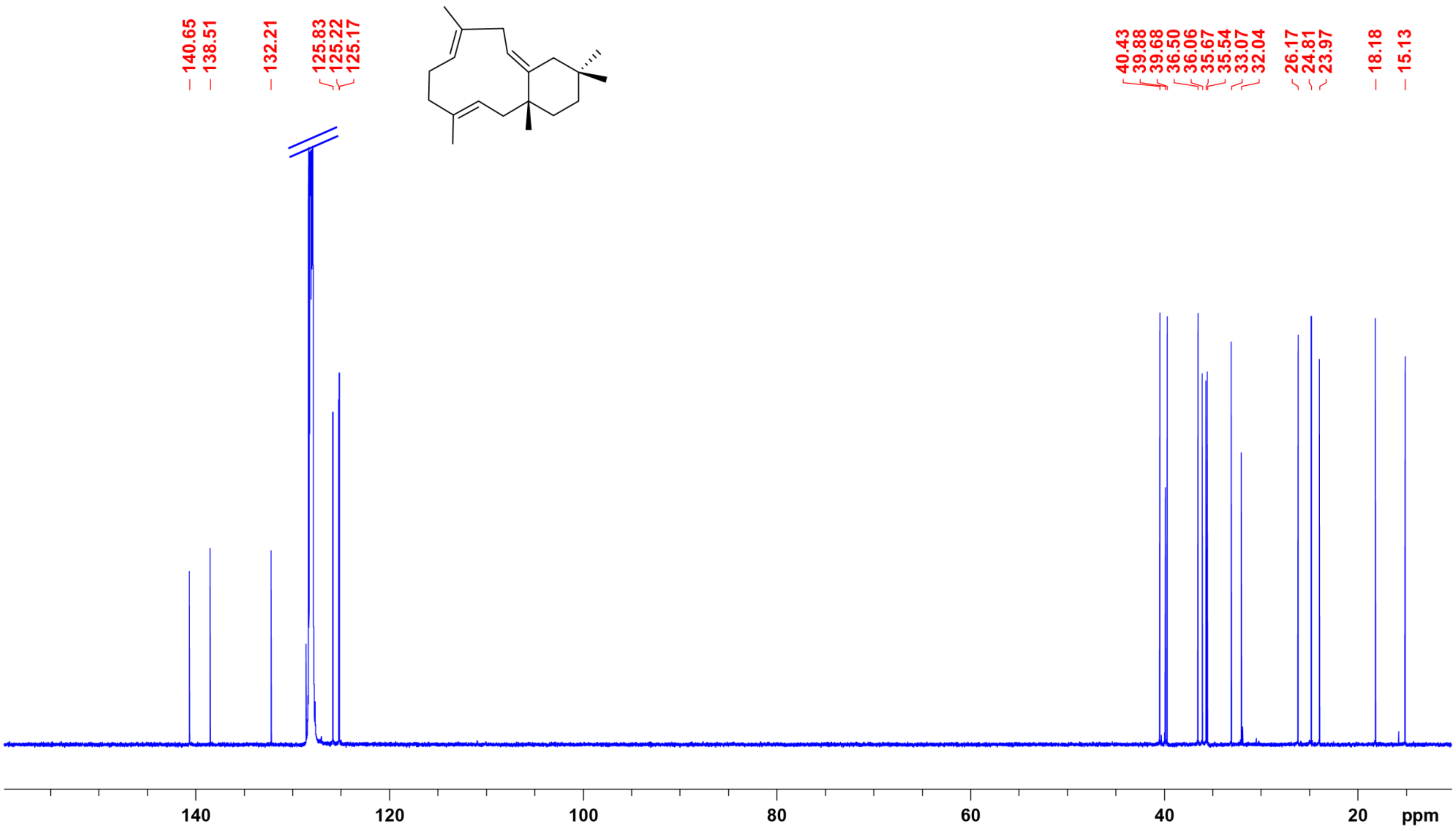

Figure S9. ^{13}C-NMR spectrum of **1** (176 MHz, C_6D_6).

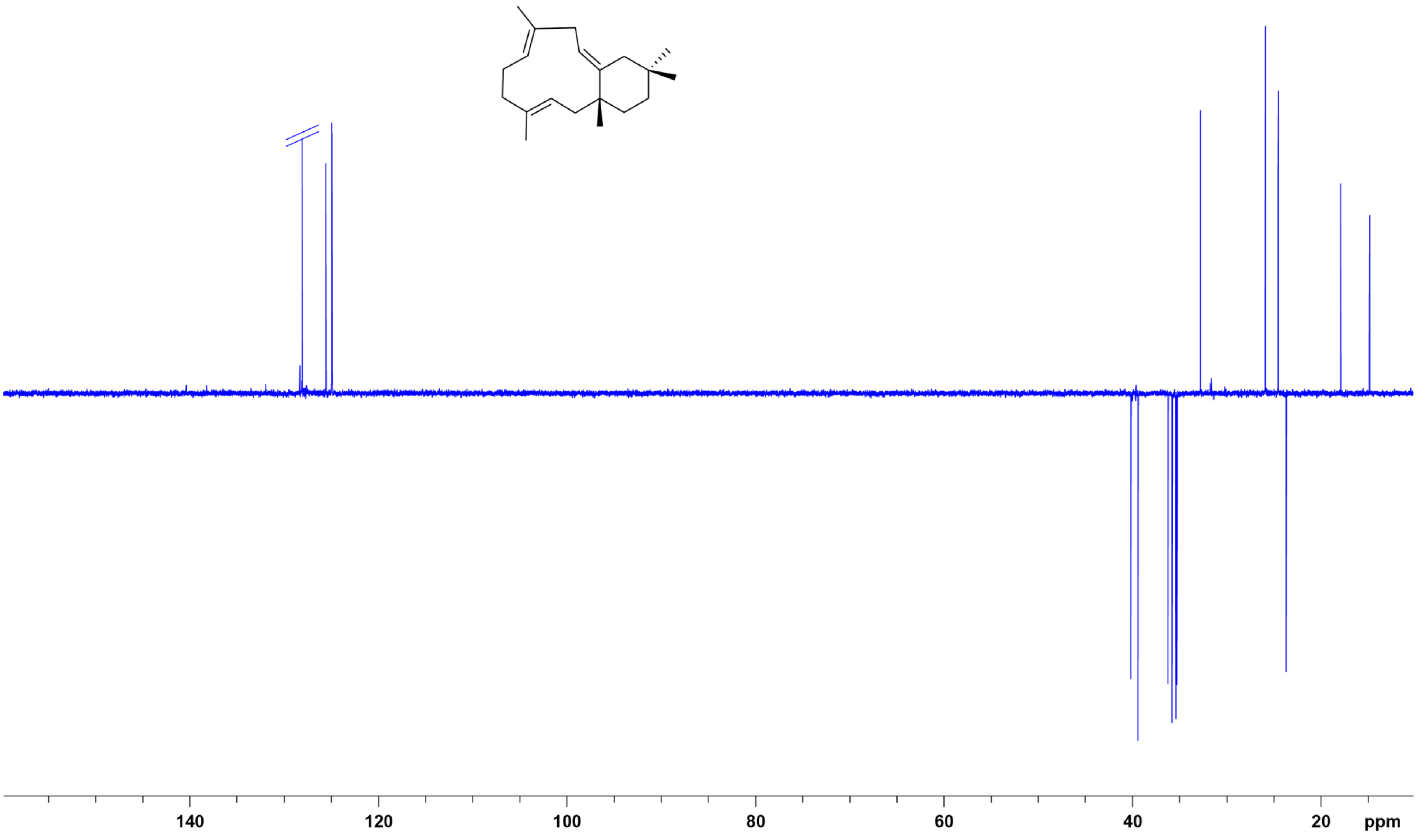

Figure S10. ^{13}C-DEPT135 spectrum of **1** (176 MHz, C_6D_6).

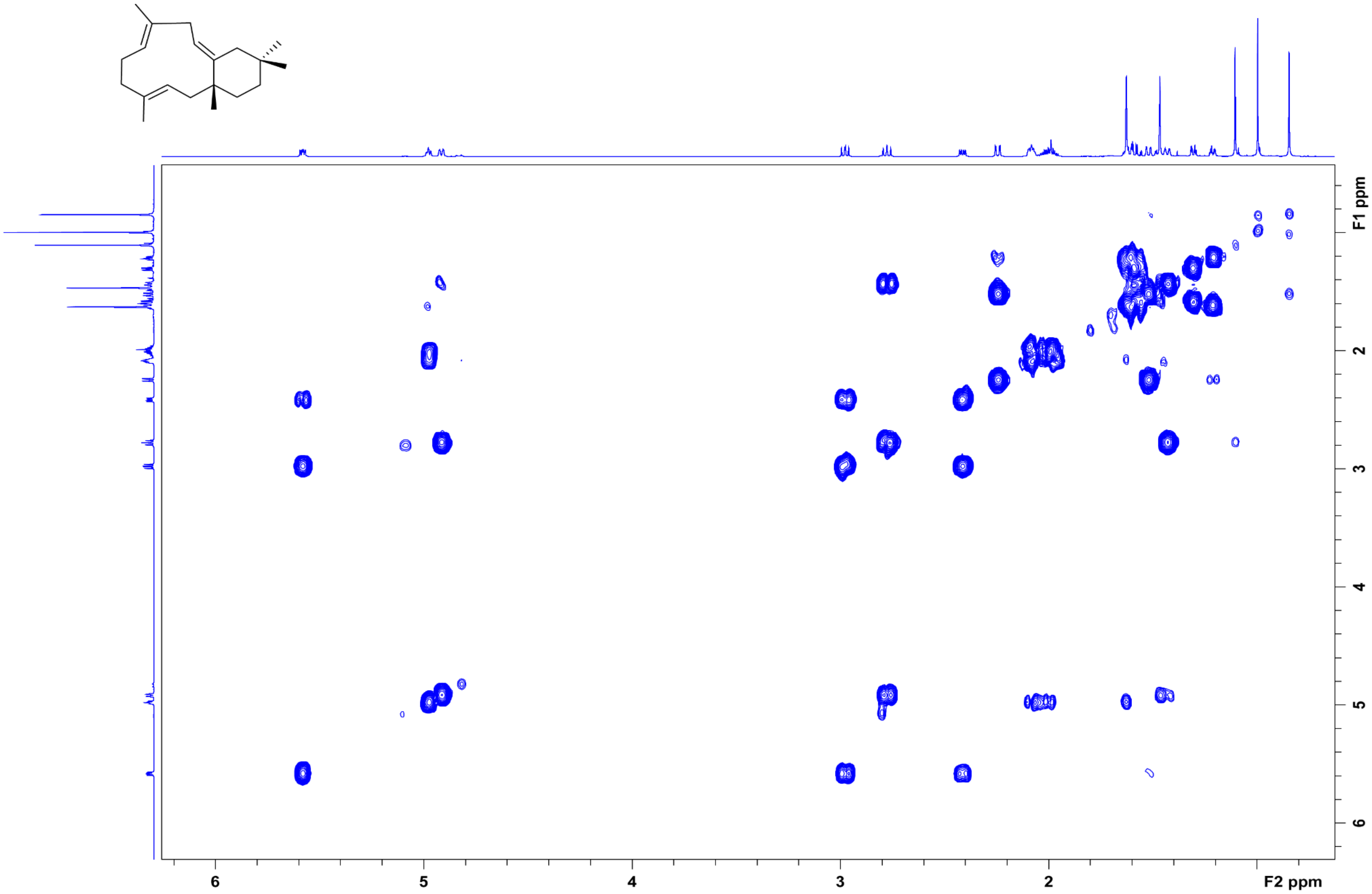

Figure S11. $^{1}H,^{1}H$-COSY spectrum (C_6D_6) of **1**.

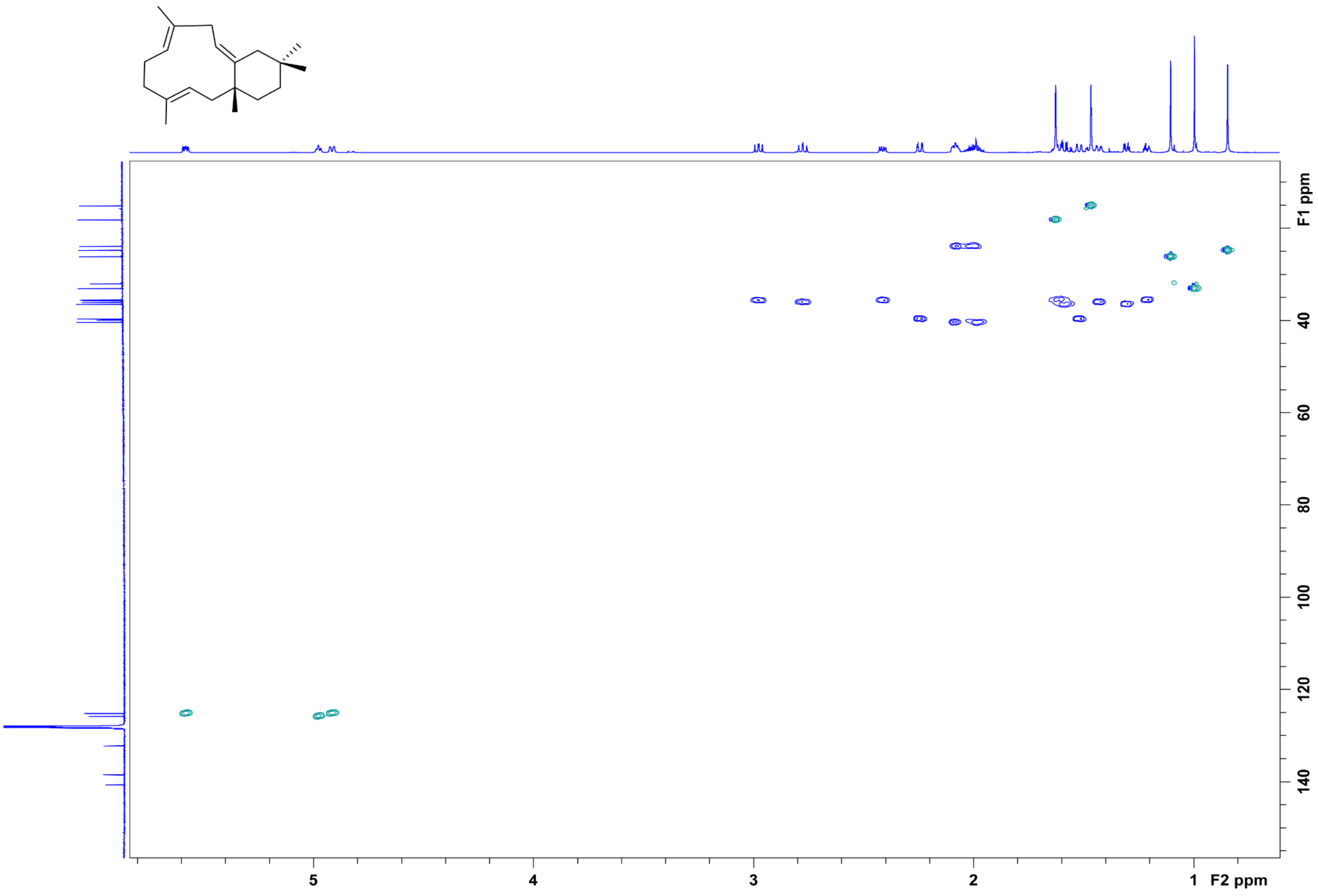

Figure S12. HSQC spectrum (C_6D_6) of **1**.

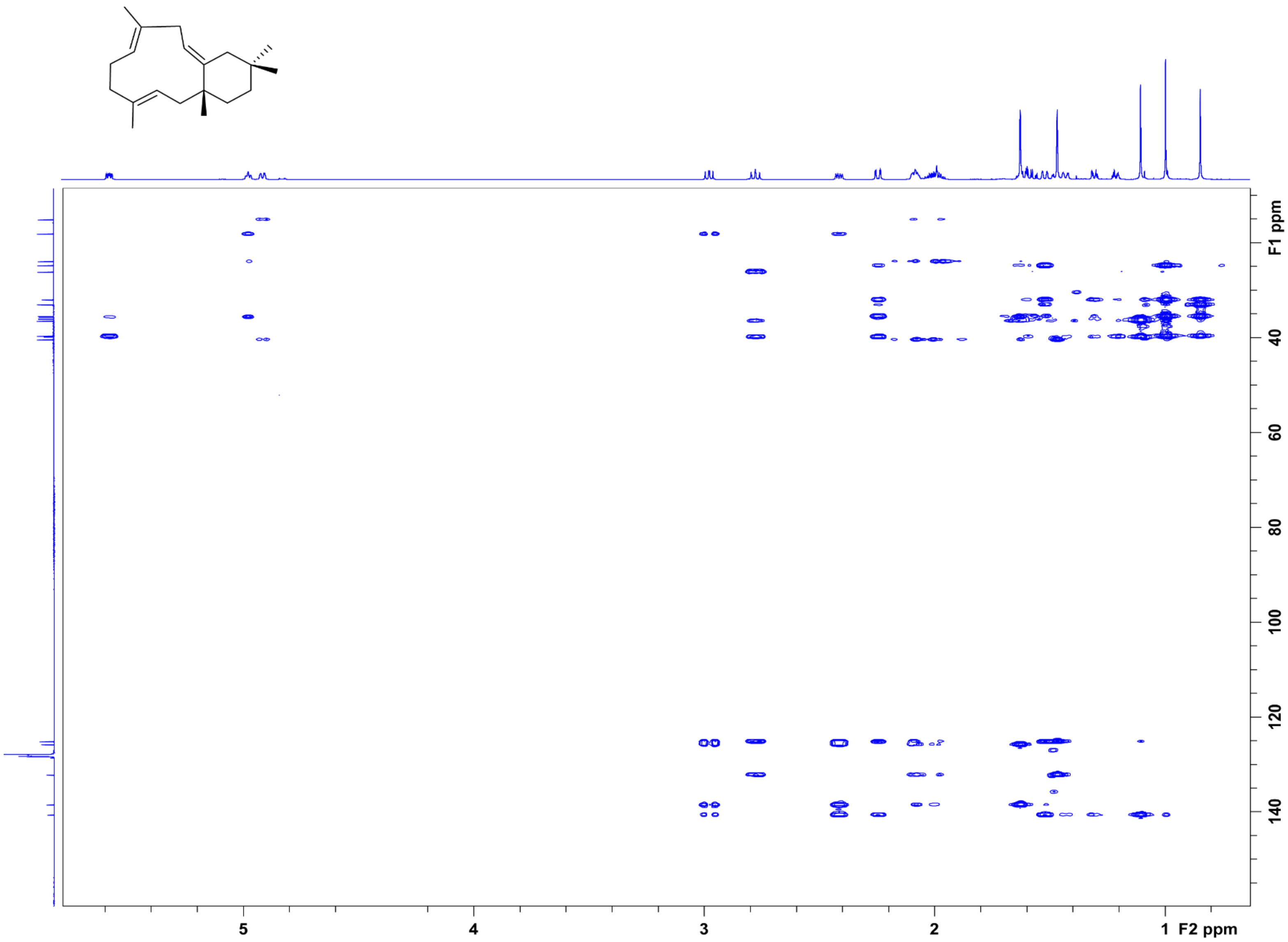

Figure S13. HMBC spectrum (C_6D_6) of **1**.

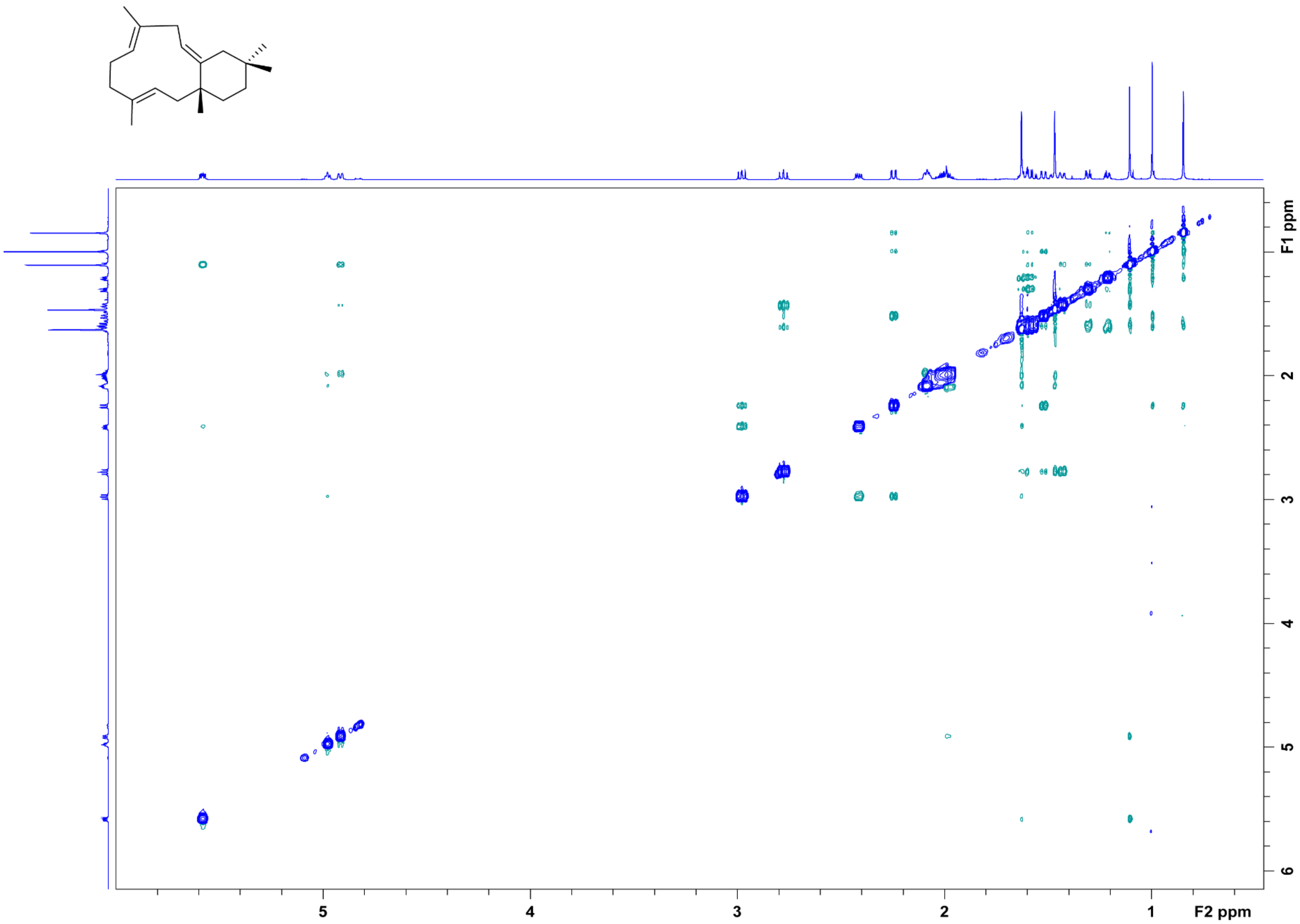

Figure S14. NOESY spectrum (C_6D_6) of **1**.

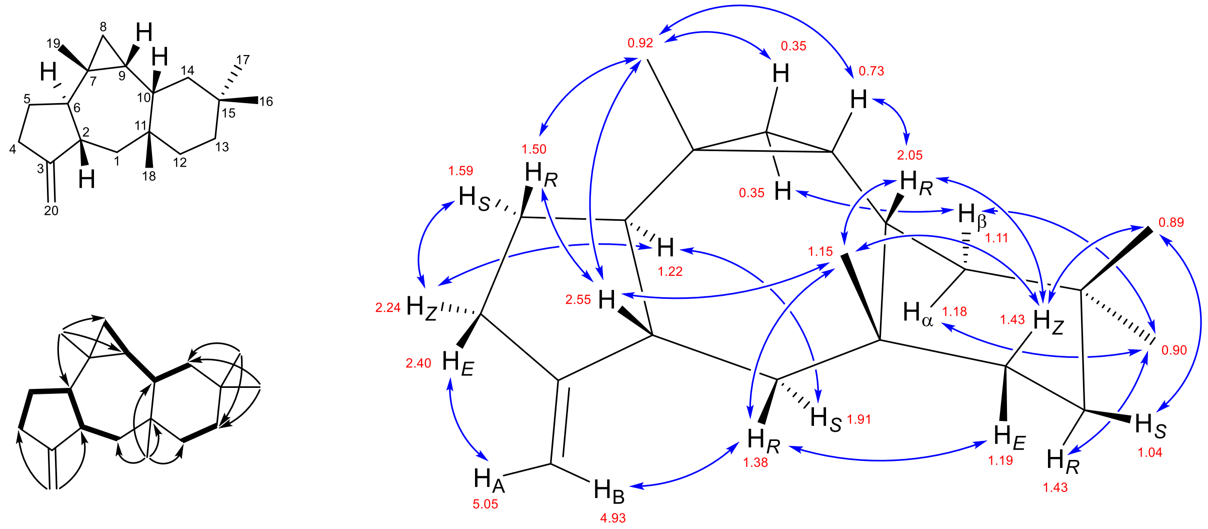

Figure S15. Structure elucidation of chryseojoostene B (**2**). Bold: ^{1}H,^{1}H-COSY correlations, single headed arrows: HMBC correlations, and blue double headed arrows: NOESY correlations. Carbon numbering follows GGPP numbering to indicate the origin of each carbon. The hydrogen labels H_R, H_S, H_E and H_Z indicate the results of stereoselective labelling experiments (Figures S41 and S42).

Table S3. NMR data of chryseojoostene B (**2**) in C_6D_6 recorded at 298 K.

C[a]	type	^{13}C[b]	^{1}H[b]
1	CH_2	41.31	1.91 (dd, J = 13.8, 12.0, H_S)<br>1.38 (dd, J = 14.0, 3.3, H_R)
2	CH	42.16	2.55 (tquin, J = 11.5, 2.5)
3	C	158.09	–
4	CH_2	33.94	2.40 (ddq, J = 16.0, 7.8, 1.3, H_E)<br>2.24 (m, H_Z)
5	CH_2	28.45	1.59 (m, H_S)<br>1.50 (dq, J = 12.0, 7.9, H_R)
6	CH	52.99	1.24 (m)
7	C	19.94	–
8	CH_2	18.76	0.35 (d, J = 7.7, 2H)
9	CH	26.98	0.73 (m, H_R)
10	CH	39.46	2.05 (ddd, J = 14.3, 8.9, 3.1)
11	C	36.89	–
12	CH_2	40.50	1.42 (m, H_Z)<br>1.19 (m, H_E)
13	CH_2	34.70	1.43 (m, H_R)<br>1.04 (m, H_S)
14	CH_2	42.27	1.18 (m, H_α)<br>1.11 (m, H_β)
15	C	31.73	–
16	CH_3	25.58	0.89 (s)
17	CH_3	33.77	0.90 (s)
18	CH_3	27.69	1.15 (s)
19	CH_3	21.43	0.92 (s)
20	CH_2	105.30	5.05 (m, H_A)<br>4.93 (m, H_B)

[a] Carbon numbering as shown in Figure S15 indicates the origin of each carbon from GGPP by same number. [b] Chemical shifts δ in ppm, multiplicity: s = singlet, d = doublet, t = triplet, q = quartet, quin = quintet, m = multiplet, coupling constants J are given in Hertz.

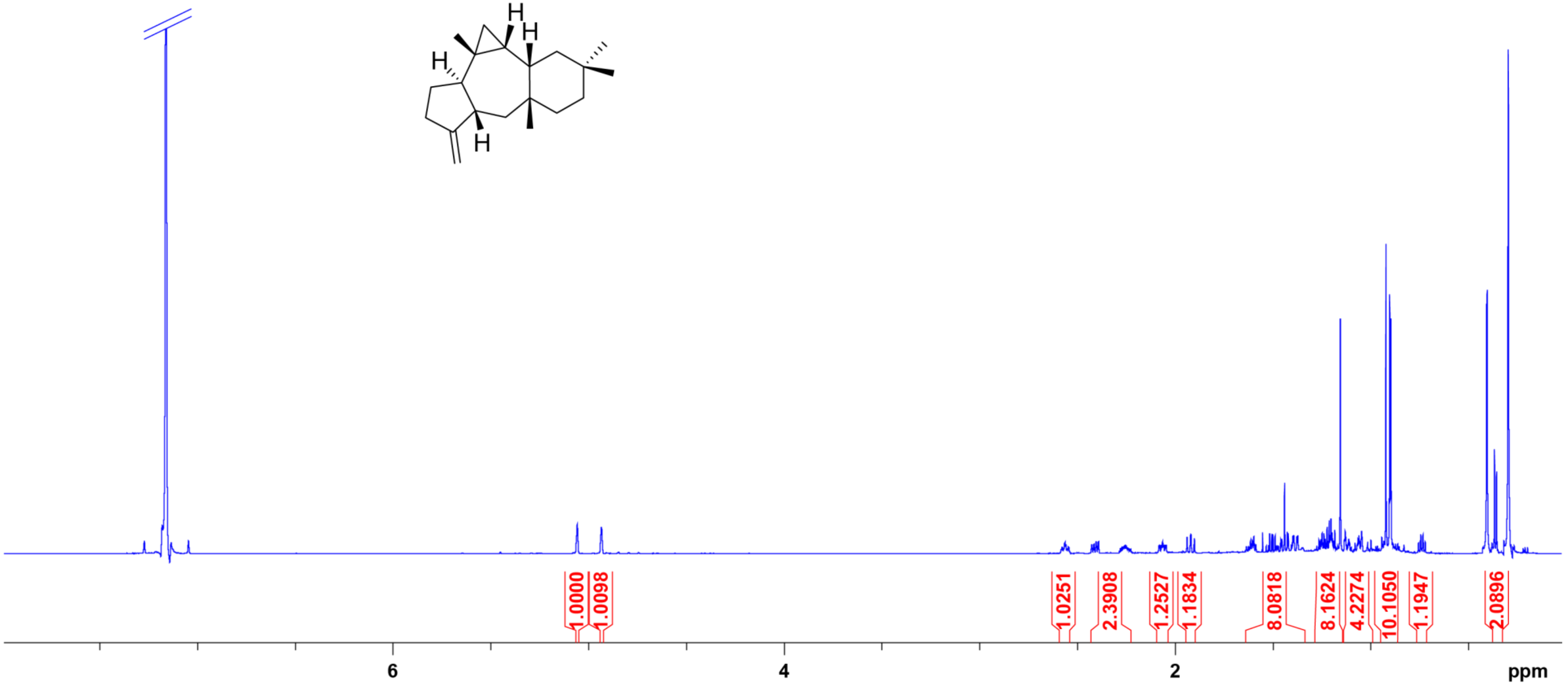

Figure S16. ^{1}H-NMR spectrum of **2** (700 MHz, C_6D_6).

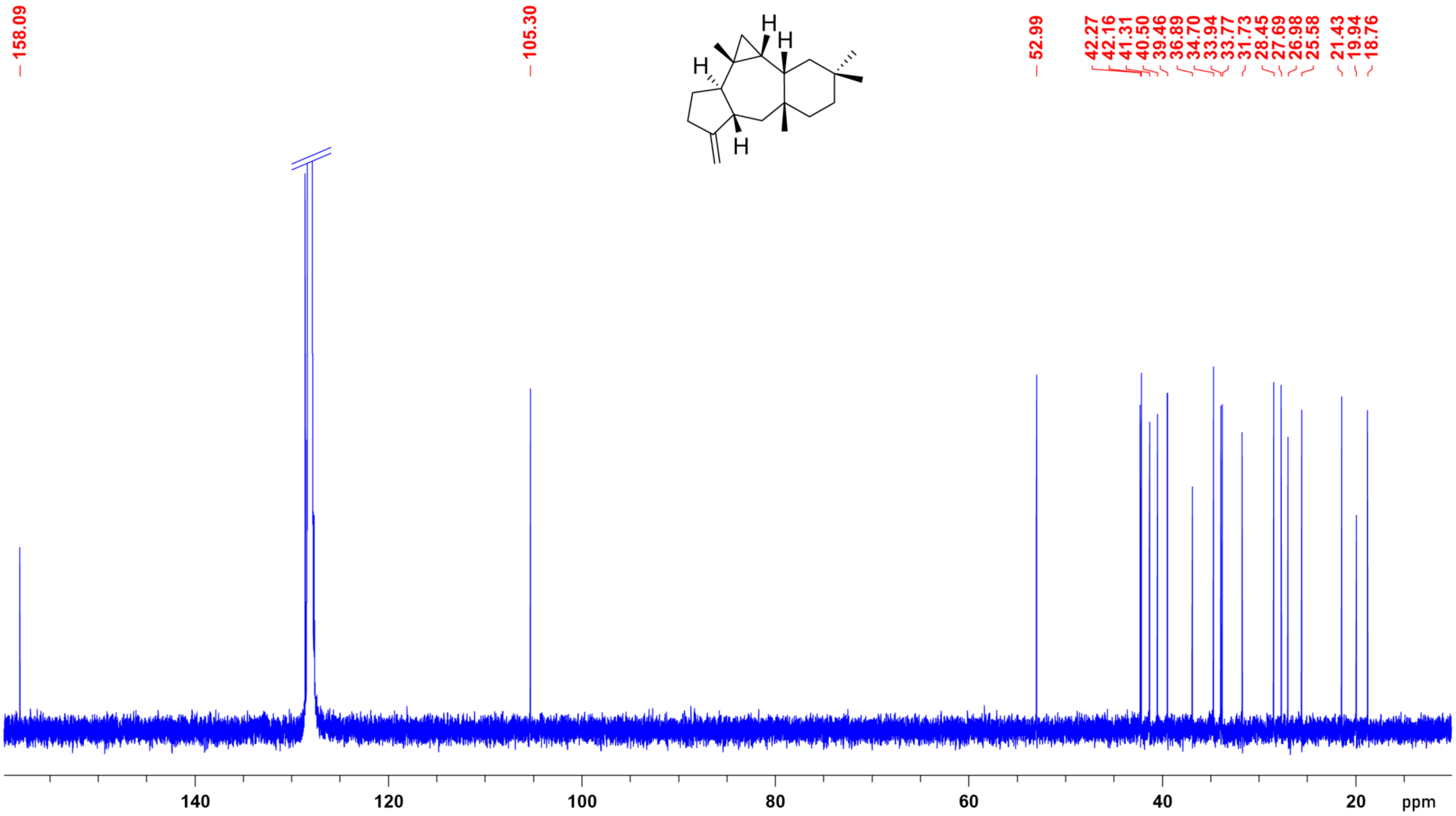

Figure S17. ^{13}C-NMR spectrum of **2** (176 MHz, C_6D_6).

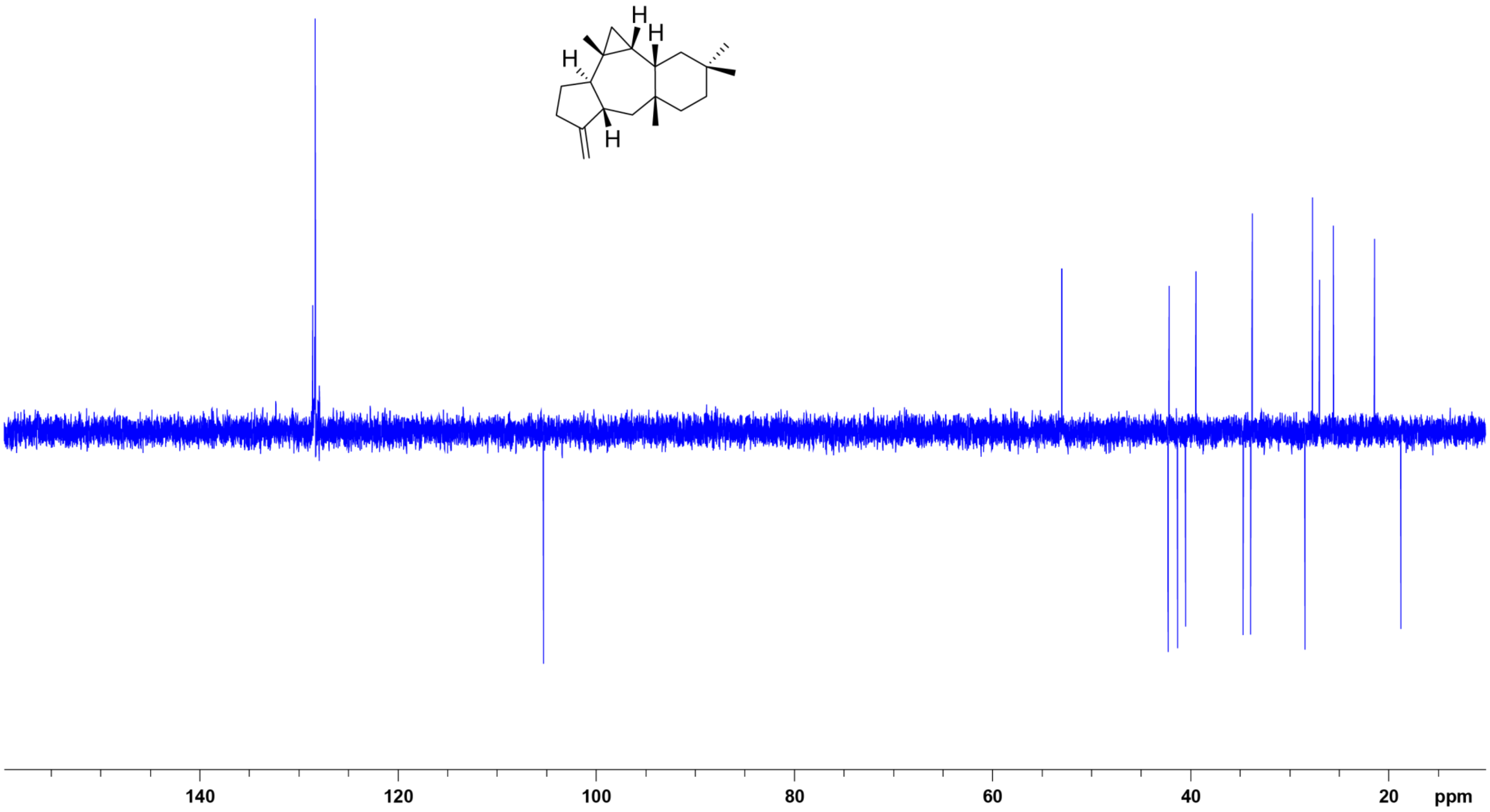

Figure S18. ^{13}C-DEPT135 spectrum of **2** (176 MHz, C_6D_6).

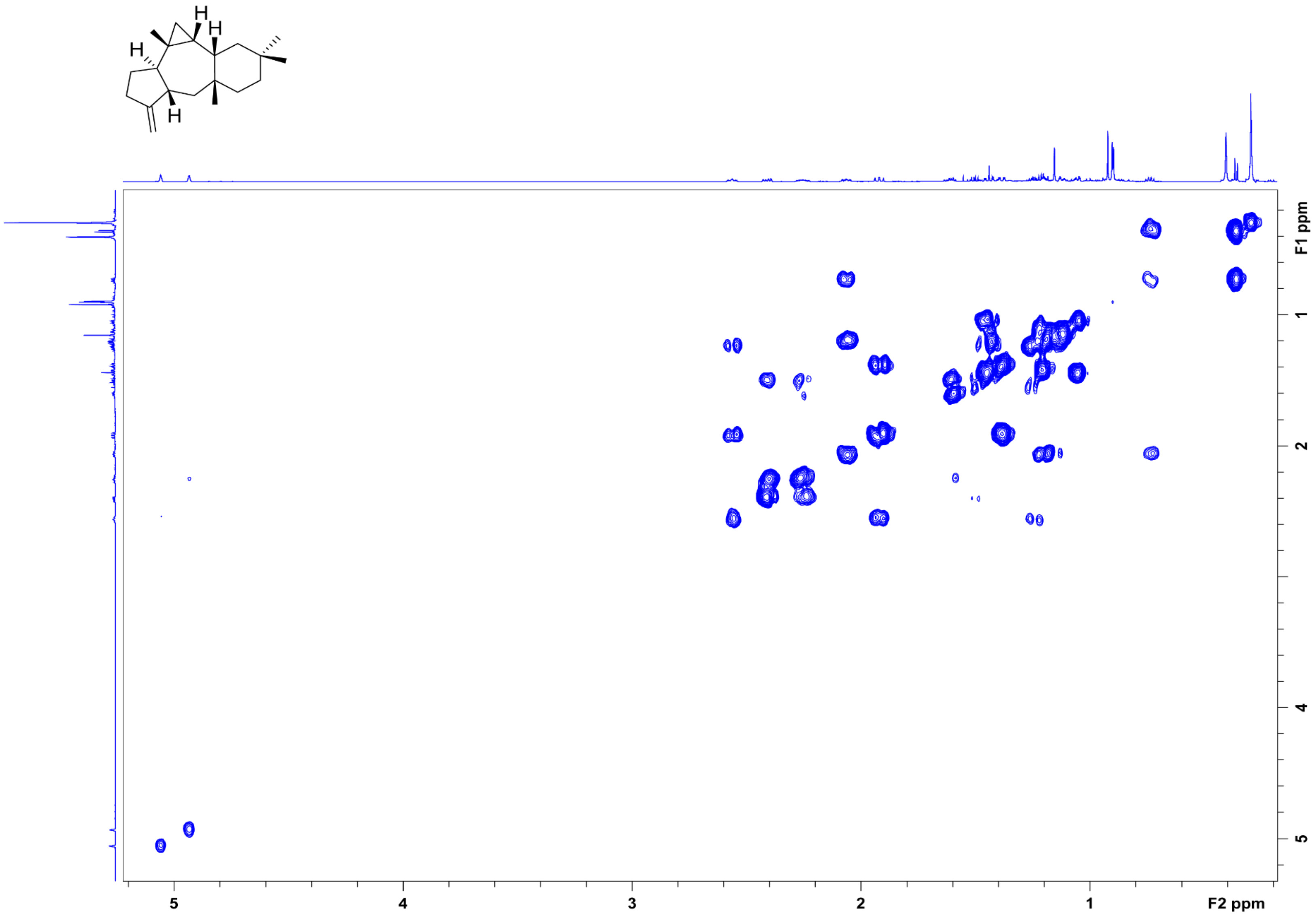

Figure S19. $^{1}H,^{1}H$-COSY spectrum (C_6D_6) of **2**.

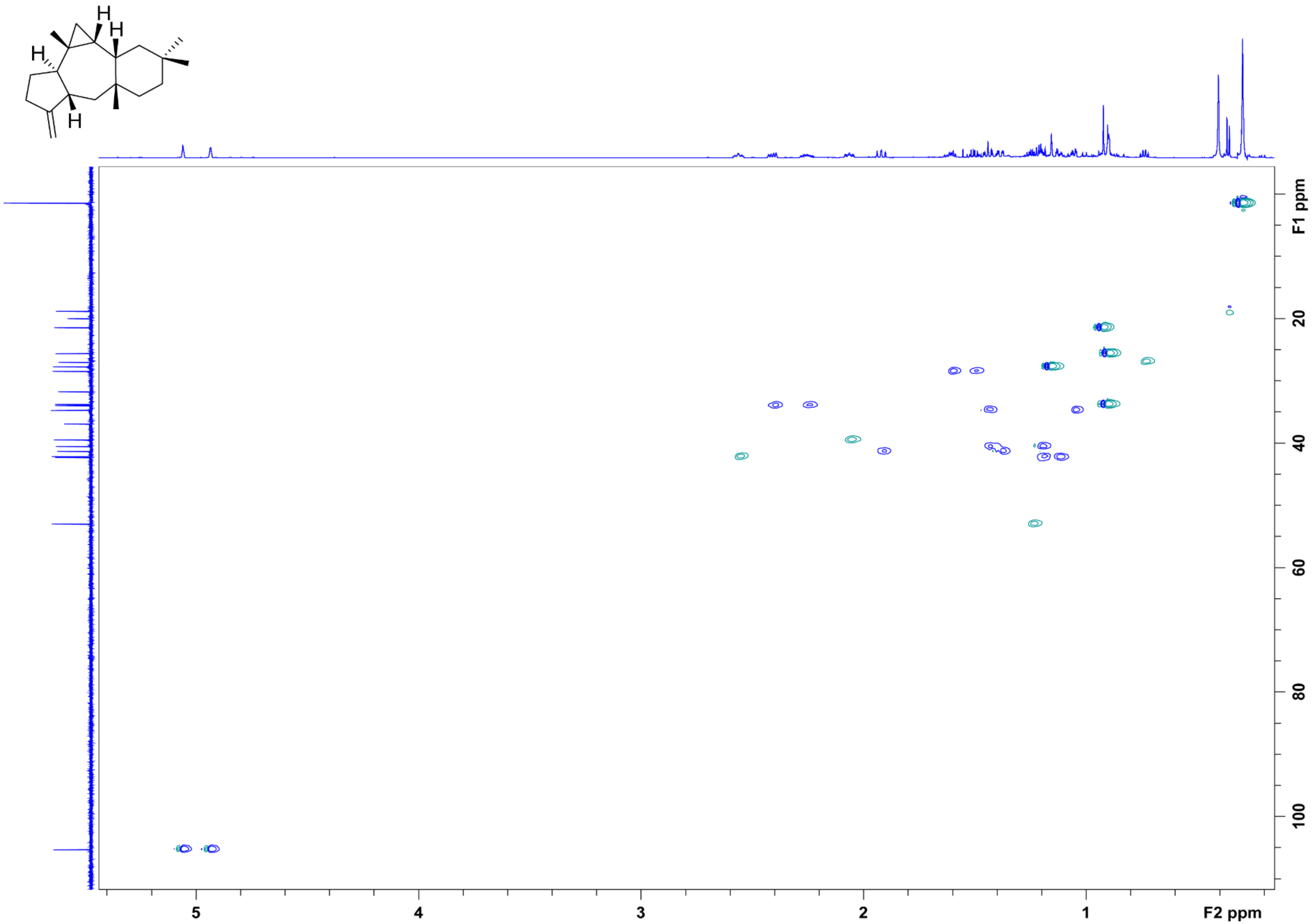

Figure S20. HSQC spectrum (C_6D_6) of **2**.

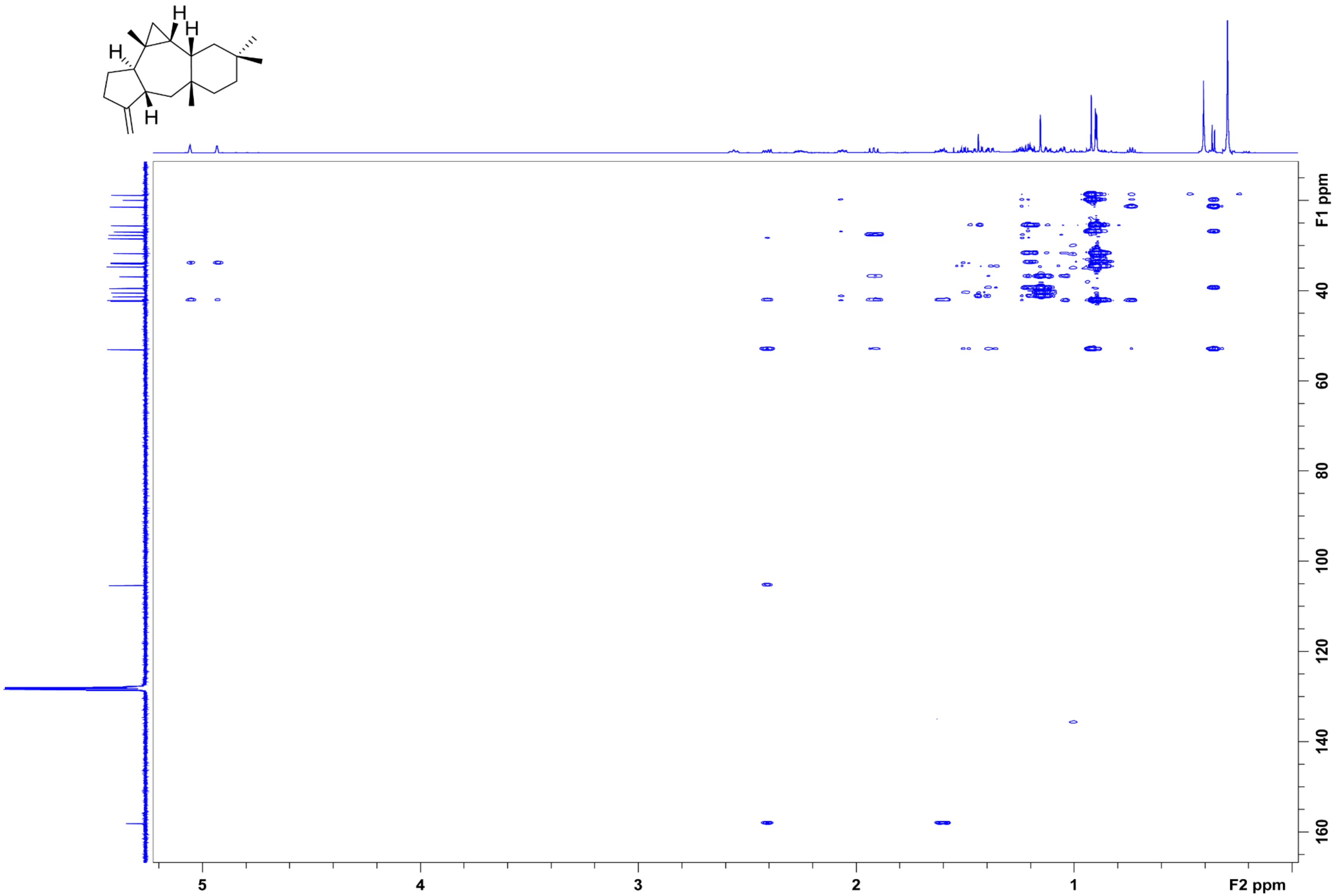

Figure S21. HMBC spectrum (C_6D_6) of **2**.

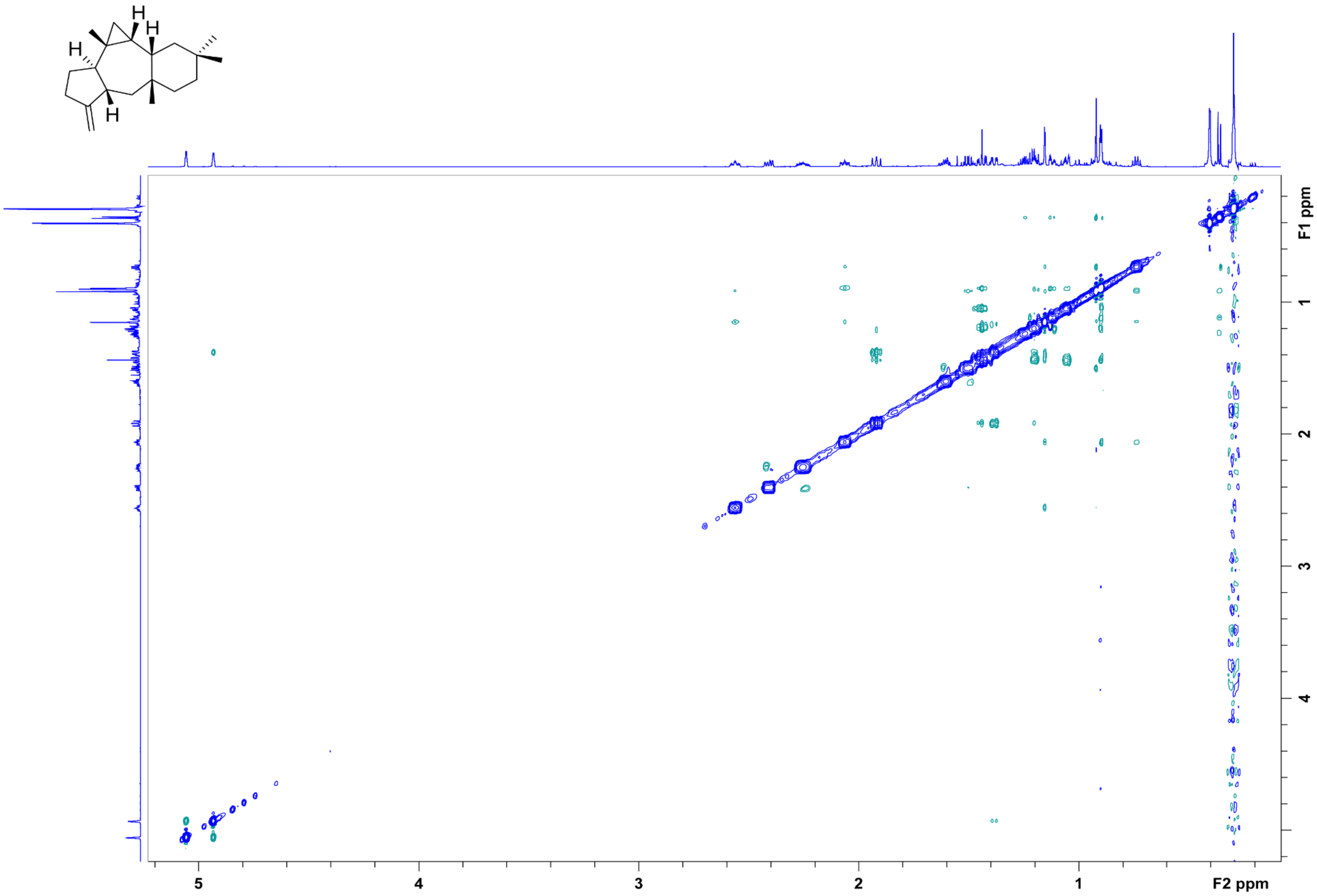

Figure S22. NOESY spectrum (C_6D_6) of **2**.

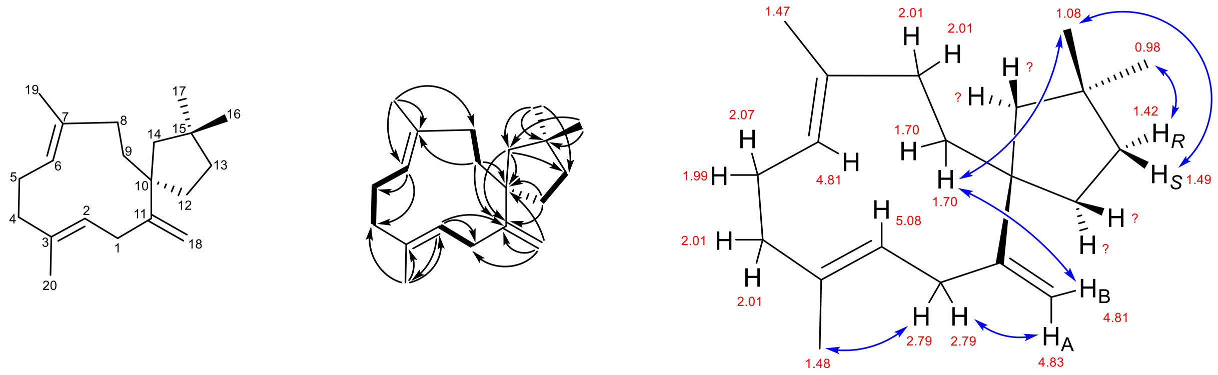

Figure S23. Structure elucidation of chryseojoostene C (**3**). Bold: ^{1}H,^{1}H-COSY correlations, single headed arrows: HMBC correlations, and blue double headed arrows: NOESY correlations. Carbon numbering follows GGPP numbering to indicate the origin of each carbon. Question marks indicate missing signals due to peak broadening. The hydrogen labels H_R and H_S indicate the results of stereoselective labelling experiments (Figure S43).

Table S4. NMR data of chryseojoostene C (**3**) in C_6D_6 recorded at 298 K.

C[a]	type	^{13}C[b]	^{1}H[b]
1	CH_2	34.80*	2.79 (m, 2H)
2	CH	127.12*	5.08 (t, *J* = 8.4)
3	C	132.58	–
4	CH_2	39.94	2.01 (m, 2H)
5	CH_2	25.01*	2.07 (m)
			1.99 (m)
6	CH	127.01	4.81 (m)
7	C	135.81*	–
8	CH_2	35.99*	2.01 (m, 2H)
9	CH_2	38.05*	1.70 (m, 2H)
10	C	52.21*	–
11	C	155.90	–
12	CH_2	37.85*	(?)
13	CH_2	40.28	1.49 (m)
			1.42 (m)
14	CH_2	(?)	(?)
15	C	37.72*	–
16	CH_3	31.89	1.08 (s)
17	CH_3	32.05	0.98 (s)
18	CH_2	110.92	4.83 (br s, H_A)
			4.81 (br s, H_B)
19	CH_3	16.79*	1.47 (d, *J* = 1.0)
20	CH_3	15.78	1.48 (d, *J* = 1.2)

[a] Carbon numbering as shown in Figure S23 indicates the origin of each carbon from GGPP by same number. [b] Chemical shifts δ in ppm, multiplicity: s = singlet, d = doublet, t = triplet, m = multiplet, br = broad, coupling constants *J* are given in Hertz. Asterisks indicate carbons that show peak broadening, (?) indicates signals that are missing due to peak broadening.

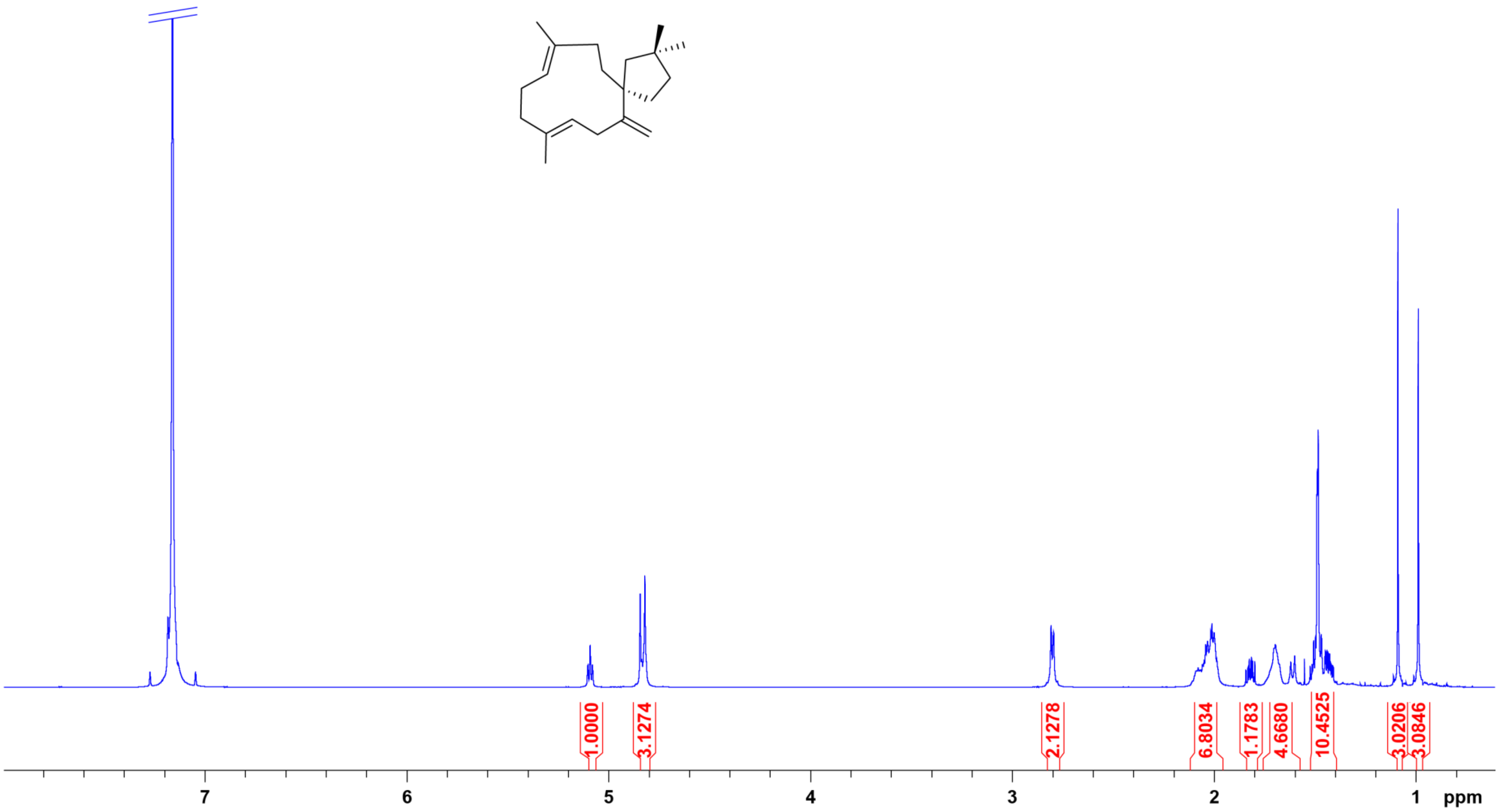

Figure S24. ^{1}H-NMR spectrum of **3** (700 MHz, C_6D_6).

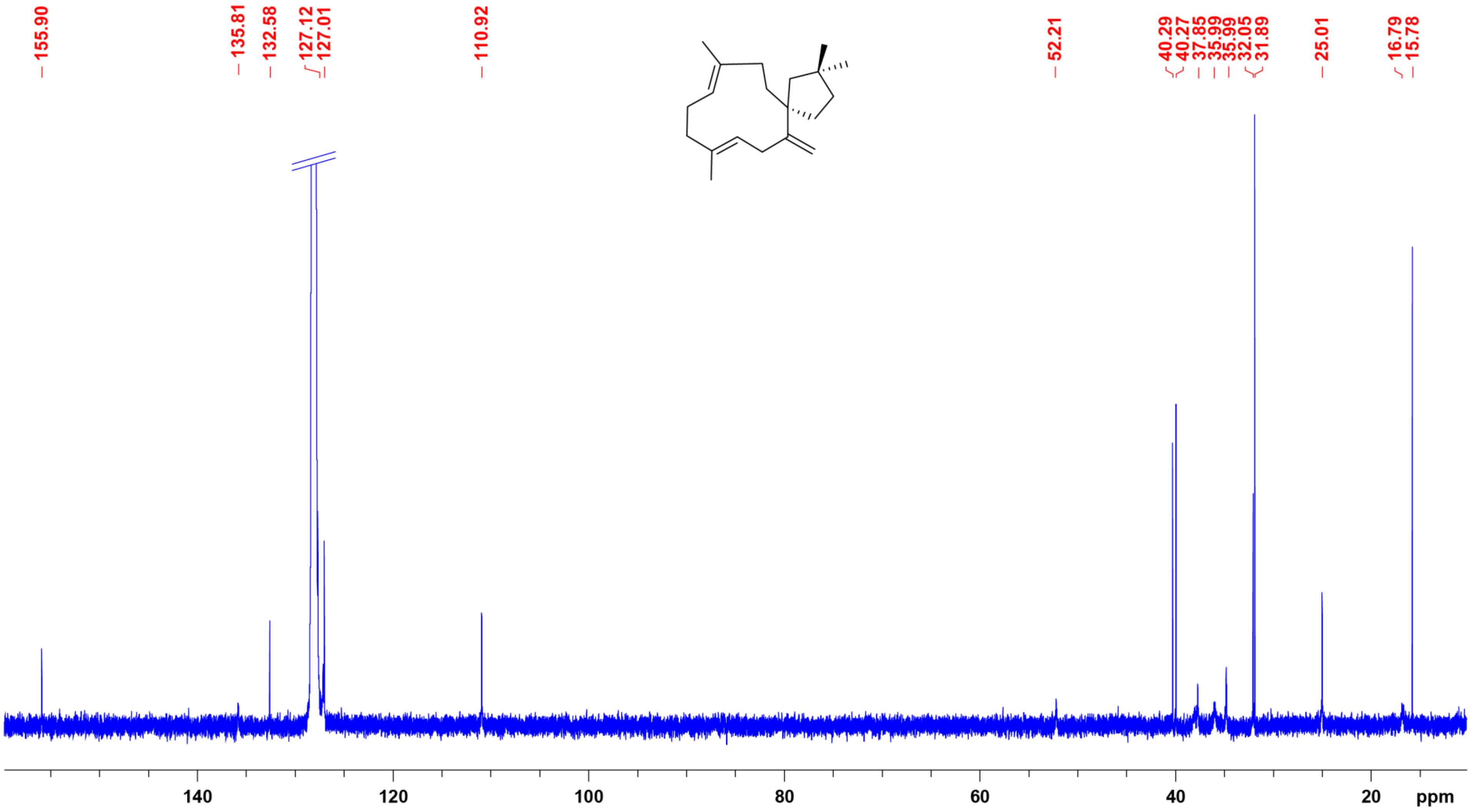

Figure S25. ^{13}C-NMR spectrum of **3** (176 MHz, C_6D_6).

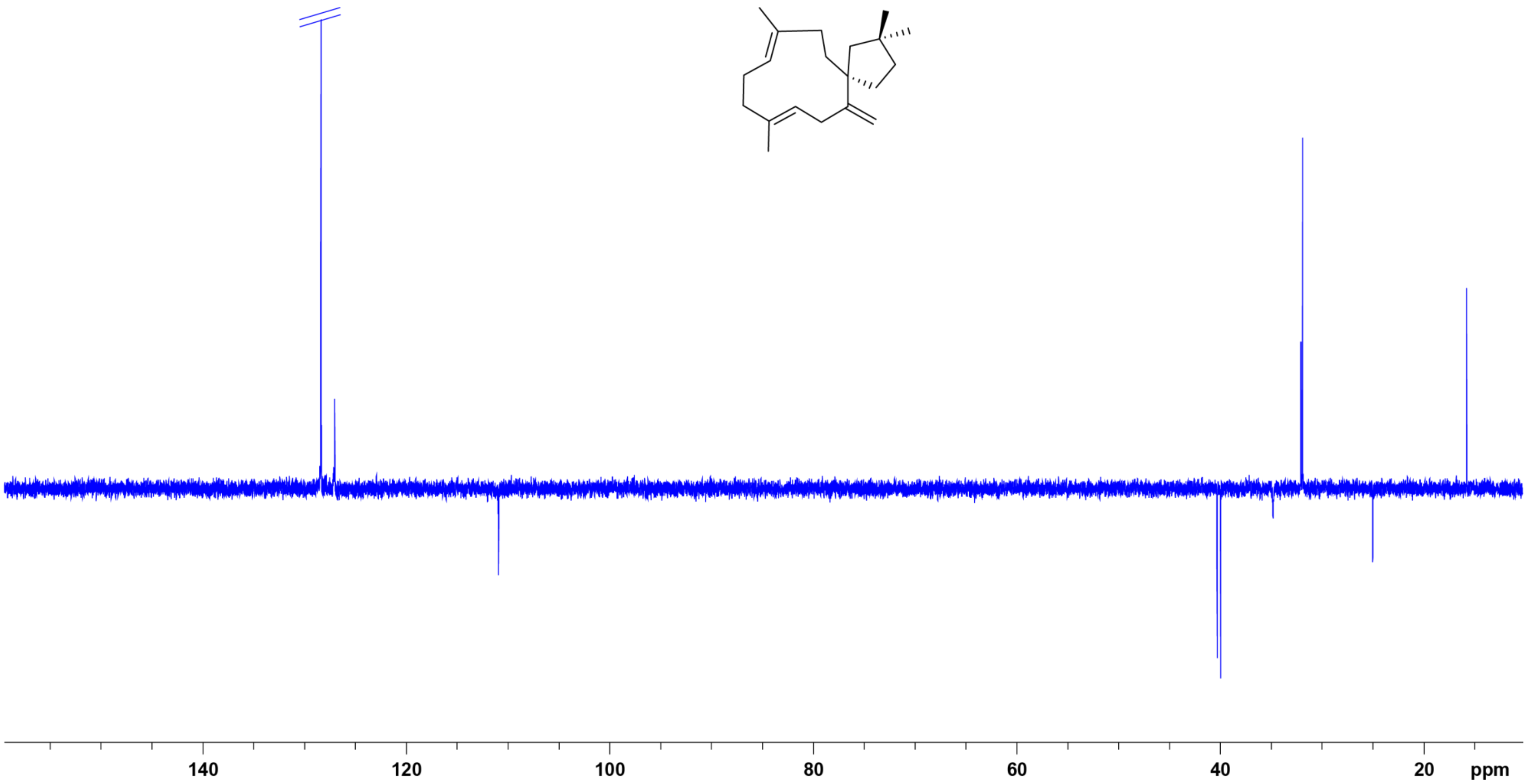

Figure S26. ^{13}C-DEPT135 spectrum of **3** (176 MHz, C_6D_6).

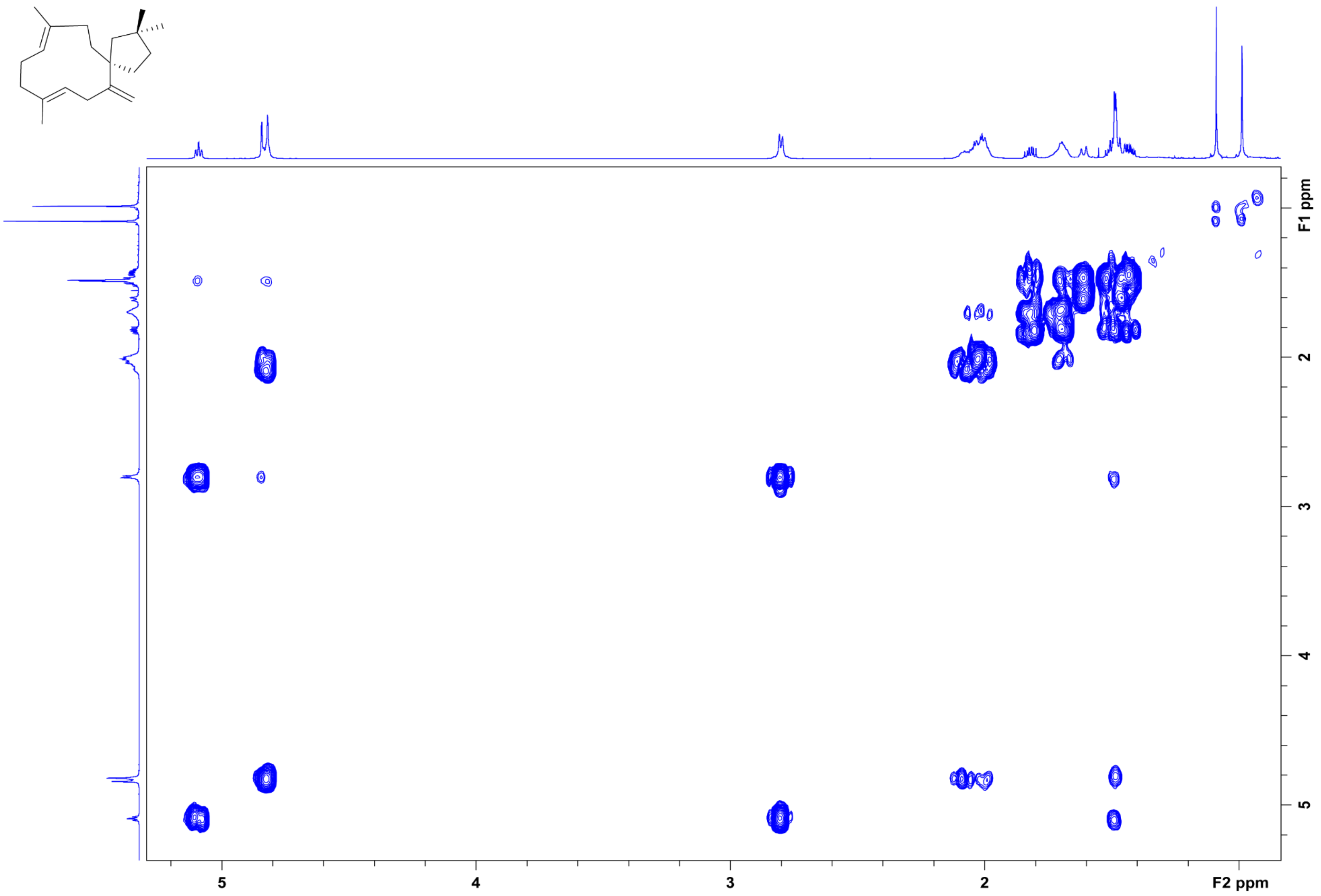

Figure S27. $^1H,^1H$-COSY spectrum (C_6D_6) of **3**.

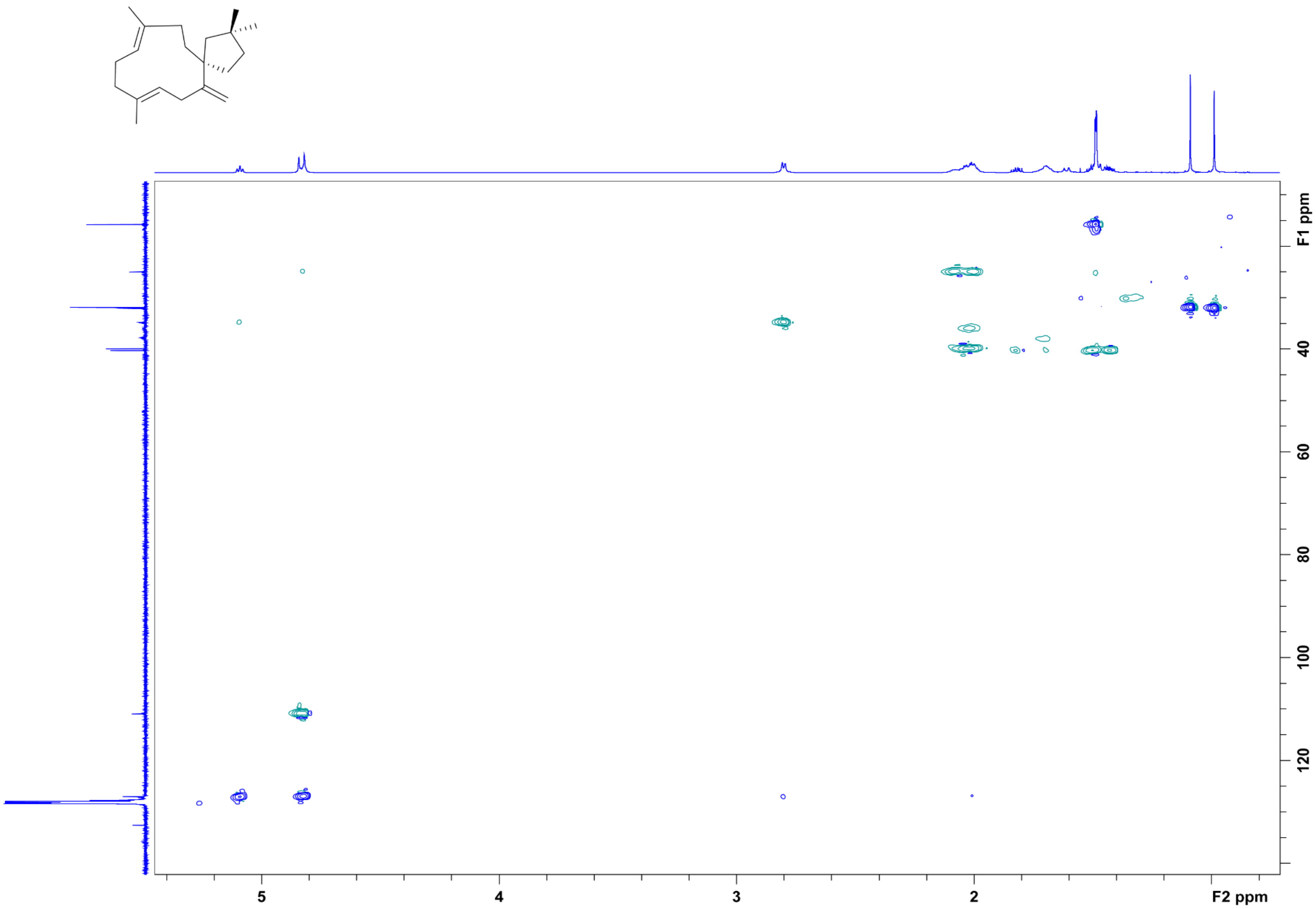

Figure S28. HSQC spectrum (C_6D_6) of **3**.

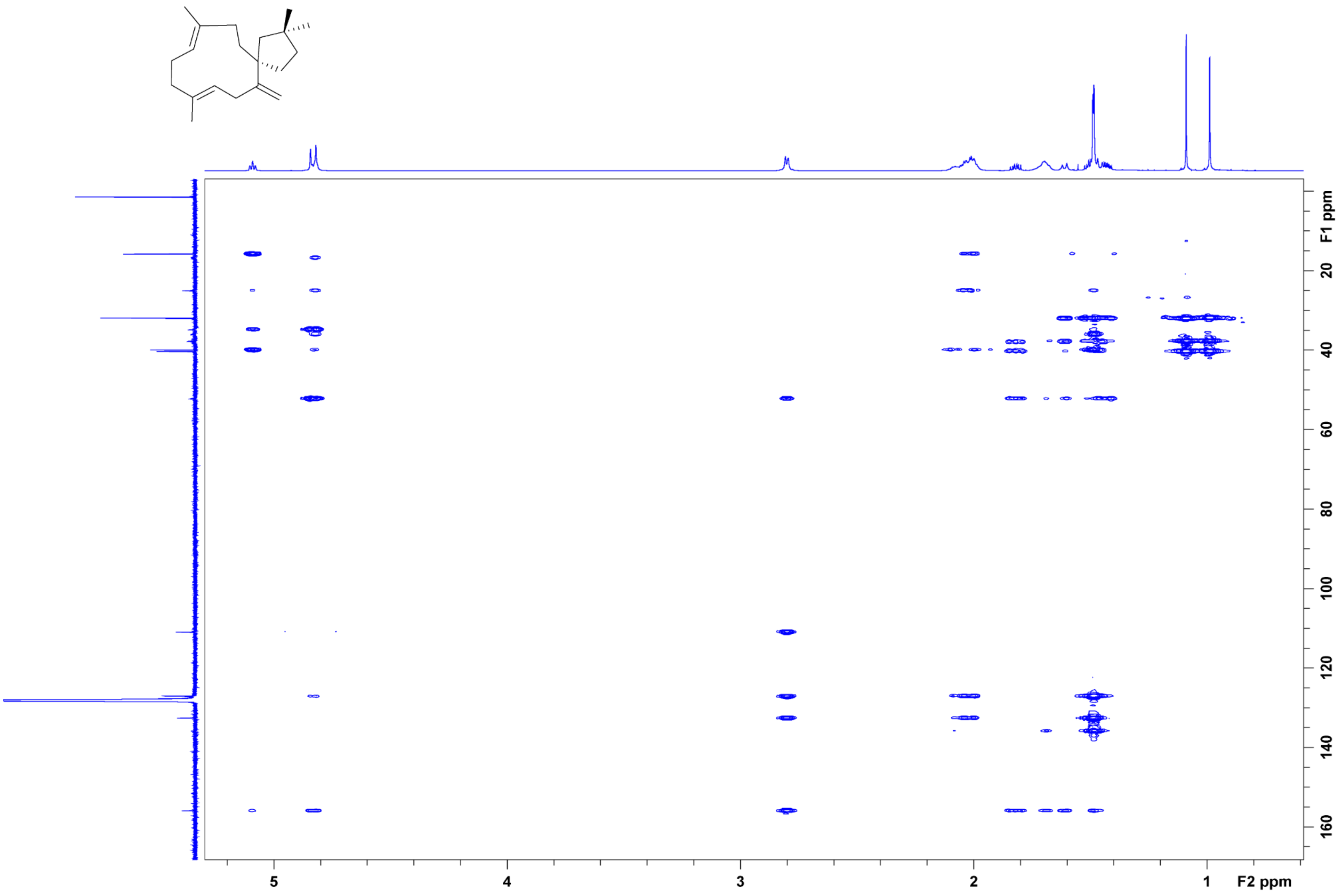

Figure S29. HMBC spectrum (C_6D_6) of **3**.

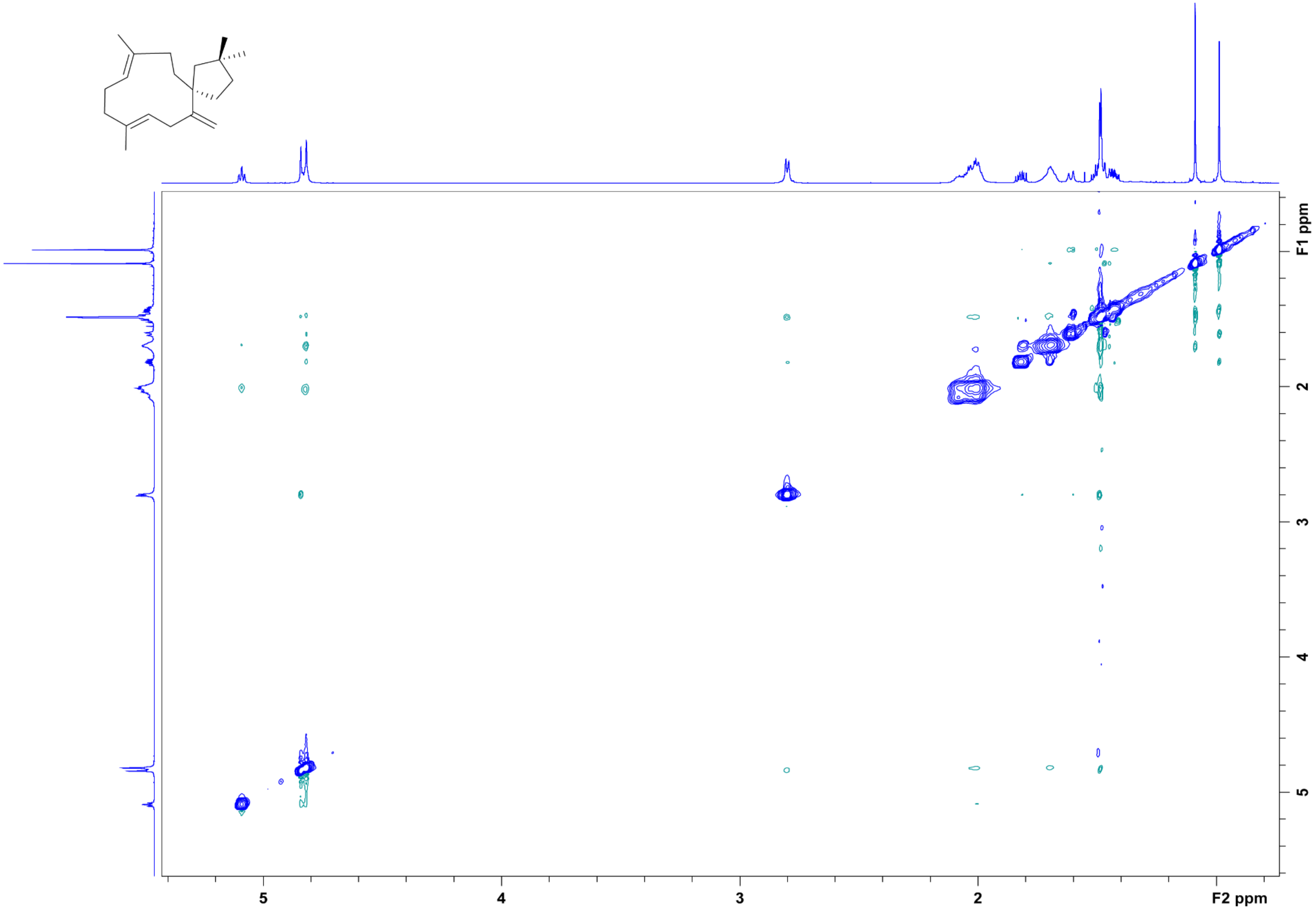

Figure S30. NOESY spectrum (C_6D_6) of **3**.

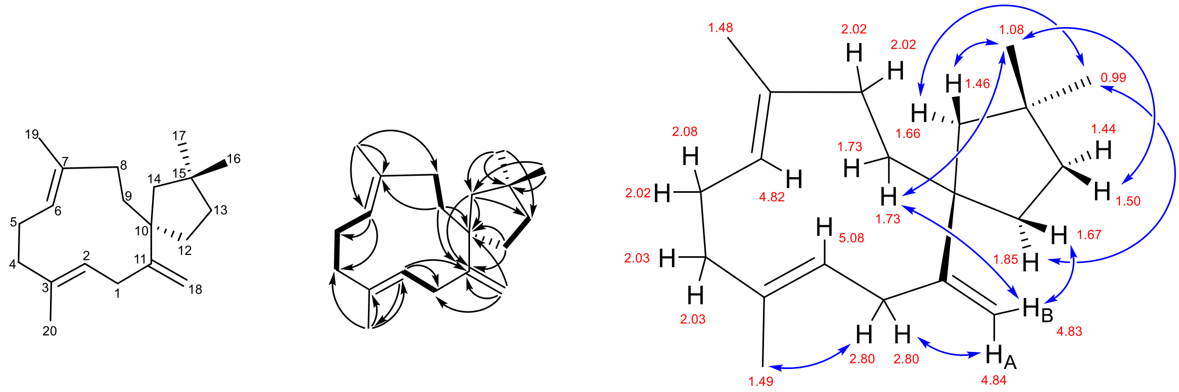

Figure S31. Structure elucidation of chryseojoostene C (**3**). Bold: ^{1}H,^{1}H-COSY correlations, single headed arrows: HMBC correlations, and blue double headed arrows: NOESY correlations. Carbon numbering follows GGPP numbering to indicate the origin of each carbon.

Table S5. NMR data of chryseojoostene C (**3**) in C_6D_6 recorded at 343 K.

C[a]	type	^{13}C[b]	^{1}H[b]
1	CH_2	34.82	2.80 (m, 2H)
2	CH	127.55	5.08 (t, *J* = 8.1)
3	C	132.42	–
4	CH_2	39.95	2.03 (m, 2H)
5	CH_2	25.03	2.08 (m)
			2.02 (m)
6	CH	127.07	4.82 (m)
7	C	135.98	–
8	CH_2	35.83*	2.02 (m, 2H)
9	CH_2	39.00*	1.73 (m, 2H)
10	C	52.65	–
11	C	156.12	–
12	CH_2	36.49	1.85 (ddd, *J* = 12.8, 10.6, 7.1)
			1.67 (dddd, *J* = 12.7, 6.5, 3.7, 1.2)
13	CH_2	40.48	1.50 (m)
			1.44 (ddd, *J* = 12.4, 7.0, 3.7)
14	CH_2	54.91*	1.66 (d, *J* = 13.5)
			1.46 (d, *J* = 13.8)
15	C	38.04	–
16	CH_3	31.82	1.08 (s)
17	CH_3	31.87	0.99 (s)
18	CH_2	111.12	4.84 (br s)
			4.83 (d, *J* = 1.3)
19	CH_3	16.89*	1.48 (d, *J* = 1.0)
20	CH_3	15.81	1.49 (m)

[a] Carbon numbering as shown in Figure S31 indicates the origin of each carbon from GGPP by same number. [b] Chemical shifts δ in ppm, multiplicity: s = singlet, d = doublet, t = triplet, m = multiplet, br = broad, coupling constants *J* are given in Hertz. Asterisks indicate carbons that show peak broadening.

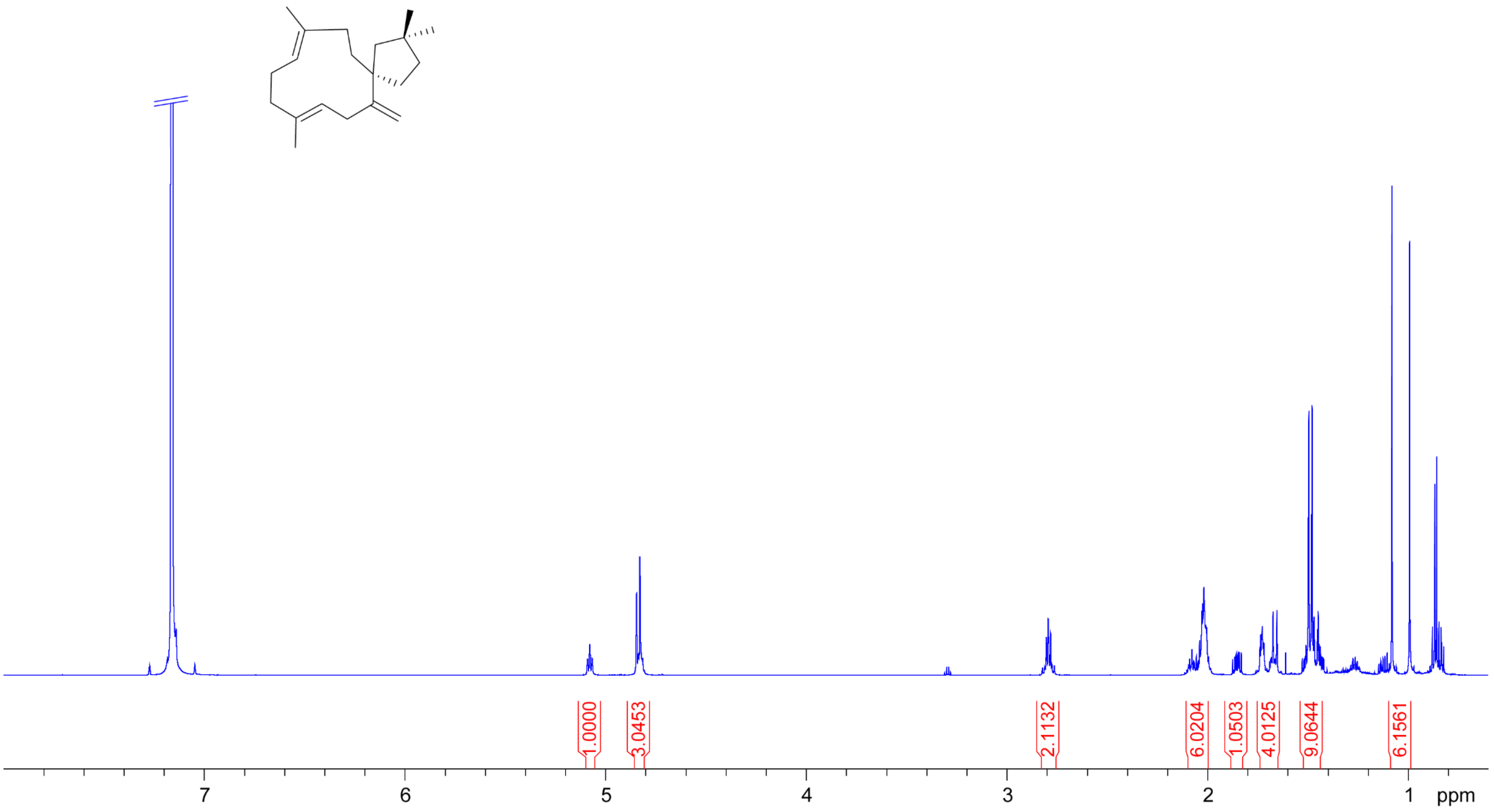

Figure S32. ^{1}H-NMR spectrum of **3** (700 MHz, C_6D_6) at 343 K.

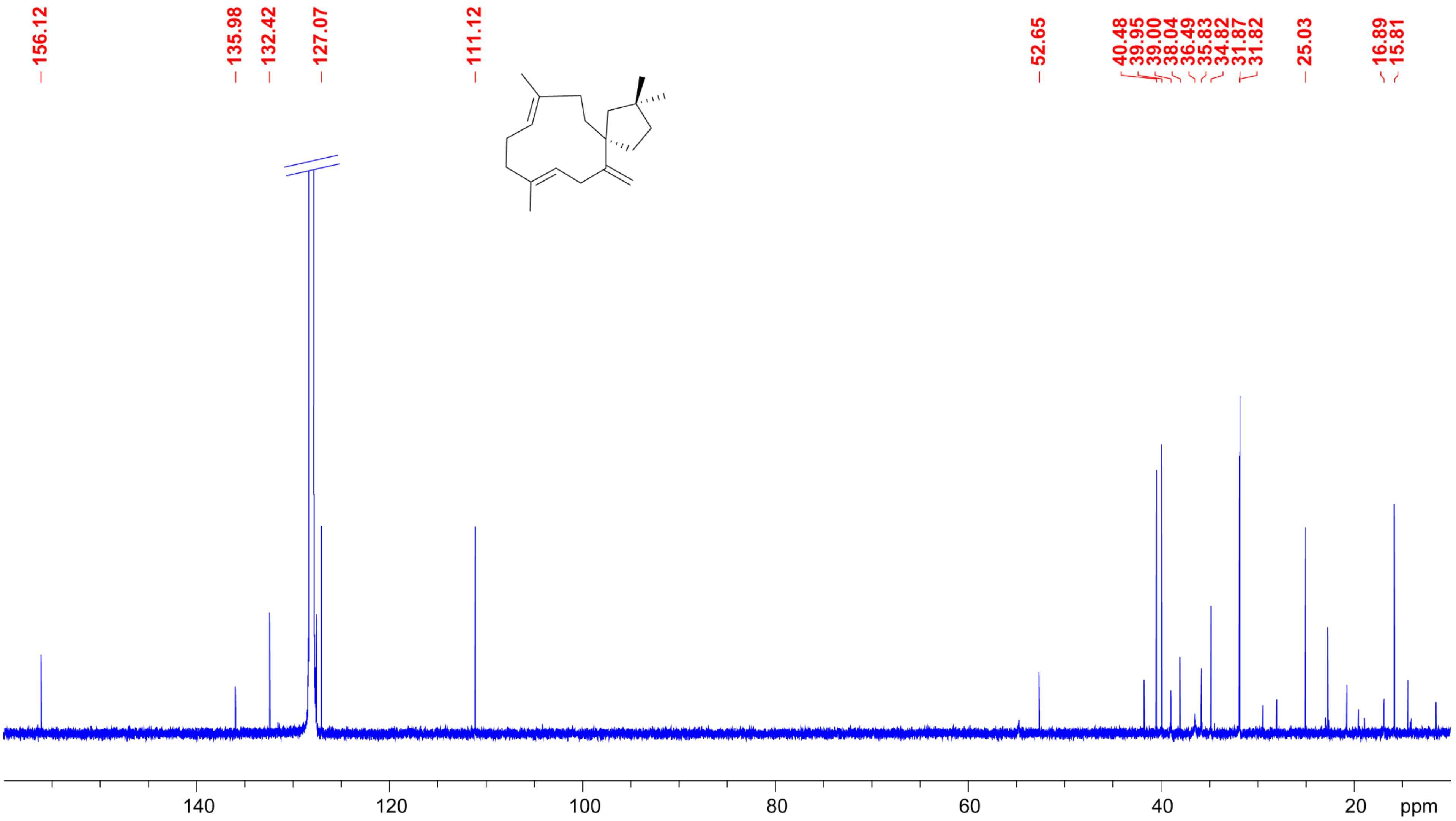

Figure S33. ^{13}C-NMR spectrum of **3** (176 MHz, C_6D_6) at 343 K.

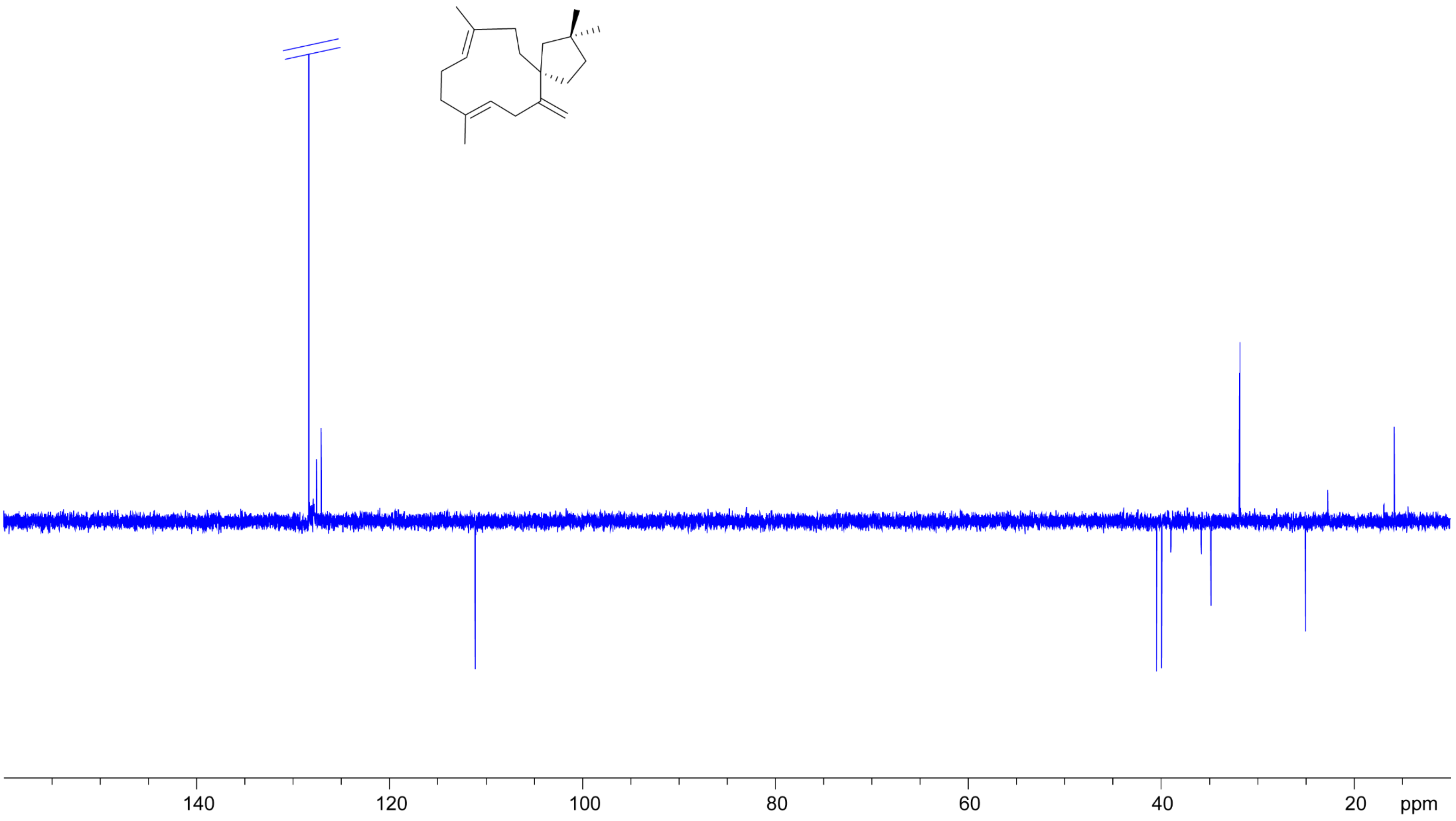

Figure S34. ^{13}C-DEPT135 spectrum of **3** (176 MHz, C_6D_6) at 343 K.

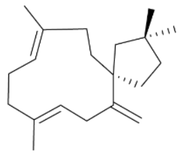

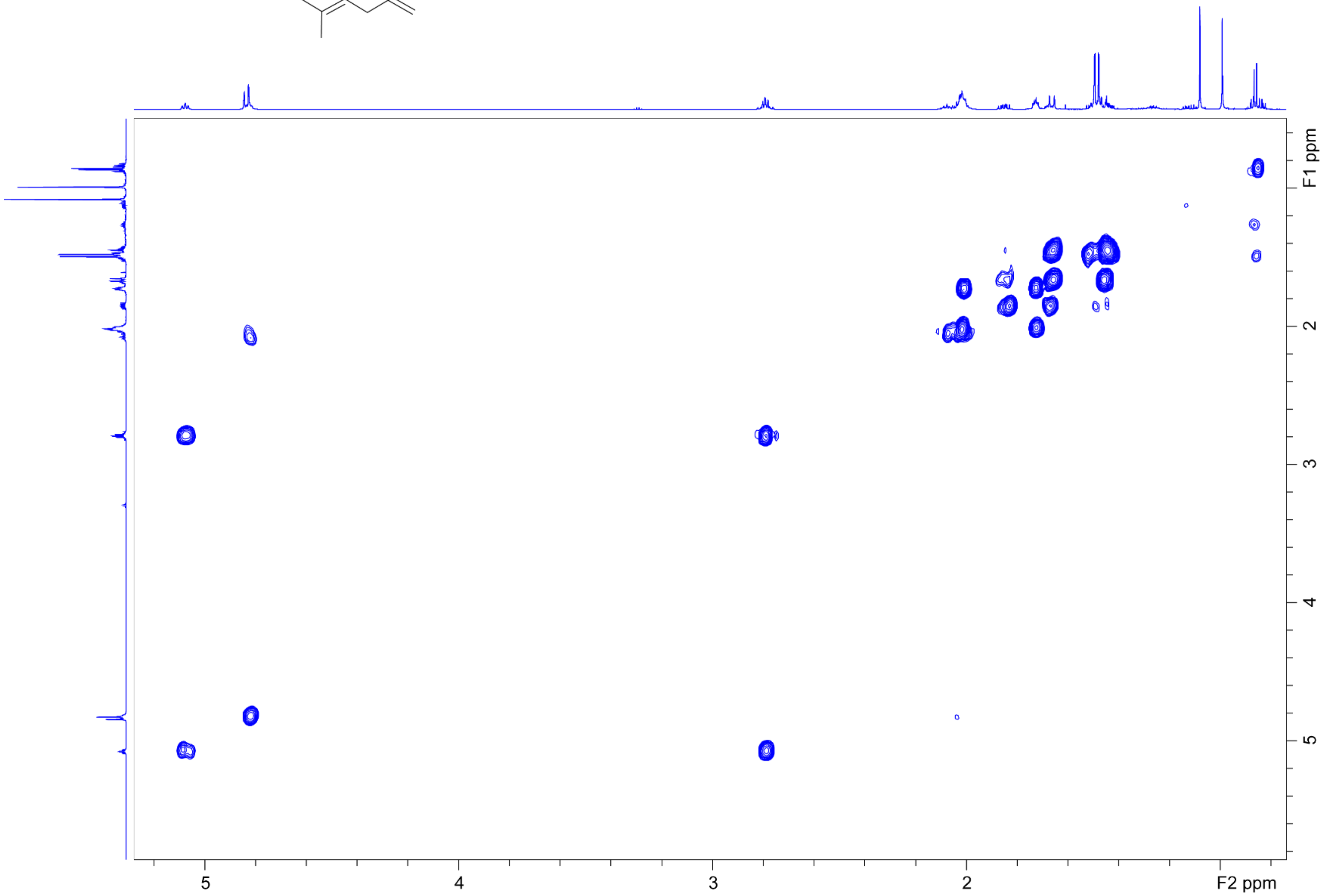

Figure S35. ^{1}H,^{1}H-COSY spectrum (C_6D_6) of **3** at 343 K.

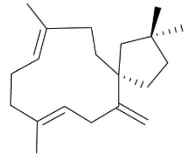

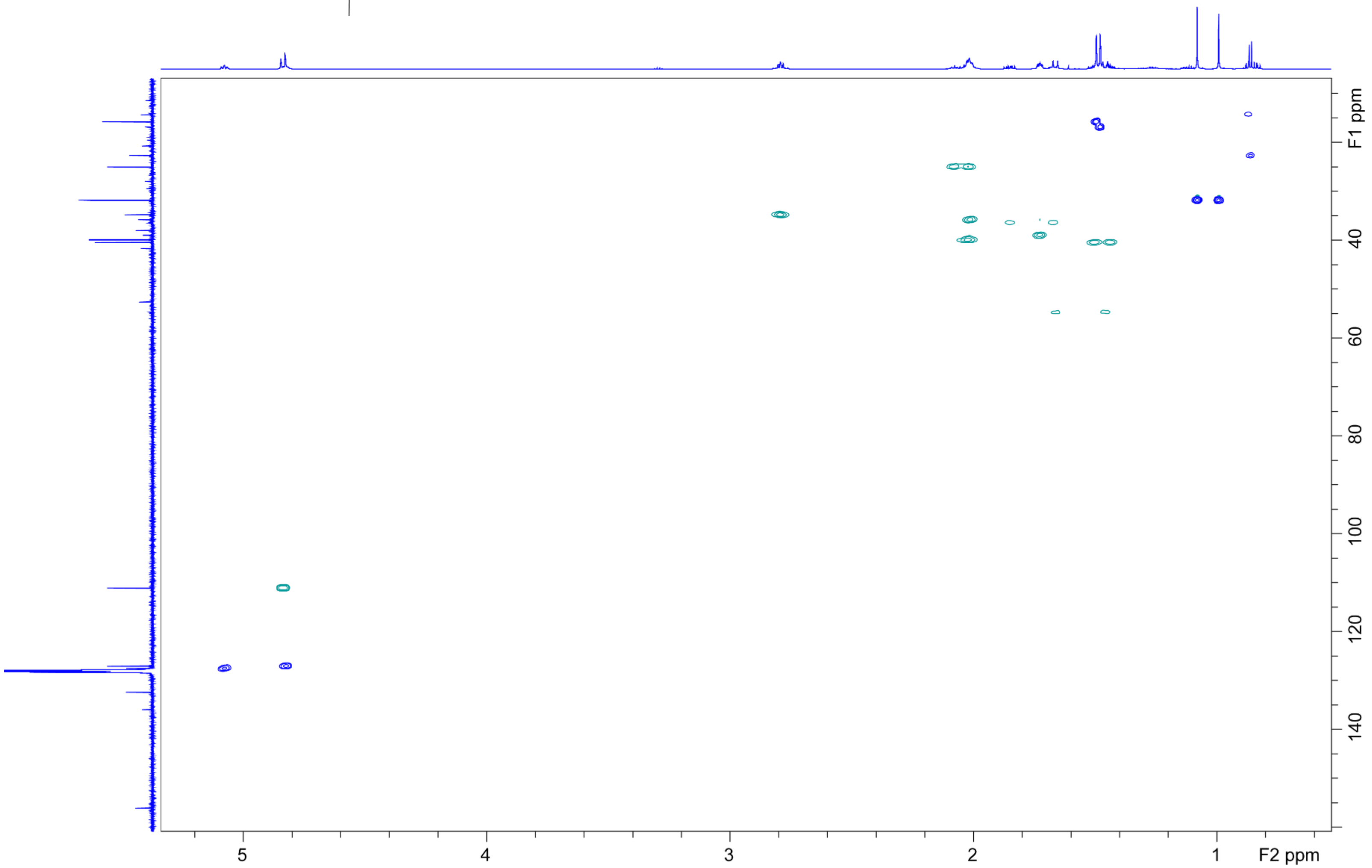

Figure S36. HSQC spectrum (C_6D_6) of **3** at 343 K.

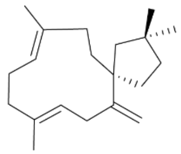

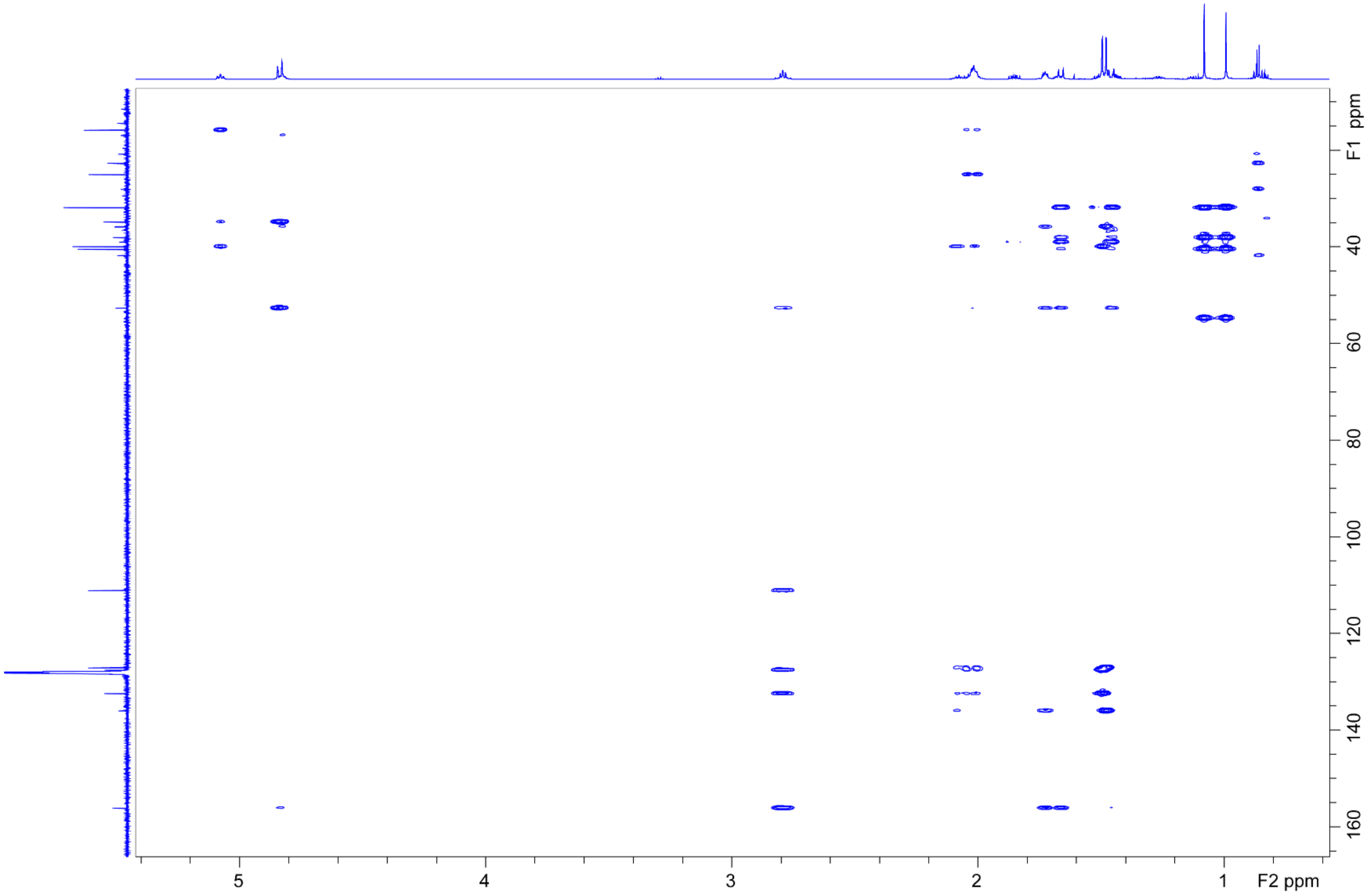

Figure S37. HMBC spectrum (C_6D_6) of **3** at 343 K.

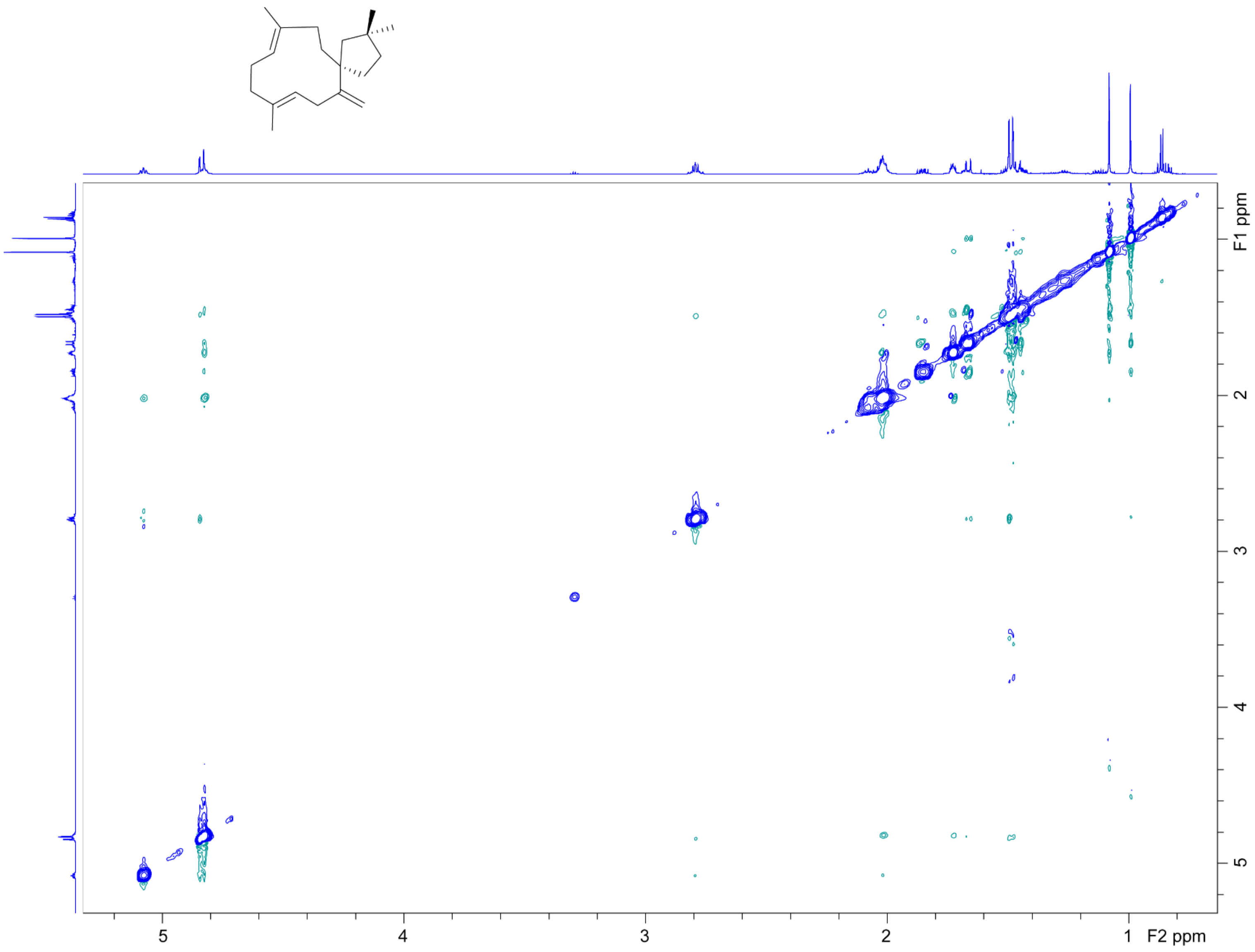

Figure S38. NOESY spectrum (C_6D_6) of **3** at 343 K.

Incubation experiments with labelled substrates

Isotopic labelling experiments were performed with the precursors of GGPP (ca. 1 mg in 1 mL 25 mM NH_4HCO_3), incubation buffer (5 mL) and preparations of purified enzymes (each 1 mL) as listed in Table S6. After incubation at 28 °C overnight, the products were extracted with C_6D_6 (600 µL + 300 µL) or *n*-hexane (500 µL), then the extracts were dried with $MgSO_4$ and analysed by NMR and/or GC/MS.

Table S6. Labelling experiments with CjCS.

no.	substrates	enzymes	results shown in
1	(*R*)-(1-^{13}C,1-^{2}H)IPP[6]	IDI,[7] GGPPS,[8] CjCS	Figures S39, S41, S43, S51 and S52
2	(*S*)-(1-^{13}C,1-^{2}H)IPP[6]	IDI,[7] GGPPS,[8] CjCS	Figures S39, S41, S43, S51 and S52
3	DMAPP,[9] (*E*)-(4-^{13}C,4-^{2}H)IPP[10]	GGPPS,[8] CjCS	Figures S40 and S42
4	DMAPP,[9] (*Z*)-(4-^{13}C,4-^{2}H)IPP[10]	GGPPS,[8] CjCS	Figures S40 and S42
5	FPP[9] + (1-^{13}C)IPP[8]	GGPPS,[8] CjCS	Figures S44, S46, S48 and S103
6	FPP[9] + (2-^{13}C)IPP[11]	GGPPS,[8] CjCS	Figures S44, S46, S48 and S103
7	FPP[9] + (3-^{13}C)IPP[8]	GGPPS,[8] CjCS	Figures S44, S46, S48 and S103
8	FPP[9] + (4-^{13}C)IPP[8]	GGPPS,[8] CjCS	Figures S44, S46, S48 and S103
9	(1-^{13}C)FPP[12] + IPP[9]	GGPPS,[8] CjCS	Figures S44, S46, S48 and S103
10	(2-^{13}C)FPP[12] + IPP[9]	GGPPS,[8] CjCS	Figures S44, S46, S48 and S103
11	(3-^{13}C)FPP[12] + IPP[9]	GGPPS,[8] CjCS	Figures S44, S46, S48 and S103
12	(4-^{13}C)FPP[12] + IPP[9]	GGPPS,[8] CjCS	Figures S44, S46, S48 and S103
13	(5-^{13}C)FPP[12] + IPP[9]	GGPPS,[8] CjCS	Figures S44, S46, S48 and S103
14	(6-^{13}C)FPP[12] + IPP[9]	GGPPS,[8] CjCS	Figures S44, S46, S48 and S103
15	(3-^{13}C)GPP[13] + IPP[9]	GGPPS,[8] CjCS	Figures S45, S47, S49 and S104
16	(8-^{13}C)FPP[12] + IPP[9]	GGPPS,[8] CjCS	Figures S45, S47, S49 and S104
17	(5-^{13}C)GPP + IPP[9]	GGPPS,[8] CjCS	Figures S45, S47, S49 and S104
18	(10-^{13}C)FPP[12] + IPP[9]	GGPPS,[8] CjCS	Figures S45, S47, S49 and S104
19	(11-^{13}C)FPP[12] + IPP[9]	GGPPS,[8] CjCS	Figures S45, S47, S49 and S104
20	(12-^{13}C)FPP[12] + IPP[9]	GGPPS,[8] CjCS	Figures S45, S47, S49 and S104
21	(9-^{13}C)GPP[14] + IPP[9]	GGPPS,[8] CjCS	Figures S45, S47, S49 and S104
22	(14-^{13}C)FPP[12] + IPP[9]	GGPPS,[8] CjCS	Figures S45, S47, S49 and S104
23	(15-^{13}C)FPP[12] + IPP[9]	GGPPS,[8] CjCS	Figures S45, S47, S49 and S104
24	FPP[9] + (5-^{13}C)IPP[15]	GGPPS,[8] CjCS	Figures S45, S47, S49 and S104
25	(2-^{2}H)GPP[14] + IPP[9]	GGPPS,[8] CjCS	Figure S50
26	GGPP + ^2H_2O	CjCS	Figures S51, S52, and S99 – S101
27	(1-^{13}C)GPP[16] + IPP + ^2H_2O	GGPPS,[8] CjCS	Figure S53
28	(*R*)-(1-^{13}C,1-^{2}H)IPP[6]	IDI,[7] GGPPS,[8] CjCS-I188L	Figures S92 and S94
29	(*S*)-(1-^{13}C,1-^{2}H)IPP[6]	IDI,[7] GGPPS,[8] CjCS-I188L	Figures S92 and S94
30	DMAPP,[9] (*E*)-(4-^{13}C,4-^{2}H)IPP[10]	GGPPS,[8] CjCS-I188L	Figures S93 and S95
31	DMAPP,[9] (*Z*)-(4-^{13}C,4-^{2}H)IPP[10]	GGPPS,[8] CjCS-I188L	Figures S93 and S95
32	(*R*)-(1-^{13}C,1-^{2}H)IPP[6]	IDI,[7] GGPPS,[8] CjCS-M86A	Figure S96
33	(*S*)-(1-^{13}C,1-^{2}H)IPP[6]	IDI,[7] GGPPS,[8] CjCS-M86A	Figure S96
34	DMAPP,[9] (*E*)-(4-^{13}C,4-^{2}H)IPP[10]	GGPPS,[8] CjCS-M86A	Figure S97
35	DMAPP,[9] (*Z*)-(4-^{13}C,4-^{2}H)IPP[10]	GGPPS,[8] CjCS-M86A	Figure S97
36	(2-^{2}H)GPP[14] + (2-^{13}C)IPP[11]	GGPPS,[8] CjCS-I188L	Figure S98
37	(2-^{13}C)GGPP[8] + ^2H_2O	CjCS-I188L	Figure S102

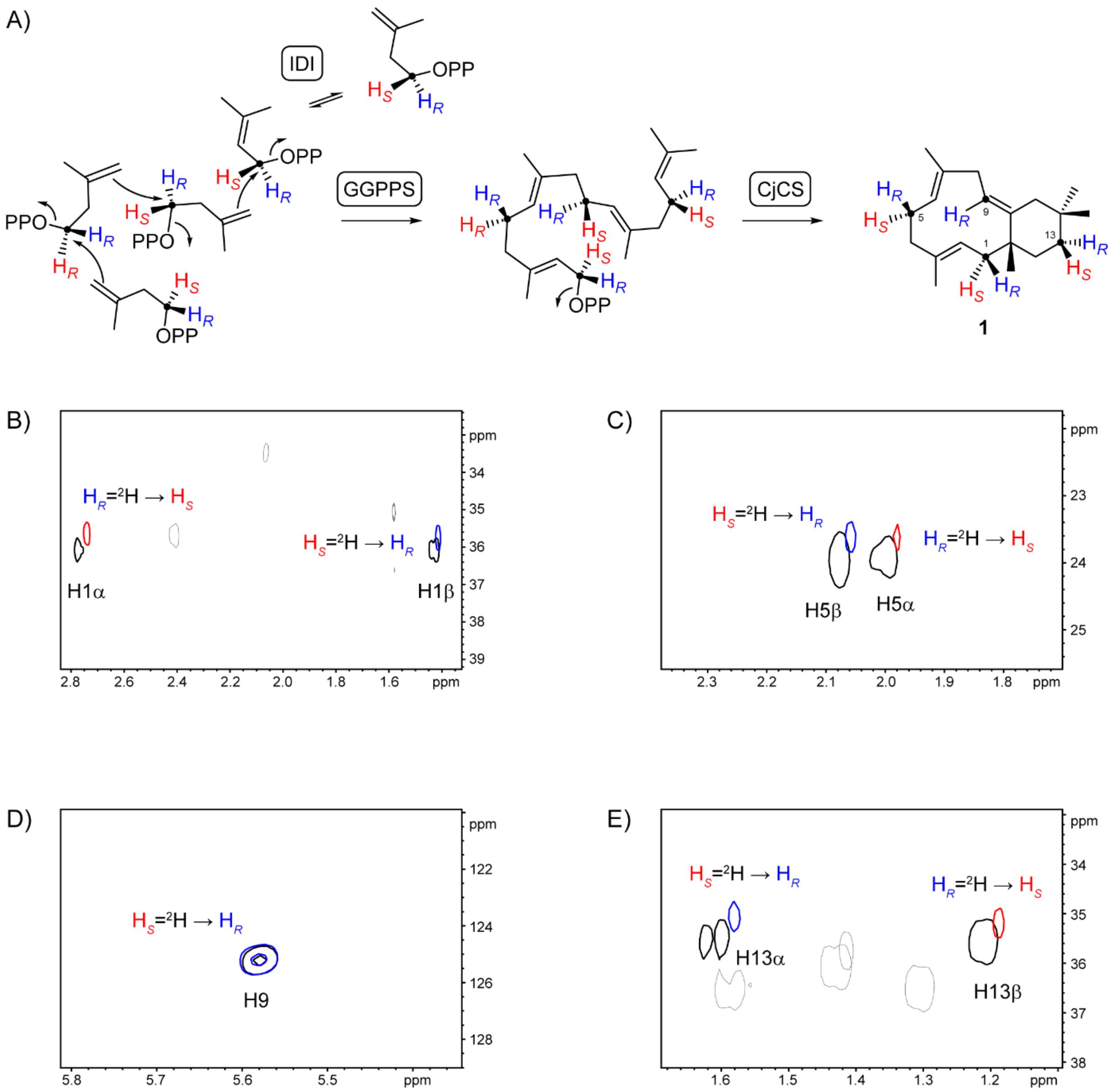

Figure S39. The absolute configuration of **1**. A) Conversion of (*R*)- and (*S*)-(1-^{13}C,1-^{2}H)IPP by IDI, GGPPS and CjCS into **1**. Three overlaid HSQC spectra at positions B) C1, C) C5, D) C9, and E) C13 for unlabelled **1** shown in black, for labelled **1** obtained with GGPPS and CjCS from (*S*)-(1-^{13}C,1-^{2}H)IPP shown in blue, and for labelled **1** obtained with GGPPS and CjCS from (*R*)-(1-^{13}C,1-^{2}H)IPP shown in red. Experiments B), C) and E) indicate the absolute configuration of **1**, while experiment D) confirms retainment of the 9-*pro*-*R* hydrogen of GGPP at C9. Crosspeaks irrelevant to the experiment are faded in grey.

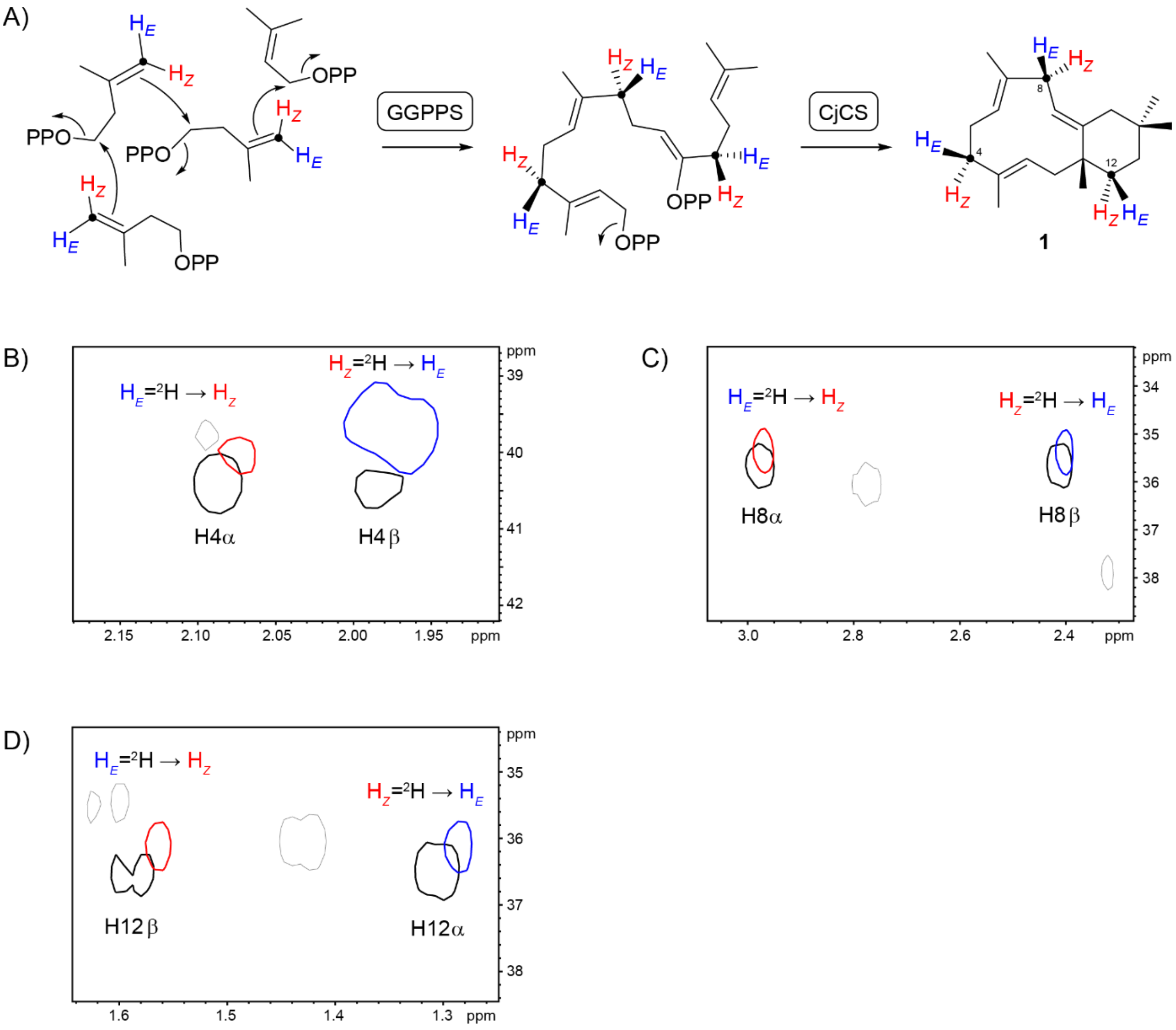

Figure S40. The absolute configuration of **1**. A) Conversion of DMAPP and (*E*)- and (*Z*)-(4-^{13}C,4-^{2}H)IPP by GGPPS and CjCS into **1**. Three overlaid HSQC spectra at positions B) C4, C) C8, and D) C12 for unlabelled **1** shown in black, for labelled **1** obtained with GGPPS and CjCS from DMAPP and (*Z*)-(4-^{13}C,4-^{2}H)IPP shown in blue, and for labelled **1** obtained with GGPPS and CjCS from DMAPP and (*E*)-(4-^{13}C,4-^{2}H)IPP shown in red. Experiments B), C) and D) indicate the absolute configuration of **1**. The blue crosspeak in B) appears large because of signal overlap with an unknown contaminant. Crosspeaks irrelevant to the experiment are faded in grey.

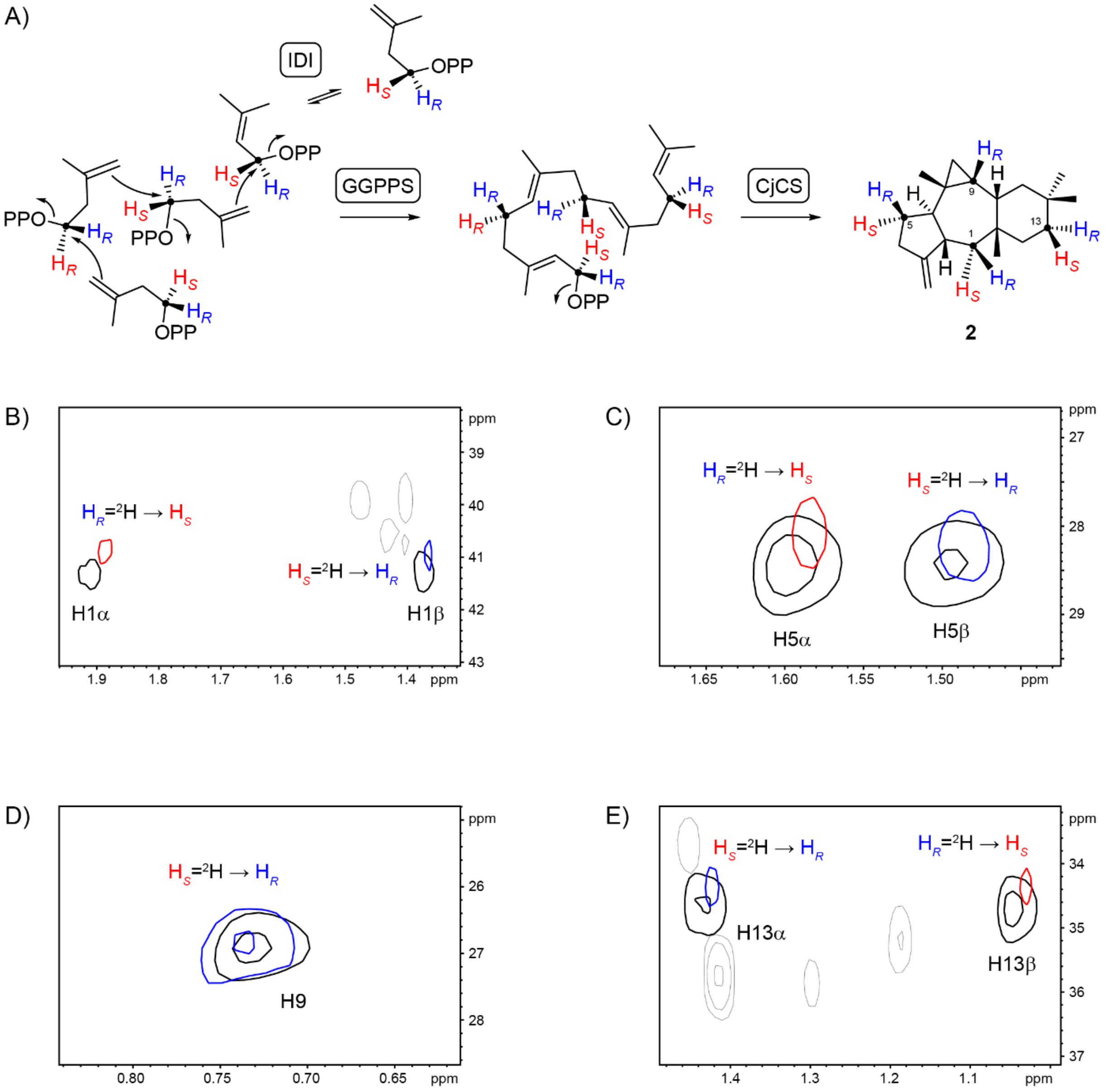

Figure S41. The absolute configuration of **2**. A) Conversion of (*R*)- and (*S*)-(1-^{13}C,1-^{2}H)IPP by IDI, GGPPS and CjCS into **2**. Three overlaid HSQC spectra at positions B) C1, C) C5, D) C9, and E) C13 for unlabelled **2** shown in black, for labelled **2** obtained with GGPPS and CjCS from (*S*)-(1-^{13}C,1-^{2}H)IPP shown in blue, and for labelled **2** obtained with GGPPS and CjCS from (*R*)-(1-^{13}C,1-^{2}H)IPP shown in red. Experiments B), C) and E) indicate the absolute configuration of **2**, while experiment D) confirms retainment of the 9-*pro-R* hydrogen of GGPP at C9. Crosspeaks irrelevant to the experiment are faded in grey.

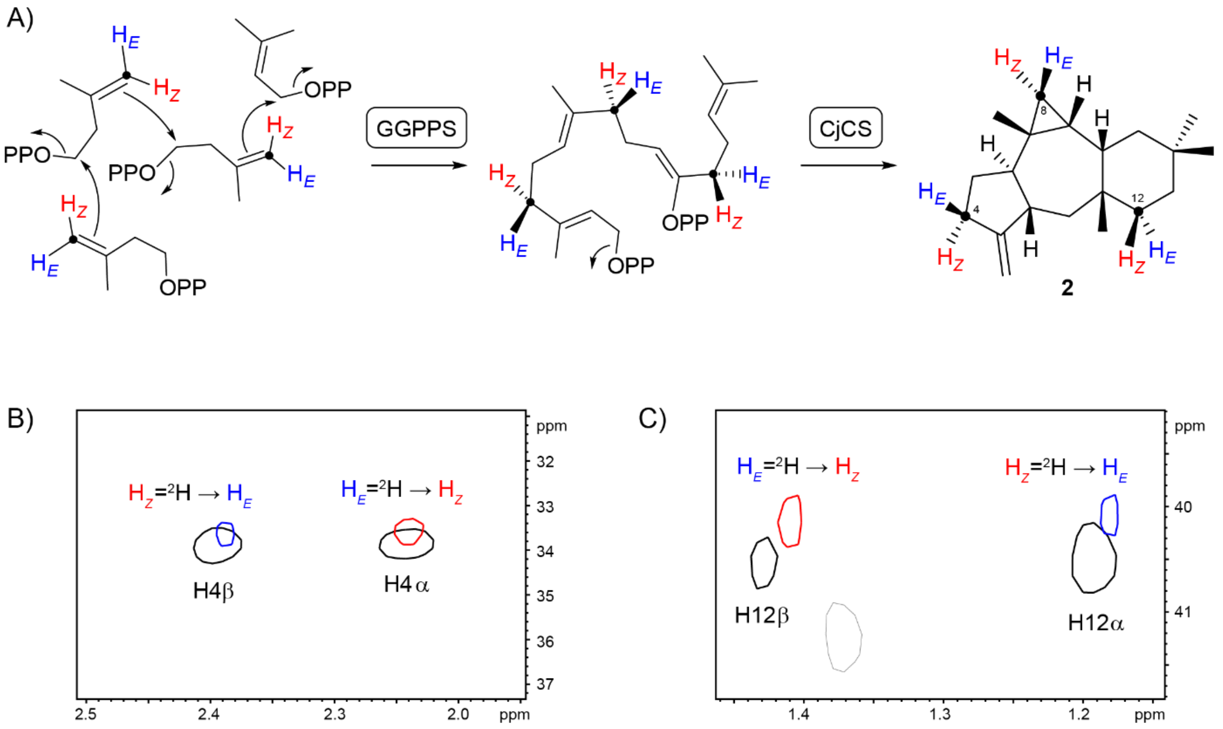

Figure S42. The absolute configuration of **2**. A) Conversion of DMAPP and (*E*)- and (*Z*)-(4-^{13}C,4-^{2}H)IPP by GGPPS and CjCS into **2**. Three overlaid HSQC spectra at positions B) C4, and C) C12 for unlabelled **2** shown in black, for labelled **2** obtained with GGPPS and CjCS from DMAPP and (*Z*)-(4-^{13}C,4-^{2}H)IPP shown in blue, and for labelled **2** obtained with GGPPS and CjCS from DMAPP and (*E*)-(4-^{13}C,4-^{2}H)IPP shown in red. Experiments B) and C) indicate the absolute configuration of **2**. The diastereotopic hydrogens at C8 are isochronic and thus this position cannot be used to determine the absolute configuration of **2**. Crosspeaks irrelevant to the experiment are faded in grey.

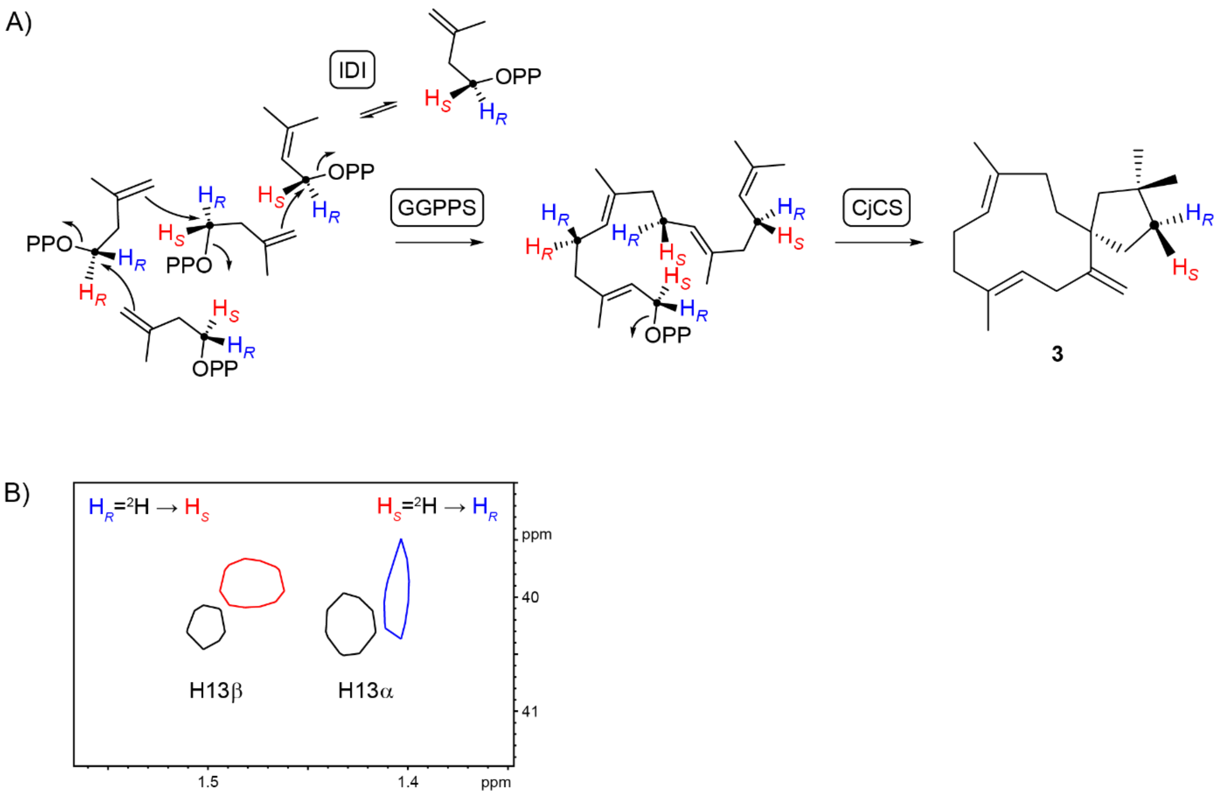

Figure S43. The absolute configuration of **3**. A) Conversion of (*R*)- and (*S*)-(1-^{13}C,1-^{2}H)IPP by IDI, GGPPS and CjCS into **3**. B) Three overlaid HSQC spectra at position C13 for unlabelled **3** shown in black, for labelled **3** obtained with GGPPS and CjCS from (*S*)-(1-^{13}C,1-^{2}H)IPP shown in blue, and for labelled **3** obtained with GGPPS and CjCS from (*R*)-(1-^{13}C,1-^{2}H)IPP shown in red. Experiment B) indicates the absolute configuration of **3**, while other potentially relevant carbon positions in these experiments (C1, C5 and C9) were inconclusive, because the protons in these positions are isochronic or the signals were not observed because of peak broadening.

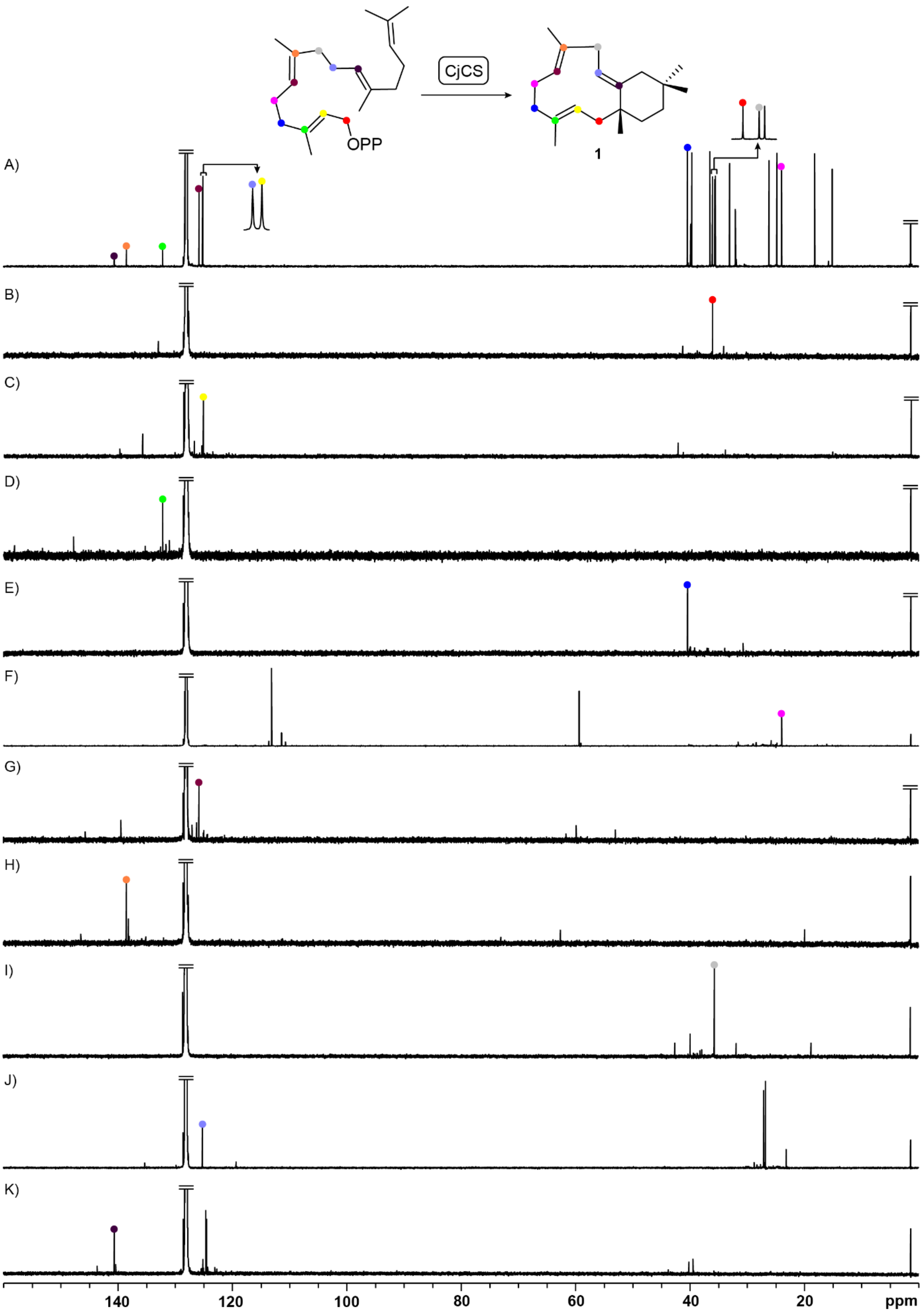

Figure S44. The biosynthetic origin of the carbon framework of **1**. ^{13}C-NMR spectra of A) unlabelled **1**, and B) – K) of labelled **1** obtained with CjCS from (1-^{13}C)GGPP – (10-^{13}C)GGPP. The coloured dots correlate the labelled carbons in GGPP and **1** with the corresponding ^{13}C-signals in the spectra.

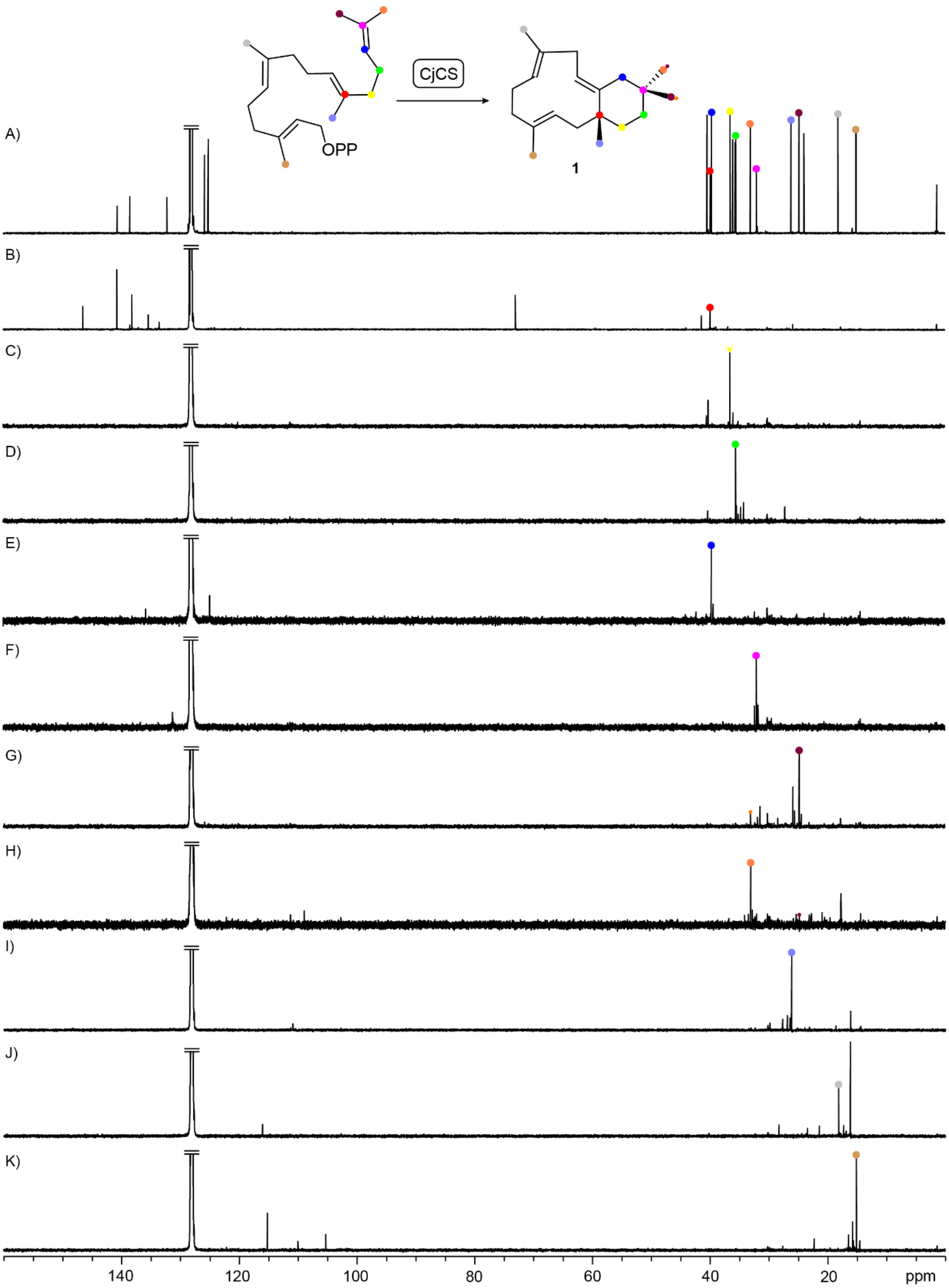

Figure S45. The biosynthetic origin of the carbon framework of **1**. ^{13}C-NMR spectra of A) unlabelled **1**, and B) – K) of labelled **1** obtained with CjCS from (11-^{13}C)GGPP – (20-^{13}C)GGPP. The coloured dots correlate the labelled carbons in GGPP and **1** with the corresponding ^{13}C-signals in the spectra.

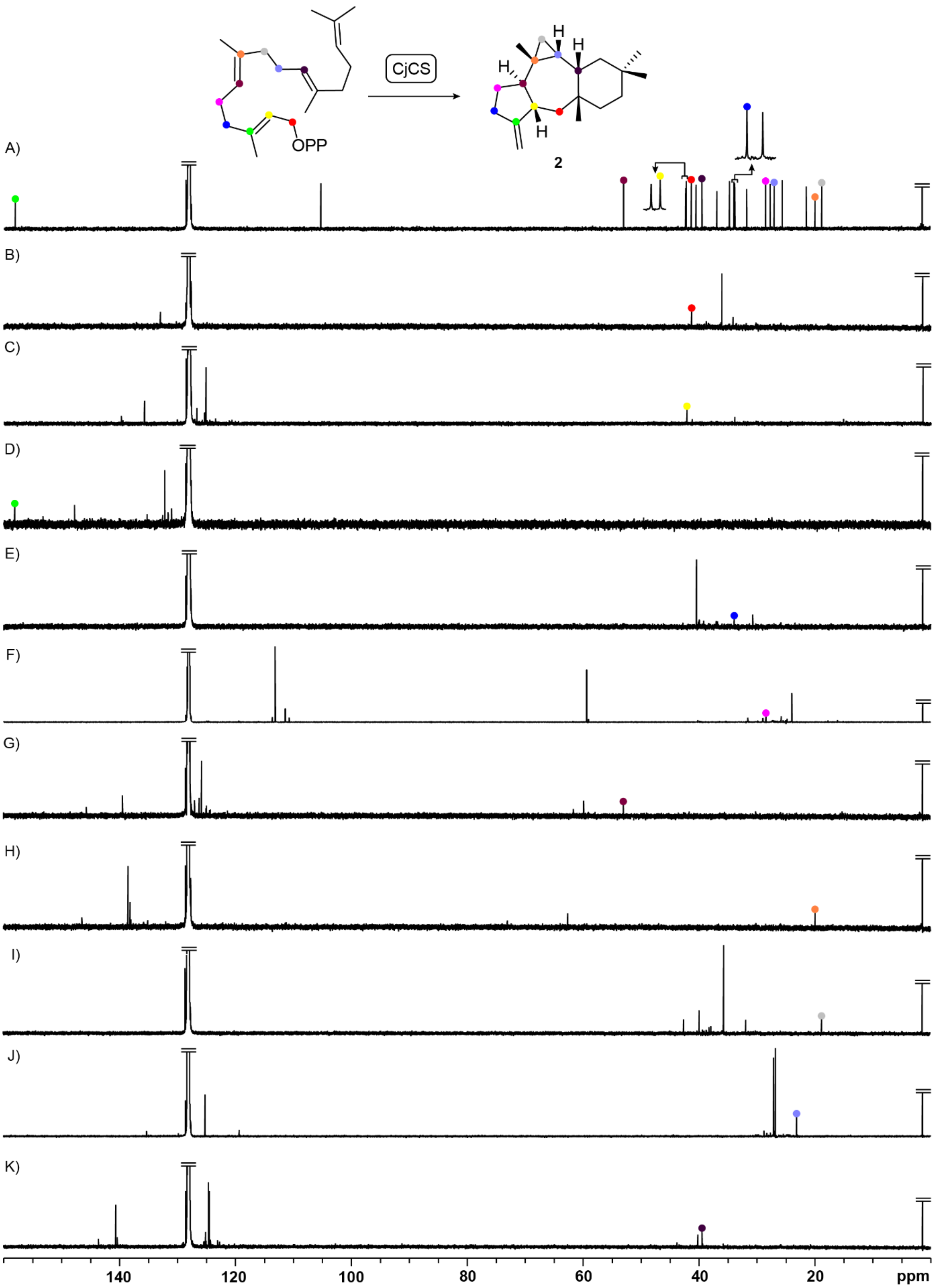

Figure S46. The biosynthetic origin of the carbon framework of **2**. ^{13}C-NMR spectra of A) unlabelled **2**, and B) – K) of labelled **2** obtained with CjCS from (1-^{13}C)GGPP – (10-^{13}C)GGPP. The coloured dots correlate the labelled carbons in GGPP and **2** with the corresponding ^{13}C-signals in the spectra.

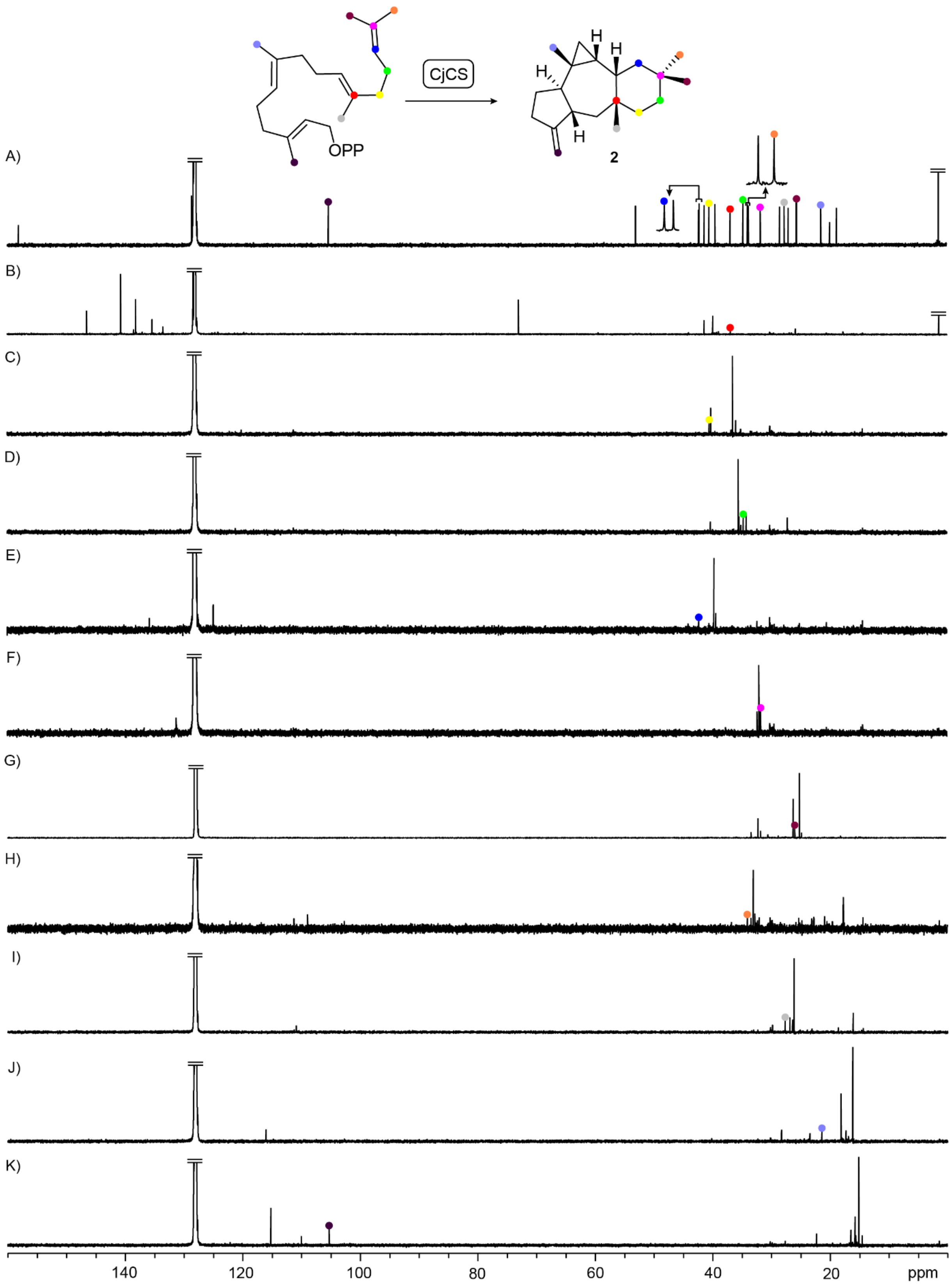

Figure S47. The biosynthetic origin of the carbon framework of **2**. ^{13}C-NMR spectra of A) unlabelled **2**, and B) – K) of labelled **2** obtained with CjCS from (11-^{13}C)GGPP – (20-^{13}C)GGPP. The coloured dots correlate the labelled carbons in GGPP and **2** with the corresponding ^{13}C-signals in the spectra.

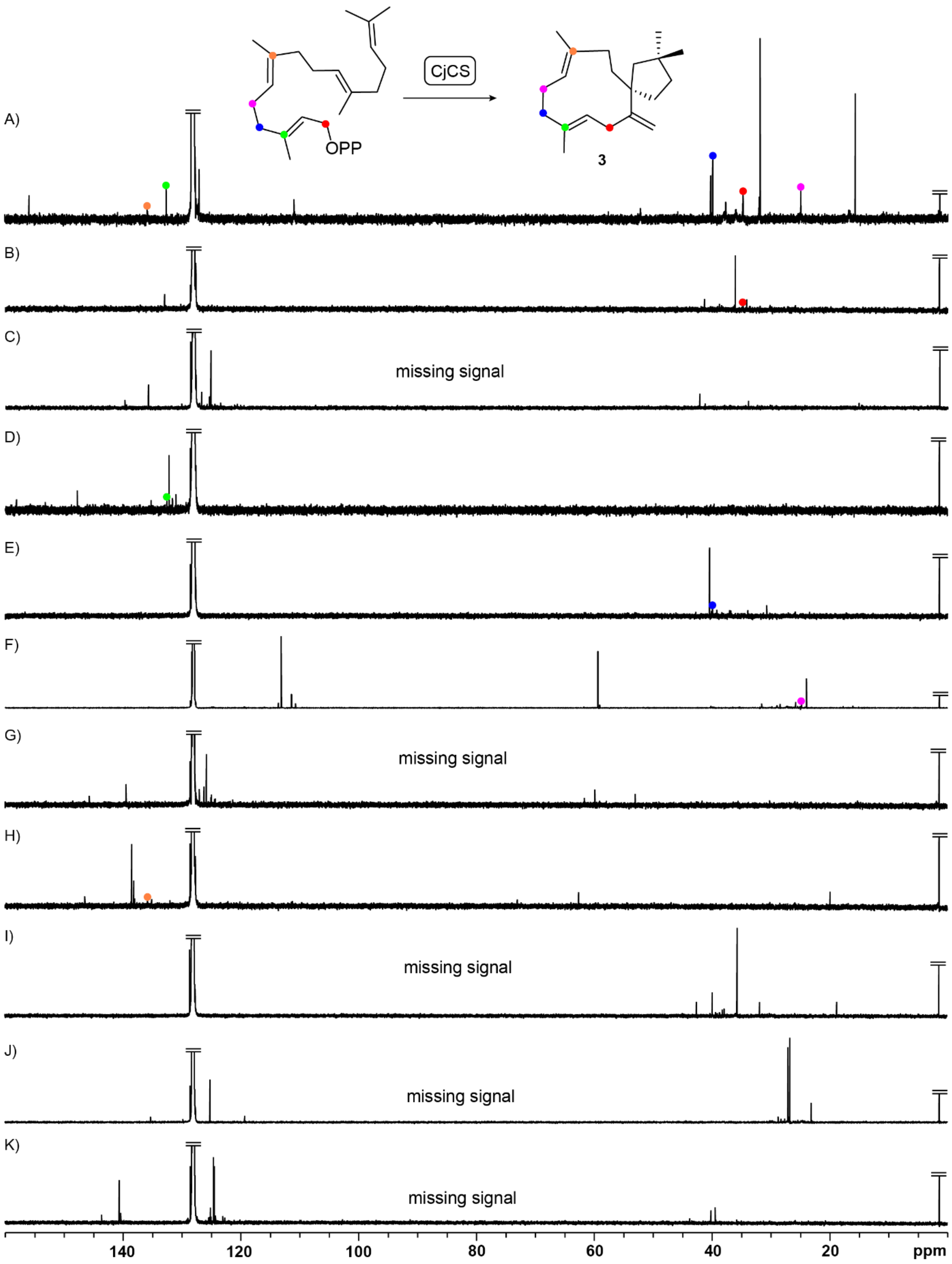

Figure S48. The biosynthetic origin of the carbon framework of **3**. ^{13}C-NMR spectra of A) unlabelled **3**, and B) – K) of labelled **3** obtained with CjCS from (1-^{13}C)GGPP – (10-^{13}C)GGPP. The coloured dots correlate the labelled carbons in GGPP and **3** with the corresponding ^{13}C-signals in the spectra.

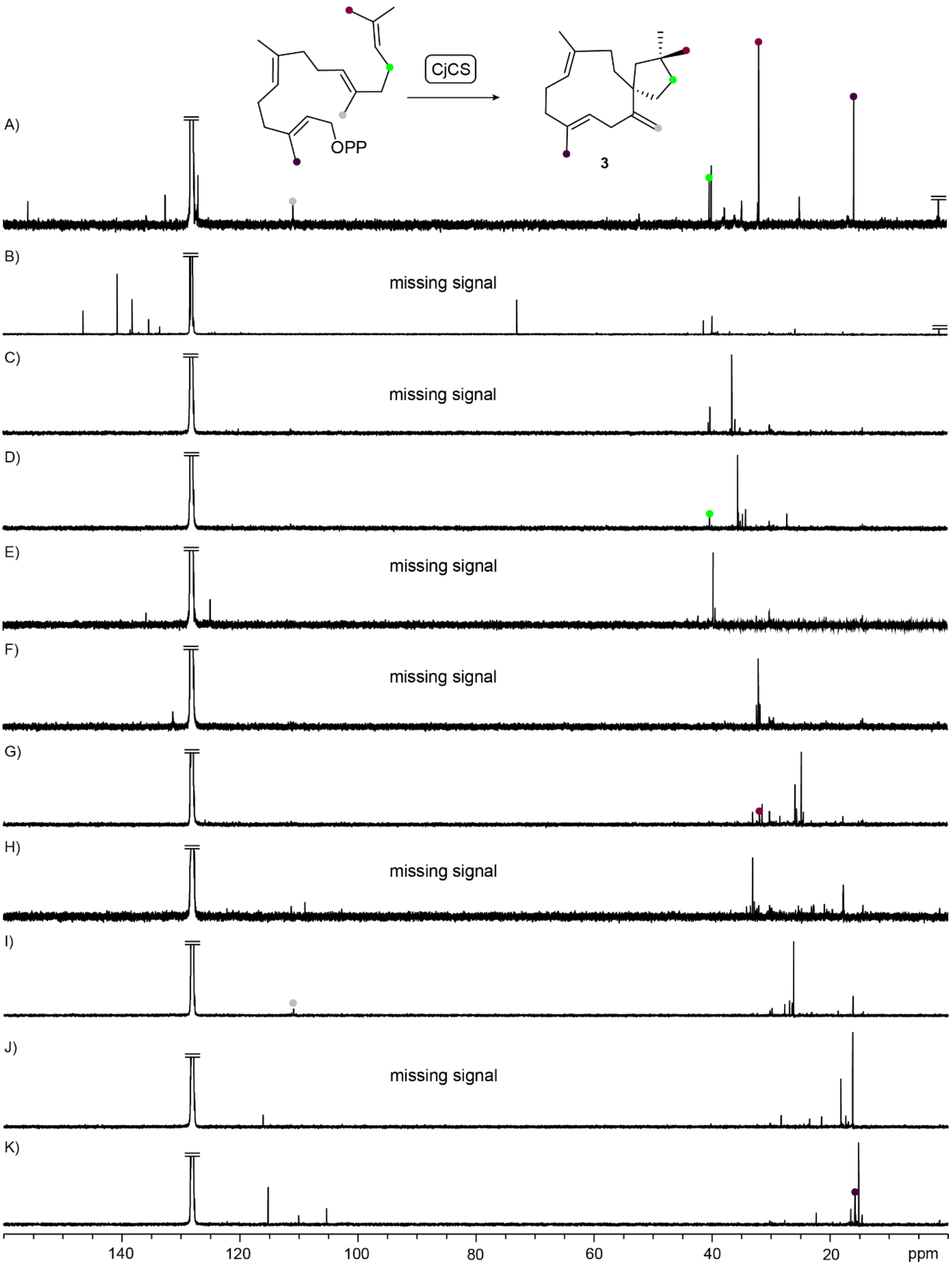

Figure S49. The biosynthetic origin of the carbon framework of **3**. ^{13}C-NMR spectra of A) unlabelled **3**, and B) – K) of labelled **3** obtained with CjCS from (11-^{13}C)GGPP – (20-^{13}C)GGPP. The coloured dots correlate the labelled carbons in GGPP and **3** with the corresponding ^{13}C-signals in the spectra.

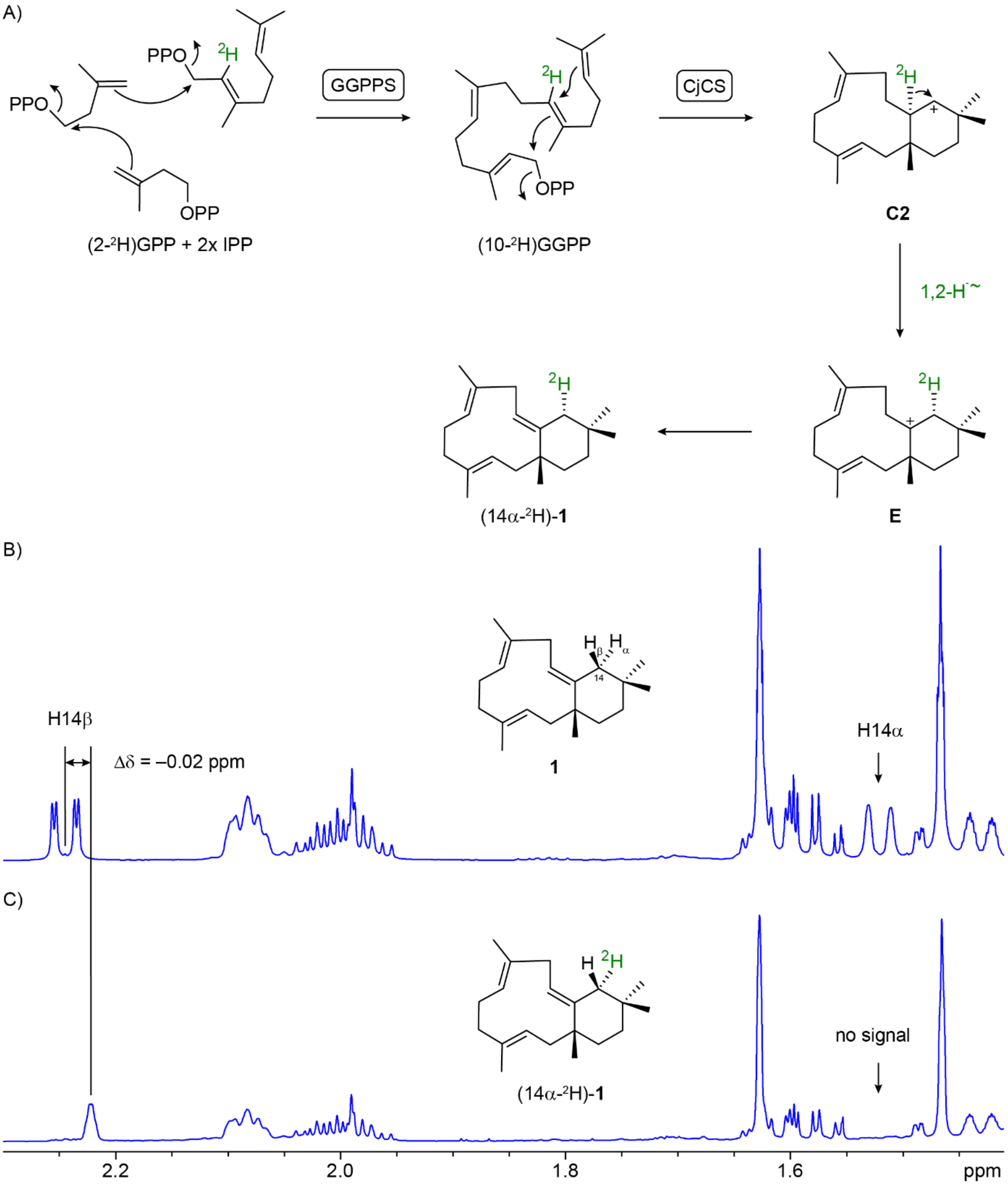

Figure S50. The 1,2-hydride shift from **C2** to **D**/**E** in the biosynthesis of **1**. A) Enzymatic formation of (10-^{2}H)GGPP from (2-^{2}H)GPP and IPP using GGPPS and conversion into (14α-^{2}H)-**1** with CjCS, B) ^{1}H-NMR spectrum of unlabelled **1**, and C) ^{1}H-NMR spectrum of (14α-^{2}H)-**1**. The missing doublet at δ = 1.51 ppm, the simplified spin multiplicity of the signal at 2.24 ppm lacking the $^{2}J_{H,H}$ coupling, and the slight upfield shift of this signal are in agreement with deuterium incorporation into H14α.

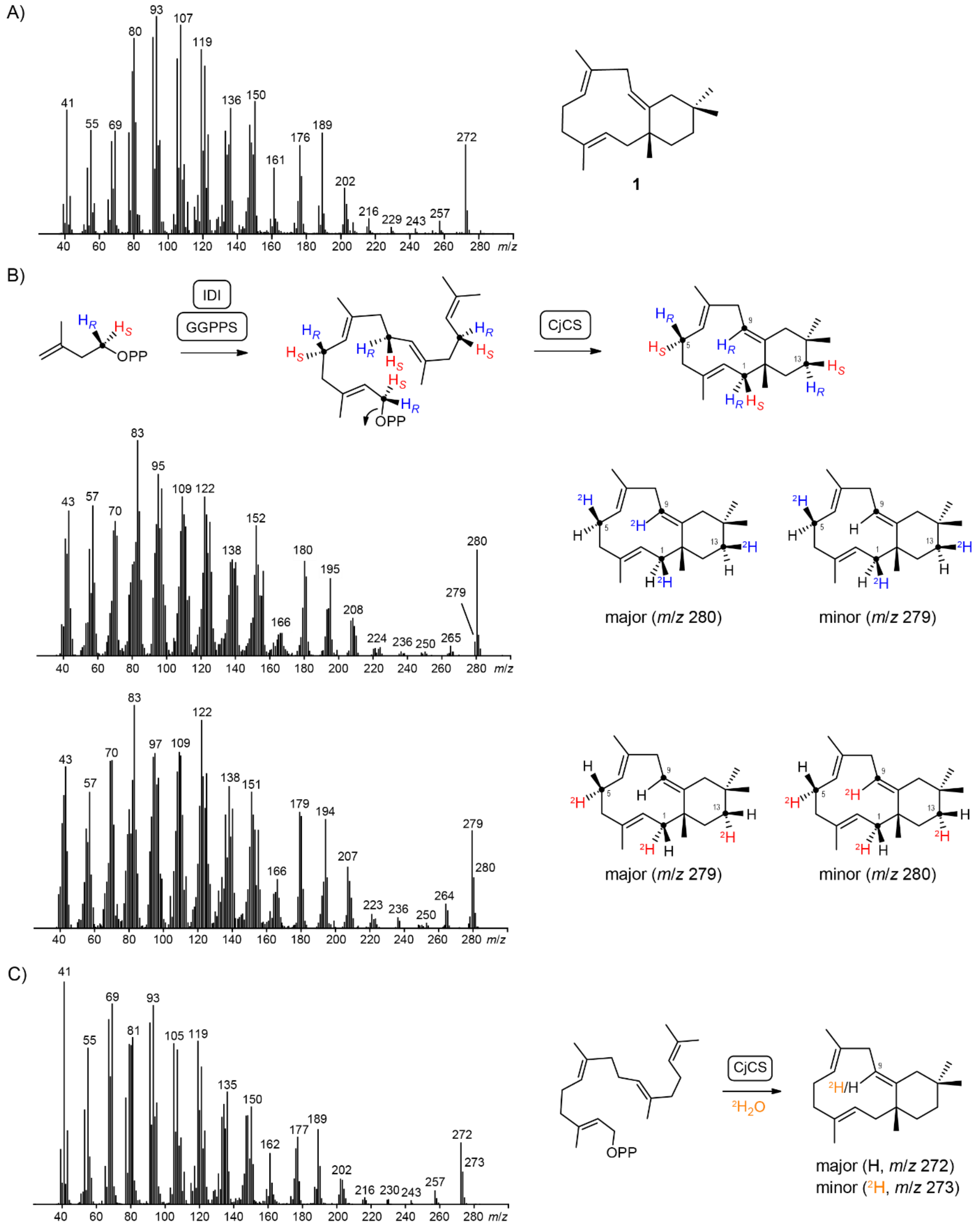

Figure S51. The deprotonation to **1** and its reprotonation by CjCS. A) EI mass spectrum of unlabelled **1**, B) conversion of (*R*)- and (*S*)-(1-^{13}C,1-^{2}H)IPP with IDI, GGPPS and CjCS into labelled **1**. From (*R*)-(1-^{13}C,1-^{2}H)IPP the major product is formed with retainment of deuterium at C1 (*m*/*z* 280), but also some loss is observed (12%, *m*/*z* 279). The opposite result is obtained with (*S*)-(1-^{13}C,1-^{2}H)IPP, showing loss of deuterium at C9 for the major product (*m*/*z* 279), but also minor retainment (34%, *m*/*z* 280). The differences account for a kinetic isotope effect. C) The incubation of GGPP with CjCS in $^{2}H_2O$ results in a deuterium incorporation into **1**, explainable by a reprotonation of **1** at C9 and deprotonation to **1** with abstraction of the other proton from C9.

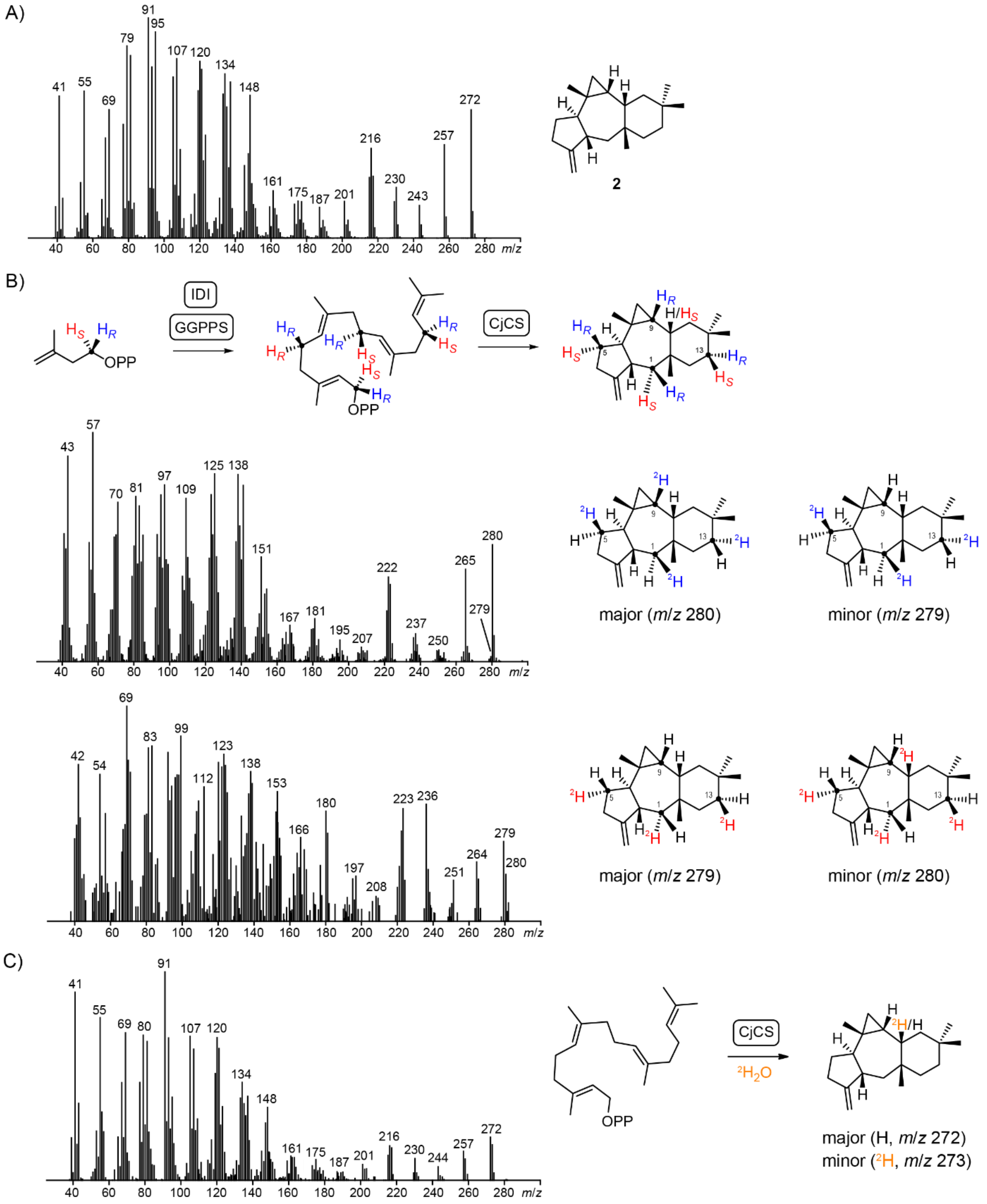

Figure S52. The formation of cation **F** by 1,2-hydride shift from **E** or reprotonation of **1**. A) EI mass spectrum of **2**, B) conversion of (*R*)- and (*S*)-(1-^{13}C,1-^{2}H)IPP with IDI, GGPPS and CjCS into labelled **2**. From (*R*)-(1-^{13}C,1-^{2}H)IPP the major product is formed with retainment of deuterium at C1 (*m*/*z* 280), but also some loss is observed (5%, *m*/*z* 279). The opposite result is obtained with (*S*)-(1-^{13}C,1-^{2}H)IPP, showing loss of deuterium at C9 for the major product (*m*/*z* 279), but also minor retainment (37%, *m*/*z* 280). The differences account for a kinetic isotope effect. C) The incubation of GGPP with CjCS in $^{2}H_2O$ results in a deuterium incorporation into **2**, explainable by a reprotonation of **1** at C10 (or at C9 followed by 1,2-hydride shift) to form intermediate **F** towards **2**.

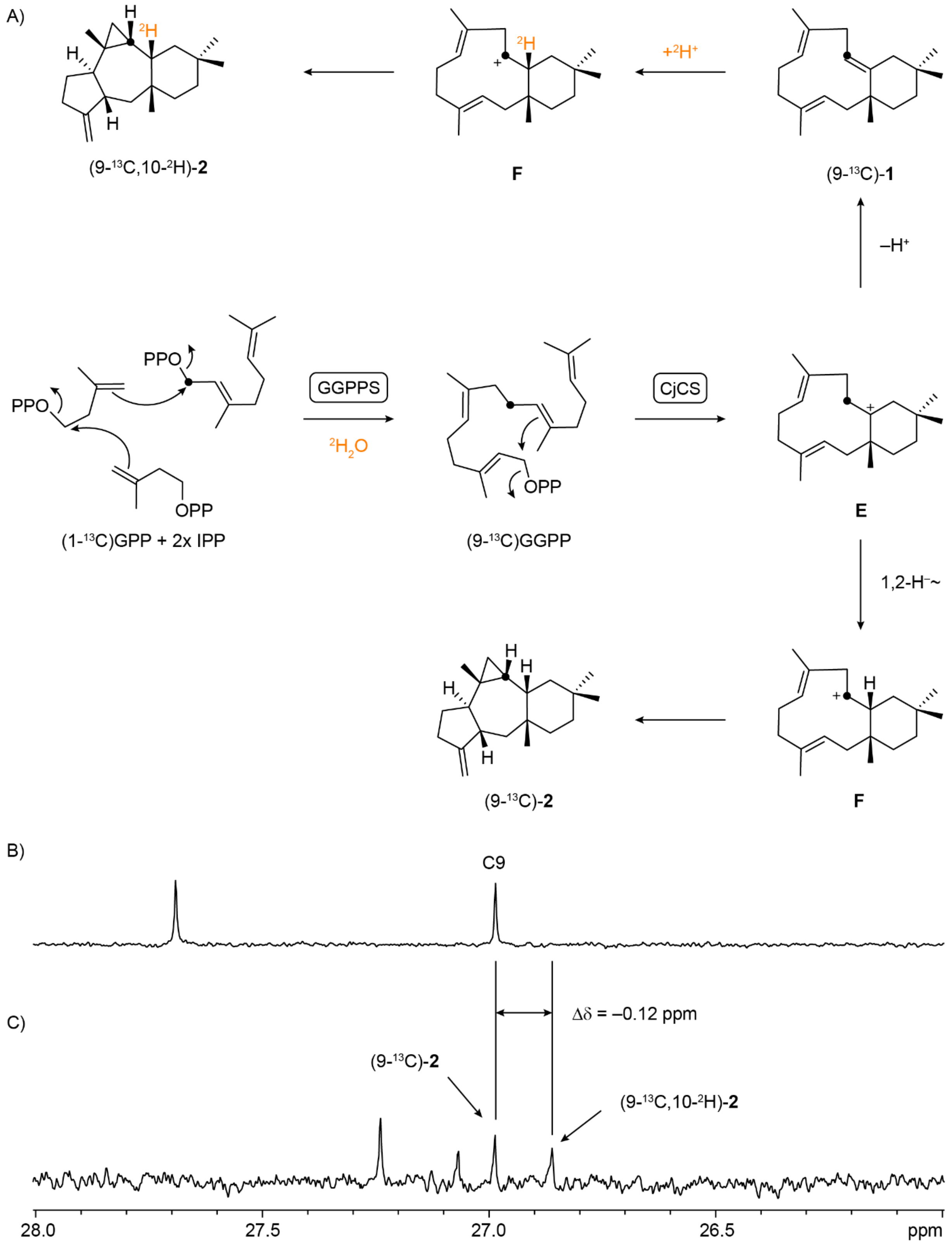

Figure S53. The 1,2-hydride shift from **E** to **F** in the biosynthesis of **2**. A) Enzymatic formation of (9-^{13}C)GGPP from (1-^{13}C)GPP and IPP using GGPPS and conversion into a mixture of (9-^{13}C)-**2** and (9-^{13}C,10-^{2}H)-**2** with CjCS in D_2O, B) ^{13}C-NMR spectrum of unlabelled **2**, and C) ^{13}C-NMR spectrum of an extract from the incubation of (1-^{13}C)GPP and IPP with GGPPS and CjCS in D_2O, showing the presence of a mixture of (9-^{13}C)-**2** (unshifted singlet) and (9-^{13}C,10-^{2}H)-**2** (slightly upfield shifted singlet as a result of deuterium in a neighbouring position).

Computational methods

All computed structures were geometry optimized without restrictions and were characterized as minima or as transition state structures by frequency analyses using the B97D3/6-31g(d,p) method with the density fitting approximation for s- and p-functions, including Grimme's empirical D3-dispersion correction[17] in Gaussian16.[18] Frequency computations also provided Gibbs corrections, which include Grimme's quasi-RRHO approach with a frequency cut-off value of 100.0 wave numbers using GoodVibes.[19,20] The python program "pyQRC, Quick Reaction Coordinate" (https://github.com/patonlab/pyQRC) of the Paton group was used, together with IRC computations, to find reactants of localised transition state structures. For single point energies, the mPW1PW91 functional was applied with the 6-311+G(d,p) basis set without density fitting and the ultra-fine integration grid, as this method was shown to be very reliable for examining carbocation cyclization and rearrangement reactions.[21-25] Conformational analyses were performed with xTB-GFN2 in the CREST 2.12 program (github.com/crest-lab), developed by the Grimme group.[26-30]

Table S7. Results of DFT calculations for the cyclisation cascade from GGPP to **1 – 7** (Scheme 1 of main text).

Structure	Gibbs energy (298.15K) in Hartree	energy relative to A in kcal/mol	reaction barrier in kcal/mol	Gibbs free energy in kcal/mol
**A**	–781.678830	0.00		
**A-B1-TS**	–781.668670	6.38	6.38	
**B1**	–781.715347	–22.91		–22.91
**B2**	–781.709552	–19.28		
**B2-C1-TS**	–781.691576	–8.00	11.28	
**C1**	–781.701384	–14.15		5.13
**C2**	–781.695110	–10.22		
**C2-D-TS**	–781.704203	–15.92	–5.71	
**D**	–781.705021	–16.44		–6.22
**D**	–781.705021	–16.44		
**D-E-TS**	–781.706629	–17.44	–1.01	
**E**	–781.726404	–29.85		–13.42
**E**	–781.726397	–29.85		
**E-F-TS**	–781.710192	–19.68	10.17	
**F**	–781.717481	–24.25		5.59
**F**	–781.717484	–24.26		
**F-G-TS**	–781.719672	–25.63	–1.37	
**G**	–781.718785	–25.07		–0.82
**G**	–781.718782	–25.07		
**G-H-TS**	–781.722066	–27.13	–2.06	
**H**	–781.744560	–41.25		–16.18
**C3**	–781.708043	–18.33		
**C3-I-TS**	–781.705941	–17.01	1.32	
**I**	–781.720223	–25.97		–7.64
**E**	–781.726401	–29.85		
**E-J-TS**	–781.712808	–21.32	8.53	
**J**	–781.717261	–24.12		5.74

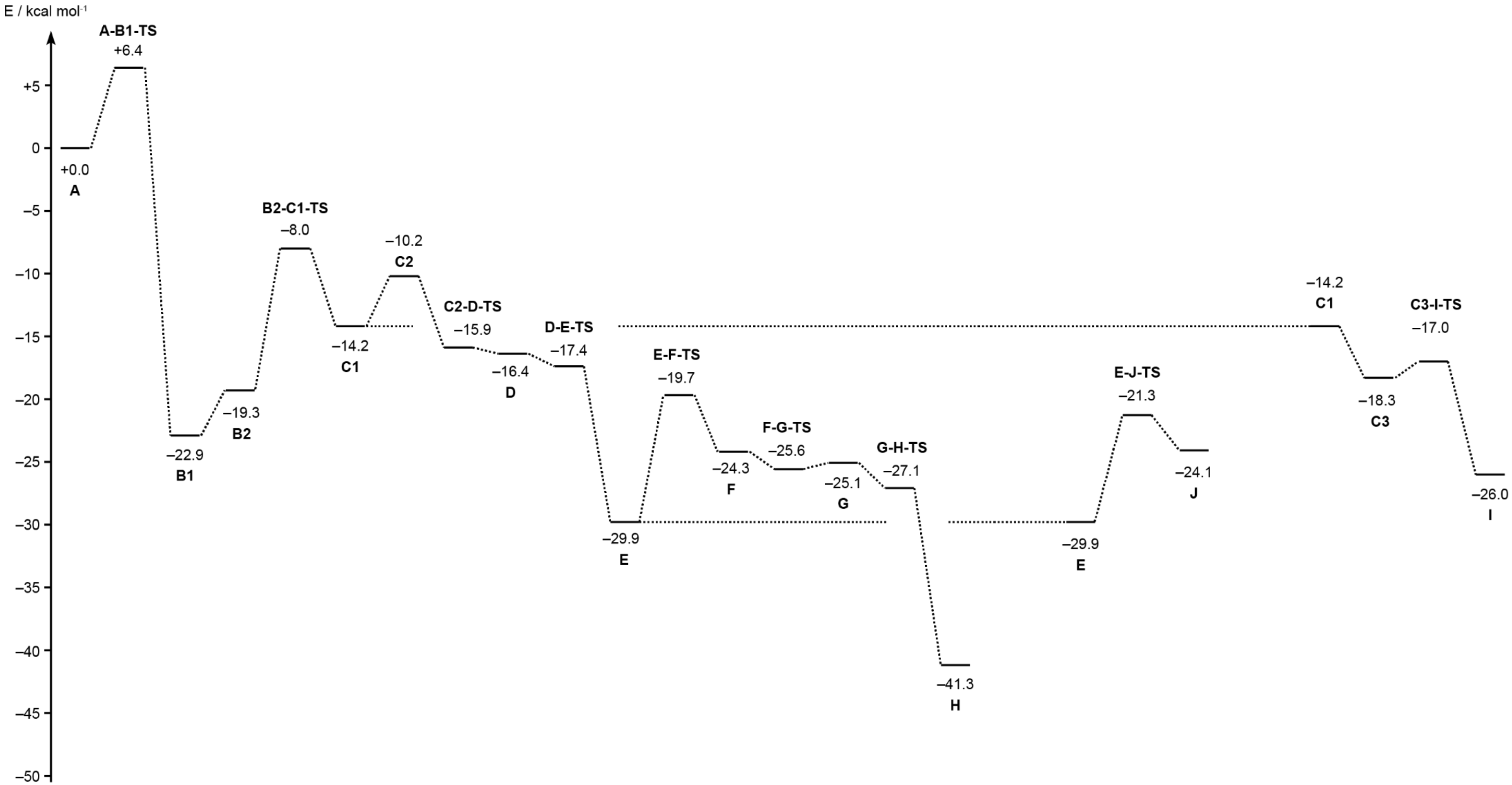

Figure S54. Computed energy profile for the transformations from **A** to **H**, **C3** to **I**, and **E** to **J** (Scheme 1 of main text, mPW1PW91/6-311+G(d,p)//B97D3/6-31g(d,p), 298 K).

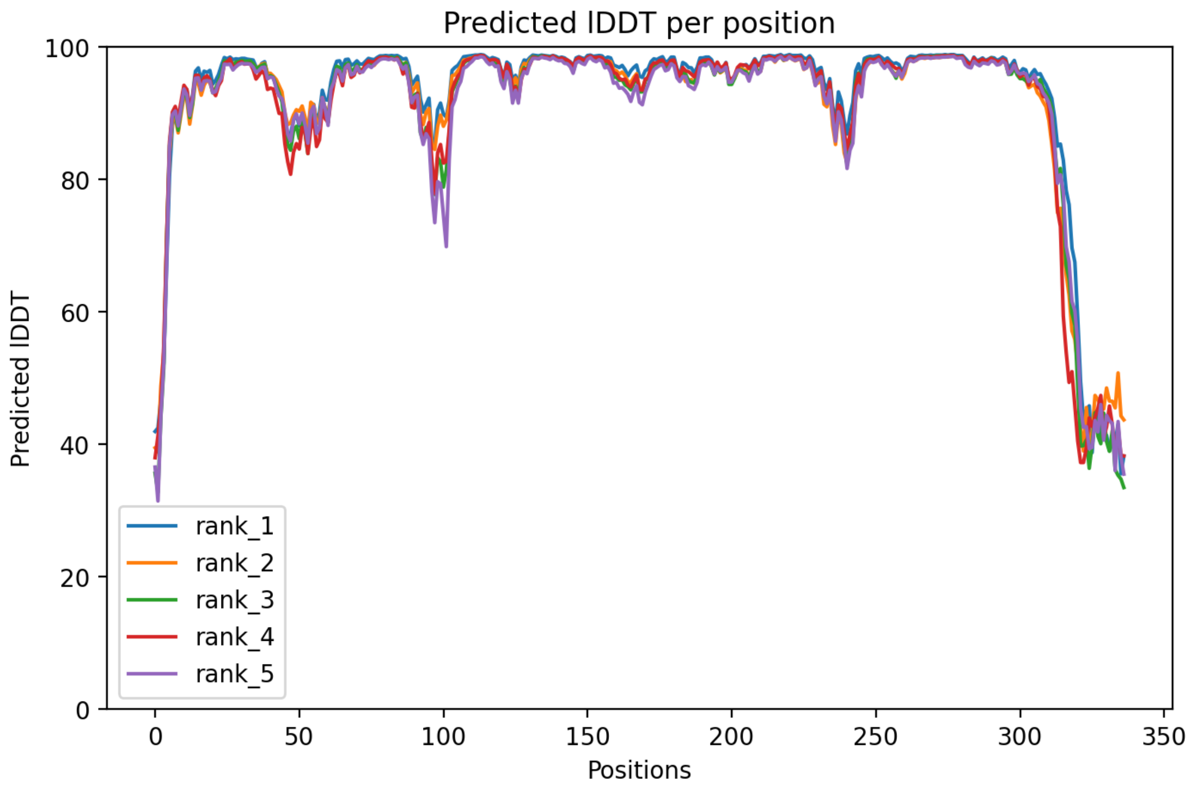

Figure S55. The predicted local distance difference test (pLDDT) for the AlphaFold2 model of CjCS. The score is a per-residue measure of local confidence, scaled from 0 to 100, with higher scores indicating higher confidence and usually a more accurate prediction. All residues, with the exception of D238, targeted by site-directed mutagenesis show a score of >90.

Site-directed mutagenesis

Point mutations were introduced using mutational primers (Table S8) in an overlap extension PCR (OE-PCR).[31] The expression plasmid that contained the wildtype gene was used as a template, and PCR amplifications were performed using Q5 High-Fidelity DNA polymerase (NEB, Ipswich, Massachusetts, USA). The mutational primers were used to amplify the CjCS gene in two overlapping fragments. These first-round PCRs were performed using the following temperature program: 1) 98 °C for 30 s; 2) 98 °C for 10 s, 68 °C for 30 s, 72 °C for 40 s; repeated 35 times; 3) 72 °C for 2 min. In the second-round PCR the two fragments were mixed. Amplification was achieved through the following process. Step 1: 1) 98 °C for 30 s; 2) 98 °C for 10 s, 70 °C for 30 s, 72 °C for 40 s; repeated 5 times; 3) 72 °C for 2 min. Step 2: addition of primers 46C9 and 46D1 for amplification of the whole CjCS gene carrying additional homology arms for cloning into the expression vector pYE-Express by homologous recombination. The PCR was then continued: 1) 98 °C for 30 s; 2) 98 °C for 10 s, 68 °C for 30 s, 72 °C for 40 s; repeated 35 times; 3) 72 °C for 2 min. The amplificates were analysed by DNA gel electrophoresis and purified by the Wizard SV Gel and PCR Clean-Up System (Promega, Madison, Wisconsin, USA). The mutated genes were incorporated into the pET28 based expression vector pYE-Express by homologous recombination in yeast using the standard PEG/LiOAc/salmon sperm protocol.[1,2] *Saccharomyces cerevisiae* cultures containing the plasmids were grown on SM URA plates for 3 days. The colonies were collected to isolate the plasmid mixture by using the Zymoprep Yeast Plasmid Miniprep II kit (Zymo Research, Irvine, CA, USA). The isolated plasmids were introduced into *E. coli* BL21(DE3) electrocompetent cells by electroporation. Cells were plated on LB medium and grown over night. Single colonies were picked and used to inoculate LB cultures (3 mL, containing kanamycin). After incubation at 37 °C for 12 h, the plasmids were extracted using the PureYield Plasmid Miniprep System (Promega) and verified by DNA sequencing. The transformants containing the correct mutations were used for gene expression and protein purification (Figure S56) that were performed as described above for the wildtype enzyme.

Table S8. Primers used for site-directed mutagenesis of CjCS.

**Enzyme variant**	**Primer**	**Nucleotide sequence (5‘ → 3‘)[a]**
CjCS wildtype	46C9	GGCAGCCATATGGCTAGCATGACTGGTGGAATGAA TACAGAAAATTTTAGT
	46D1	TCTCAGTGGTGGTGGTGGTGGTGCTCGAGTGTTATA TTTTATTCAGCTTTTC
I66W		
Fragment 1	46D2	ATGAATACAGAAAATTTTAGT
	51I6	AAAACAGCGCAT**TGG**GCCGCTCAATGG
Fragment 2	51I5	CCATTGAGCGGC**CCA**ATGCGCTGTTTT
	46D3	TTATATTTTATTCAGCTTTTC
I66Y		
Fragment 1	46D2	ATGAATACAGAAAATTTTAGT
	51I8	AAAACAGCGCAT**TAT**GCCGCTCAATGG
Fragment 2	51I7	CCATTGAGCGGC**ATA**ATGCGCTGTTTT
	46D3	TTATATTTTATTCAGCTTTTC
I66F		
Fragment 1	46D2	ATGAATACAGAAAATTTTAGT
	51I4	GACTAAAACAGCGCAT**TTT**GCCGCTCAATGGTTTC
Fragment 2	51I3	GAAACCATTGAGCGGC**AAA**ATGCGCTGTTTTAGTC
	46D3	TTATATTTTATTCAGCTTTTC
I66A		
Fragment 1	46D2	ATGAATACAGAAAATTTTAGT
	51I2	AAAACAGCGCAT**GCG**GCCGCTCAATGG
Fragment 2	51I1	CCATTGAGCGGC**CGC**ATGCGCTGTTTT
	46D3	TTATATTTTATTCAGCTTTTC
M86F		
Fragment 1	46D2	ATGAATACAGAAAATTTTAGT
	51A3	ATTTGTAGATTA**TTT**CTTTGGACCCTT
Fragment 2	51A2	AAGGGTCCAAAG**AAA**TAATCTACAAAT
	46D3	TTATATTTTATTCAGCTTTTC

Enzyme variant	Primer	Nucleotide sequence (5‘ → 3‘)[a]
M86I		
Fragment 1	46D2	ATGAATACAGAAAATTTTAGT
	51A5	ATTTGTAGATTA**ATT**CTTTGGACCCTT
Fragment 2	51A4	AAGGGTCCAAAG**AAT**TAATCTACAAAT
	46D3	TTATATTTTATTCAGCTTTTC
M86L		
Fragment 1	46D2	ATGAATACAGAAAATTTTAGT
	52A7	ATTTGTAGATTA**CTG**CTTTGGACCCTT
Fragment 2	52A6	AAGGGTCCAAAG**CAG**TAATCTACAAAT
	46D3	TTATATTTTATTCAGCTTTTC
M86A		
Fragment 1	46D2	ATGAATACAGAAAATTTTAGT
	52A1	ATTTGTAGATTA**GCG**CTTTGGACCCTT
Fragment 2	51I9	AAGGGTCCAAAG**CGC**TAATCTACAAAT
	46D3	TTATATTTTATTCAGCTTTTC
L90F		
Fragment 1	46D2	ATGAATACAGAAAATTTTAGT
	53C5	ATGCTTTGGACC**TTT**TATAATGATGAT
Fragment 2	53C4	ATCATCATTATA**AAA**GGTCCAAAGCAT
	46D3	TTATATTTTATTCAGCTTTTC
L90I		
Fragment 1	46D2	ATGAATACAGAAAATTTTAGT
	53D2	ATGCTTTGGACC**ATT**TATAATGATGAT
Fragment 2	53D1	ATCATCATTATA**AAT**GGTCCAAAGCAT
	46D3	TTATATTTTATTCAGCTTTTC
L90V		
Fragment 1	46D2	ATGAATACAGAAAATTTTAGT
	53C9	ATGCTTTGGACC**GTG**TATAATGATGAT
Fragment 2	53C8	ATCATCATTATA**CAC**GGTCCAAAGCAT
	46D3	TTATATTTTATTCAGCTTTTC

Enzyme variant	Primer	Nucleotide sequence (5‘ → 3‘)[a]
L90A		
Fragment 1	46D2	ATGAATACAGAAAATTTTAGT
	53C7	ATGCTTTGGACC**GCG**TATAATGATGAT
Fragment 2	53C6	ATCATCATTATA**CGC**GGTCCAAAGCAT
	46D3	TTATATTTTATTCAGCTTTTC
I188L		
Fragment 1	46D2	ATGAATACAGAAAATTTTAGT
	53D8	CGCGAAAATTCC**CTG**TGTCTATATCCT
Fragment 2	53D7	AGGATATAGACA**CAG**GGAATTTTCGCG
	46D3	TTATATTTTATTCAGCTTTTC
I188V		
Fragment 1	46D2	ATGAATACAGAAAATTTTAGT
	53D4	CGCGAAAATTCC**GTG**TGTCTATATCCT
Fragment 2	53D3	AGGATATAGACA**CAC**GGAATTTTCGCG
	46D3	TTATATTTTATTCAGCTTTTC
I188A		
Fragment 1	46D2	ATGAATACAGAAAATTTTAGT
	53D6	CGCGAAAATTCC**GCG**TGTCTATATCCT
Fragment 2	53D5	AGGATATAGACA**CGC**GGAATTTTCGCG
	46D3	TTATATTTTATTCAGCTTTTC
C189S		
Fragment 1	46D2	ATGAATACAGAAAATTTTAGT
	53E1	GAAAATTCCATC**TCT**CTATATCCTTTT
Fragment 2	53D9	AAAAGGATATAG**AGA**GATGGAATTTTC
	46D3	TTATATTTTATTCAGCTTTTC
C189A		
Fragment 1	46D2	ATGAATACAGAAAATTTTAGT
	53E3	GAAAATTCCATC**GCG**CTATATCCTTTT
Fragment 2	53E2	AAAAGGATATAG**CGC**GATGGAATTTTC
	46D3	TTATATTTTATTCAGCTTTTC

**Enzyme variant**	**Primer**	**Nucleotide sequence (5‘ → 3‘)[a]**
L190I		
Fragment 1	46D2	ATGAATACAGAAAATTTTAGT
	53E5	AATTCCATCTGT**ATT**TATCCTTTTCTT
Fragment 2	53E4	AAGAAAAGGATA**AAT**ACAGATGGAATT
	46D3	TTATATTTTATTCAGCTTTTC
L190V		
Fragment 1	46D2	ATGAATACAGAAAATTTTAGT
	53E9	AATTCCATCTGT**GTG**TATCCTTTTCTT
Fragment 2	53E8	AAGAAAAGGATA**CAC**ACAGATGGAATT
	46D3	TTATATTTTATTCAGCTTTTC
L190A		
Fragment 1	46D2	ATGAATACAGAAAATTTTAGT
	53E7	AATTCCATCTGT**GCG**TATCCTTTTCTT
Fragment 2	53E6	AAGAAAAGGATA**CGC**ACAGATGGAATT
	46D3	TTATATTTTATTCAGCTTTTC
D238E		
Fragment 1	46D2	ATGAATACAGAAAATTTTAGT
	53C3	TCTGTTATAAAA**GAA**GAGGCAACGGGT
Fragment 2	53C2	ACCCGTTGCCTC**TTC**TTTTATAACAGA
	46D3	TTATATTTTATTCAGCTTTTC

[a] Homology arms for gene cloning are underlined. Changed triplet codons are shown in bold.

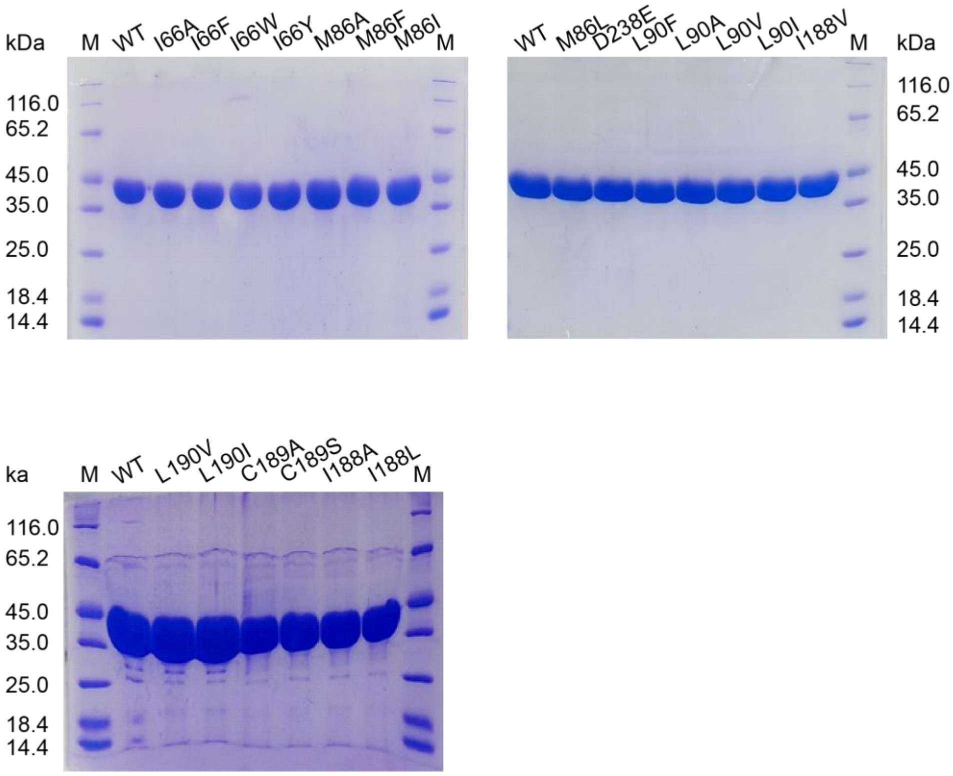

Figure S56. SDS-PAGE analysis of the purified CjCS variants.

Relative productivity of enzyme variants

The concentrations of the purified proteins were determined by Bradford assay[3] and adjusted to 0.8 mg mL^{-1} for all enzyme variants for activity testings. The protein preparation (0.1 mL), incubation buffer (0.8 mL; 50 mM TRIS, 10 mM $MgCl_2$, 10% glycerol, pH 8.2), and GGPP (0.1 mL; 1 mg mL^{-1} in 25 mM aqueous NH_4HCO_3) were mixed and incubated at 28 °C overnight. The reaction was extracted with hexane (0.5 mL; containing 0.08 mg mL^{-1} tetradecane as internal standard). The layers were separated by centrifugation (16,000 rpm, 5 min). The organic layer was pipetted off and analysed by GC/MS (Figures S57 – S59). Triplicates were performed for every enzyme variant, and productivities were determined by peak integration of all diterpene products. The sum of the production of all compounds by the wild-type (mean) was set to 100% (± standard deviation).

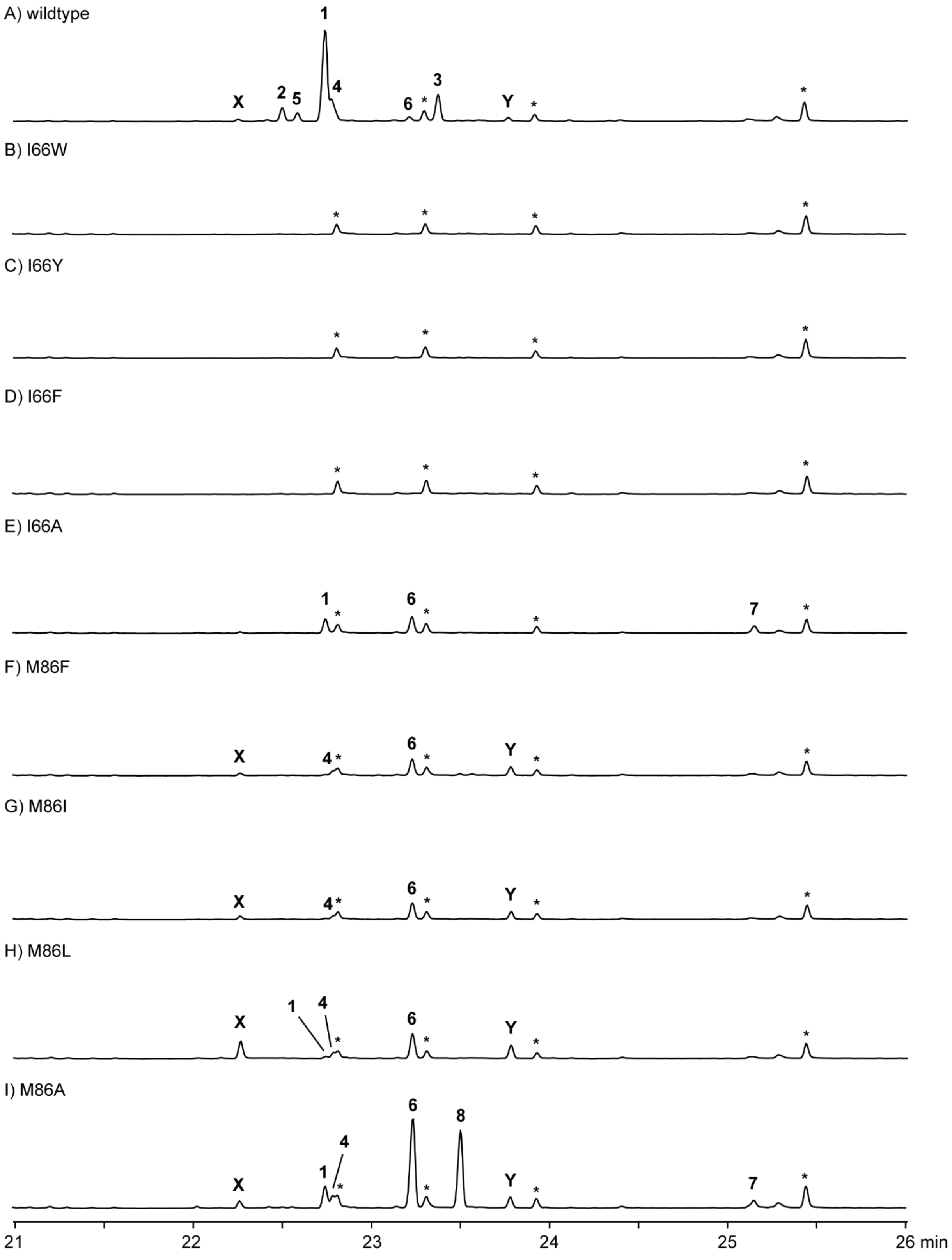

Figure S57. Total ion chromatograms of extracts from incubations of CjCS and its enzyme variants.

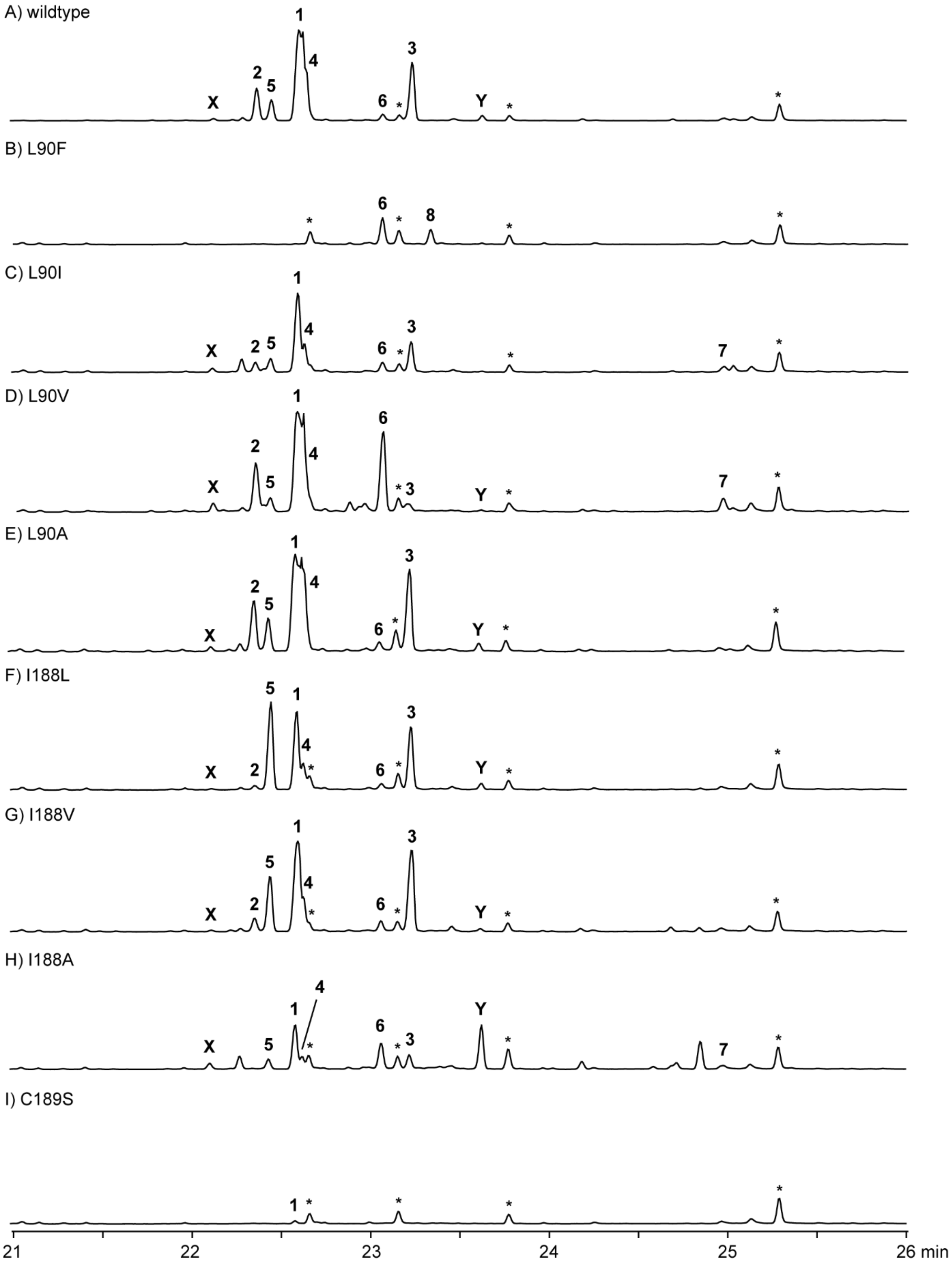

Figure S58. Total ion chromatograms of extracts from incubations of CjCS and its enzyme variants.

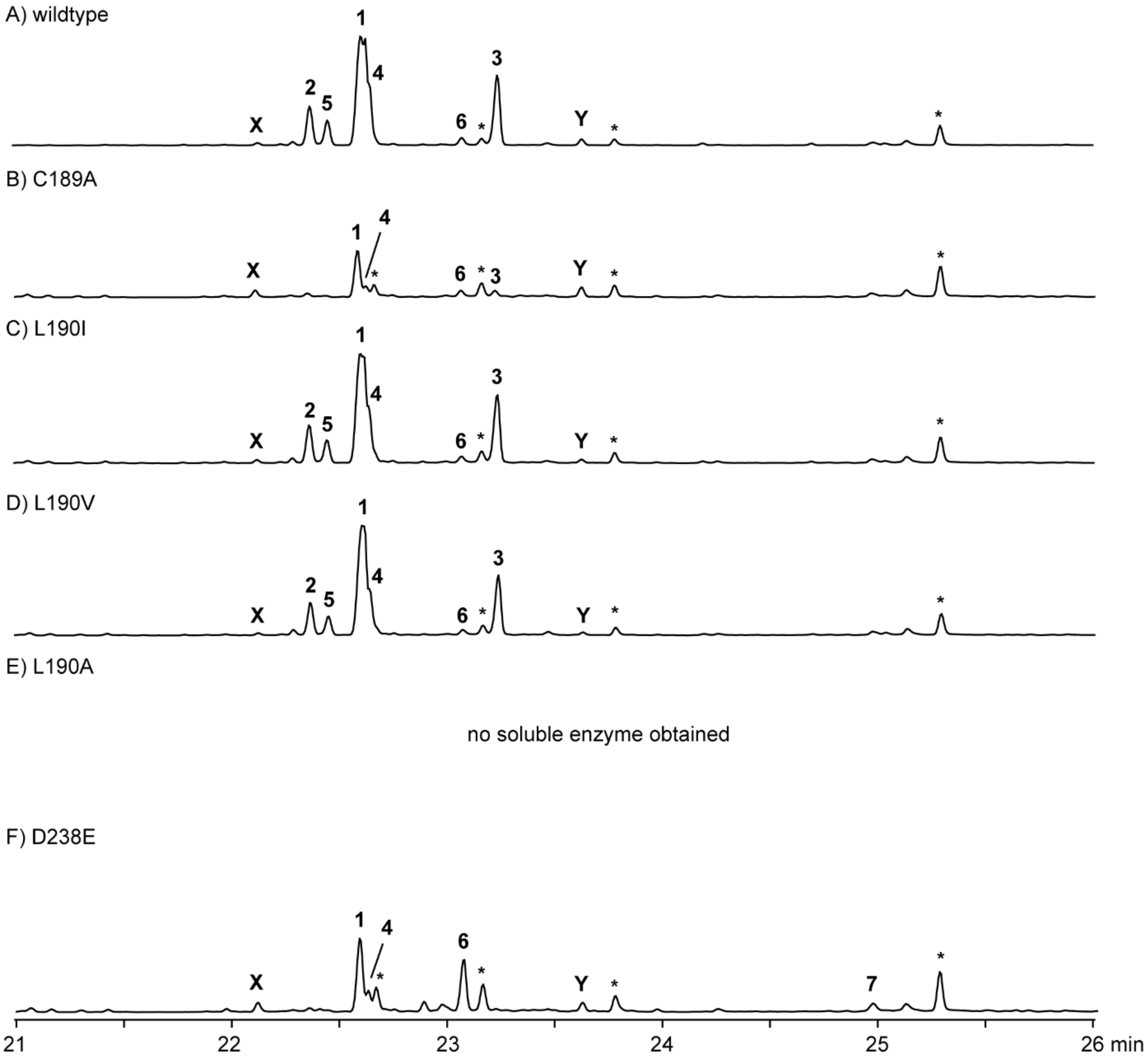

Figure S59. Total ion chromatograms of extracts from incubations of CjCS and its enzyme variants.

Table S9. Results obtained with CjCS enzyme variants in this study.

**Variant**	**Total[a]**	**1 + 4[b]**	**2**	**3**	**5**	**6**	**7**	**8**	**X[c]**	**Y[c]**
wildtype	100±14	66±9	7.1±1.2	16±2	4.8±0.8	2.9±0.5	0	0	1.2±0.3	2.1±0.5
I66W	0	0	0	0	0	0	0	0	0	0
I66Y	0	0	0	0	0	0	0	0	0	0
I66F	0	0	0	0	0	0	0	0	0	0
I66A	29±3	10±1	0	0	0	12±2	6.9±0.4	0	0	0
M86F	32±1	10±1	0	0	0	13±0	0	0	1.9±0.1	6.8±0.2
M86I	30±6	10±0	0	0	0	13±2	0	0	2.7±0.4	4.5±3.7
M86L	47±6	11±1	0	0	0	16±2	0	0	12±1	8.8±1.1
M86A	118±9	19±3	0	0	0	46±3	5.8±0.0	39±2	3.5±0.3	5.9±0.3
L90F	17±1	11±1	0	0	0	0	0	6.2±0.5	0	0
L90I	62±5	37±3	3.2±0.3	10±1	5.1±0.4	3.6±0.4	2.3±0.1	0	1.4±0.2	0
L90V	53±1	23±0	3.5±0.0	7.0±0.0	2.4±0.3	3.4±0.1	7.8±0.3	0	1.8±0.1	3.6±0.1
L90A	132±1	72±0	16±0	27±0	10±0	3.4±0.1	0	0	1.4±0.0	2.6±0.0
I188L	78±10	29±4	1.4±0.2	18±2	25±3	2.0±0.1	0	0	0.4±0.0	1.8±0.1
I188V	102±6	47±2	4.3±0.3	29±2	18±1	3.5±0.3	0	0	0.5±0.1	1.1±0.1
I188A	55±2	19±1	0	5.3±0.1	3.6±0.1	9.3±0.4	2.4±0.0	0	2.2±0.1	14±1
C189S	0	0	0	0	0	0	0	0	0	0
C189A	22±2	13±1	1.1±0.1	1.9±0.2	0	1.8±0.1	0	0	1.6±0.1	0
L190I	98±3	58±1	10±0	19±1	6.2±0.3	2.2±0.1	0	0	0.9±0.1	1.1±0.0
L190V	90±6	55±3	9.1±0.9	17±2	5.5±0.5	2.0±0.2	0	0	0.8±0.1	1.1±0.1
L190A[d]	–	–	–	–	–	–	–	–	–	–
D238E	39±2	20±1	0	0	0	11±1	3.0±0.1	0	2.1±0.1	2.3±0.2

[a] Relative production in % with the total production by the wildtype set to 100%. The data represent mean ± standard deviation from triplicates. [b] Compounds **1** and **4** coelute in the GC/MS analysis. [c] Unidentified compounds. [d] No expression of soluble enzyme.

Table S10. Preparative scale conversions with enzyme variants.

Enzyme variants	Substrate	Scale	Isolated compounds
CjCS-M86A	GGPP	80 mg (160 μmol)	**1** (0.71 mg, 2.6 μmol, 1.6%)
			**4** (0.56 mg, 2.1 μmol, 1.2%)
			**6** (3.1 mg, 1.4 μmol, 7.1%)
			**7** (0.90 mg, 3.1 μmol, 1.9%)
			**8** (0.36 mg, 1.3 μmol, 0.08%)
CjCS-I188L	GGPP	80 mg (160 μmol)	**1** (0.92 mg, 3.3 μmol, 2.1%)
			**3** (0.81 mg, 2.9 μmol, 1.8%)
			**4** (0.62 mg, 2.3 μmol, 1.4%)
			**5** (3.1 mg, 1.4 μmol, 7.1%)

Expression and purification of enzyme variants
For preparative scale enzymatic conversions (Table S10), a solution of the trisammonium salt of GGPP (80 mg, 160 μmol) in substrate buffer (20 mL) was added to incubation buffer (100 mL) containing the respective enzyme variant (0.6 mg L^{-1}), followed by incubation for 16 h at 30 °C. The reaction mixtures were extracted with *n*-hexane, the extracts were dried with $MgSO_4$ and the solvent was evaporated, followed by compound isolation through column chromatography on silica gel or on silica gel activated with $AgNO_3$. For the activation of silica gel with $AgNO_3$, 50 g of silica gel were suspended in a solution of $AgNO_3$ (2.5 g) in methanol (150 mL) overnight, followed by rigorous evaporation of the solvent.

Chryseojoostene D (4). TLC (pentane): R_f = 0.55. GC (HP-5MS): *I* = 1921. MS (EI, 70 eV): *m*/*z* (%) = 272 (16), 257 (13), 243 (2), 229 (4), 216 (6), 203 (6), 189 (26), 175 (28), 161 (35), 147 (26), 135 (77), 121 (100), 107 (65), 93 (70), 79 (44), 67 (26), 55 (20), 41 (15). IR (diamond ATR): ṽ = 2952 (s), 2923 (s), 2853 (s), 1661 (w), 1632 (w), 1452 (w), 1381 (m), 1370 (w), 1260 (s), 1102 (w), 1088 (w), 1053 (w), 1018 (w), 820 (w), 801 (m) cm^{-1}. ESI-(+)-HR-MS: calc. for $[C_{20}H_{33}]^+$ *m*/*z* = 273.2577; found: *m*/*z* = 273.2577. Optical rotation: $[\alpha]_D^{25}$ = +28.5 (*c* 0.06, CH_2Cl_2). NMR data are given in Table S11.

12-*epi*-Dolabella-3,7,18-triene (6). TLC (pentane): R_f = 0.49. GC (HP-5MS): *I* = 1961. MS (EI, 70 eV): *m*/*z* (%) = 272 (18), 257 (14), 243 (2), 229 (21), 215 (11), 201 (9), 189 (45), 175 (24), 161 (41), 147 (51), 135 (69), 121 (91), 107 (100), 93 (97), 79 (77), 67 (71), 55 (37), 41 (28). IR (diamond ATR): ṽ = 2942 (s), 2918 (s), 2855 (s), 1665 (w), 1445 (m), 1376 (m), 1260 (w), 1186 (w), 1095 (m), 1018 (m), 927 (s), 885 (w), 800 (m) cm^{-1}. ESI-(+)-HR-MS: calc. for $[C_{20}H_{33}]^+$ *m*/*z* = 273.2577; found: *m*/*z* = 273.2577. Optical rotation: $[\alpha]_D^{25}$ = +41.4 (*c* 0.31, CH_2Cl_2). NMR data are given in Table S12.

12-*epi*-18-Hydroxydolabella-3,7-diene (7). TLC (pentane/Et_2O 4:1): R_f = 0.56. GC (HP-5MS): *I* = 2165. MS (EI, 70 eV): *m*/*z* (%) = 290 (2), 272 (15), 257 (12), 243 (2), 229 (20), 216 (6), 203 (8), 189 (30), 175 (17), 161 (40), 148 (31), 135 (74), 121 (65), 108 (66), 95 (77), 81 (69), 67 (57), 59 (100), 41 (32). IR (diamond ATR): ṽ = 3465 (m), 2956 (s), 2923 (s), 2852 (s), 1668 (w), 1448 (m), 1375 (m), 1206 (w), 1150 (w), 1093 (w), 1021 (w), 935 (w), 923 (w), 835 (w) cm⁻

[1]. ESI-(+)-HR-MS: calc. for $[C_{20}H_{35}O]^+$ *m*/*z* = 291.2682; found: *m*/*z* = 291.2682. Optical rotation: $[\alpha]_D^{25}$ = +22.2 (*c* 0.09, CH_2Cl_2). NMR data are given in Table S13.

Cembrene A (8). TLC (pentane): R_f = 0.50. GC (HP-5MS): *I* = 1990. MS (EI, 70 eV): *m*/*z* (%) = 272 (8), 257 (18), 244 (1), 229 (5), 215 (6), 201 (7), 189 (10), 175 (8), 161 (16), 147 (19), 133 (28), 121 (55), 107 (59), 93 (83), 81 (60), 68 (100), 53 (35), 41 (27). IR (diamond ATR): ṽ = 2924 (s), 2853 (s), 1729 (w), 1667 (w), 1643 (w), 1444 (m), 1092 (s), 1375 (w), 1260 (w), 1099 (w), 1050 (w), 1021 (w), 967 (w), 887 (m), 846 (w), 805 (w) cm^{-1}. ESI-(+)-HR-MS: calc. for $[C_{20}H_{33}]^+$ *m*/*z* = 273.2577; found: *m*/*z* = 273.2577. Optical rotation: $[\alpha]_D^{25}$ = +5.5 (*c* 0.04, CH_2Cl_2). NMR data are given in Table S14.

Chryseojoostene E (5). TLC (pentane): R_f = 0.50. GC (HP-5MS): *I* = 1898 MS (EI, 70 eV): *m*/*z* (%) = 272 (18), 257 (24), 243 (6), 229 (6), 216 (26), 201 (15), 189 (25), 175 (37), 162 (40), 147 (41), 133 (58), 121 (100), 107 (86), 91 (90), 79 (56), 67 (49), 55 (37), 41 (34). IR (diamond ATR): ṽ = 2952 (s), 2925 (s), 2854 (s), 1636 (w), 1453 (m), 1382 (w), 1358 (w), 1091 (w), 1027 (w), 880 (m), 846 (w), 815 (w) cm^{-1}. ESI-(+)-HR-MS: calc. $[C_{20}H_{33}]^+$ *m*/*z* = 273.2577; found: *m*/*z* = 273.2577. Optical rotation: $[\alpha]_D^{25}$ = +140.3 (*c* 0.31, CH_2Cl_2). NMR data are given in Table S15.

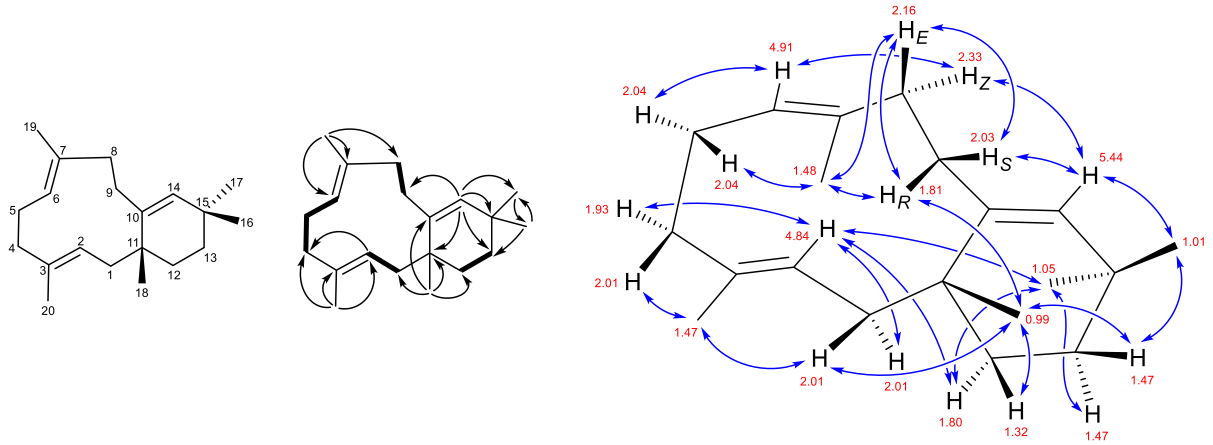

Figure S60. Structure elucidation of chryseojoostene D (**4**). Bold: $^{1}H,^{1}H$-COSY correlations, single headed arrows: HMBC correlations, and blue double headed arrows: NOESY correlations. Carbon numbering follows GGPP numbering to indicate the origin of each carbon. The hydrogen labels H_R, H_S, H_E and H_Z indicate the results from stereoselective deuteration experiments (Figures S92 and S93).

Table S11. NMR data of chryseojoostene D (**4**) in C_6D_6 recorded at 298 K.

C[a]	type	^{13}C[b]	^{1}H[b]
1	CH_2	38.79	2.01 (m, 2H)
2	CH	126.74	4.84 (t, *J* = 5.5)
3	C	131.03	–
4	CH_2	39.21	1.93 (m)
			2.01 (m)
5	CH_2	25.81	2.04 (m, 2H)
6	CH	125.03	4.91 (m)
7	C	138.00	–
8	CH_2	37.86	2.33 (td, *J* = 13.0, 12.4)
			2.16 (dd, *J* = 12.6, 6.5)
9	CH_2	29.73*	2.01 (m)
			1.81 (m)
10	C	140.38	–
11	C	38.92	–
12	CH_2	35.17	1.80 (m)
			1.32 (m)
13	CH_2	34.22	1.47 (m, 2H)
14	CH	134.15	5.44 (d, *J* = 2.5)
15	C	32.35	–
16	CH_3	28.95	1.05 (s)
17	CH_3	31.44	1.01 (s)
18	CH_3	26.88	1.00 (s)
19	CH_3	17.98	1.48 (s)
20	CH_3	16.46	1.47 (s)

[a] Carbon numbering as shown in Figure S60 indicates the origin of each carbon from GGPP by same number. [b] Chemical shifts δ in ppm, multiplicity: s = singlet, d = doublet, t = triplet, m = multiplet, br = broad, coupling constants *J* are given in Hertz. Asterisks indicate carbons showing peak broadening in the ^{13}C-NMR.

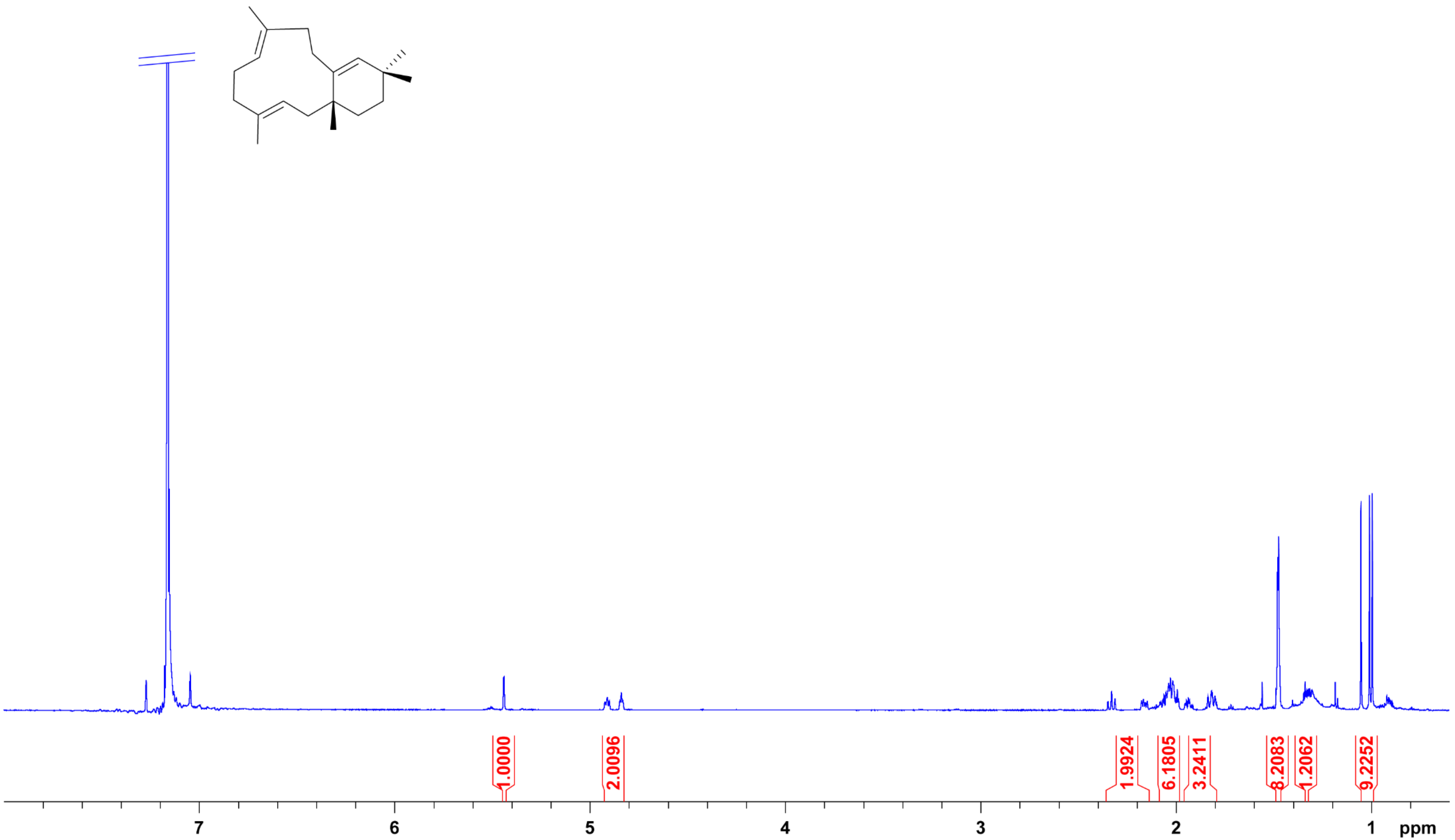

Figure S61. ^{1}H-NMR spectrum of **4** (700 MHz, C_6D_6).

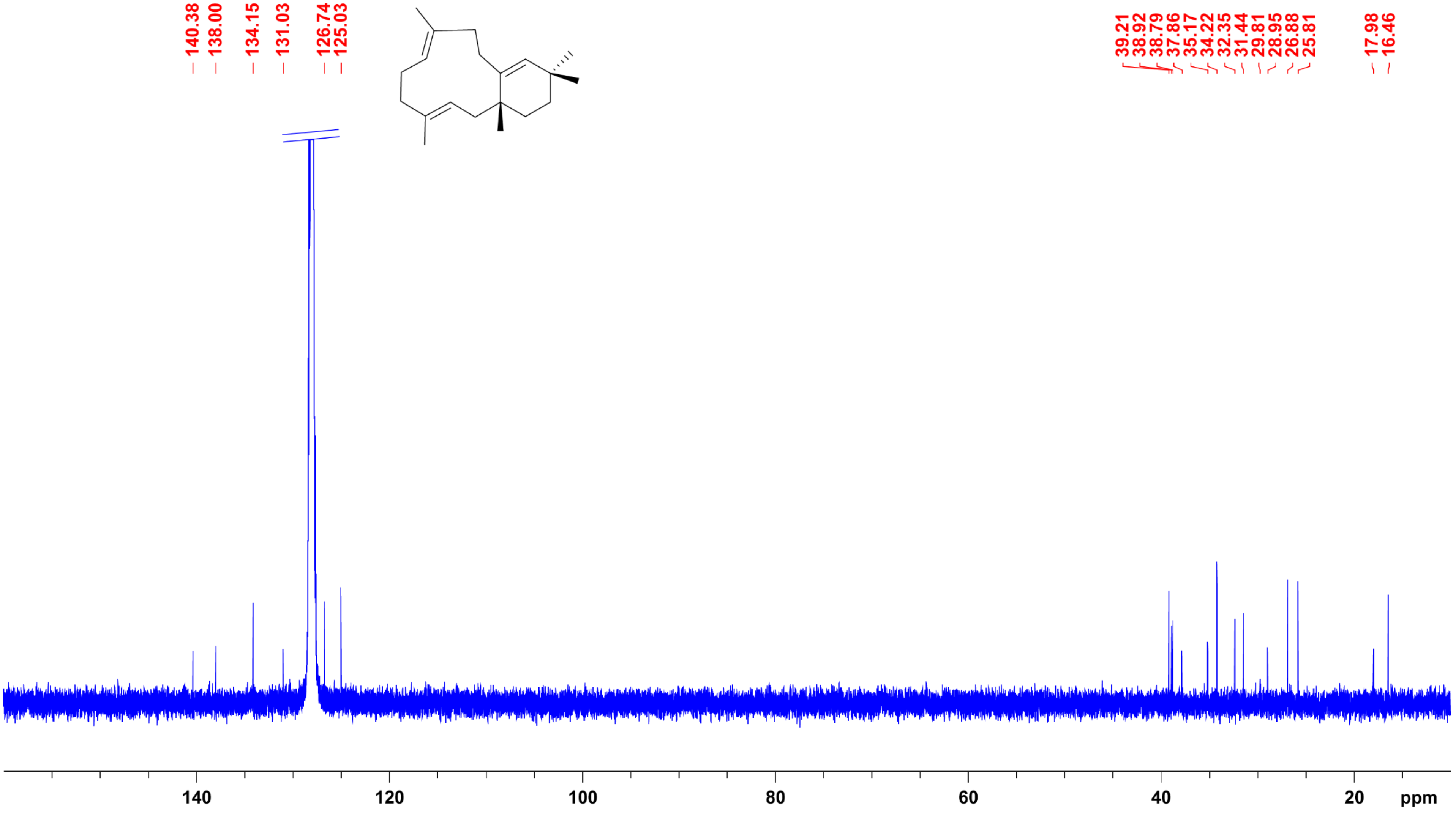

Figure S62. ^{13}C-NMR spectrum of **4** (176 MHz, C_6D_6).

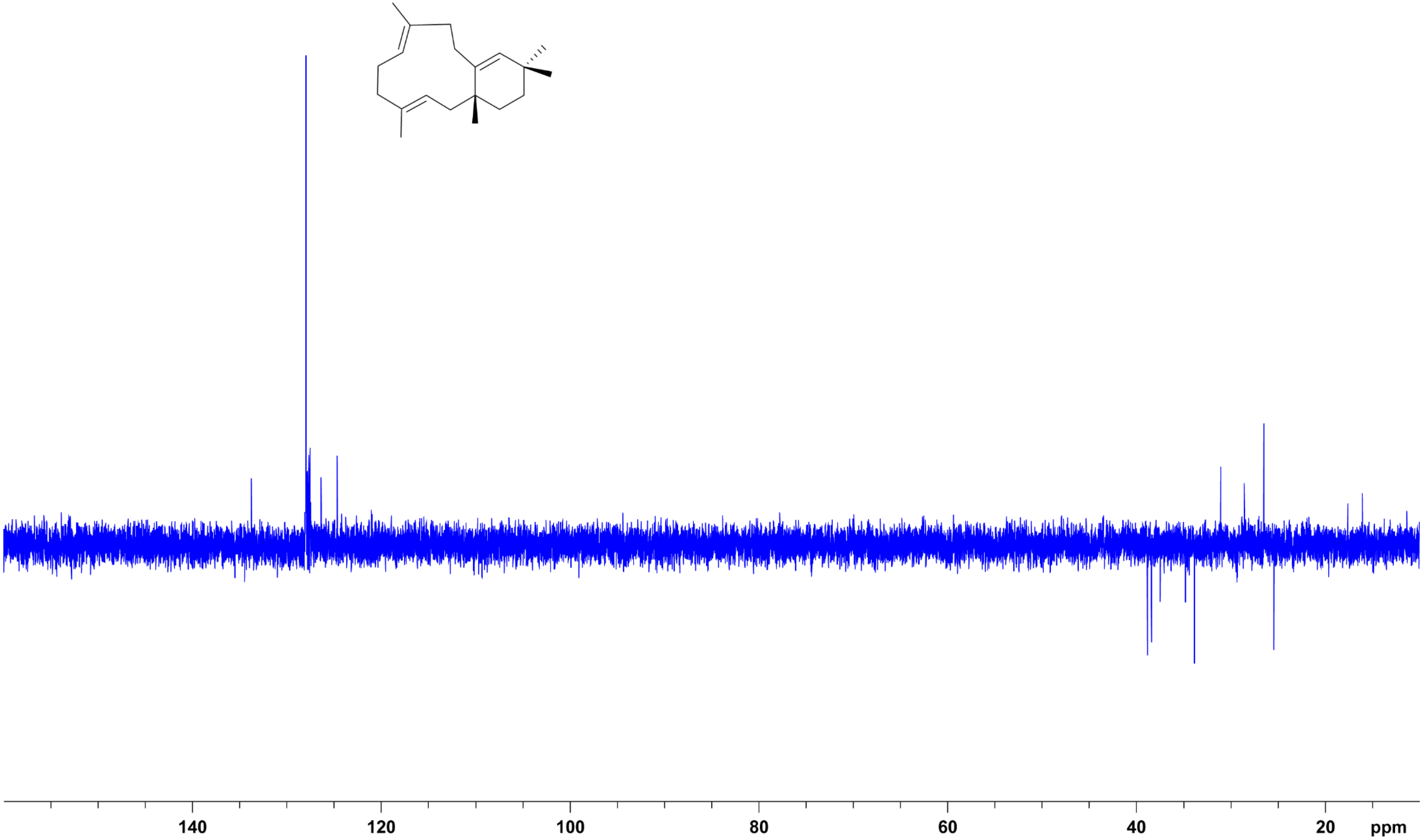

Figure S63. ^{13}C-DEPT135 spectrum of **4** (176 MHz, C_6D_6).

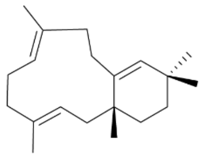

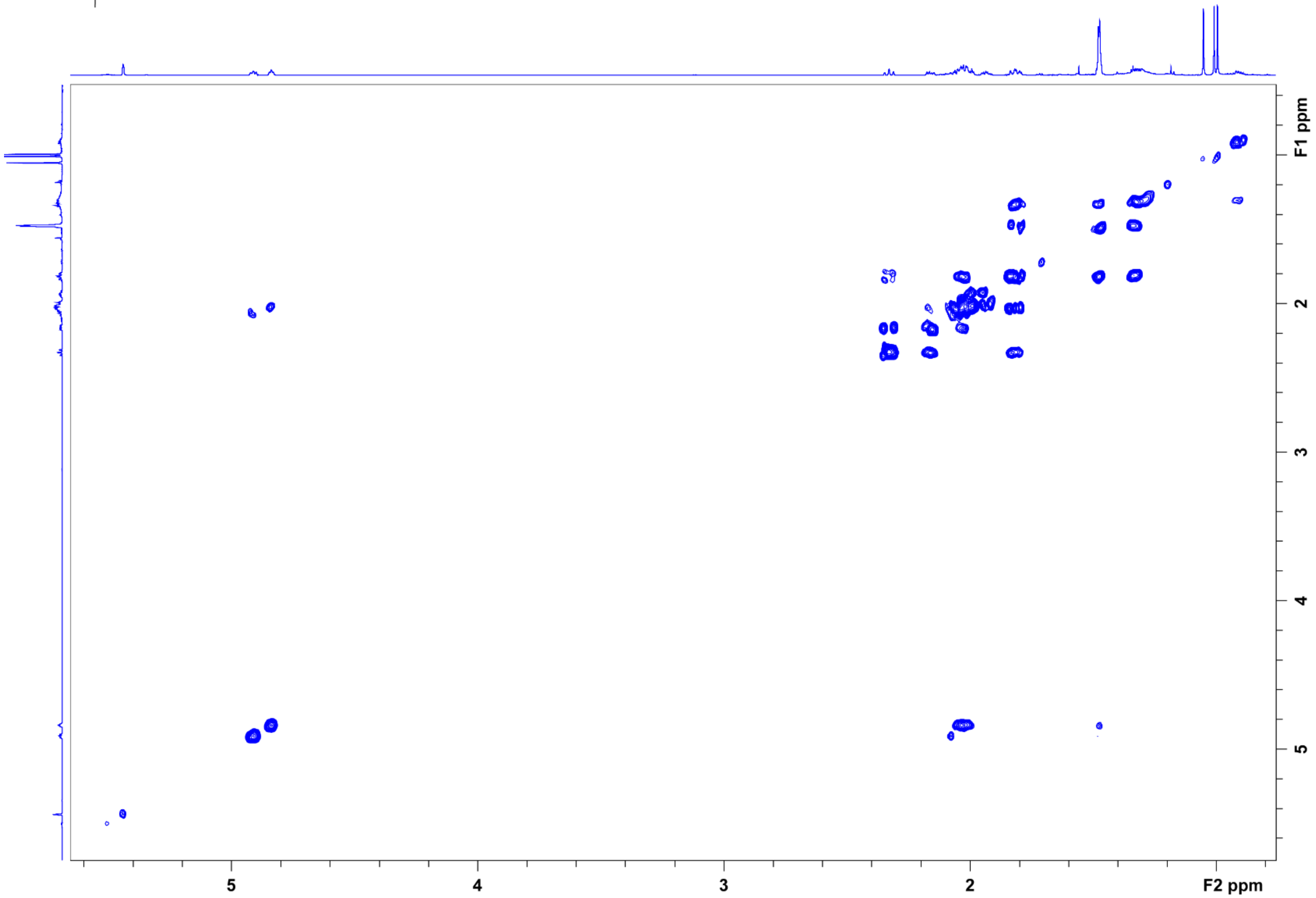

Figure S64. ^{1}H,^{1}H-COSY spectrum (C_6D_6) of **4**.

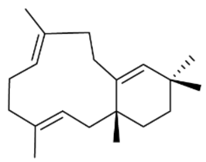

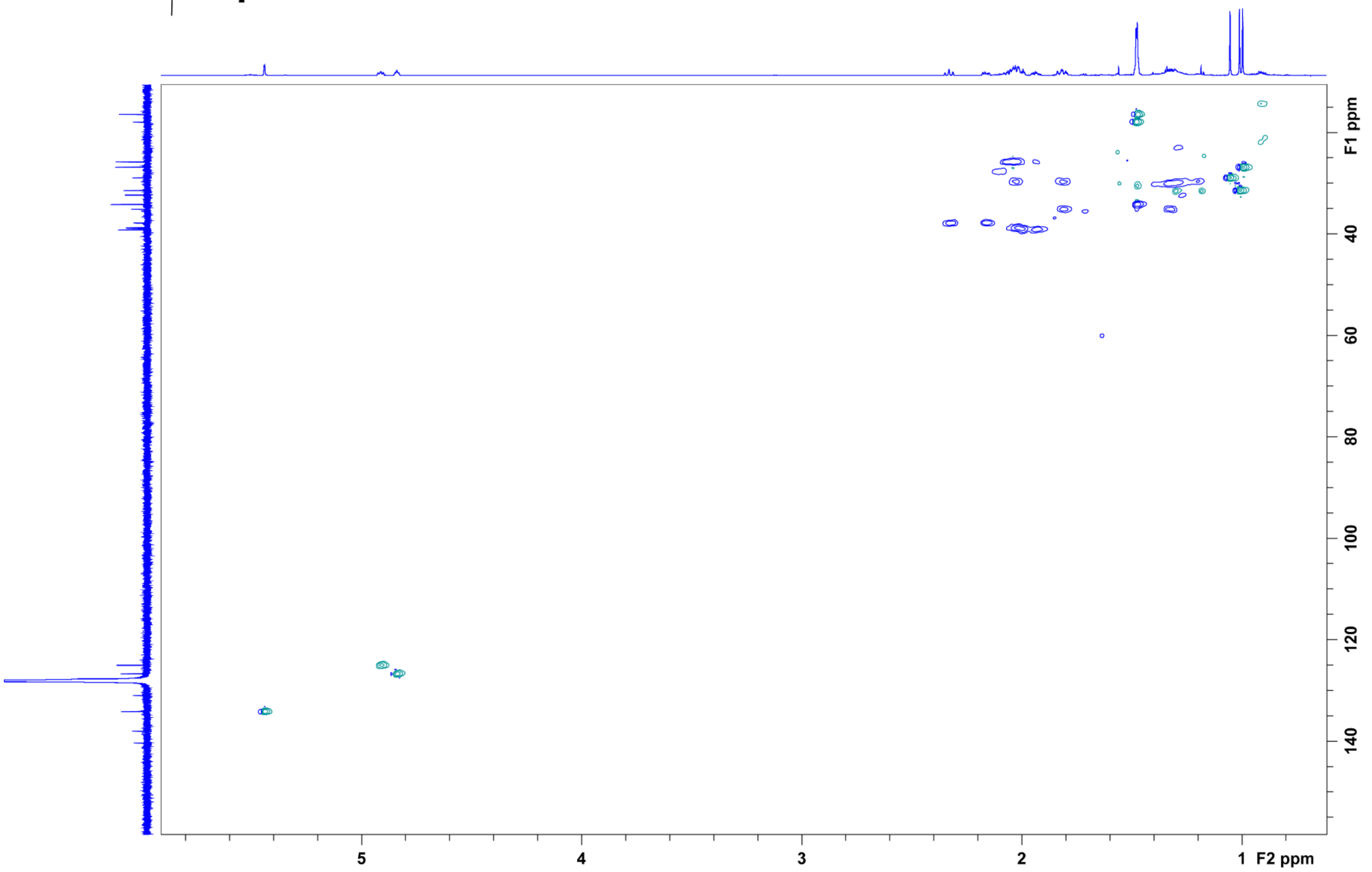

Figure S65. HSQC spectrum (C_6D_6) of **4**.

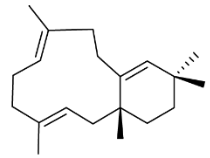

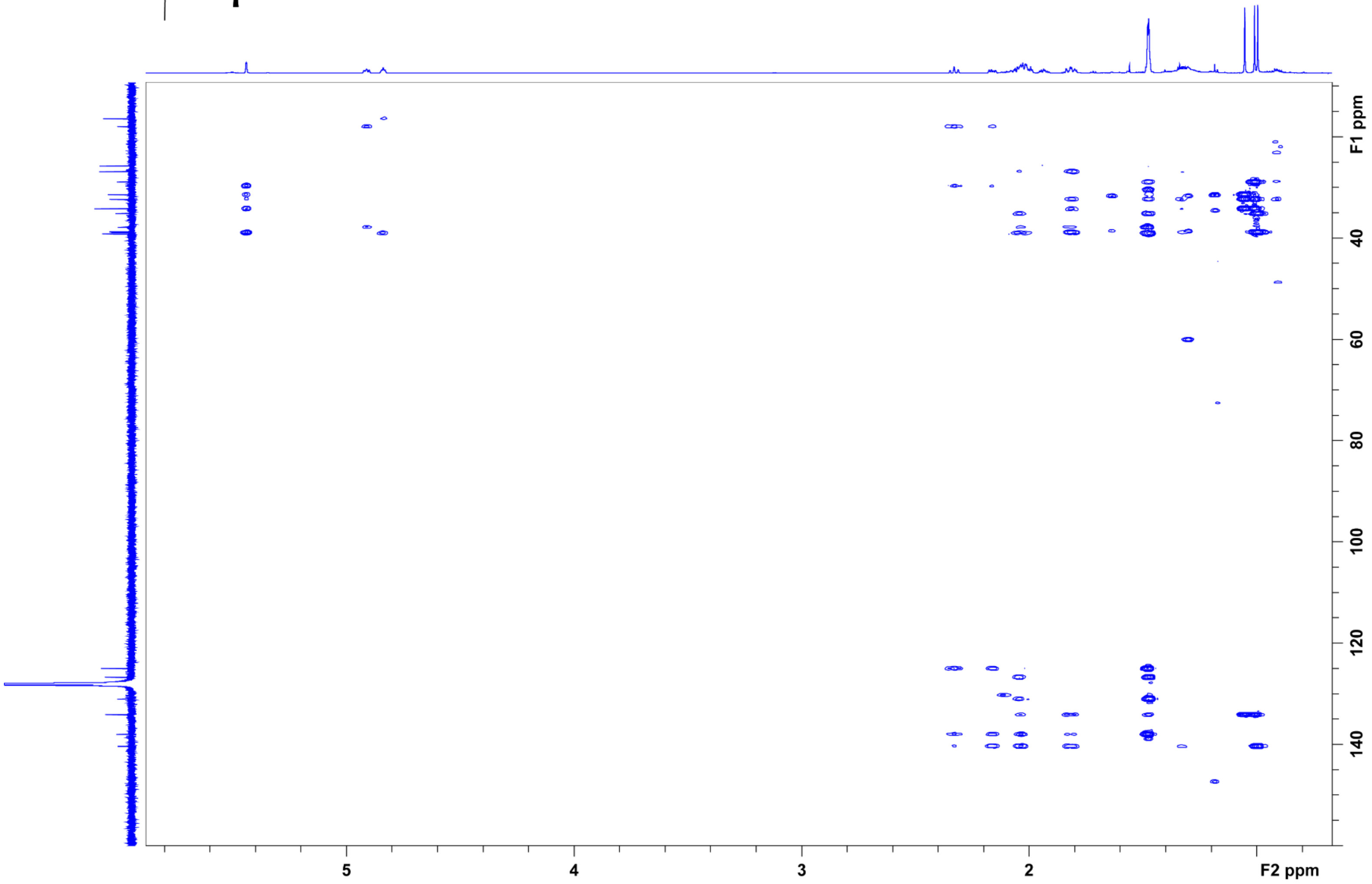

Figure S66. HMBC spectrum (C_6D_6) of **4**.

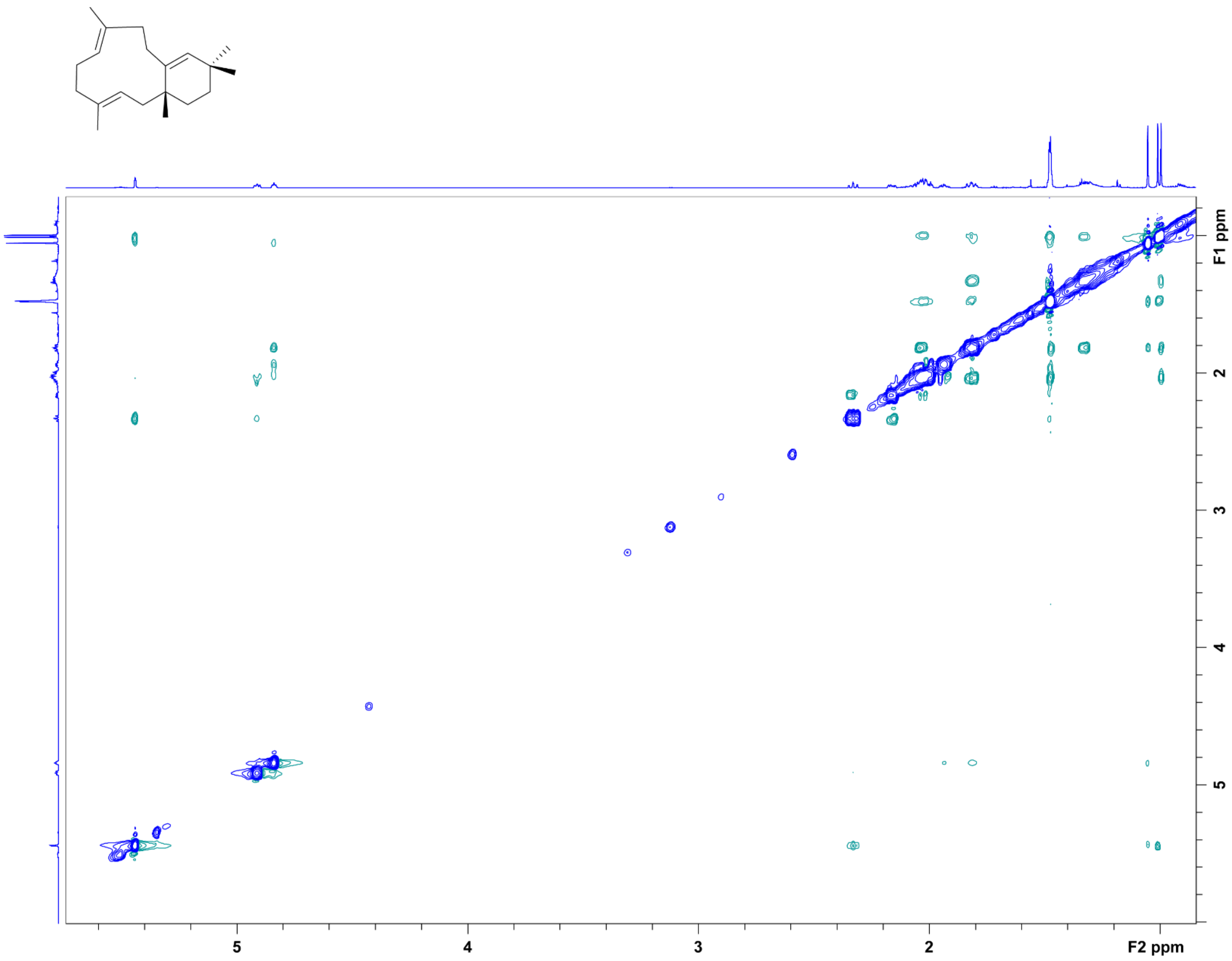

Figure S67. NOESY spectrum (C_6D_6) of **4**.

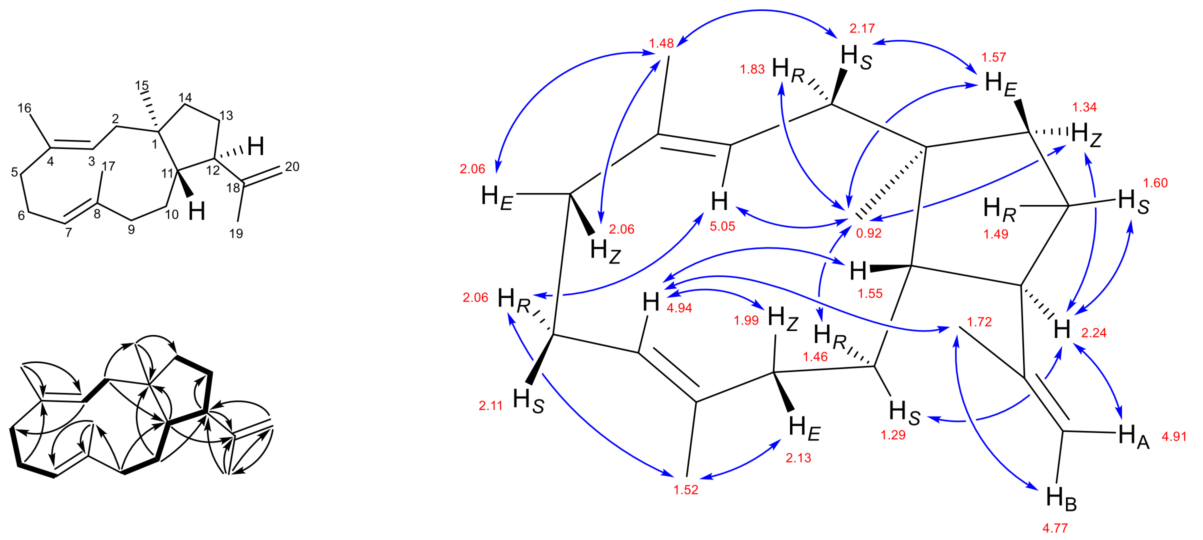

Figure S68. Structure elucidation of 12-*epi*-dolabela-3,7,18-triene (**6**). Bold: ^{1}H,^{1}H-COSY correlations, single headed arrows: HMBC correlations, and blue double headed arrows: NOESY correlations. The hydrogen labels H_R, H_S, H_E and H_Z indicate the results from stereoselective deuteration experiments (Figures S96 and S97).

Table S12. NMR data of 12-*epi*-dolabela-3,7,18-triene (**6**) in C_6D_6 recorded at 298 K.

C[a]	type	[13]C[b]	[1]H[b]
1	C	44.96	–
2	CH_2	40.98	2.17 (dd, *J* = 14.1, 8.8)
			1.83 (dd, *J* = 14.1, 5.6)
3	CH	124.52	5.05 (ddd, *J* = 8.8, 5.6, 1.4)
4	C	133.78	–
5	CH_2	39.83	2.06 (m, 2H)
6	CH_2	24.99	2.11 (m)
			2.06 (m)
7	CH	125.55	4.94 (m)
8	C	135.59	–
9	CH_2	37.58	2.13 (m)
			1.99 (ddd, *J* = 13.4, 10.7, 2.8)
10	CH_2	31.28	1.46 (m)
			1.29 (m)
11	CH	44.68	1.55 (m)
12	CH	58.32	2.24 (td, *J* = 9.9, 7.4)
13	CH_2	29.17	1.60 (m)
			1.49 (m)
14	CH_2	41.93	1.57 (m)
			1.34 (m)
15	CH_3	23.29	0.92 (s)
16	CH_3	15.99	1.48 (d, *J* = 1.3)
17	CH_3	17.53	1.52 (d, *J* = 1.6)
18	C	148.42	–
19	CH_2	111.27	4.91 (d, *J* = 2.7, H_A)
			4.77 (dq, *J* = 2.8, 1.4, H_B)
20	CH_3	19.06	1.72 (t, *J* = 1.2)

[a] Carbon numbering as shown in Figure S68. [b] Chemical shifts δ in ppm, multiplicity: s = singlet, d = doublet, t = triplet, q = quartet, m = multiplet, coupling constants *J* are given in Hertz.

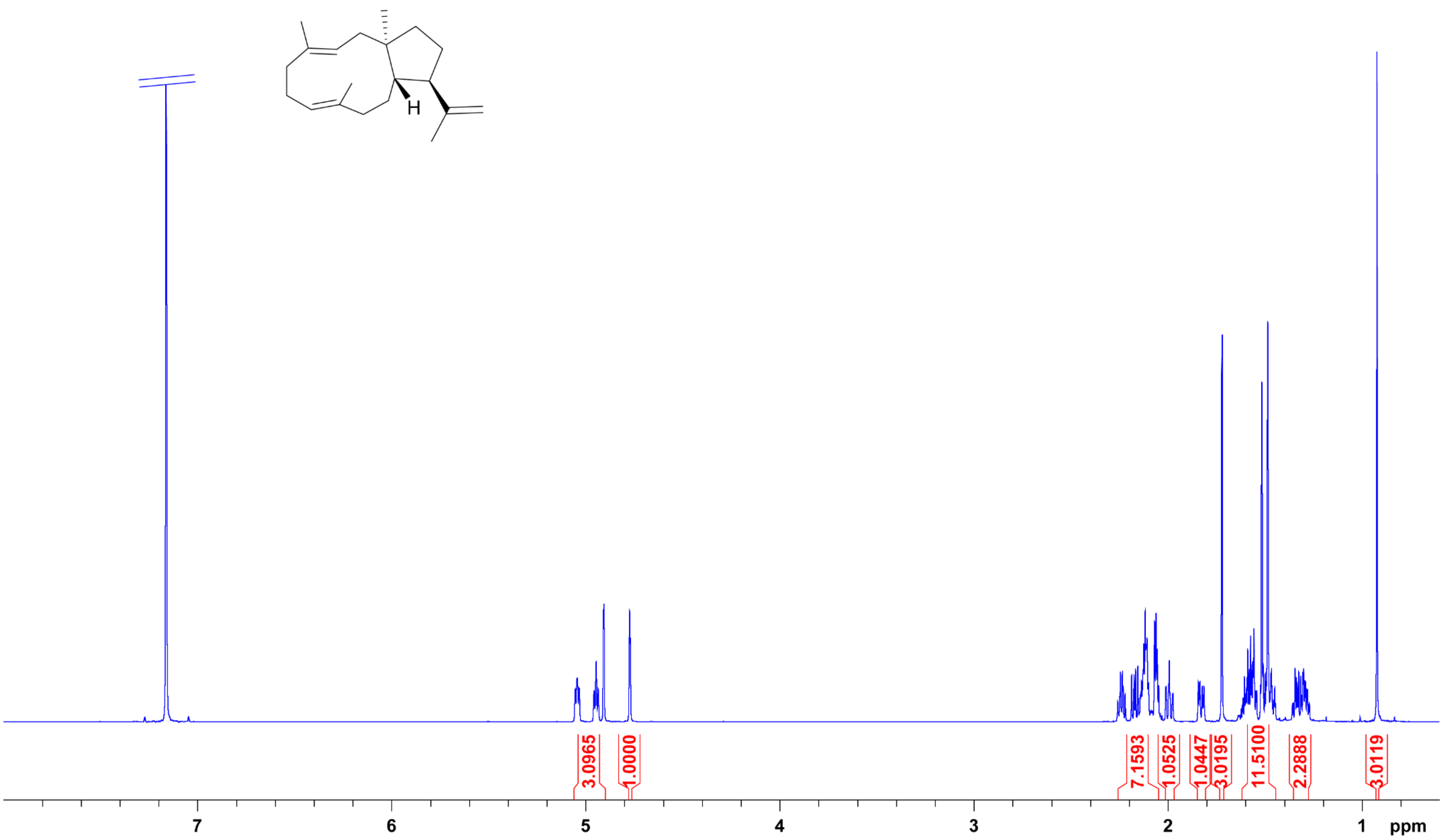

Figure S69. ^{1}H-NMR spectrum of **6** (700 MHz, C_6D_6).

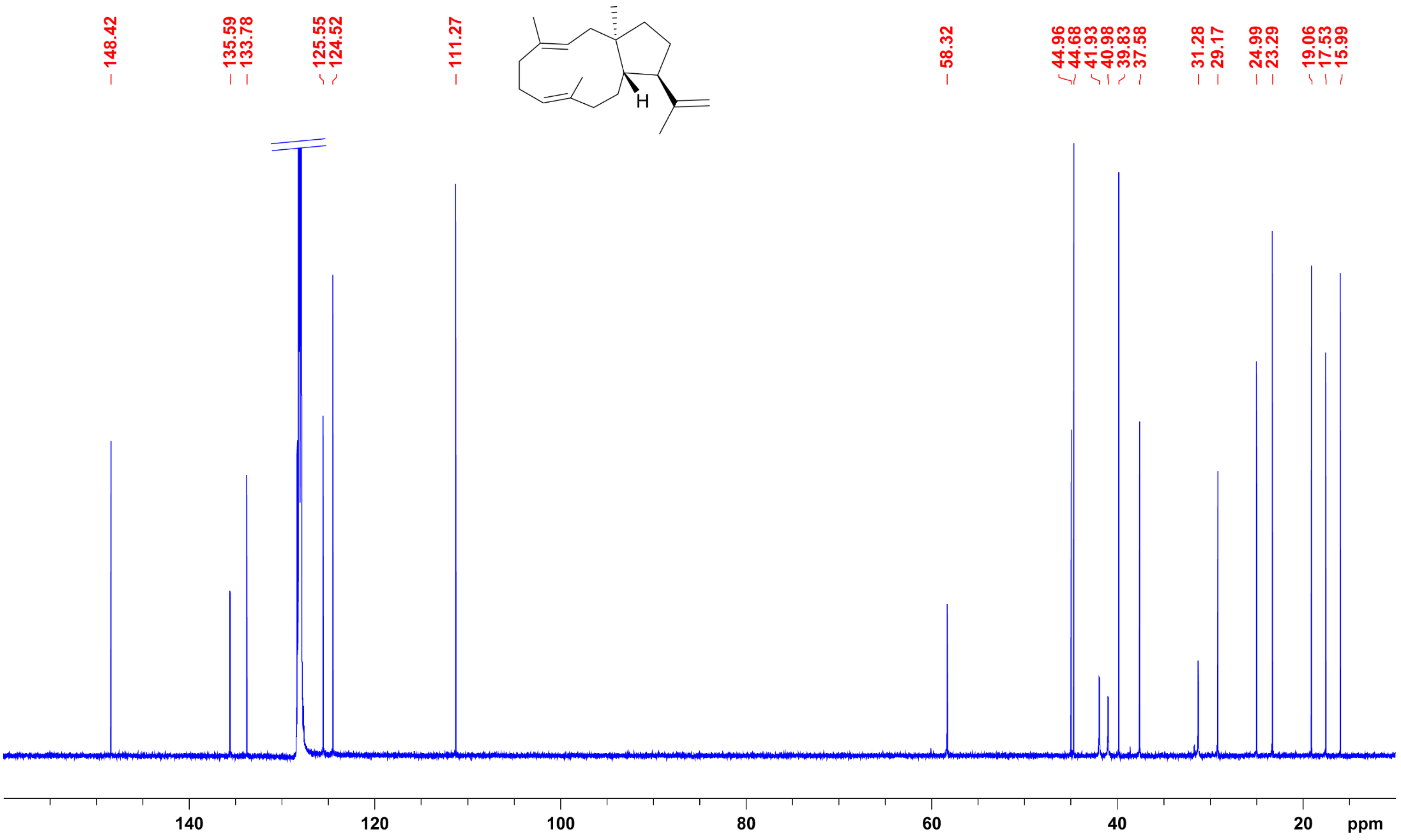

Figure S70. ^{13}C-NMR spectrum of **6** (176 MHz, C_6D_6).

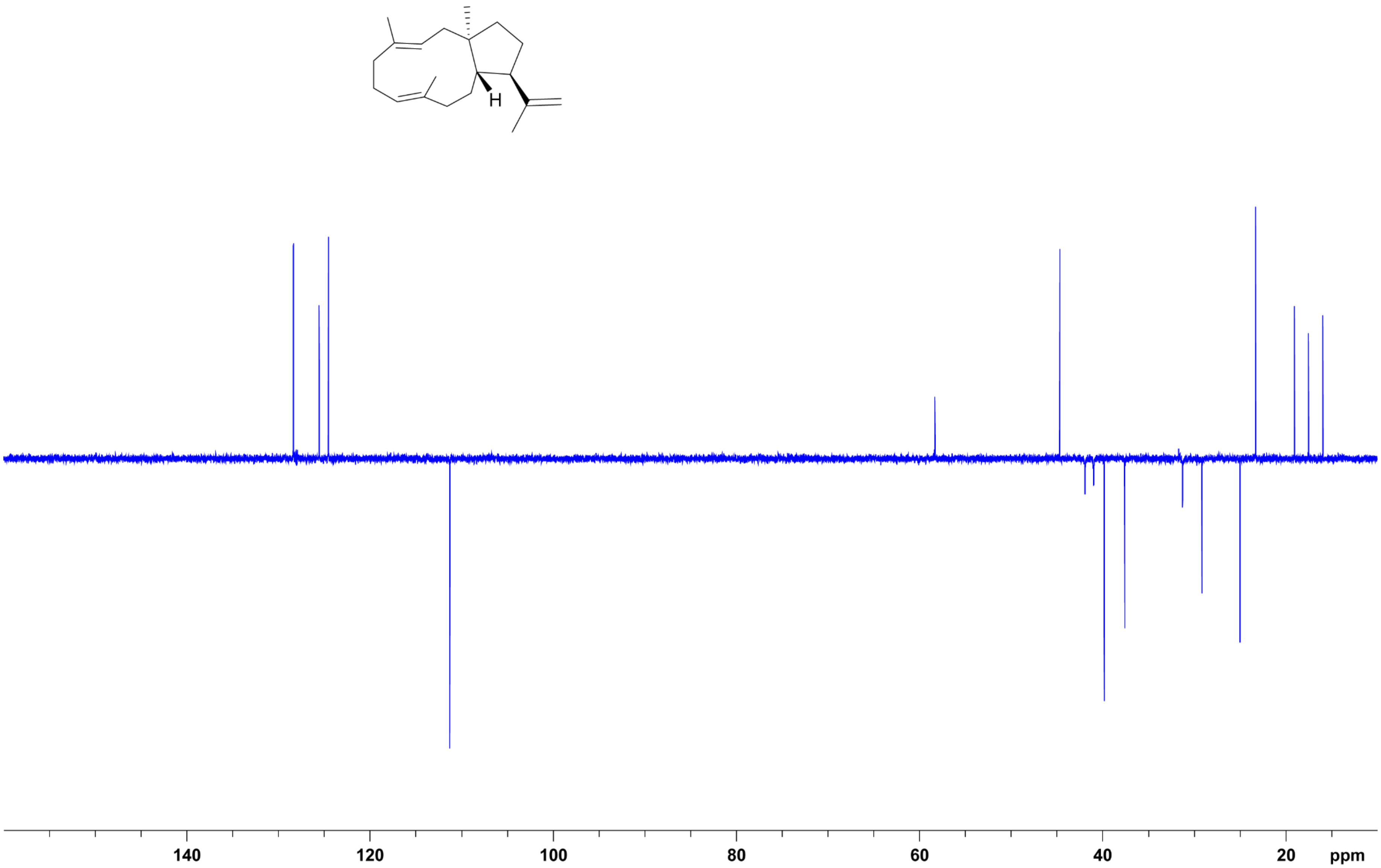

Figure S71. ^{13}C-DEPT135 spectrum of **6** (176 MHz, C_6D_6).

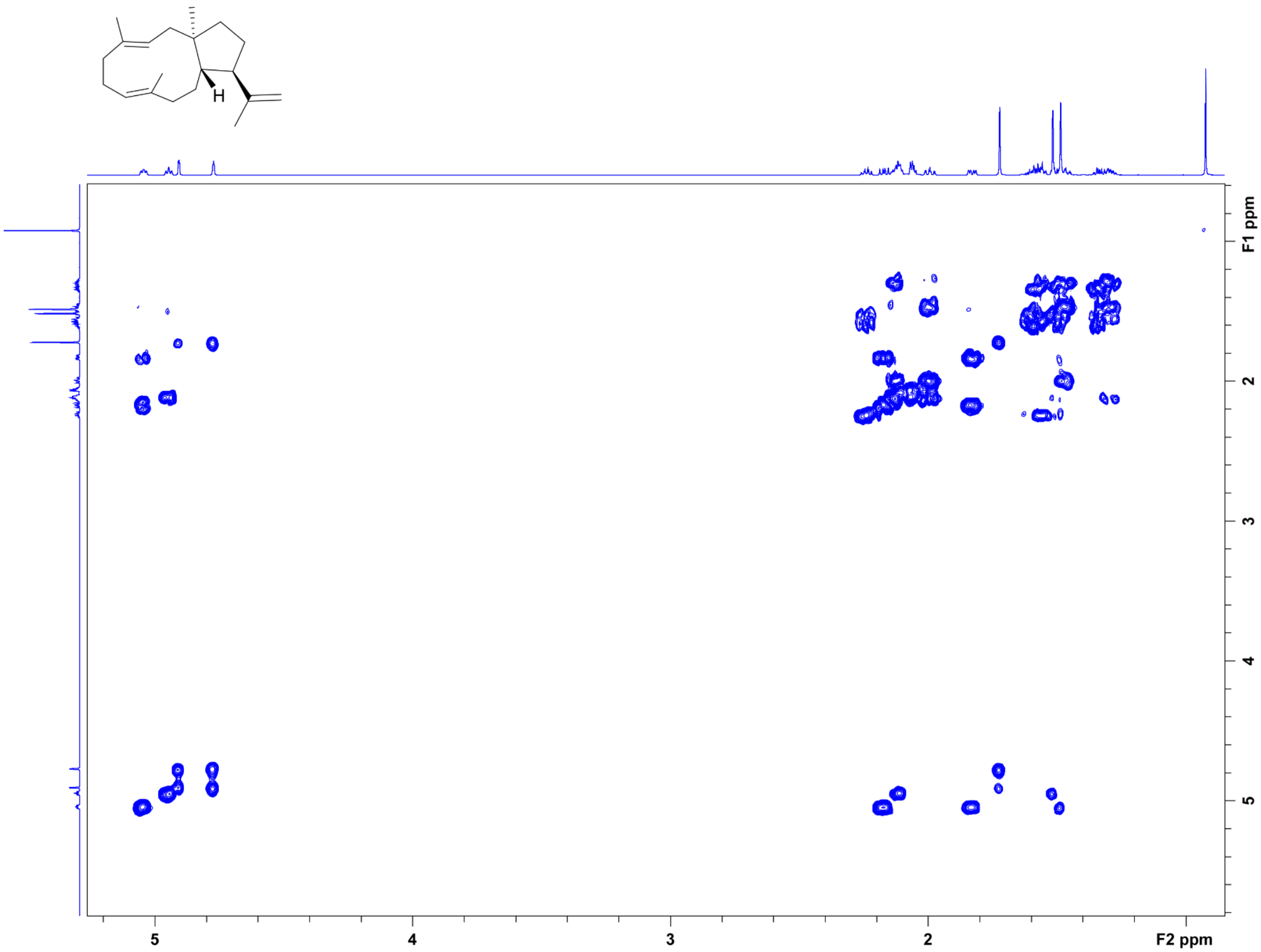

Figure S72. $^{1}H,^{1}H$-COSY spectrum (C_6D_6) of **6**.

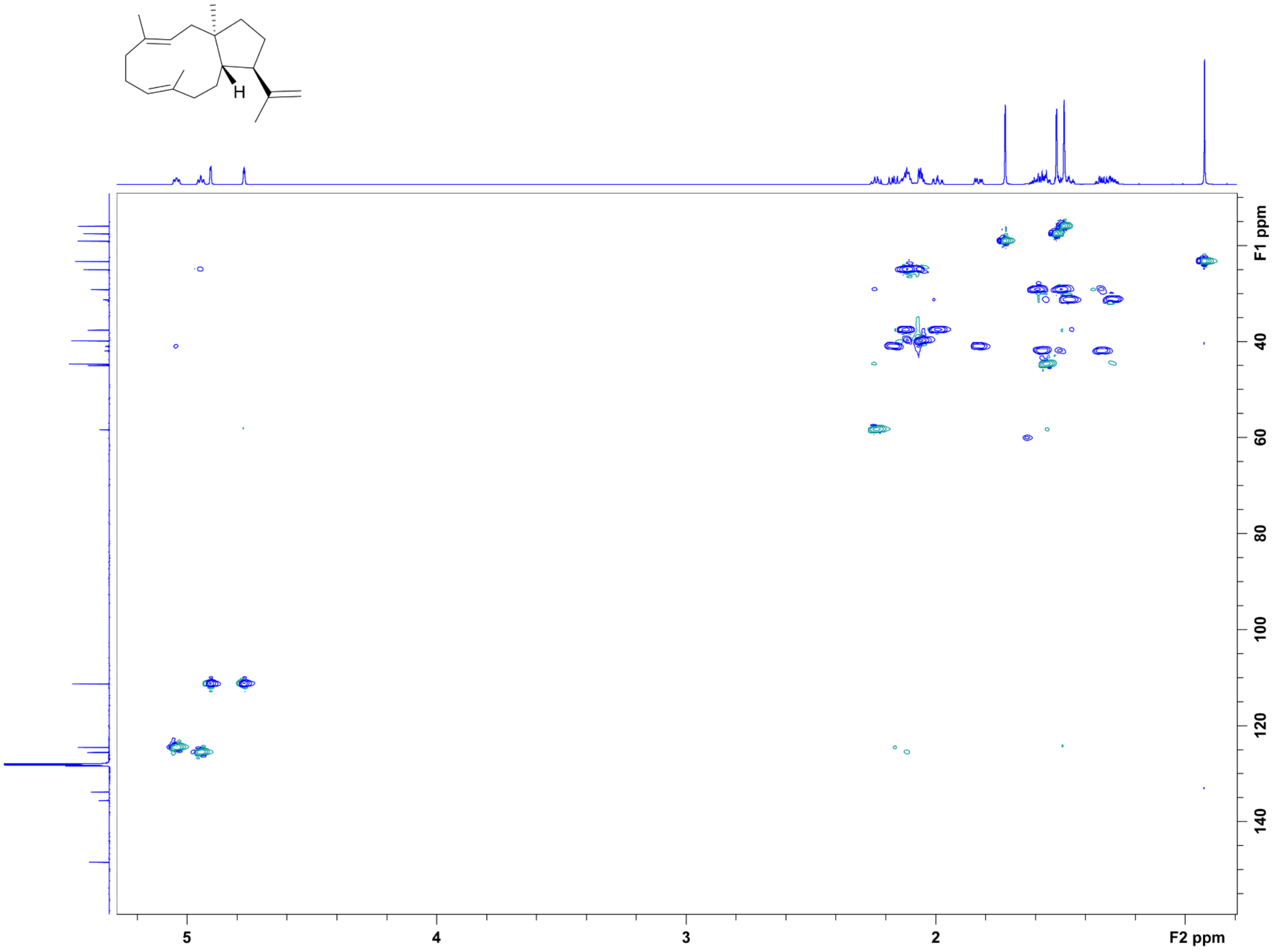

Figure S73. HSQC spectrum (C_6D_6) of **6**.

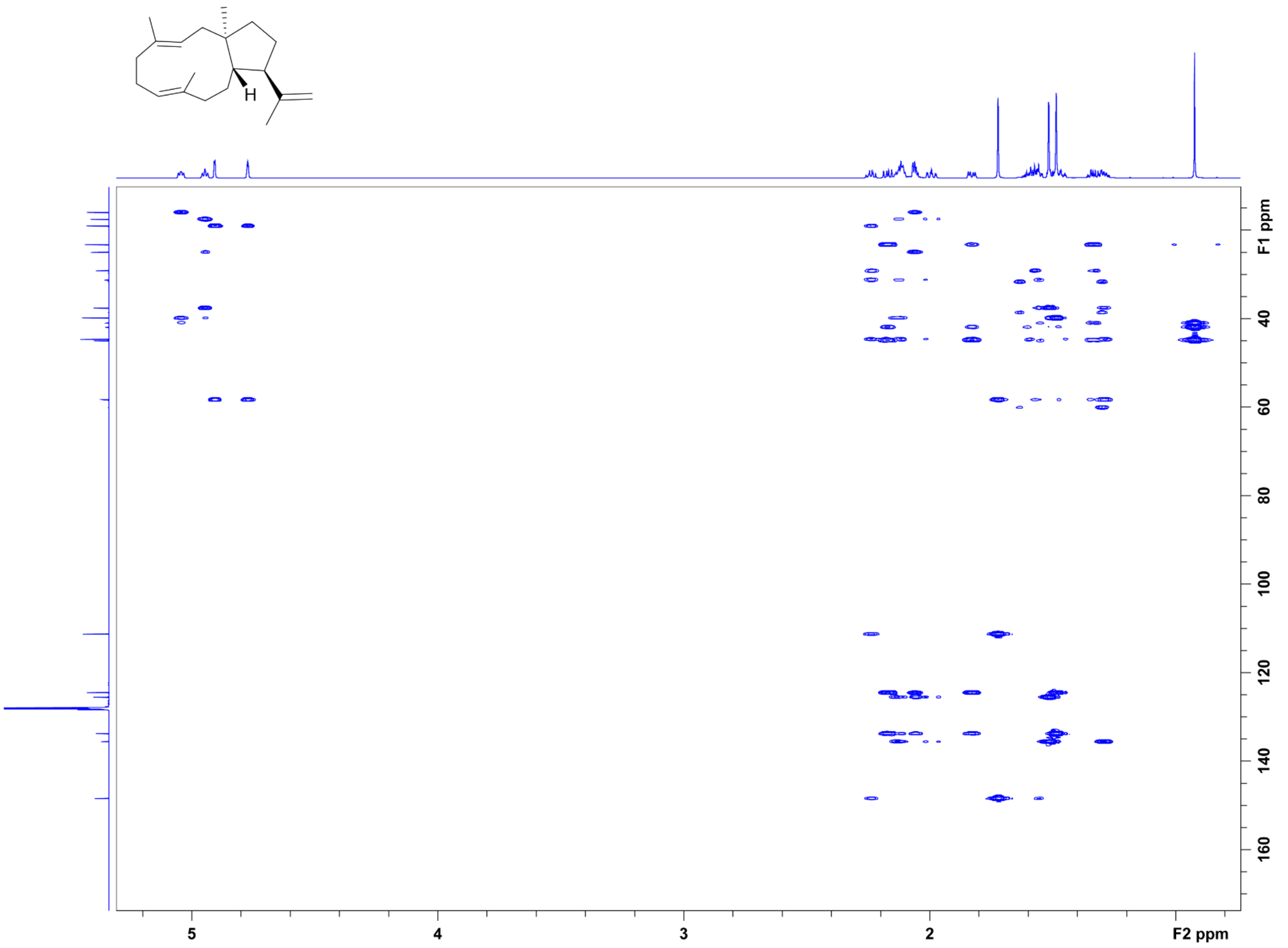

Figure S74. HMBC spectrum (C_6D_6) of **6**.

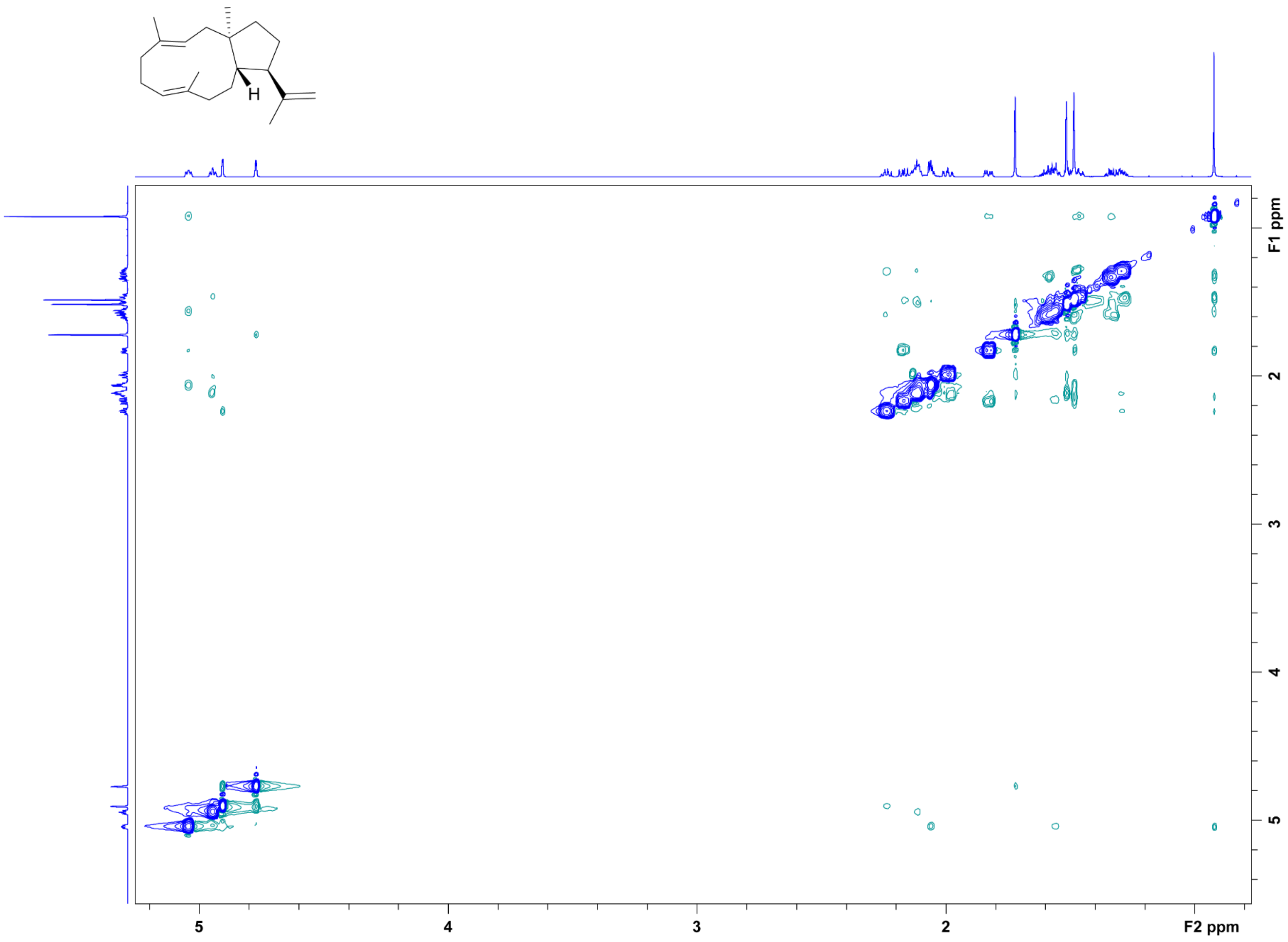

Figure S75. NOESY spectrum (C_6D_6) of **6**.

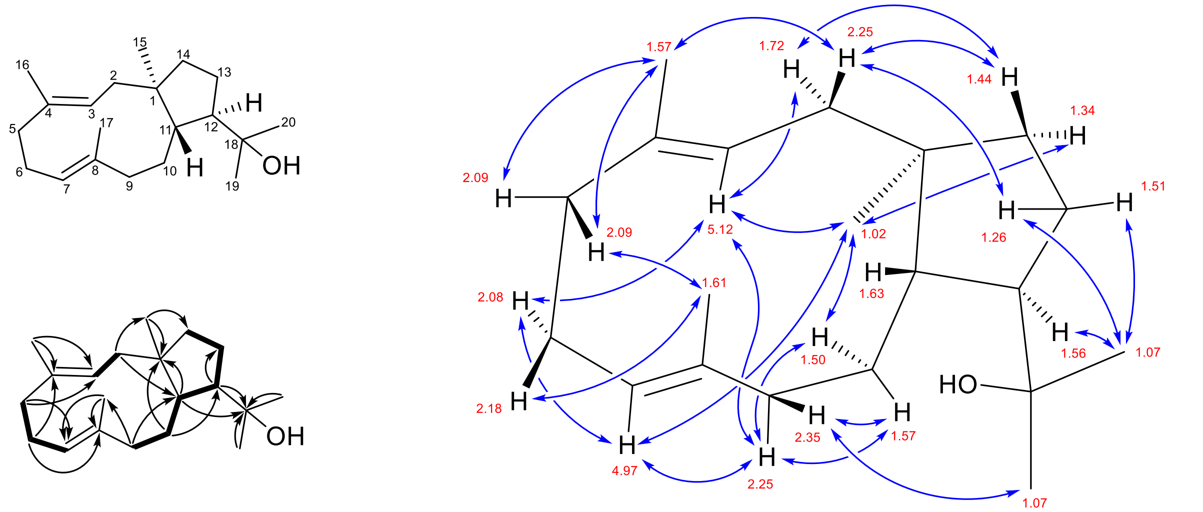

Figure S76. Structure elucidation of 12-*epi*-18-hydroxydolabela-3,7-diene (**7**). Bold: $^{1}H,^{1}H$-COSY, single headed arrows: key HMBC, and blue double headed arrows: NOESY correlations.

Table S13. NMR data of 12-*epi*-18-hydroxydolabela-3,7-diene (**7**) in C_6D_6 recorded at 298 K.

C[a]	type	^{13}C[b]	^{1}H[b]
1	C	46.91	–
2	CH_2	39.95	2.25 (m)
			1.72 (dd, *J* = 13.7, 5.8)
3	CH	125.17	5.12 (ddd, *J* = 9.2, 5.6, 1.4)
4	C	133.09	–
5	CH_2	40.07	2.09 (m, 2H)
6	CH_2	25.26	2.18 (m)
			2.08 (m)
7	CH	127.09	4.97 (m)
8	C	135.34	–
9	CH_2	39.04	2.35 (ddd, *J* = 11.8, 7.3, 4.3)
			2.25 (m)
10	CH_2	32.32	1.57 (m)
			1.50 (m)
11	CH	41.79	1.63 (dt, *J* = 7.3, 4.5)
12	CH	60.55	1.56 (m)
13	CH_2	26.85	1.51 (m)
			1.26 (m)
14	CH_2	41.92	1.44 (m)
			1.34 (ddd, *J* = 12.2, 9.3, 6.8)
15	CH_3	23.51	1.02 (s)
16	CH_3	16.57	1.57 (d, *J* = 1.3)
17	CH_3	16.74	1.61 (d, *J* = 1.4)
18	C	72.48	–
19	CH_3	31.30	1.07 (s)
20	CH_3	26.66	1.07 (s)

[a] Carbon numbering as shown in Figure S76. [b] Chemical shifts δ in ppm, multiplicity: s = singlet, d = doublet, t = triplet, m = multiplet, coupling constants *J* are given in Hertz.

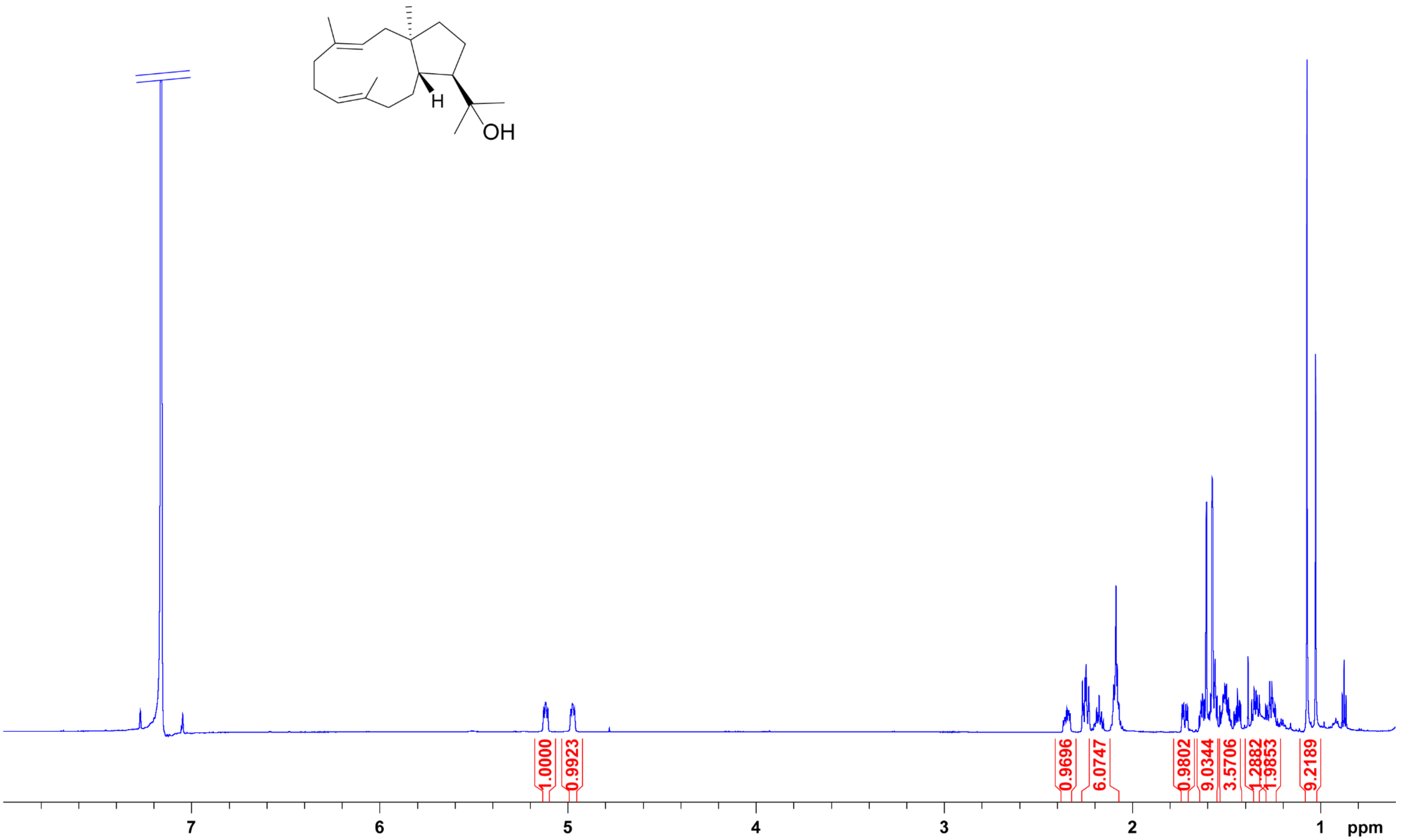

Figure S77. ^{1}H-NMR spectrum of **7** (700 MHz, C_6D_6).

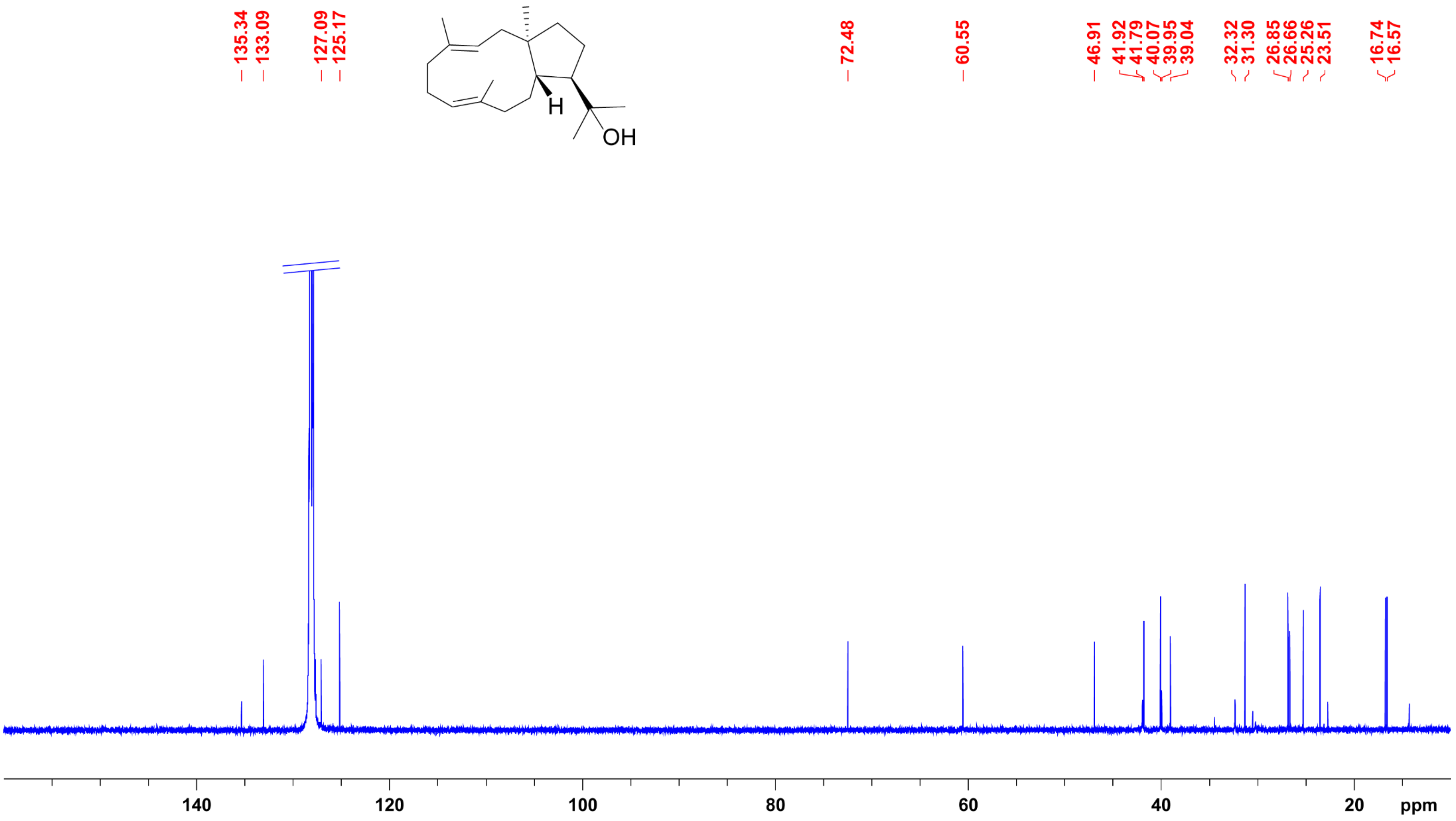

Figure S78. ^{13}C-NMR spectrum of **7** (176 MHz, C_6D_6).

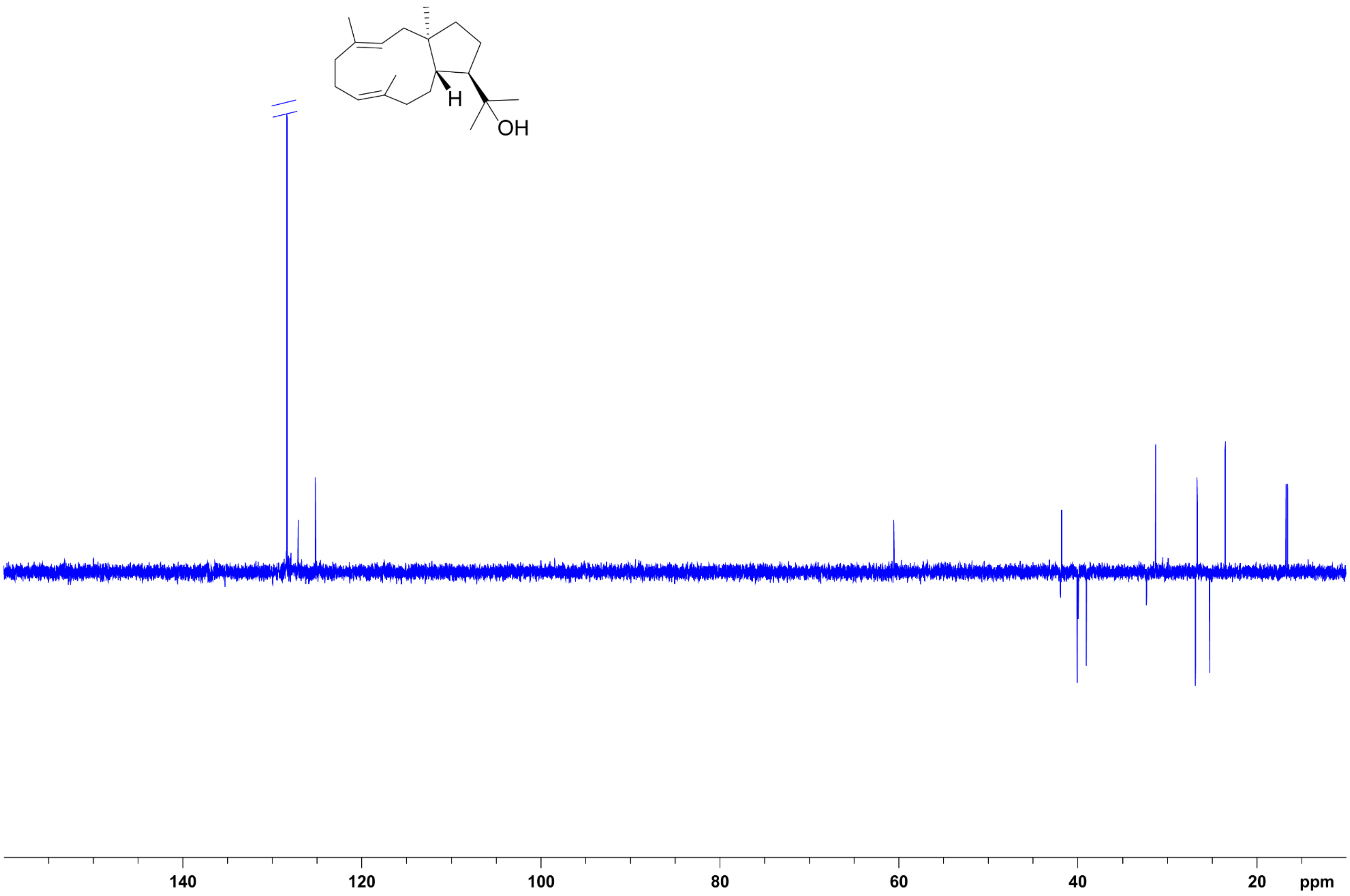

Figure S79. ^{13}C-DEPT135 spectrum of **7** (176 MHz, C_6D_6).

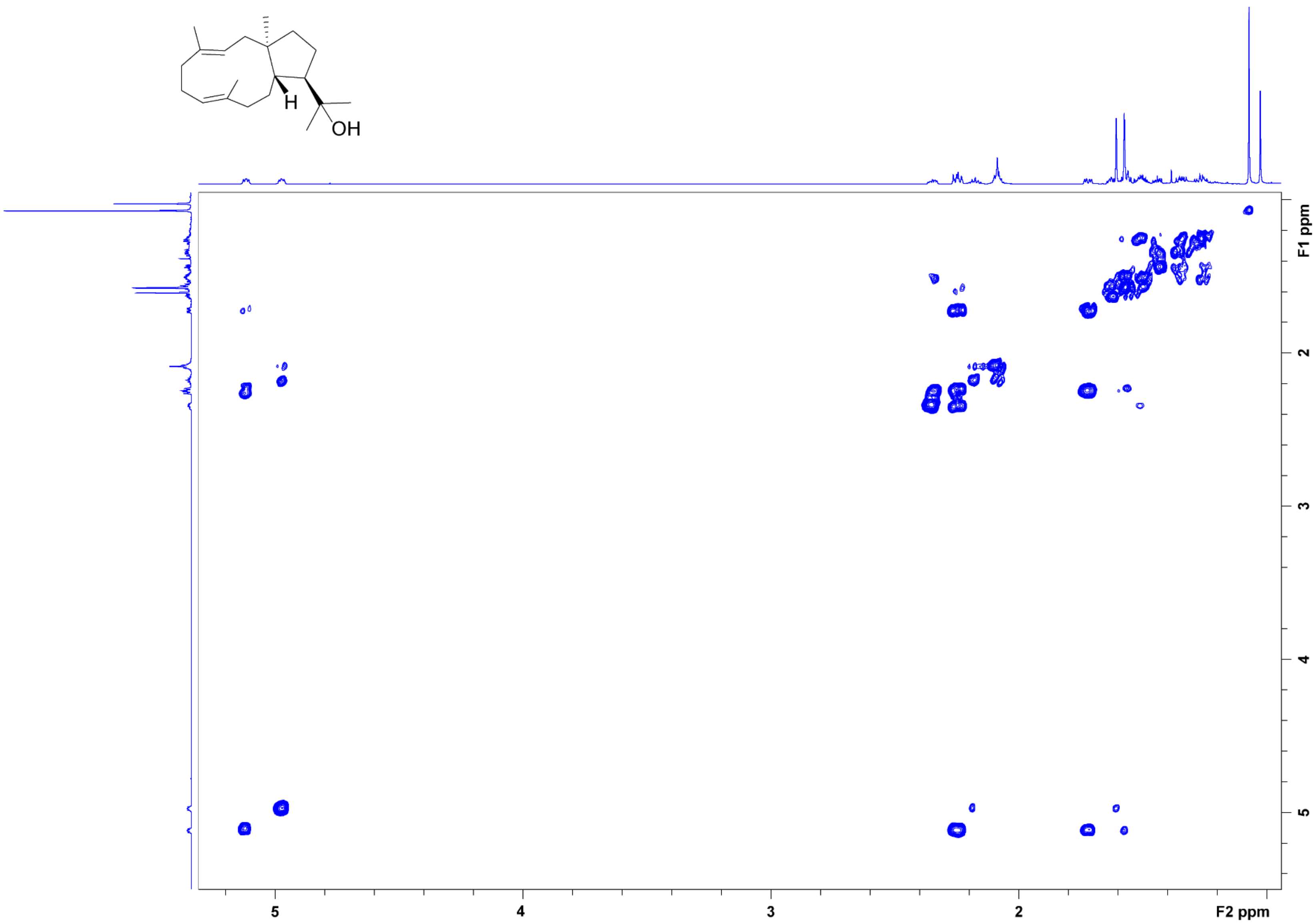

Figure S80. $^1H,^1H$-COSY spectrum (C_6D_6) of **7**.

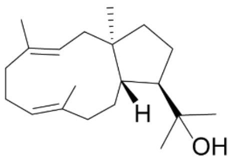

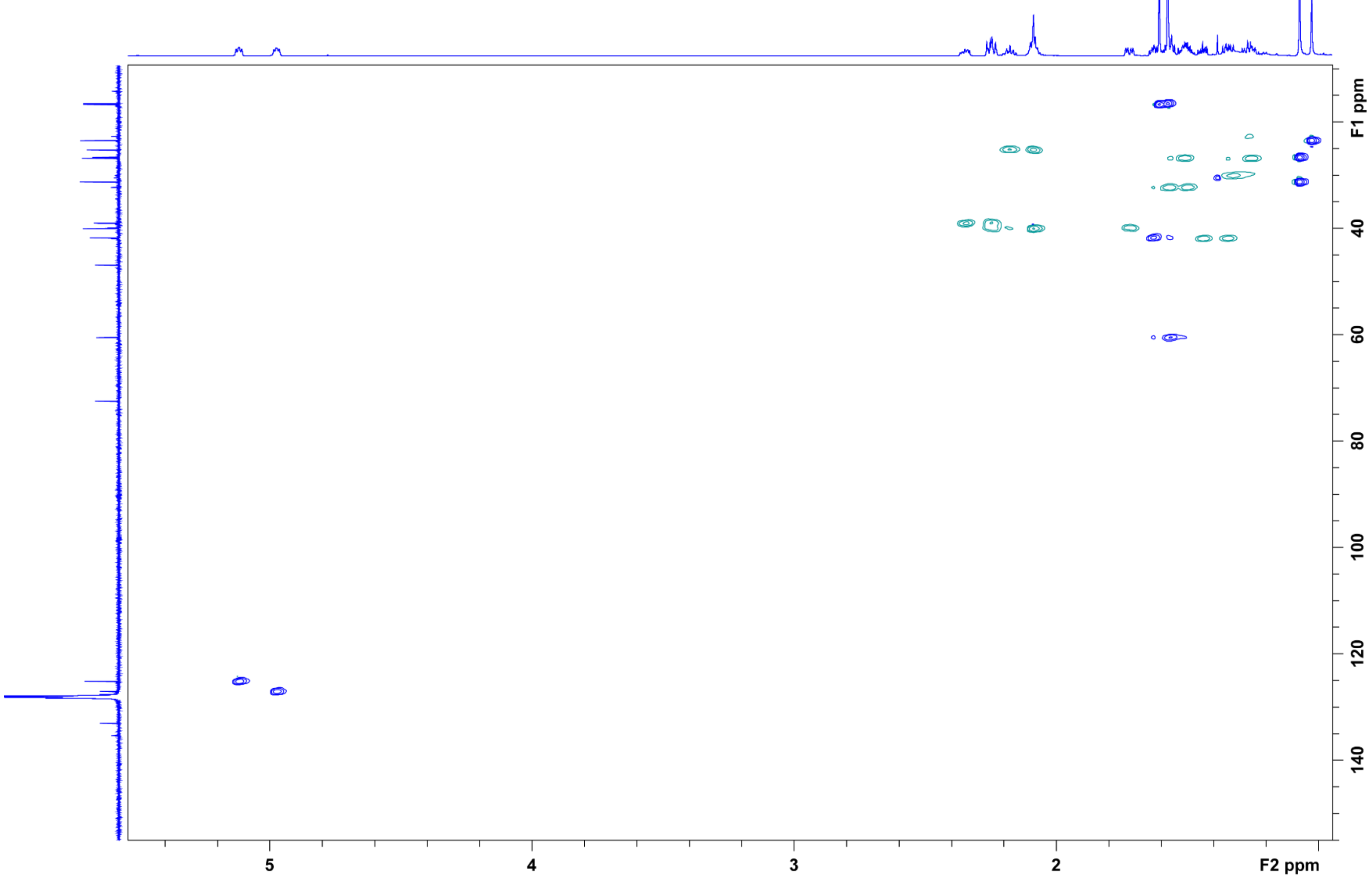

Figure S81. HSQC spectrum (C_6D_6) of **7**.

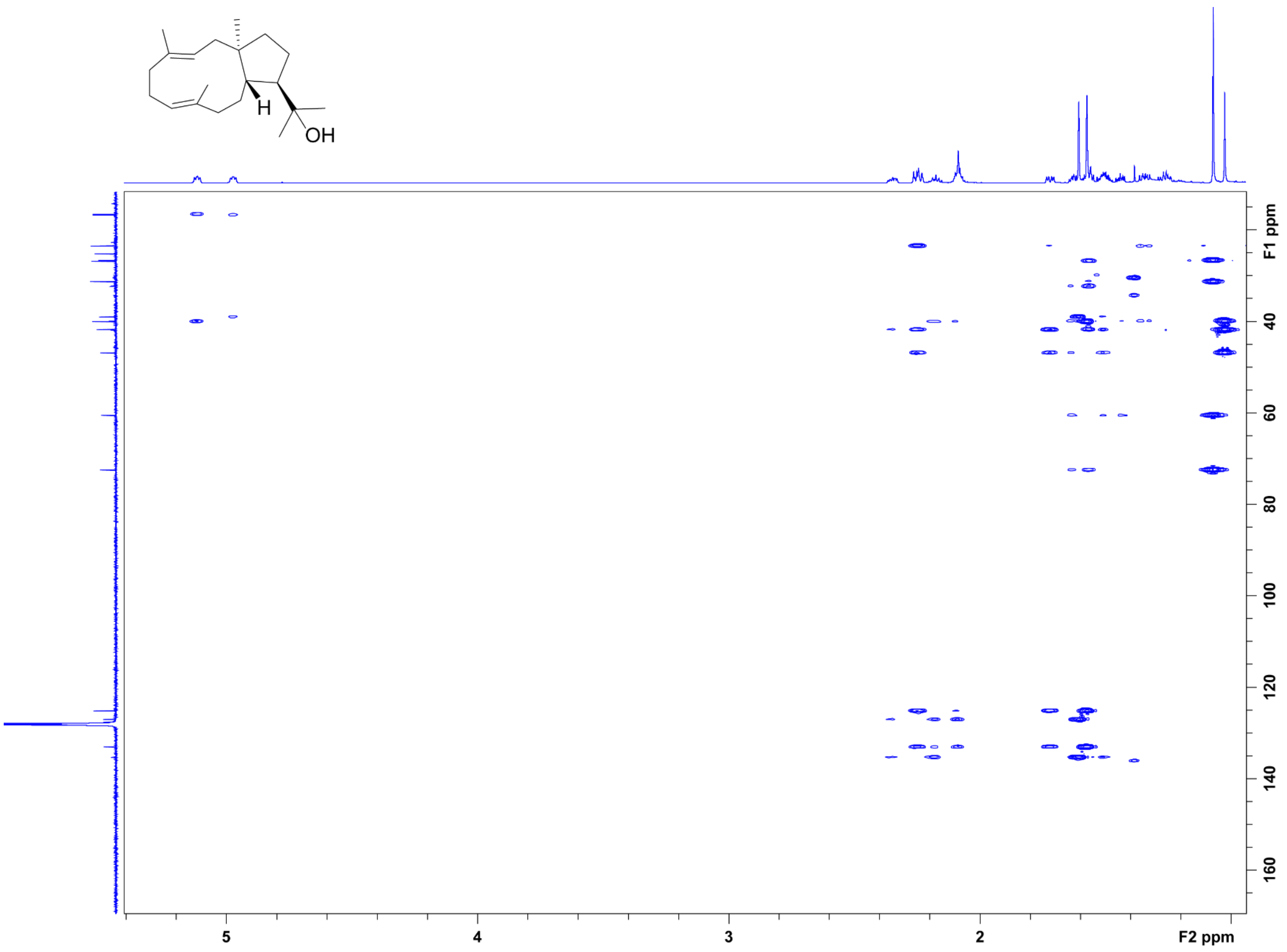

Figure S82. HMBC spectrum (C_6D_6) of **7**.

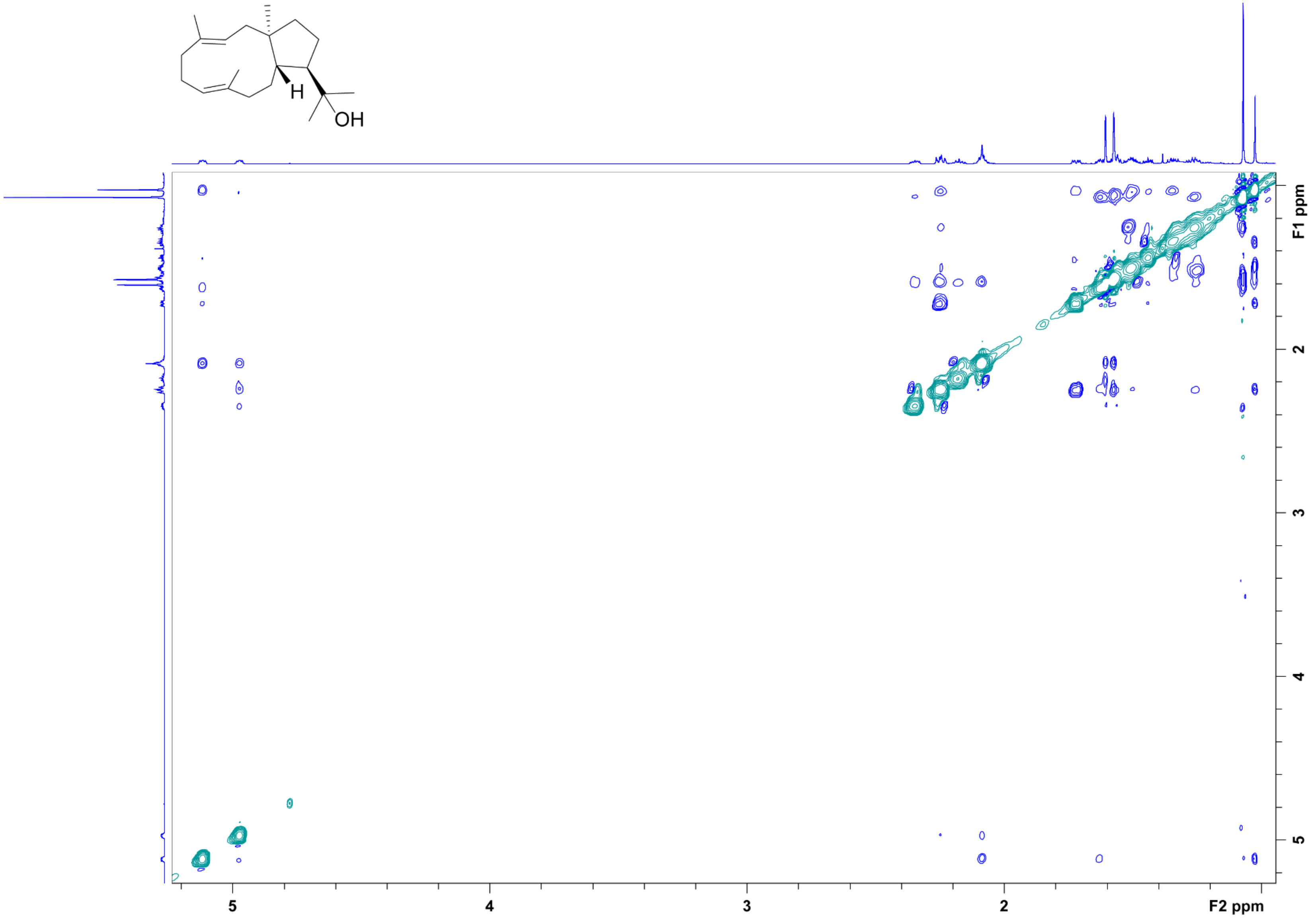

Figure S83. NOESY spectrum (C_6D_6) of **7**.

Table S14. NMR data of cembrene A (**8**) in C_6D_6 recorded at 298 K.

C[a]	type	^{13}C[b]	^{1}H[b]	^{13}C[b,c]
1	CH_2	33.02	2.16 (m)<br>2.01 (m)	33.0
2	CH	124.57	5.32 (ddq, *J* = 8.2, 6.8, 1.3)	124.6
3	C	134.91	–	135.0
4	CH_2	39.37	2.16 (m, 2H)	39.4
5	CH_2	25.35	2.27 (m)<br>2.15 (m)	25.4
6	CH	126.45	5.08 (ddq, *J* = 7.2, 4.1, 1.4)	126.5
7	C	133.47	–	133.5
8	CH_2	39.85	2.14 (m, 2H)	39.9
9	CH_2	24.22	2.17 (m, 2H)	24.2
10	CH	122.32	5.25 (tq, *J* = 6.6, 1.6)	122.3
11	C	133.85	–	133.9
12	CH_2	34.37	2.02 (m)<br>1.93 (m)	34.4
13	CH_2	28.59	1.79 (m)<br>1.41 (m)	28.6
14	CH	46.55	2.21 (m)	46.6
15	C	149.24	–	149.2
16	CH_3	19.37	1.63 (s)	19.4
17	CH_3	110.80	4.83 (br s)	110.9
18	CH_3	18.32	1.60 (s)	18.3
19	CH_3	15.40	1.54 (s)	15.4
20	CH_3	15.64	1.55 (s)	15.7

[a] Carbon numbering indicates the origin of each carbon from GGPP by same number. [b] Chemical shifts δ in ppm, multiplicity: s = singlet, d = doublet, t = triplet, q = quartet, m = multiplet, br = broad, coupling constants *J* are given in Hertz. [c] Literature data from reference [32] for comparison.

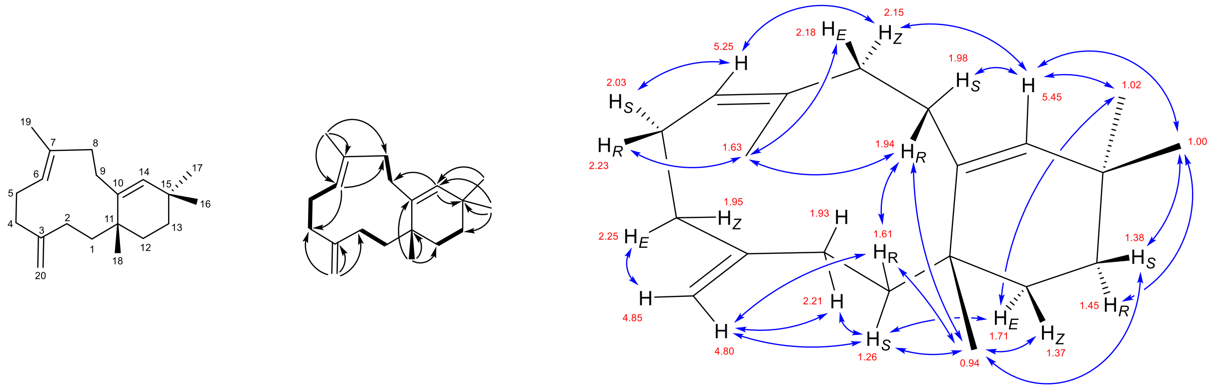

Figure S84. Structure elucidation of chryseojoostene E (**5**). Bold: $^{1}H,^{1}H$-COSY correlations, single headed arrows: HMBC correlations, and blue double headed arrows: NOESY correlations. Carbon numbering follows GGPP numbering to indicate the origin of each carbon. The hydrogen labels H_R, H_S, H_E and H_Z indicate the results from stereoselective deuteration experiments (Figures S94 and S95).

Table S15. NMR data of chryseojoostene E (**5**) in C_6D_6 recorded at 298 K.

C[a]	type	^{13}C[b]	^{1}H[b]
1	CH_2	38.36	1.61 (m)
			1.26 (m)
2	CH_2	33.89	2.21 (m)
			1.94 (m)
3	C	153.22	–
4	CH_2	36.80	2.25 (m)
			1.96 (m)
5	CH_2	29.72	2.23 (m)
			2.03 (dq, J = 10.3, 3.2)
6	CH	126.22	5.25 (tm, J = 7.8)
7	C	135.11	–
8	CH_2	39.31	2.18 (m)
			2.15 (m)
9	CH_2	28.21	1.98 (m)
			1.94 (m)
10	C	140.61	–
11	C	37.79	–
12	CH_2	33.18	1.71 (ddt, J = 12.9, 7.9, 3.6)
			1.38 (m)
13	CH_2	35.10	1.45 (ddd, J = 14.4, 7.9, 3.6)
			1.39 (m)
14	CH	135.77	5.45 (d, J = 1.6)
15	C	31.98	–
16	CH_3	30.20	1.02 (s)
17	CH_3	30.04	1.00 (s)
18	CH_3	29.85	0.94 (s)
19	CH_3	18.12	1.64 (d, J = 1.5)
20	CH_2	110.02	4.85 (br s)
			4.80 (br s)

[a] Carbon numbering as shown in Figure S84 indicates the origin of each carbon from GGPP by same number. [b] Chemical shifts δ in ppm, multiplicity: s = singlet, d = doublet, t = triplet, q = quartet, m = multiplet, br = broad, coupling constants J are given in Hertz.

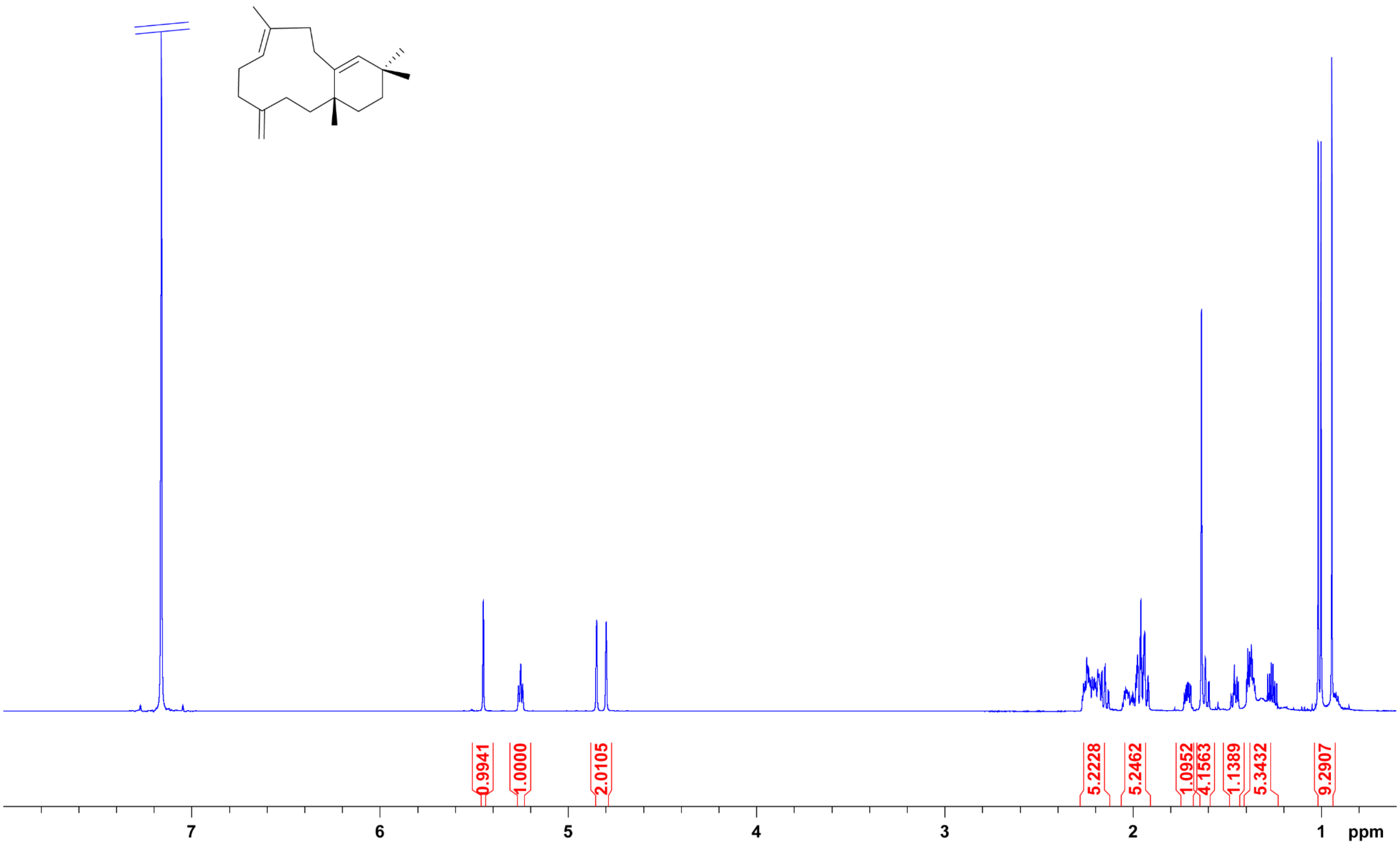

Figure S85. ^{1}H-NMR spectrum of **5** (700 MHz, C_6D_6).

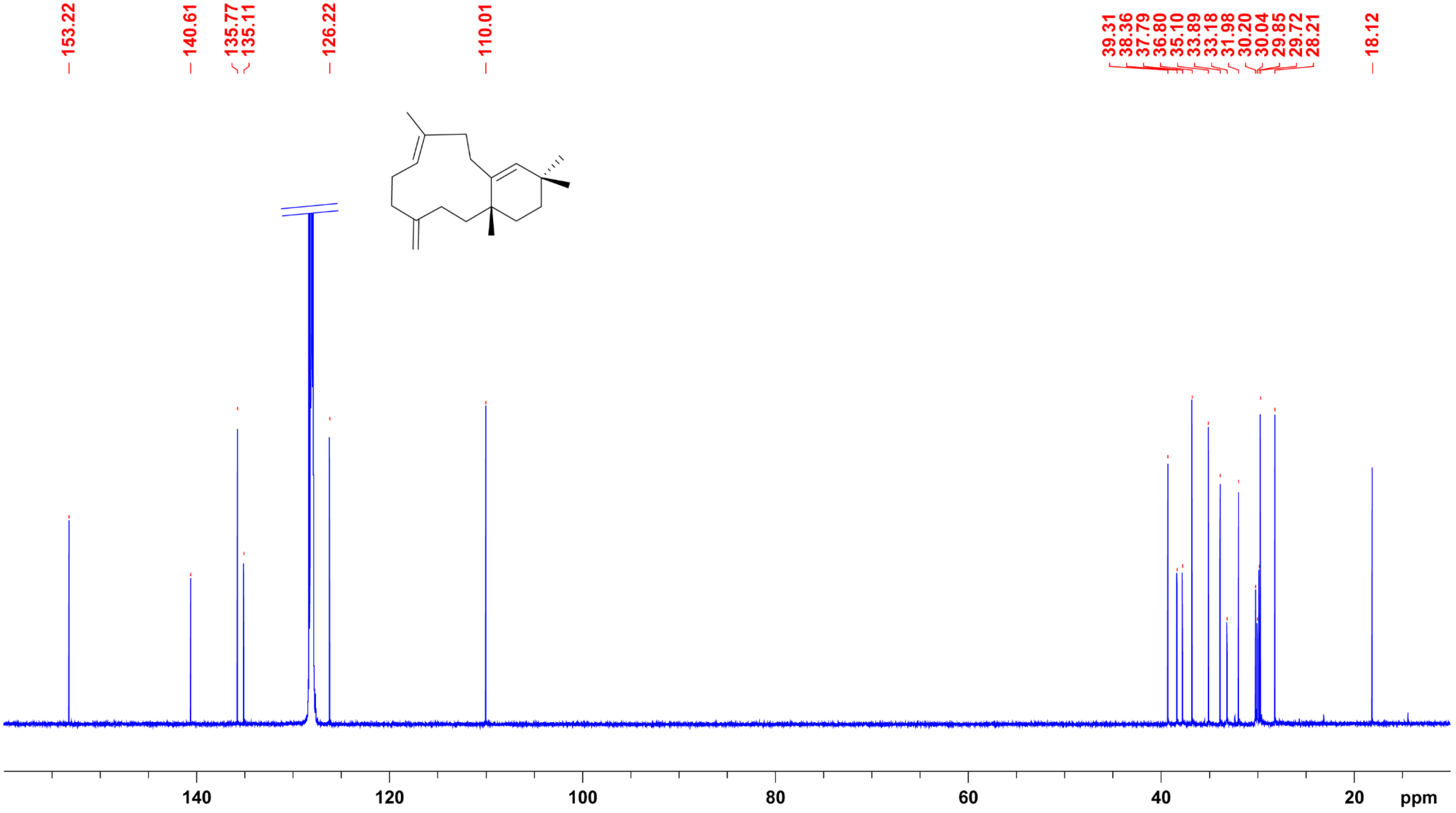

Figure S86. ^{13}C-NMR spectrum of **5** (176 MHz, C_6D_6).

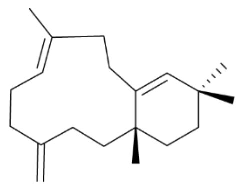

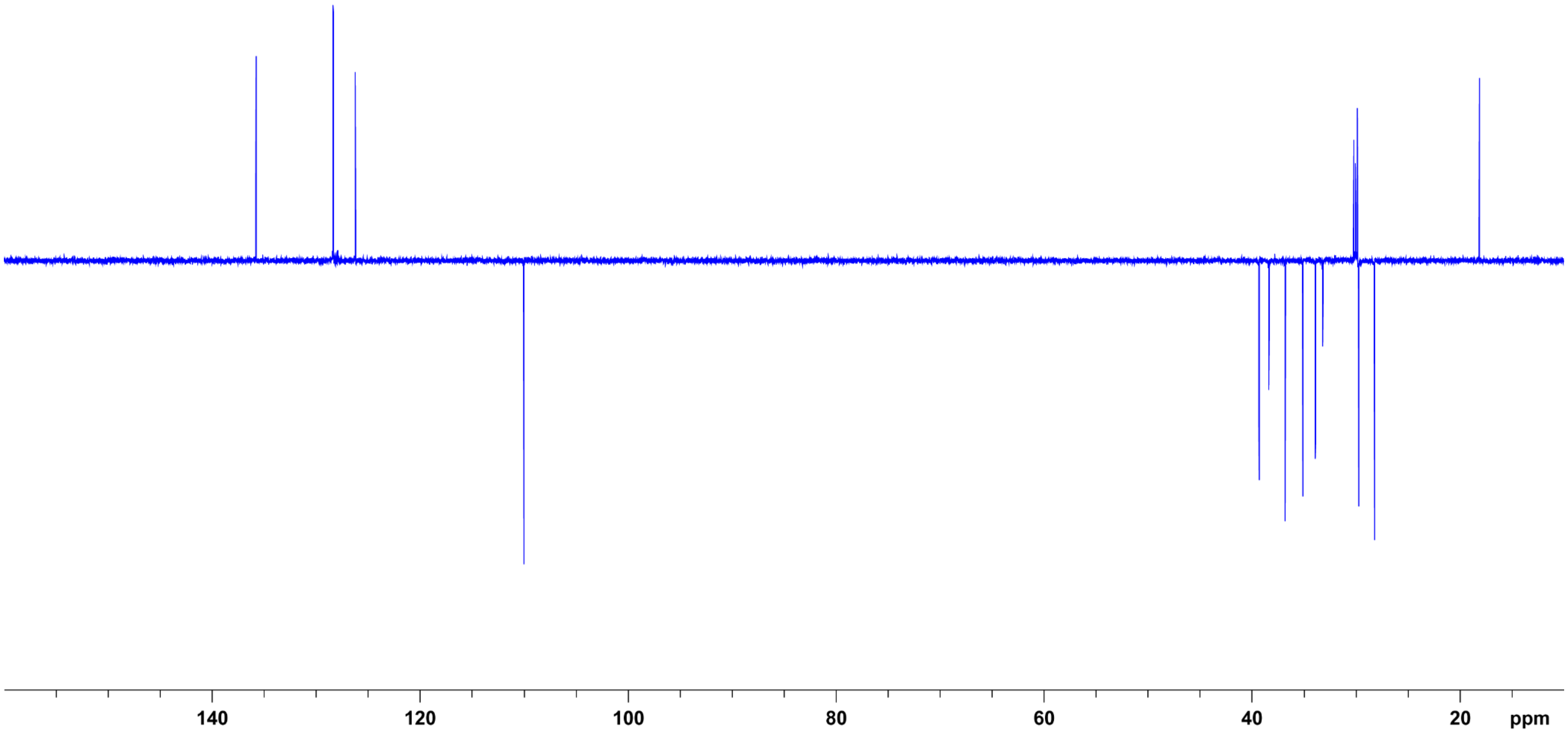

Figure S87. ^{13}C-DEPT135 spectrum of **5** (176 MHz, C_6D_6).

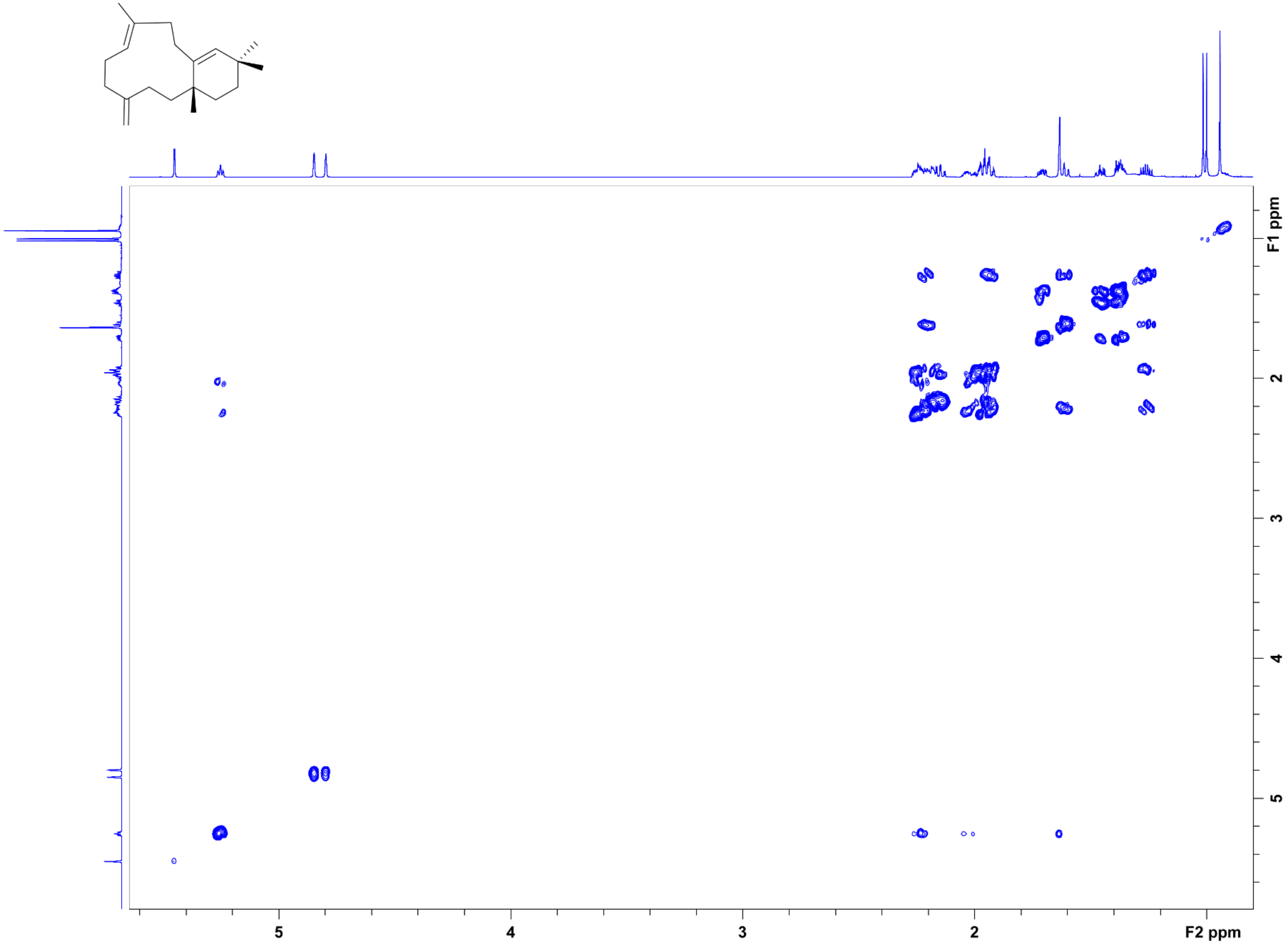

Figure S88. $^{1}H,^{1}H$-COSY spectrum (C_6D_6) of **5**.

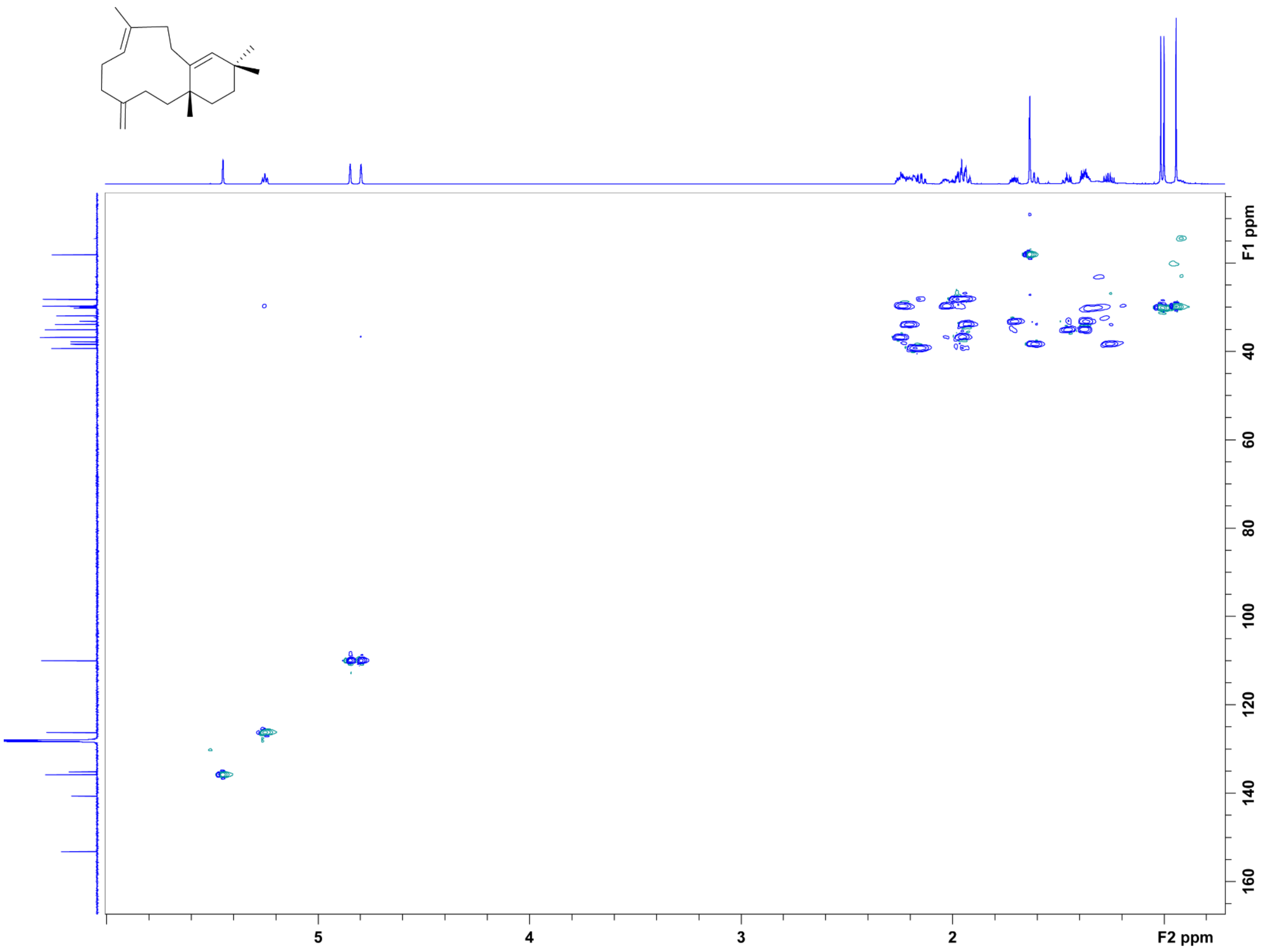

Figure S89. HSQC spectrum (C_6D_6) of **5**.

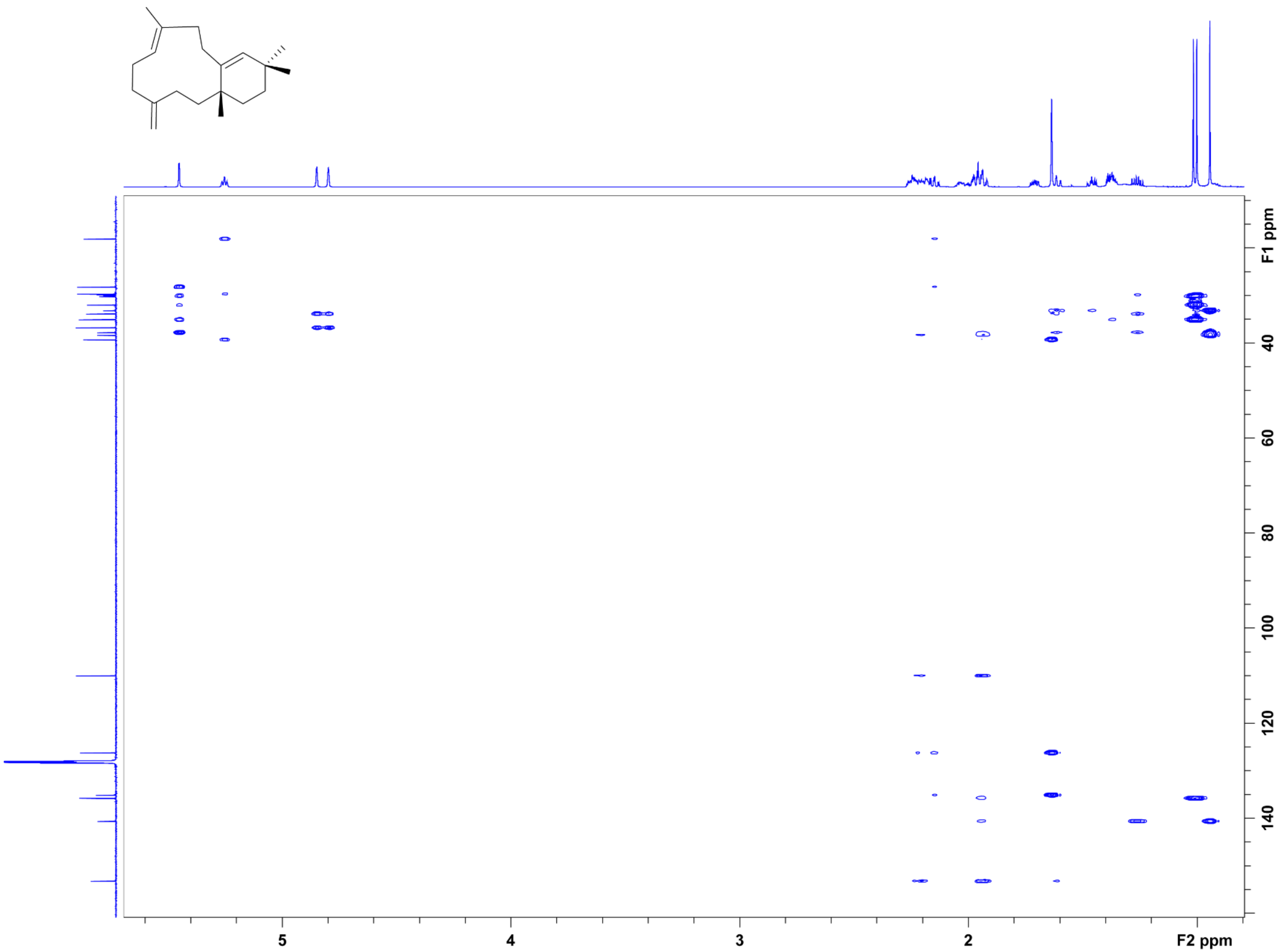

Figure S90. HMBC spectrum (C_6D_6) of **5**.

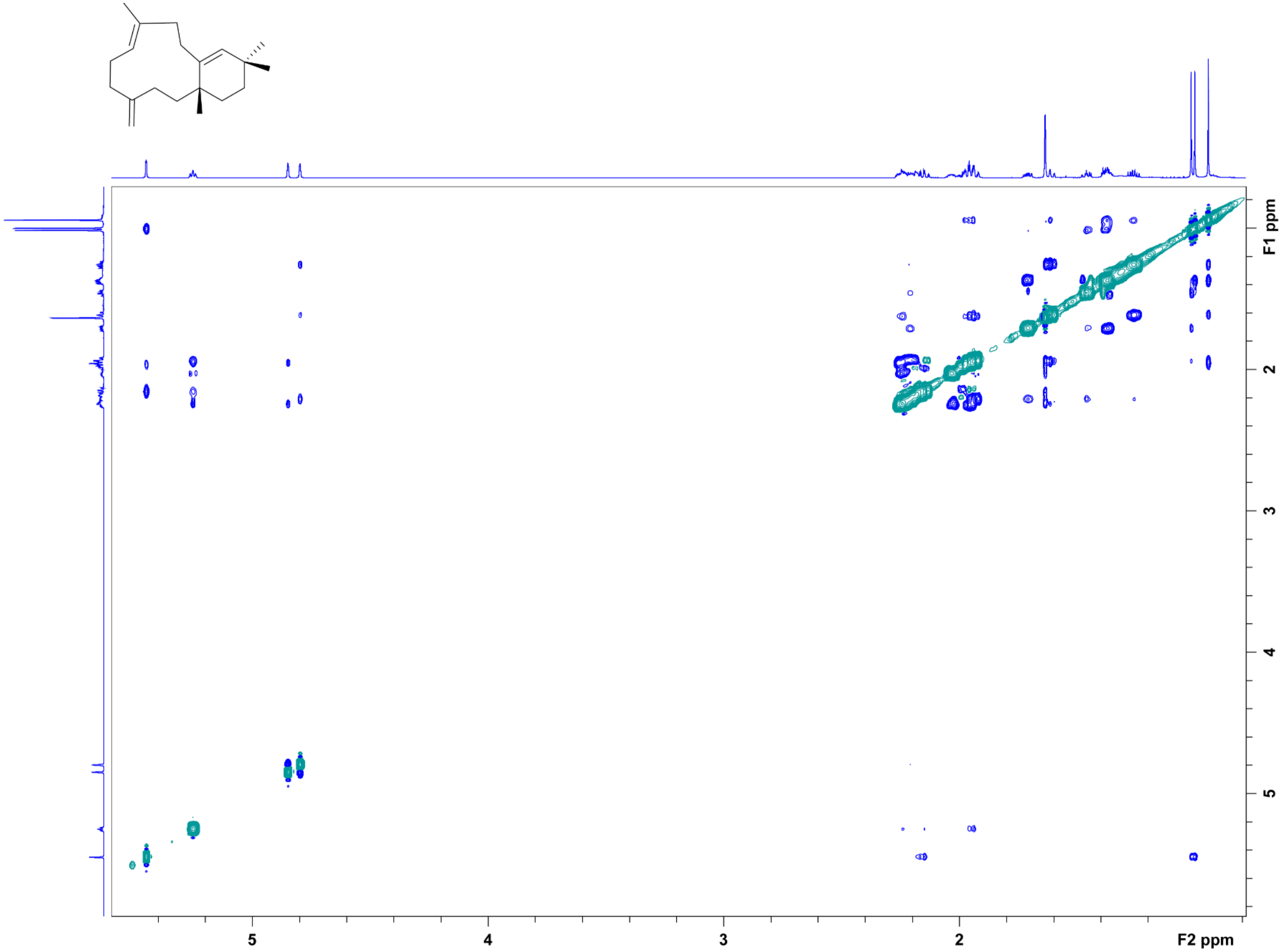

Figure S91. NOESY spectrum (C_6D_6) of **5**.

A)

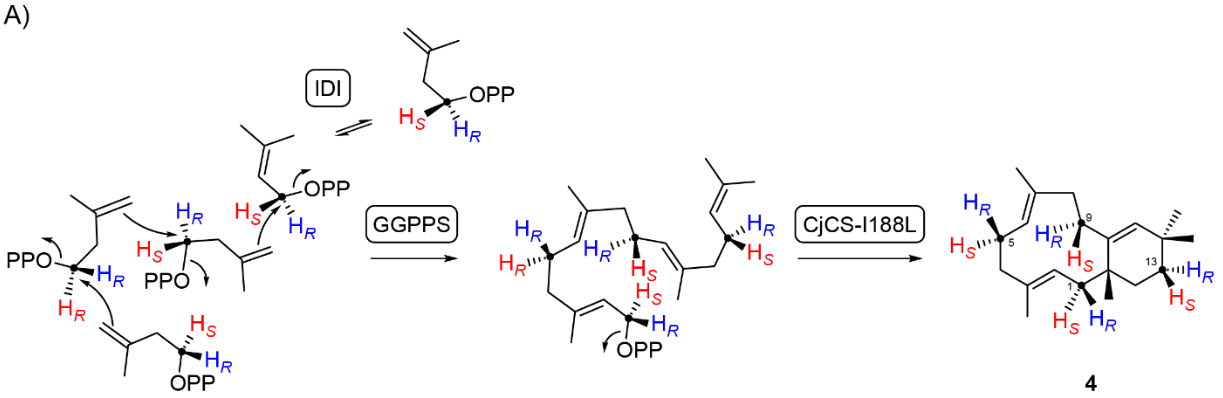

B)

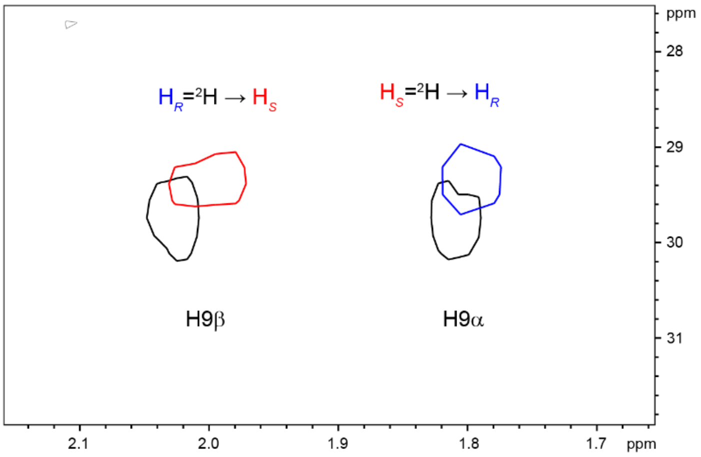

Figure S92. The absolute configuration of **4**. A) Conversion of (*R*)- and (*S*)-(1-^{13}C,1-^{2}H)IPP by IDI, GGPPS and CjCS into **4**. Three overlaid HSQC spectra at position C9 for unlabelled **4** shown in black, for labelled **4** obtained with GGPPS and CjCS-I188L from (*S*)-(1-^{13}C,1-^{2}H)IPP shown in blue, and for labelled **4** obtained with GGPPS and CjCS-I188L from (*R*)-(1-^{13}C,1-^{2}H)IPP shown in red. Positions C1, C5 and C13 were unsuitable for absolute configuration determination, because the diastereotopic hydrogens in these positions are isochronous.

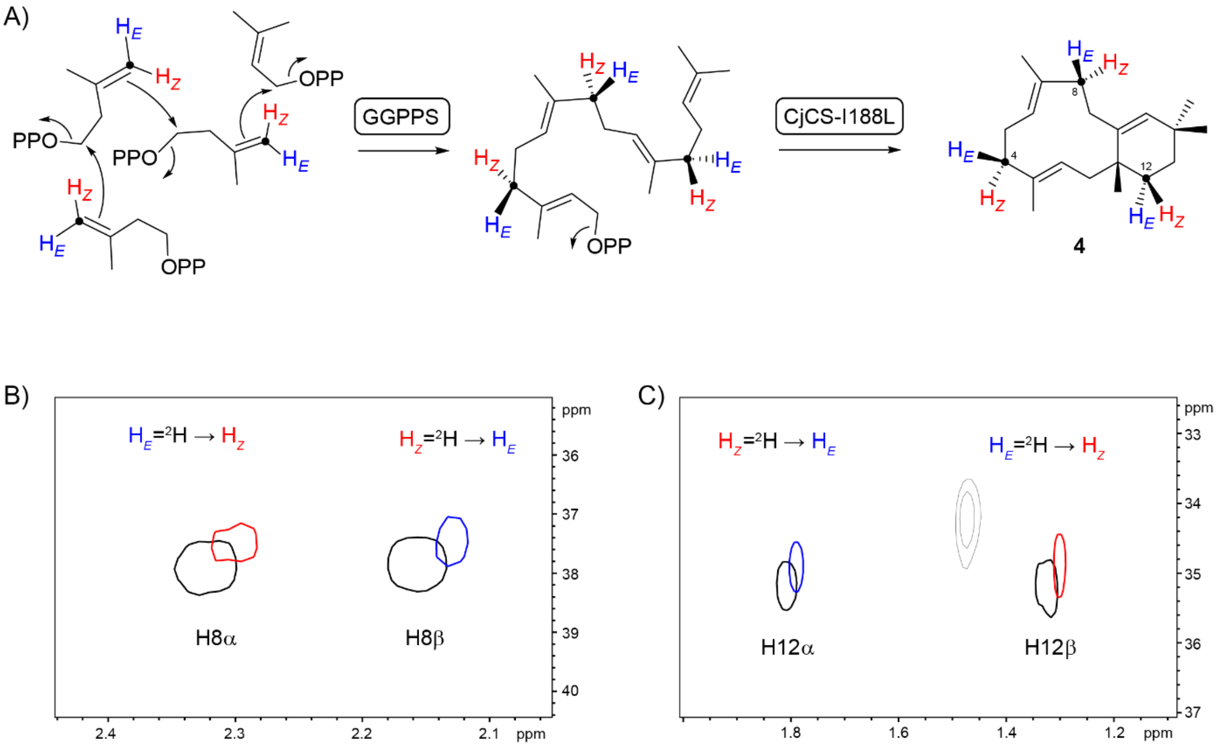

Figure S93. The absolute configuration of **4**. A) Conversion of DMAPP and (*E*)- and (*Z*)-(4-^{13}C,4-^{2}H)IPP by IDI, GGPPS and CjCS into **4**. Three overlaid HSQC spectra at position B) C8 and C) C12 for unlabelled **4** shown in black, for labelled **4** obtained with GGPPS and CjCS-I188L from (*Z*)-(4-^{13}C,4-^{2}H)IPP shown in blue, and for labelled **4** obtained with GGPPS and CjCS-I188L from (*E*)-(4-^{13}C,4-^{2}H)IPP shown in red. Position C4 was unsuitable for absolute configuration determination due to signal overlap with the signals of other enzyme products.

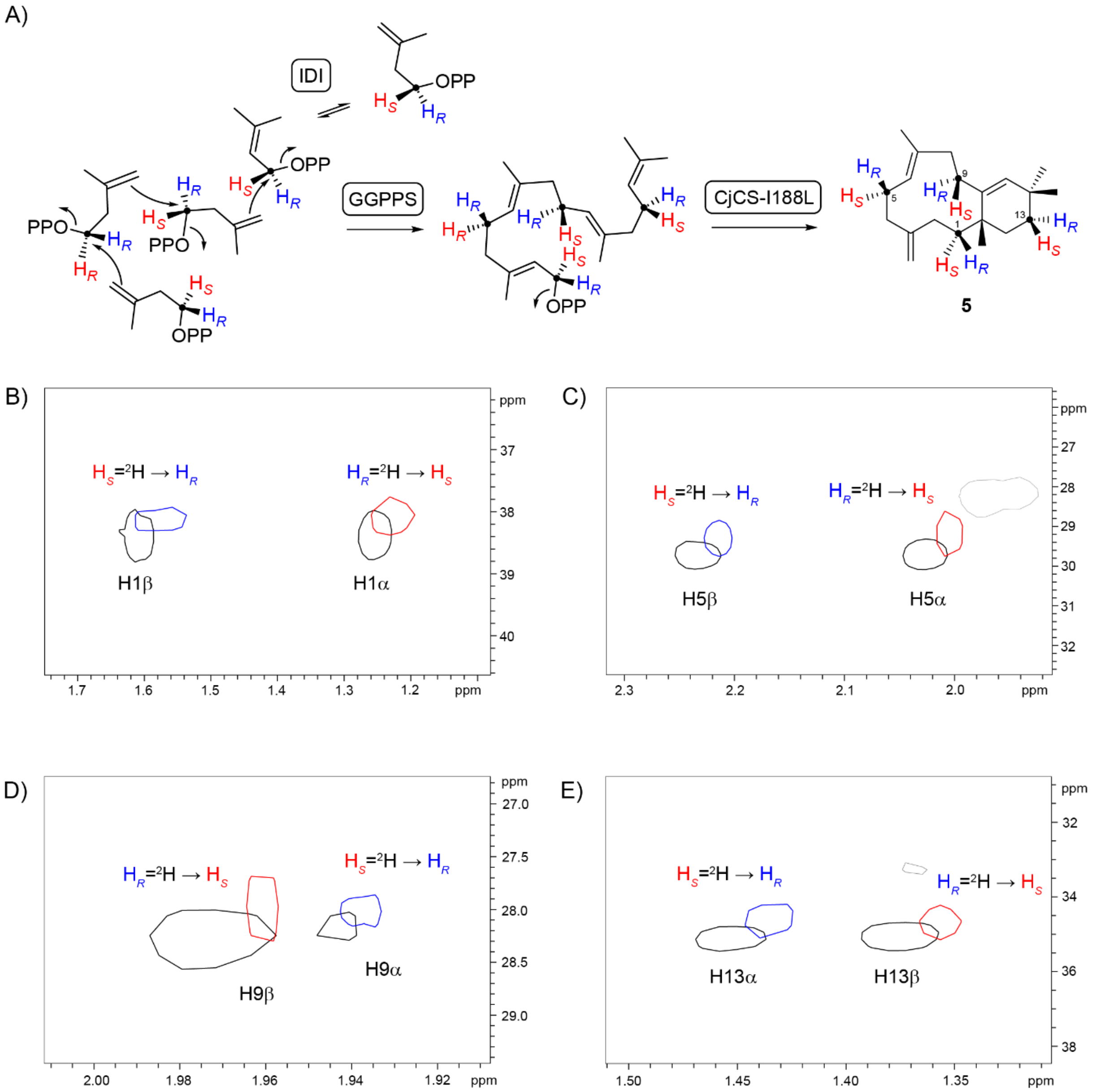

Figure S94. The absolute configuration of **5**. A) Conversion of (*R*)- and (*S*)-(1-^{13}C,1-^{2}H)IPP by IDI, GGPPS and CjCS-I188L into **5**. Three overlaid HSQC spectra at positions B) C1, C) C5, D) C9, and E) C13 for unlabelled **5** shown in black, for labelled **5** obtained with GGPPS and CjCS-I188L from (*S*)-(1-^{13}C,1-^{2}H)IPP shown in blue, and for labelled **5** obtained with GGPPS and CjCS-I188L from (*R*)-(1-^{13}C,1-^{2}H)IPP shown in red. Experiments C), D) and E) indicate the absolute configuration of **5**, while experiment B) confirms the inversion of configuration at C1 during terpene cyclisation.

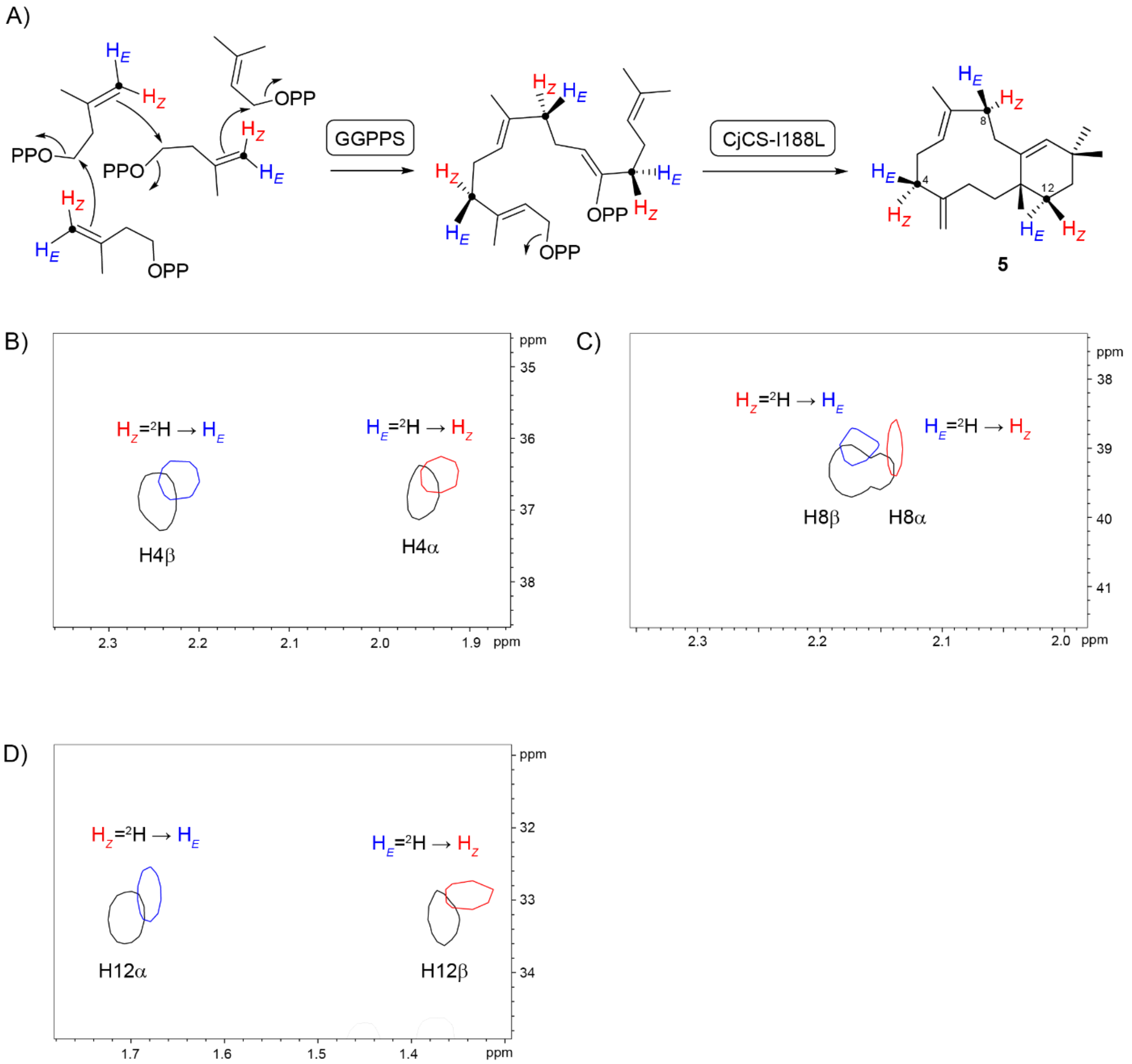

Figure S95. The absolute configuration of **5**. A) Conversion of DMAPP and (*E*)- and (*Z*)-(4-^{13}C,4-^{2}H)IPP by IDI, GGPPS and CjCS into **5**. Three overlaid HSQC spectra at positions B) C4, C) C8, and D) C12 for unlabelled **5** shown in black, for labelled **5** obtained with GGPPS and CjCS-I188L from (*Z*)-(4-^{13}C,4-^{2}H)IPP shown in blue, and for labelled **5** obtained with GGPPS and CjCS-I188L from (*E*)-(4-^{13}C,4-^{2}H)IPP shown in red. Experiments B), C) and D) indicate the absolute configuration of **5**.

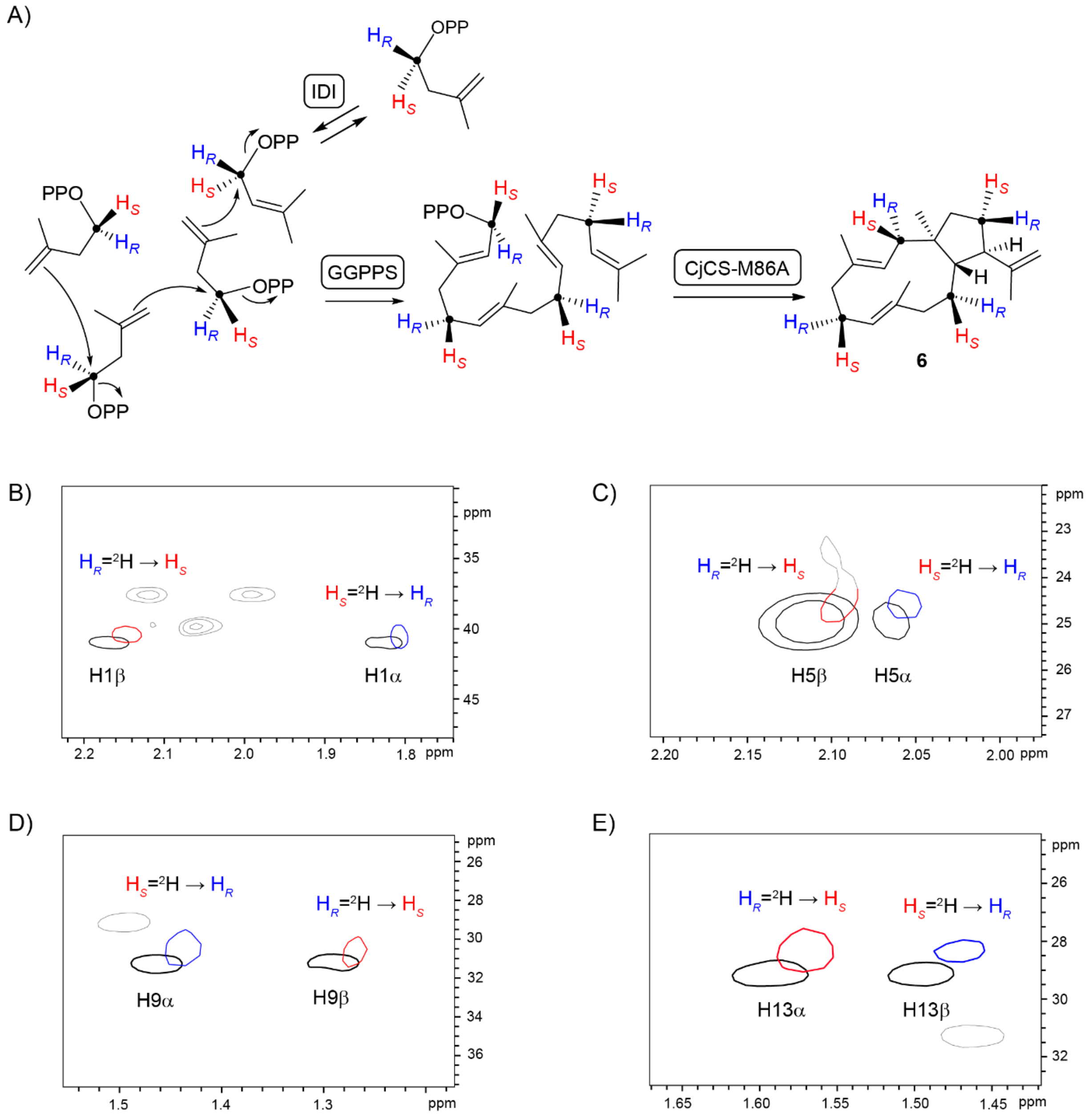

Figure S96. The absolute configuration of **6**. A) Conversion of (*R*)- and (*S*)-(1-^{13}C,1-^{2}H)IPP by IDI, GGPPS and CjCS-M86A into **6**. Three overlaid HSQC spectra at positions B) C1, C) C5, D) C9, and E) C13 for unlabelled **6** shown in black, for labelled **6** obtained with GGPPS and CjCS-M86A from (*S*)-(1-^{13}C,1-^{2}H)IPP shown in blue, and for labelled **5** obtained with GGPPS and CjCS-M86A from (*R*)-(1-^{13}C,1-^{2}H)IPP shown in red. Experiments C), D) and E) indicate the absolute configuration of **5**, while experiment B) confirms the inversion of configuration at C1 during terpene cyclisation.

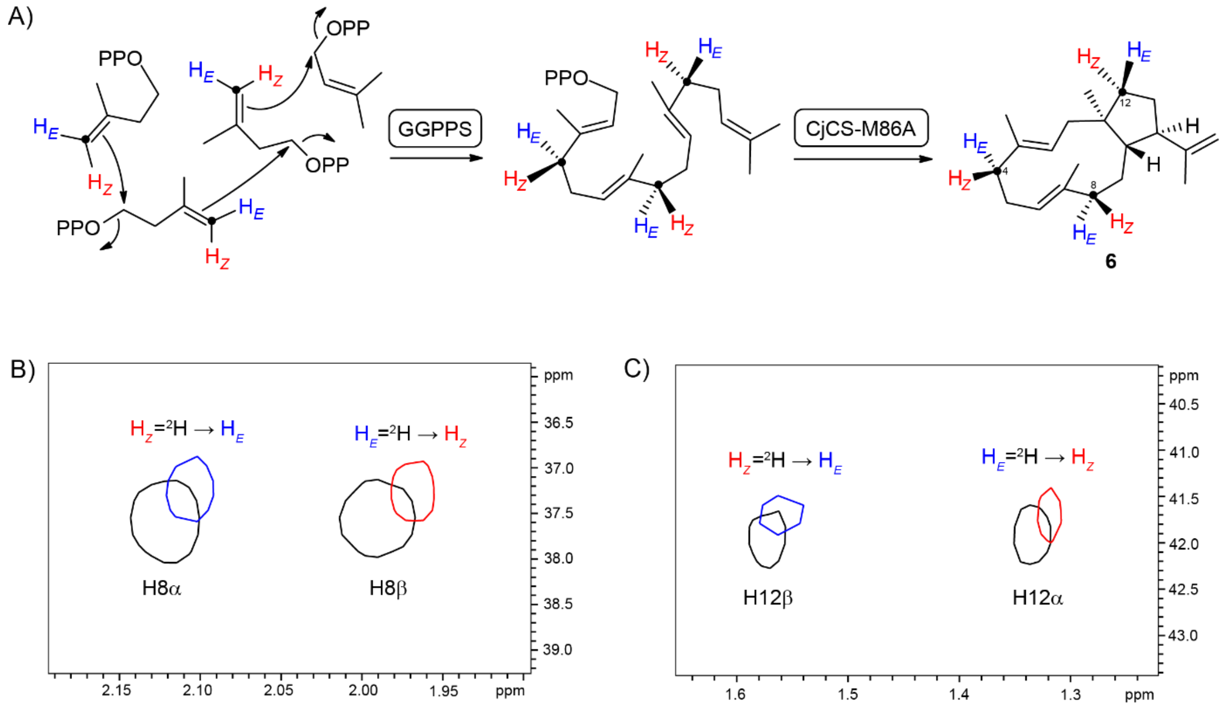

Figure S97. The absolute configuration of **6**. A) Conversion of DMAPP and (*E*)- and (*Z*)-(4-^{13}C,4-^{2}H)IPP by IDI, GGPPS and CjCS-M86A into **6**. Three overlaid HSQC spectra at positions B) C8, and C) C12 for unlabelled **6** shown in black, for labelled **6** obtained with GGPPS and CjCS-M86A from (*Z*)-(4-^{13}C,4-^{2}H)IPP shown in blue, and for labelled **6** obtained with GGPPS and CjCS-M86A from (*E*)-(4-^{13}C,4-^{2}H)IPP shown in red. Experiments B) and C) indicate the absolute configuration of **6**.

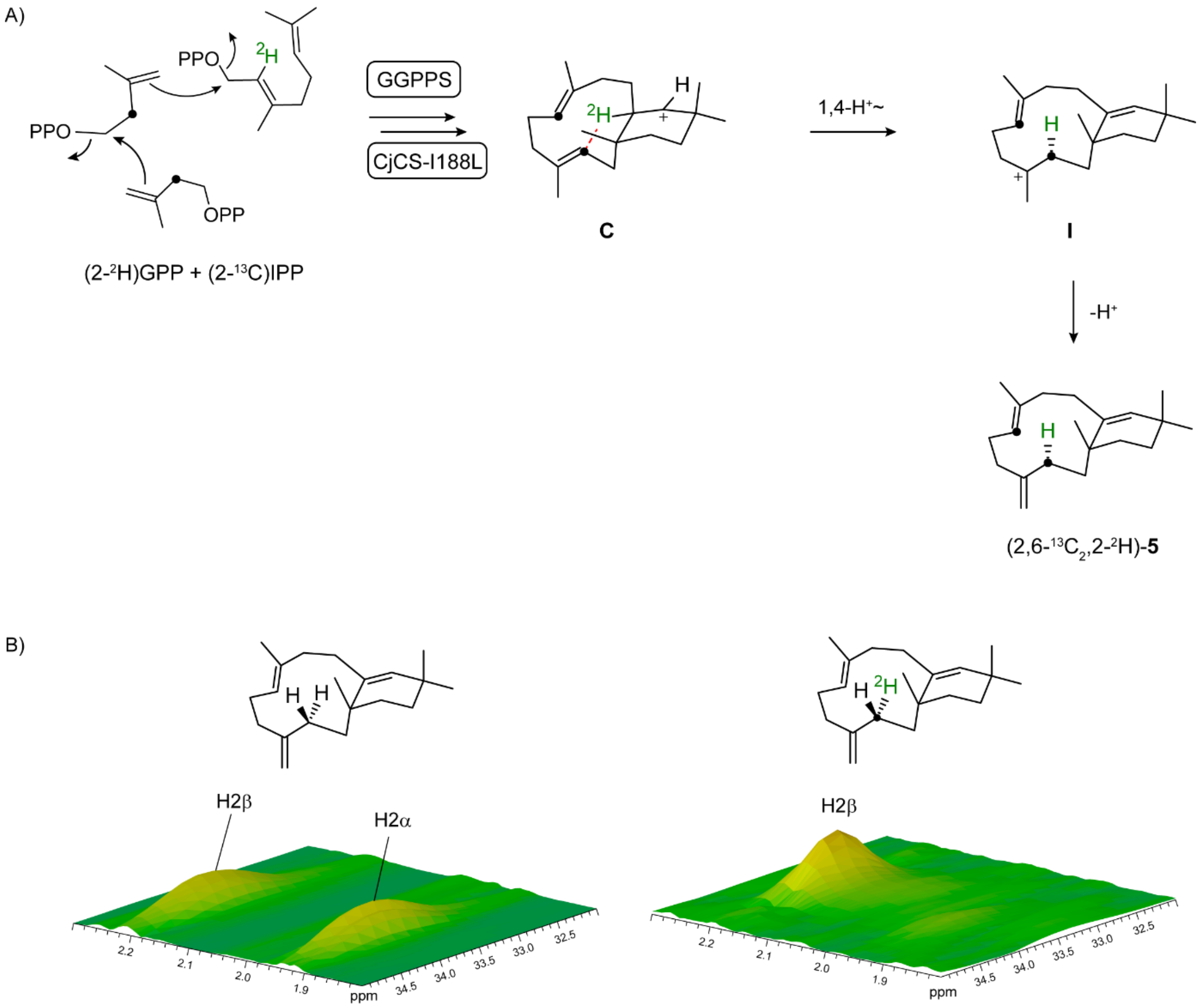

Figure S98. The 1,4-proton shift from **C** to **I** in the biosynthesis of **5**. A) Conversion of (2-^{2}H)GPP and (2-^{13}C)IPP with GGPPS and CjCS-I188L into labelled **5**. B) HSQC spectra of unlabelled **5** (left) showing the crosspeaks for the two hydrogens at C2, and of labelled **5** obtained from (2-^{2}H)GPP and (2-^{13}C)IPP showing only one crosspeak for H2β. This experiment confirms the proton migration from **C** to **I** with specific incorporation of deuterium into the H2α position.

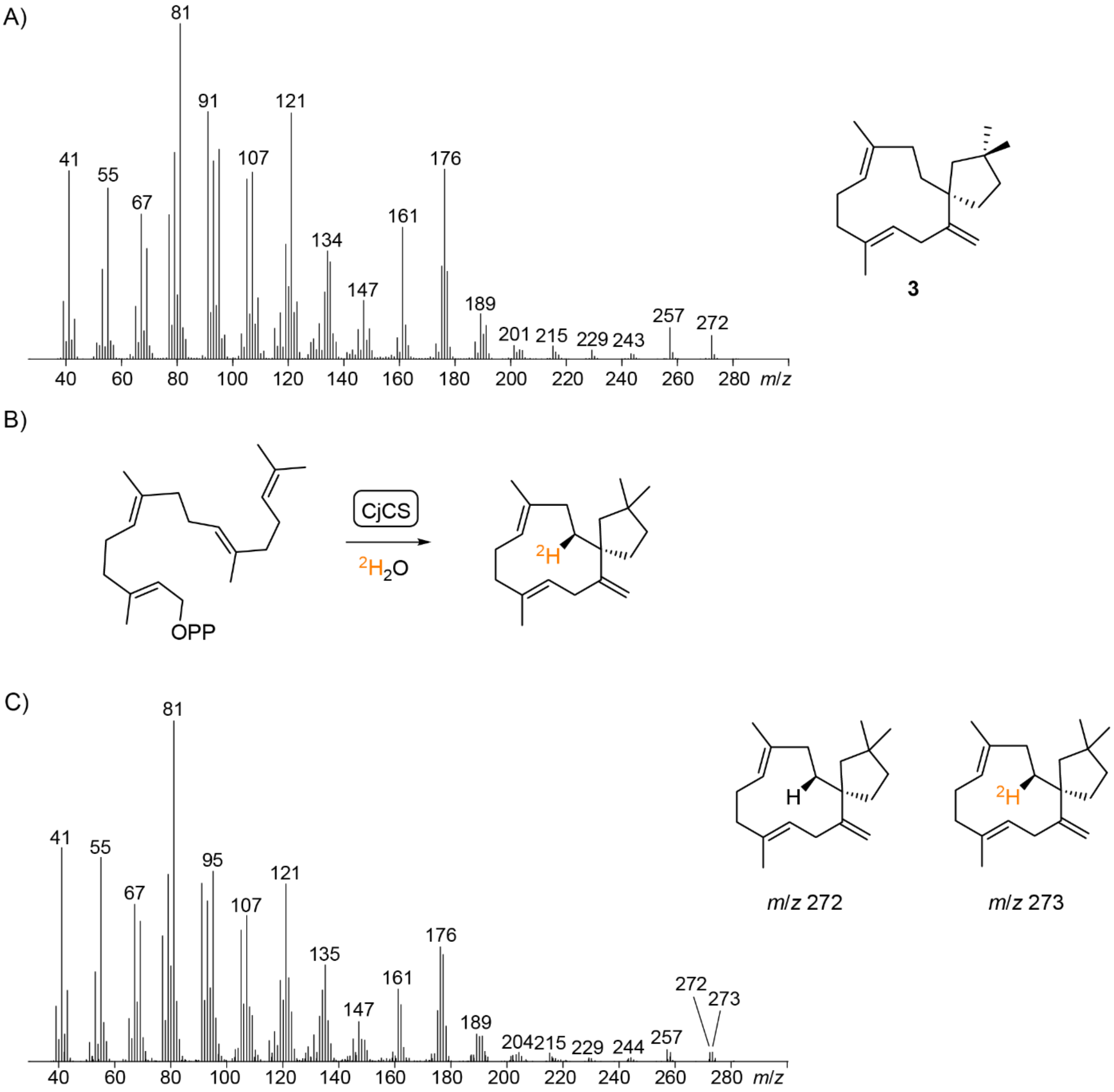

Figure S99. The deprotonation to **1** and its reprotonation by CjCS, leading to **3**. A) EI mass spectrum of unlabelled **3**, B) conversion of GGPP in deuterium oxide buffer by CjCS into labelled **3**. C) EI mass spectrum of labelled **3**, showing ca. 40% deuterium labelling in **3**.

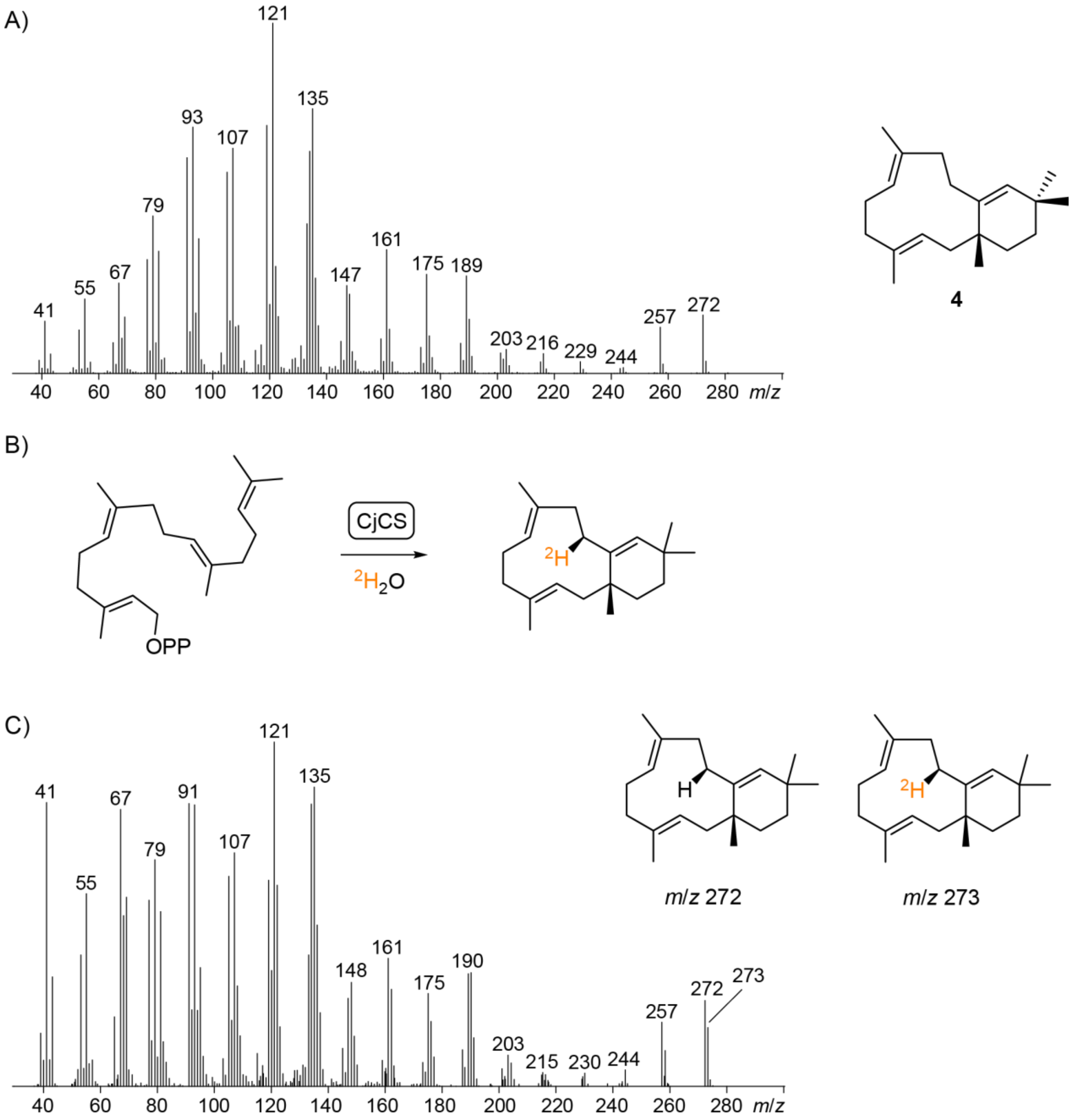

Figure S100. The deprotonation to **1** and its reprotonation by CjCS, leading to **4**. A) EI mass spectrum of unlabelled **4**, B) conversion of GGPP in deuterium oxide buffer by CjCS into labelled **4**. C) EI mass spectrum of labelled **4**, showing ca. 25% deuterium labelling in **3**.

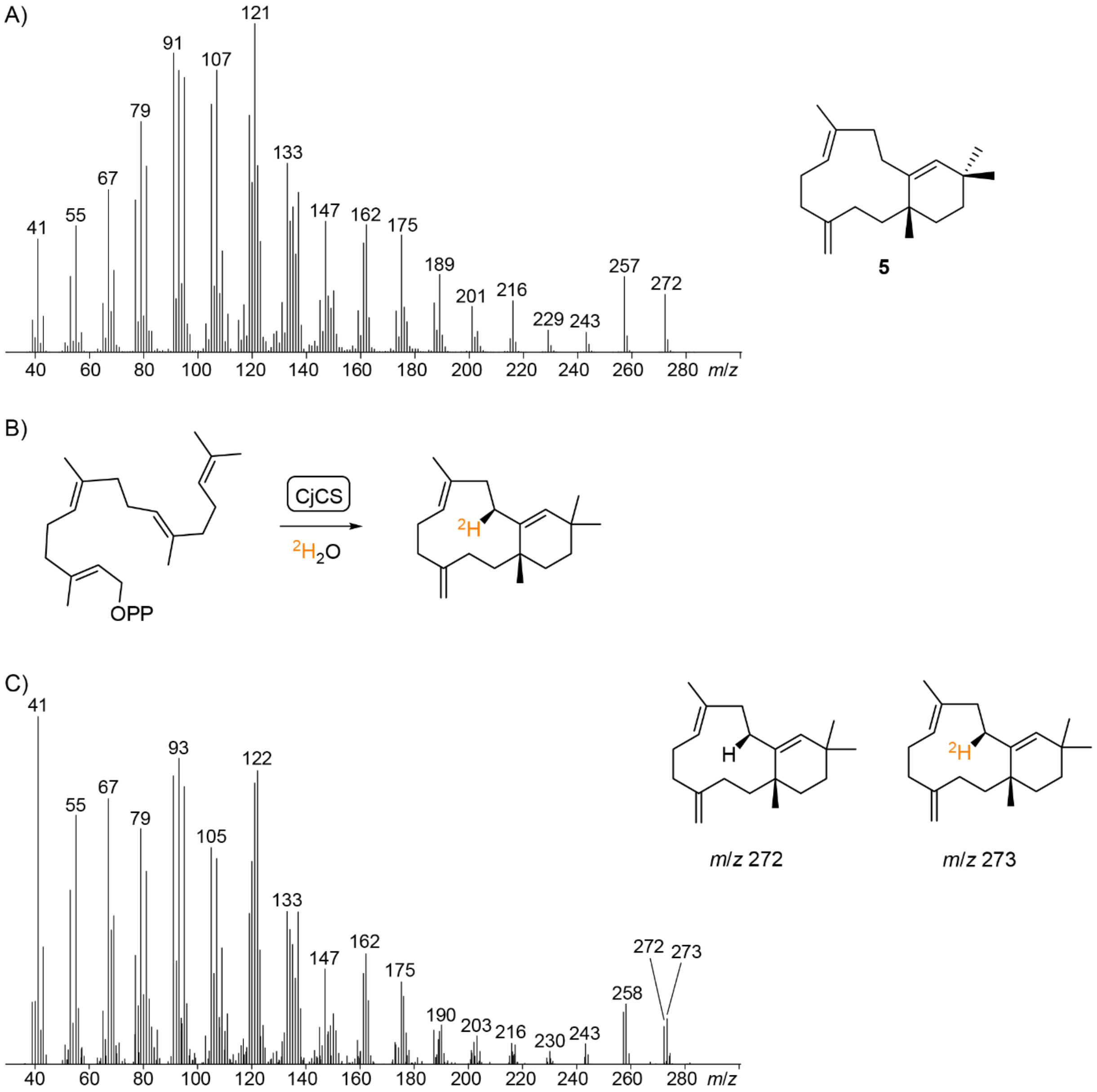

Figure S101. The deprotonation to **1** and its reprotonation by CjCS, leading to **5**. A) EI mass spectrum of unlabelled **5**, B) conversion of GGPP in deuterium oxide buffer by CjCS into labelled **5**. C) EI mass spectrum of labelled **5**, showing ca. 40% deuterium labelling in **5**.

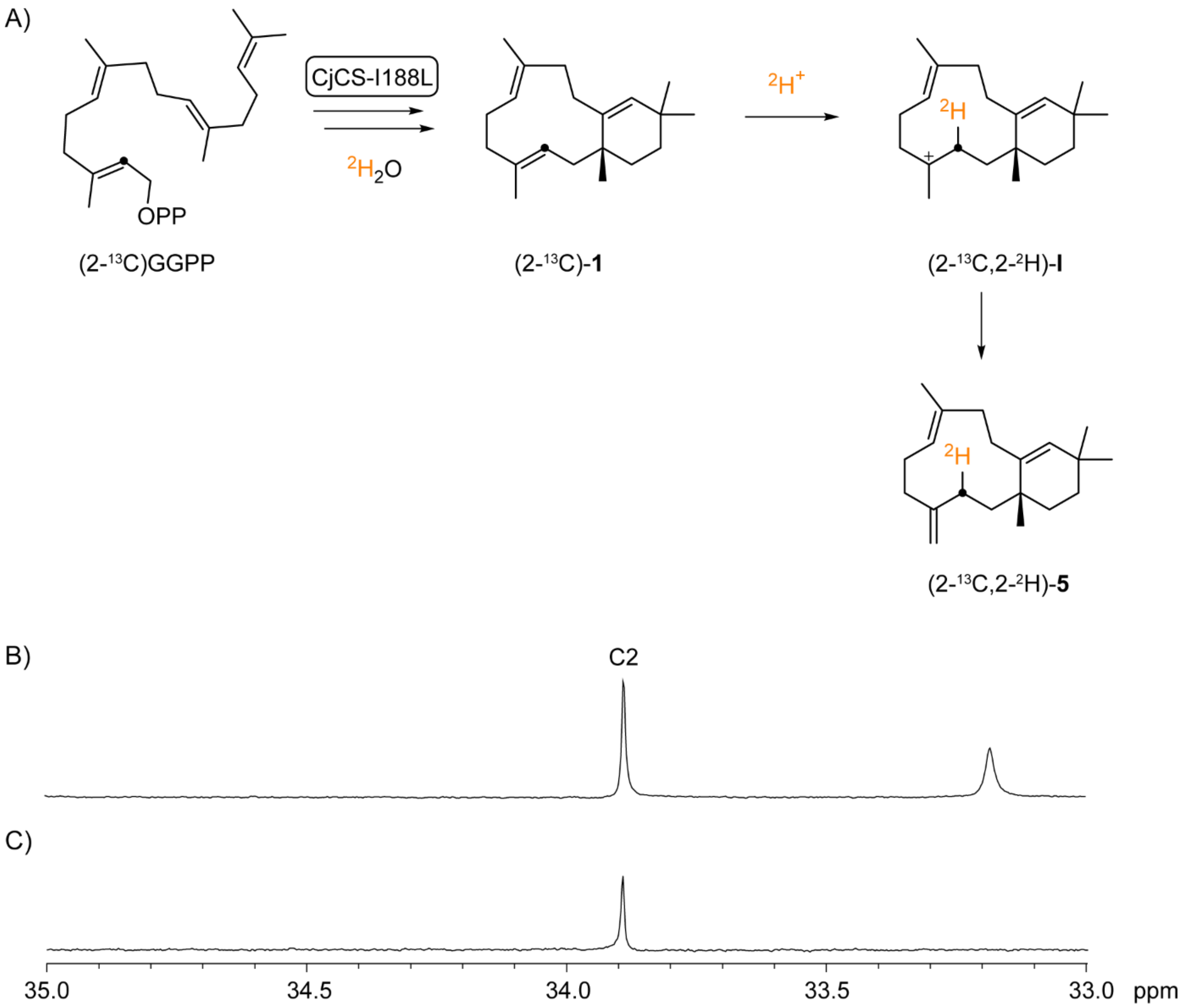

Figure S102. The reprotonation of **1** in the biosynthesis of **5**. A) Hypothetical mechanism involving a reprotonation of **1** at C2 to yield intermediate **I**. ^{13}C-NMR spectra of B) unlabelled **5** showing the region for C2, and C) labelled **5** obtained from (2-^{13}C)GGPP after incubation with CjCS-I188L in deuterium oxide buffer. The unchanged singlet for C2 of labelled **5** excludes a direct formation through reprotonation of **1** at C2. Black dots indicate ^{13}C-labelled carbons.

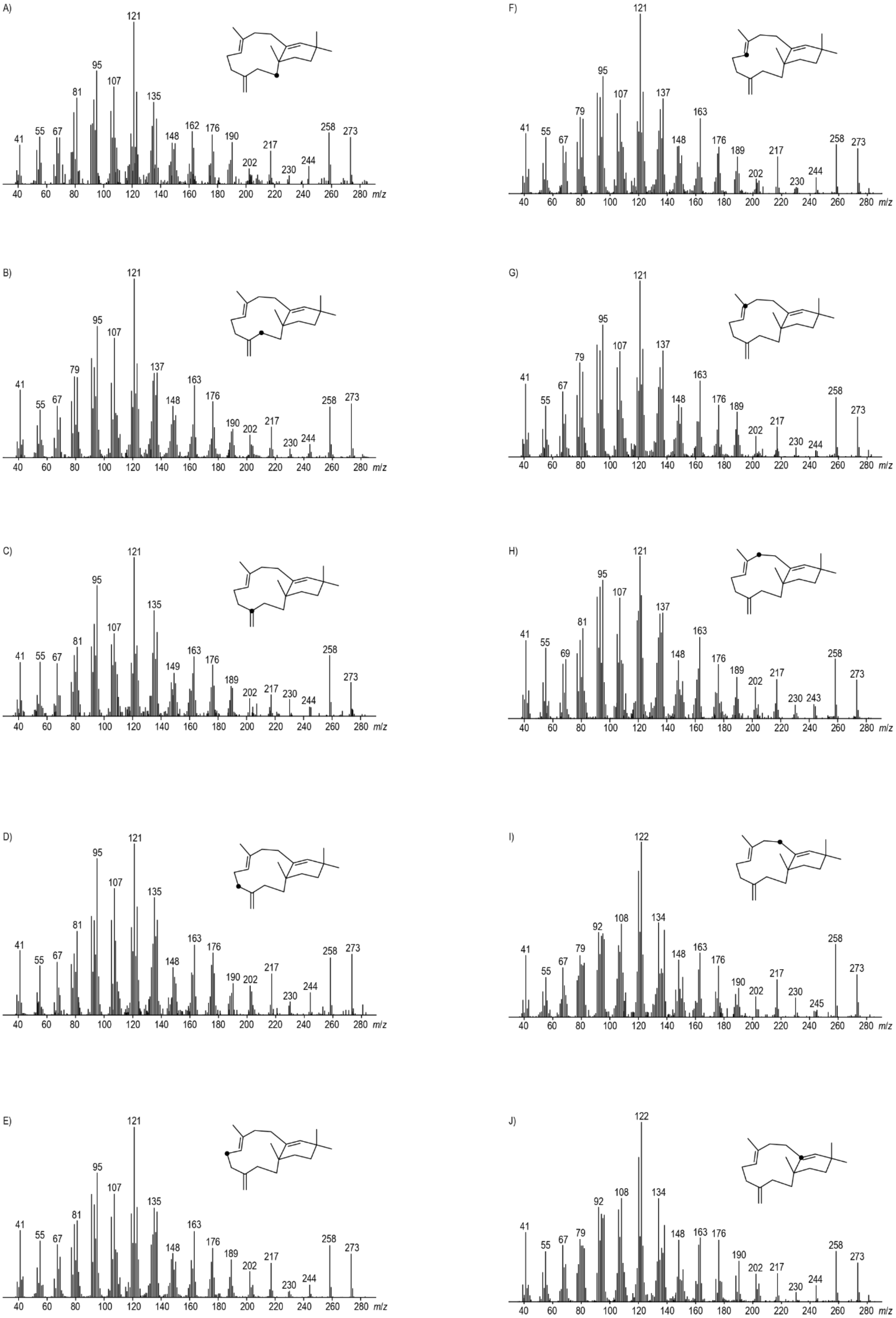

Figure S103. EI mass spectra of the ten isotopomers of (^{13}C)-**5** obtained with CjCS from A) (1-^{13}C)GGPP to J) (10-^{13}C)GGPP. Black dots indicate ^{13}C-labelled carbons.

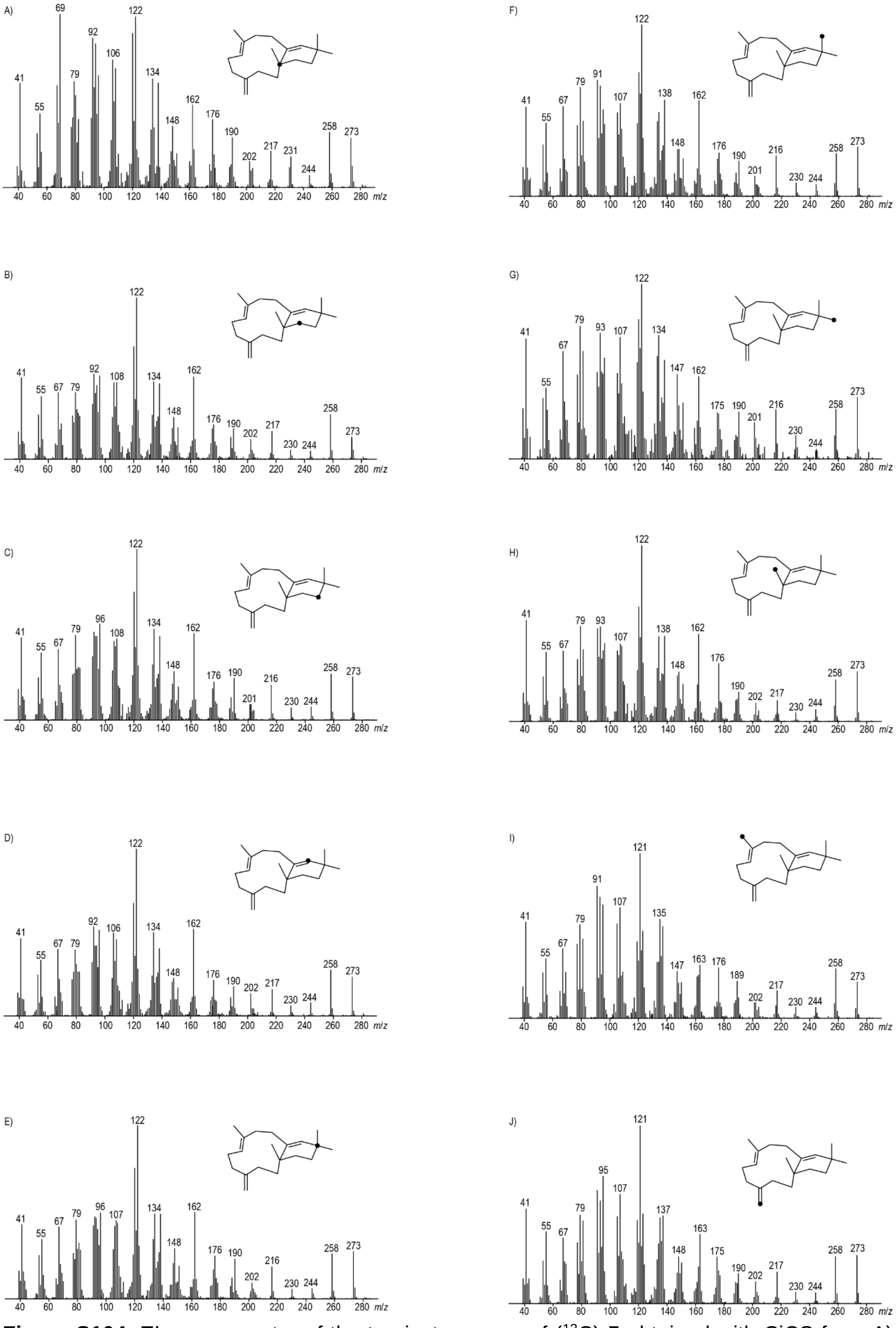

Figure S104. EI mass spectra of the ten isotopomers of (^{13}C)-**5** obtained with CjCS from A) (11-^{13}C)GGPP to J) (20-^{13}C)GGPP. Black dots indicate ^{13}C-labelled carbons.

Table S16. Results of DFT calculations for the EI-MS fragmentation from $\mathbf{5^{+\bullet}}$ – $\mathbf{O^{+\bullet}}$ (Scheme 2 of main text).

Structure	Gibbs energy (298.15K) in Hartree	energy relative to $5^{+\bullet}$ in kcal/mol	reaction barrier in kcal/mol	Gibbs free energy in kcal/mol
**$5^{+\bullet}$**	–781.088383	0.00		
**$5^{+\bullet}$-$L^{+\bullet}$-TS**	–781.057613	19.31	19.31	
**$L^{+\bullet}$**	–781.077208	7.01		7.01
**$L^{+\bullet}$**	–781.077207	7.01		
**$L^{+\bullet}$-$M^{+\bullet}$-TS**	–781.072939	9.69	2.68	
**$M^{+\bullet}$**	–781.079026	5.87		–1.14
**$M^{+\bullet}$**	–781.071219	10.77		
**$M^{+\bullet}$-$O^{+\bullet}$-TS**	–781.009982	49.20	38.43	
**$O^{+\bullet}$**	–781.006668	51.28		40.51

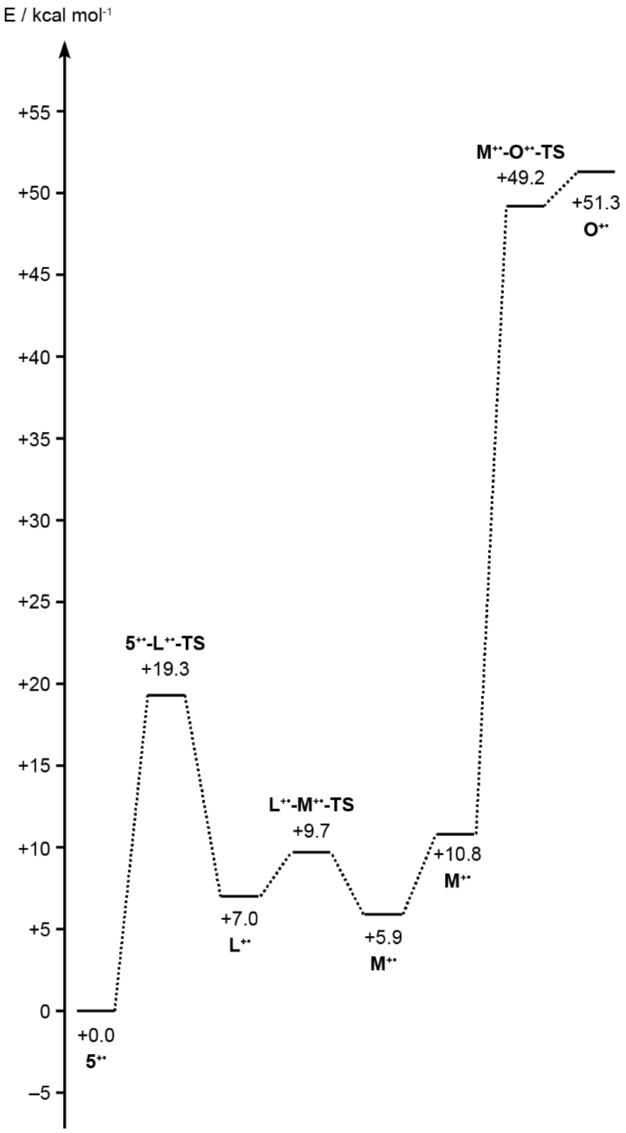

Figure S105. Computed energy profile for the EI-MS fragmentations from $\mathbf{5^{+\bullet}}$ to $\mathbf{O^{+\bullet}}$ (Scheme 2 of main text, mPW1PW91/6-311+G(d,p)//B97D3/6-31g(d,p), 298 K).

Enzymatic conversion of substrate analogs with CjCS and compound isolation
Large scale incubation was done by dissolving 20-*nor*-GGPP (80 mg, 0.17 mmol) in substrate buffer (15 mL). A mixture of protein preparations of recombinant CjCS (2.5 mL; from 8 L expression culture, 1.9 mg/mL) and incubation buffer (100 mL) were then added. The reaction mixture was incubated overnight at 28 °C and then extracted n-hexane (3 x 200 mL). The combined organic extracts were dried with $MgSO_4$ and concentrated in vaccuo. Repeated column chromatography on silica gel with n-pentane yielded 20-*nor*-chryseojoostene A (1.29 mg, 5.0 μmol, 3.1%), macrojoostene (1.21 mg, 4.7 μmol, 2.9%) and a mixture of two compounds which was separated by reversed-phase HPLC eluted with acetonitrile-water (9:1) to give 20-*nor* chryseojoostene C (0.76 mg, 2.9 μmol, 1.8%) and 20-*nor*-chryseojoostene D (0.83 mg, 3.2 μmol, 1.9%).

Following the same method, 6,7-dihydro-GPP (trisammonium salt, 80 mg, 0.25 mmol), IPP (trisammonium salt, 60 mg, 0.25 mmol) in NH_4HCO_3 (25 mM, 20 mL) and an enzyme preparation of CjCS (40 mL; from 8 L expression culture, 2.3 mg mL^{-1}) and GGPPS (30 mL; from 6 L expression culture, 2.1 mg mL^{-1}) were added to incubation buffer (100 mL). Several rounds of purification of the obtained crude extract afforded 13-isopentenyl-β-selinene (0.63 mg, 2.25 μmol, 0.93%) and (*Z*)-12-isopentenyl-β-selinene (0.68 mg, 2.43 μmol, 1.1%).

13-Isopentenyl-β-selinene (11). TLC (pentane): R_f = 0.42. GC (HP-5MS): *I* = 1956. MS (EI, 70 eV): *m*/*z* (%) = 274 (42), 259 (12), 245 (4), 231 (4), 217 (3), 204 (13), 189 (56), 175 (23), 161 (100), 147 (50), 133 (54), 121 (53), 105 (91), 93 (73), 79 (70), 67 (51), 55 (35), 41 (23). IR (diamond ATR): ṽ = 2927 (s), 2866 (s), 2844 (m), 1738 (m), 1645 (m), 1465 (m), 1441 (m), 1229 (w), 1216 (w), 1090 (w), 1019 (w), 886 (m), 799 (w) cm^{-1}. ESI-(+)-HR-MS: calc. $[C_{20}H_{35}]^+$ *m*/*z* = 275.2733; found: *m*/*z* = 275.2733. Optical rotation: $[\alpha]_D^{25}$ = +17.5 (*c* 0.063, CH_2Cl_2). NMR data are given in Table S17.

(*Z*)-12-Isopentenyl-β-selinene (13). TLC (pentane): R_f = 0.42. GC (HP-5MS): *I* = 1918. MS (EI, 70 eV): *m*/*z* (%) = 274 (54), 259 (10), 245 (4), 231 (3), 217 (5), 203 (24), 189 (41), 175 (13), 161 (100), 147 (41), 133 (50), 121 (47), 107 (61), 95 (73), 81 (72), 67 (41), 55 (34), 41 (21). IR (diamond ATR): ṽ = 2951 (s), 2925 (s), 2865 (m), 1735 (w), 1645 (w), 1632 (w), 1457 (w), 1409 (w), 1378 (m), 1260 (w), 1092 (m), 1015 (m), 885 (m), 799 (m) cm^{-1}. ESI-(+)-HR-MS: calc. $[C_{20}H_{35}]^+$ *m*/*z* = 275.2733; found: *m*/*z* = 275.2733. Optical rotation: $[\alpha]_D^{25}$ = –1.5 (*c* 0.068, CH_2Cl_2). NMR data are given in Table S18.

20-*nor*-Chryseojoostene A (1a). TLC (pentane): R_f = 0.62. GC (HP-5MS): *I* = 1847. MS (EI, 70 eV): *m*/*z* (%) = 258 (45), 243 (13), 229 (3), 215 (5), 203 (20), 189 (53), 176 (50), 161 (33), 148 (3), 133 (60), 119 (78), 105 (100), 91 (99), 80 (84), 69 (40), 55 (33), 41 (31). IR (diamond ATR): ṽ = 2949 (s), 2925 (s), 2852 (s), 1664 (w), 1445 (m), 1381 (m), 1363 (m), 1293 (w), 1260 (w), 1207 (w), 1045 (w), 967 (m), 872 (w), 826 (w) cm^{-1}. ESI-(+)-HR-MS: calc. $[C_{19}H_{31}]^+$ *m*/*z* = 259.2420; found: *m*/*z* = 259.2420. Optical rotation: $[\alpha]_D^{25}$ = +141.8 (*c* 0.129, CH_2Cl_2). NMR data are given in Table S19.

20-*nor*-Chryseojoostene C (3a). TLC (pentane): R_f = 0.63. GC (HP-5MS): *I* = 1888. MS (EI, 70 eV): *m*/*z* (%) = 258 (2), 243 (9), 230 (3), 217 (2), 204 (5), 191 (12), 177 (24), 161 (35), 148 (16), 135 (33), 121 (97), 108 (79), 95 (100), 91 (89), 81 (88), 67 (44), 54 (30), 41 (21). IR (diamond ATR): ṽ = 2948 (s), 2925 (s), 2856 (s), 1659 (w), 1625 (w), 1461 (m), 1445 (m), 1363 (w), 1259 (w), 1021 (w), 964 (w), 884 (w), 818 (w), 803 (w) cm^{-1}. ESI-(+)-HR-MS: calc. $[C_{19}H_{31}]^+$ *m*/*z* = 259.2420; found: *m*/*z* = 259.2420. Optical rotation: $[\alpha]_D^{25}$ = +6.6 (*c* 0.076, CH_2Cl_2). NMR data are given in Table S20.

20-*nor*-Chryseojoostene D (4a). TLC (pentane): R_f = 0.58. GC (HP-5MS): *I* = 1854. MS (EI, 70 eV): *m*/*z* (%) = 258 (18), 243 (30), 229 (5), 215 (5), 202 (29), 189 (65), 175 (52), 161 (51),

147 (38), 135 (100), 121 (95), 105 (76), 91 (83), 79 (57), 67 (28), 54 (21), 41 (20). IR (diamond ATR): ṽ = 2952 (s), 2921 (s), 2858 (s), 1660 (w), 1632 (w), 1455 (m), 1378 (w), 1259 (s), 1089 (s), 1075 (s), 1012 (s), 969 (m), 834 (m), 791 (s), 701 (w) cm^{-1}. ESI-(+)-HR-MS: calc. $[C_{19}H_{31}]^+$ *m*/*z* = 259.2420; found: *m*/*z* = 259.2420. Optical rotation: $[\alpha]_D^{25}$ = +16.8 (*c* 0.083, CH_2Cl_2). NMR data are given in Table S21.

Macrojoostene (14). TLC (pentane): R_f = 0.48. GC (HP-5MS): *I* = 1952. MS (EI, 70 eV): *m*/*z* (%) = 258 (15), 243 (4), 229 (2), 215 (3), 201 (2), 187 (4), 175 (5), 161 (5), 147 (10), 133 (10), 121 (16), 107 (26), 94 (58), 79 (100), 67 (29), 55 (15), 41 (10). IR (diamond ATR): ṽ = 2956 (s), 2923 (s), 2853 (s), 1633 (w), 1467 (w), 1434 (m), 1380 (w), 1345 (w), 1101 (w), 1020 (w), 967 (m), 878 (w), 805 (w) cm^{-1}. ESI-(+)-HR-MS: calc. $[C_{19}H_{31}]^+$ *m*/*z* = 259.2420; found: *m*/*z* = 259.2420. NMR data are given in Table S22.

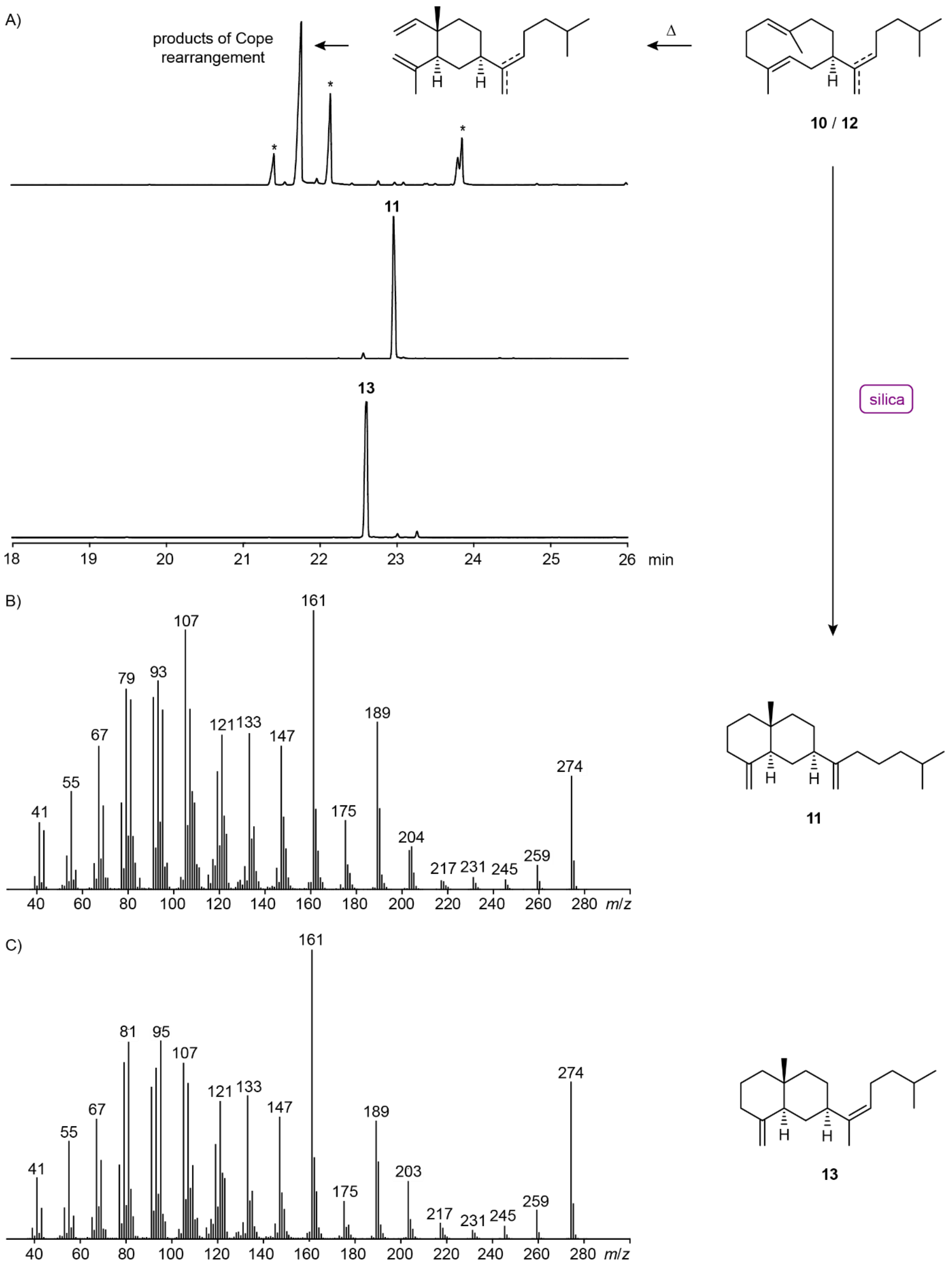

Figure S106. Products obtained from 14,15-dihydro-GGPP with CjCS. A) Total ion chromatograms of a crude extract of an incubation of 6,7-dihydro-GPP and IPP with GGPPS and CjCS and of purified **10** and **12**. The crude extract does not show the presence of **10** and **12**, suggesting their formation during column chromatography on silica gel. Because of the short retention time the (coeluting) compound(s) detected in the crude extracts likely represent Cope rearrangement product(s). B) EI mass spectrum of isolated **9**, and C) EI mass spectrum of isolated **10**.

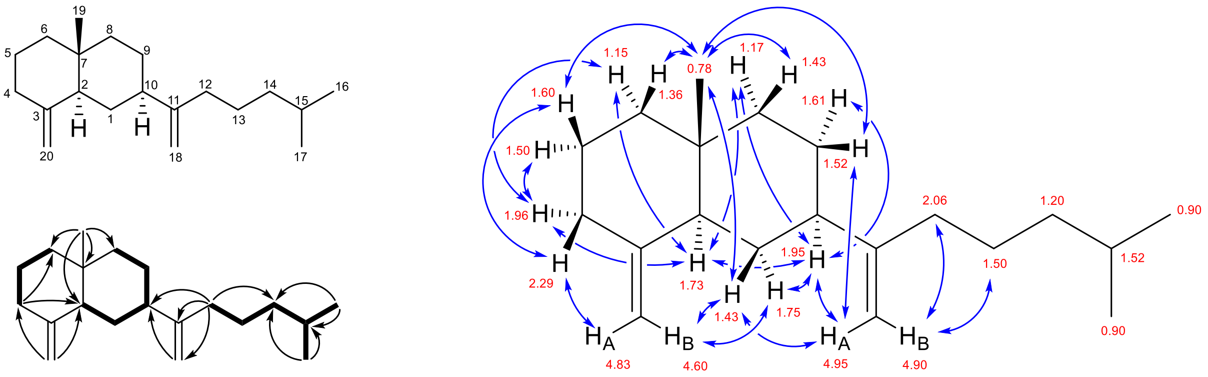

Figure S107. Structure elucidation of 13-isopentenyl-β-selinene (**11**). Bold: ^{1}H,^{1}H-COSY correlations, single headed arrows: HMBC correlations, and blue double headed arrows: NOESY correlations. Carbon numbering follows 14,15-dihydro-GGPP numbering to indicate the origin of each carbon.

Table S17. NMR data of 13-isopentenyl-β-selinene (**11**) in C_6D_6 recorded at 298 K.

C[a]	type	^{13}C[b]	^{1}H[b]
1	CH_2	30.57	1.75 (m)
			1.43 (m)
2	CH	50.30	1.73 (m)
3	C	150.86	–
4	CH_2	37.25	2.29 (dddd, J = 13.3, 3.9, 2.2, 2.1)
			1.96 (m)
5	CH_2	23.88	1.60 (m)
			1.50 (m)
6	CH_2	42.28	1.36 (m)
			1.15 (m)
7	C	36.23	–
8	CH_2	41.63	1.43 (m)
			1.17 (m)
9	CH_2	27.86	1.61 (m)
			1.52 (m)
10	CH	44.97	1.95 (m)
11	C	154.94	–
12	CH	35.68	2.06 (t, J = 7.6)
13	CH_2	26.52	1.50 (m, 2H)
14	CH_2	39.24	1.20 (m, 2H)
15	CH	28.32	–
16	CH_3	22.85	0.90 (d, J = 6.6)
17	CH_3	22.85	0.90 (d, J = 6.6)
18	CH_2	107.55	4.95 (br s, H_A)
			4.90 (br s, H_B)
19	CH_3	16.59	0.78 (s)
20	CH_2	105.97	4.83 (br s, H_A)
			4.60 (br s, H_B)

[a] Carbon numbering as shown in Figure S107 indicates the origin of each carbon from 14,15-dihydro-GGPP by same number. [b] Chemical shifts δ in ppm, multiplicity: s = singlet, d = doublet, t = triplet, m = multiplet, br = broad, coupling constants J are given in Hertz.

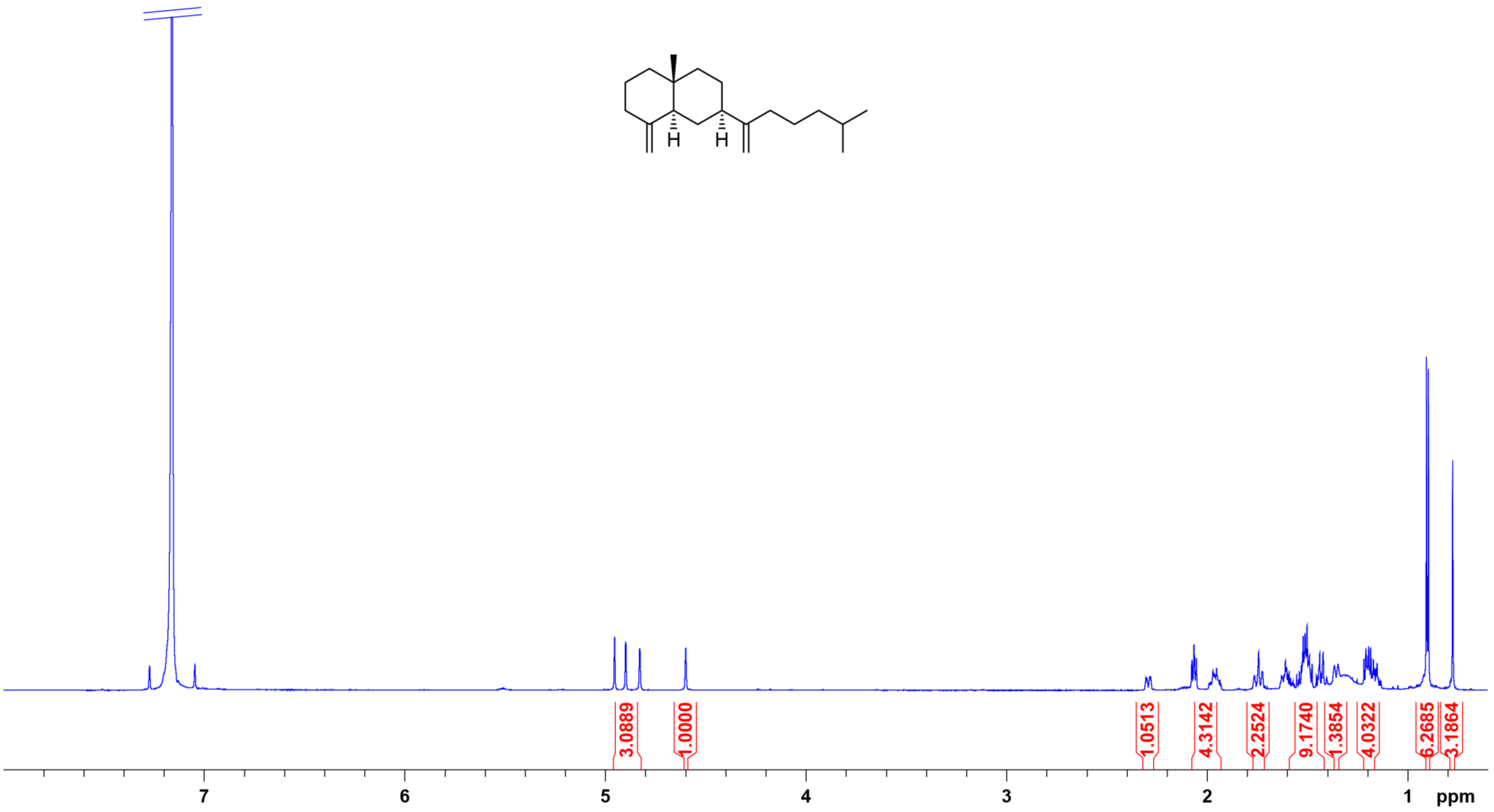

Figure S108. ^{1}H-NMR spectrum of **11** (700 MHz, C_6D_6).

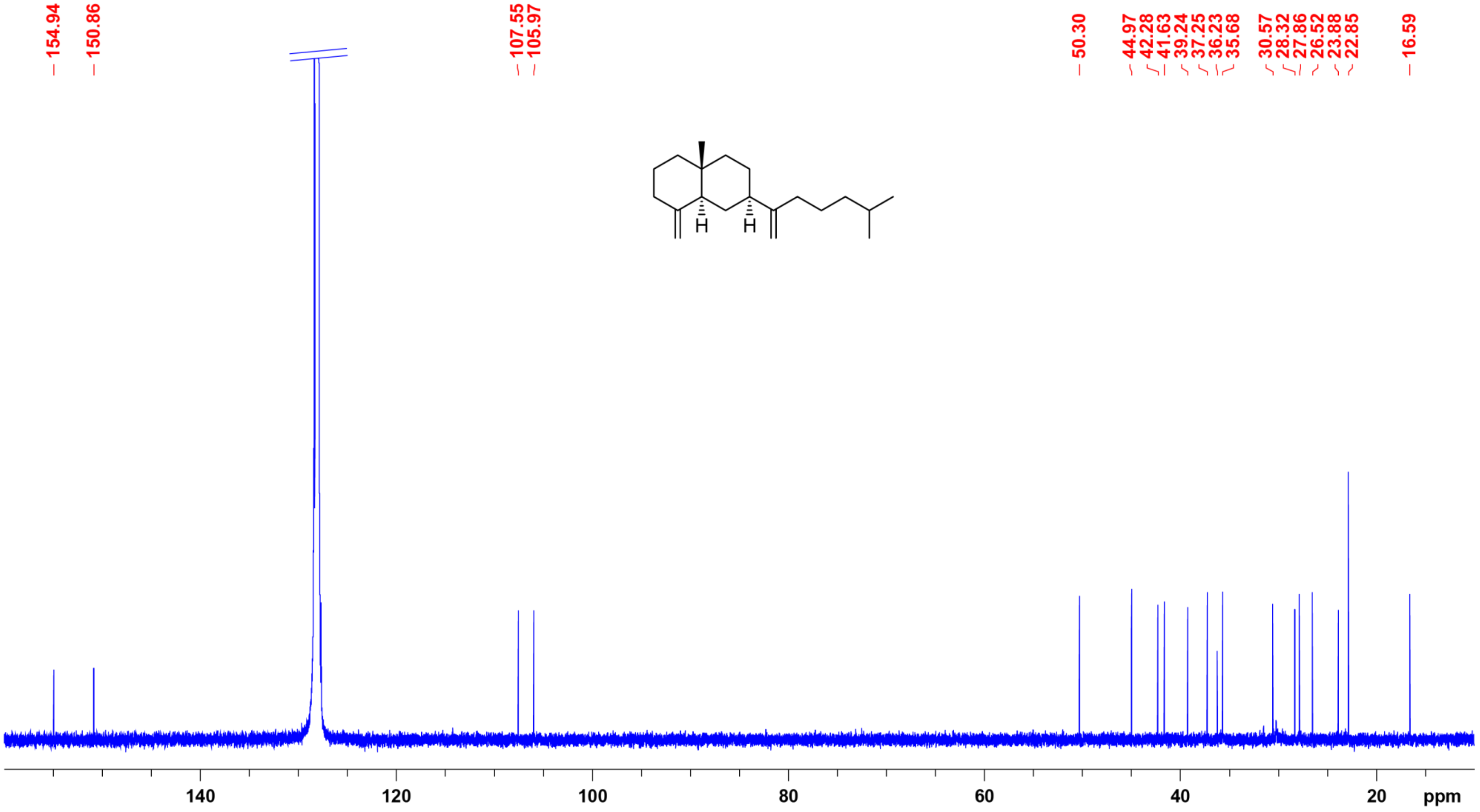

Figure S109. ^{13}C-NMR spectrum of **11** (176 MHz, C_6D_6).

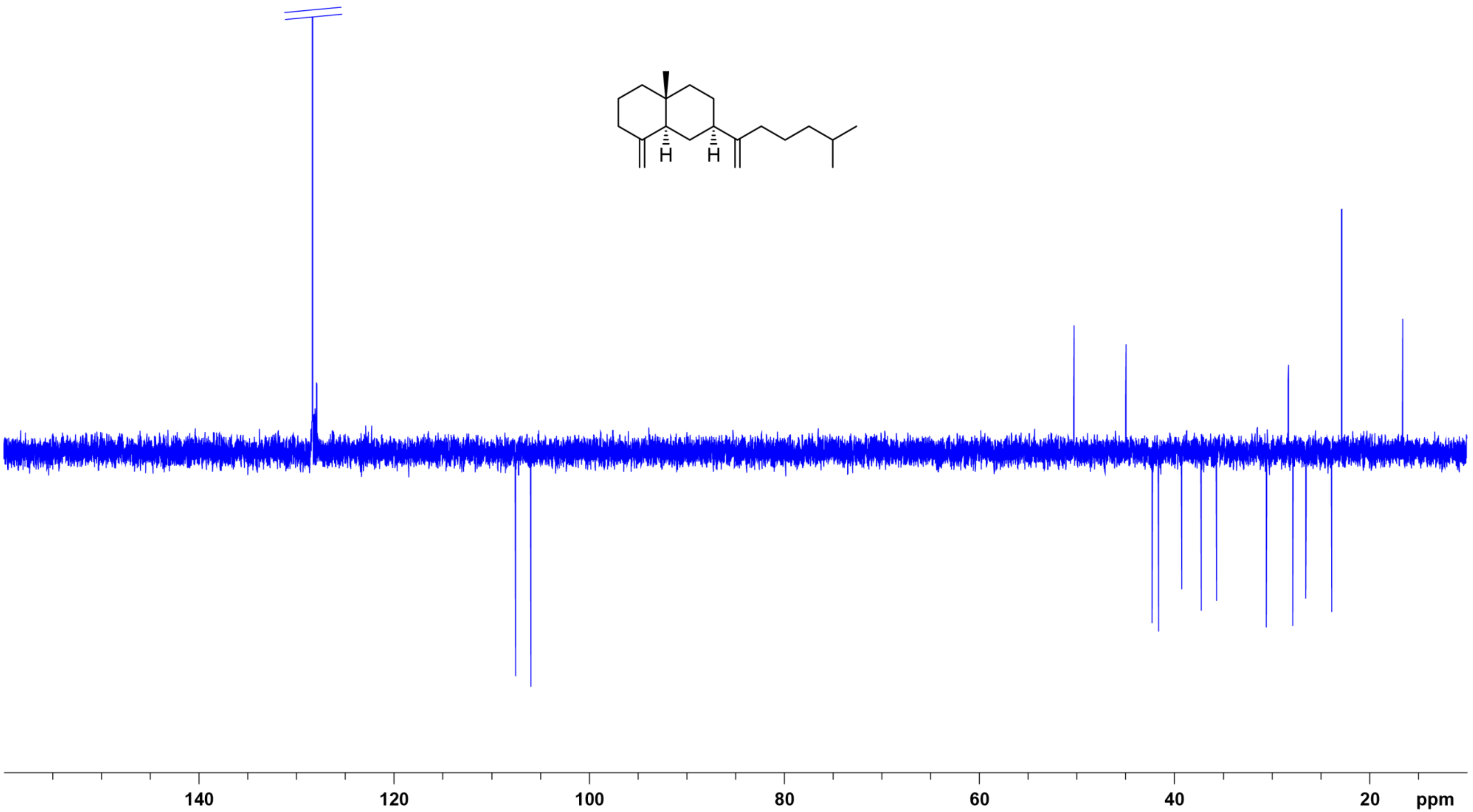

Figure S110. ^{13}C-DEPT135 spectrum of **11** (176 MHz, C_6D_6).

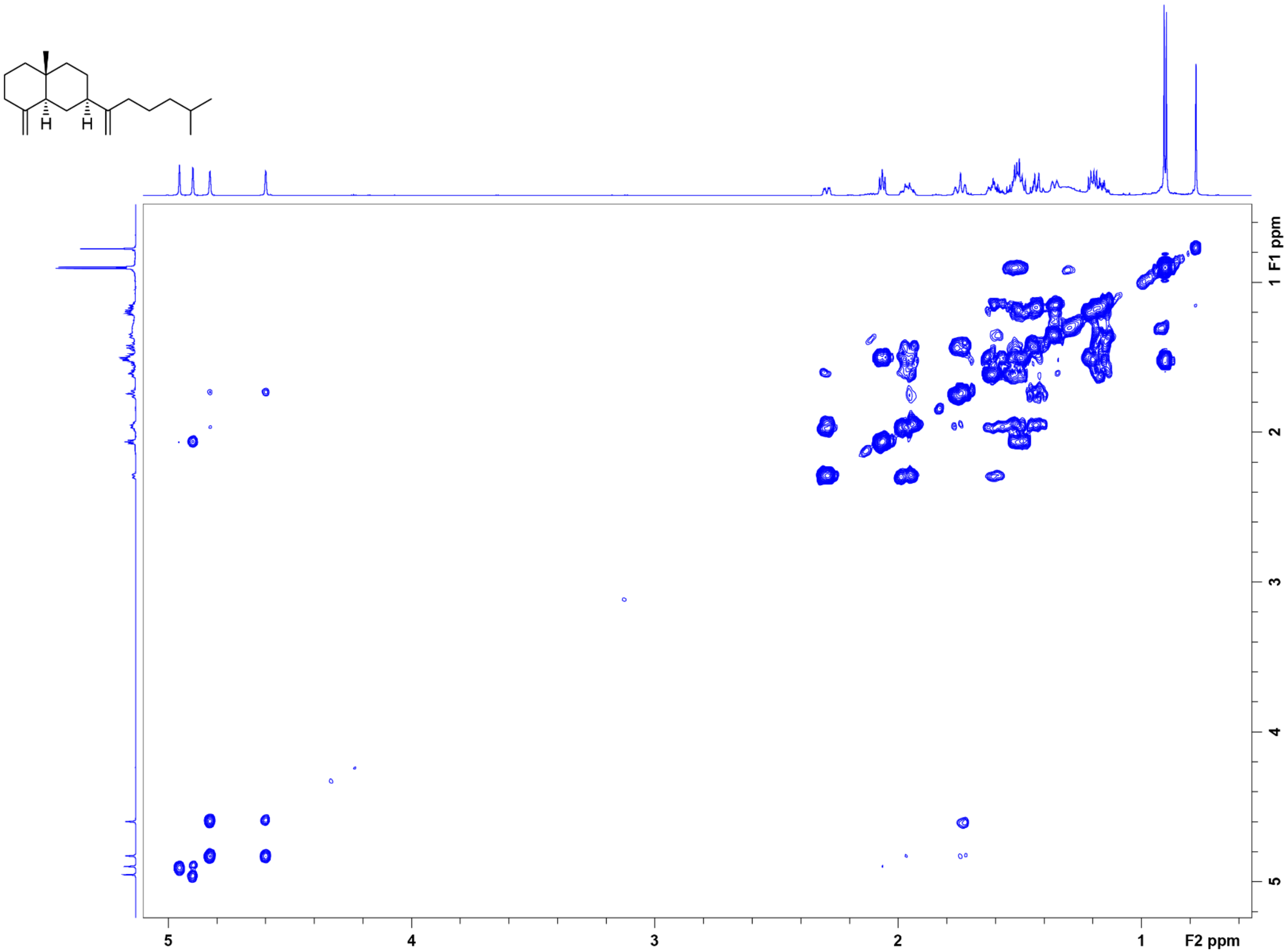

Figure S111. $^{1}H,^{1}H$-COSY spectrum (C_6D_6) of **11**.

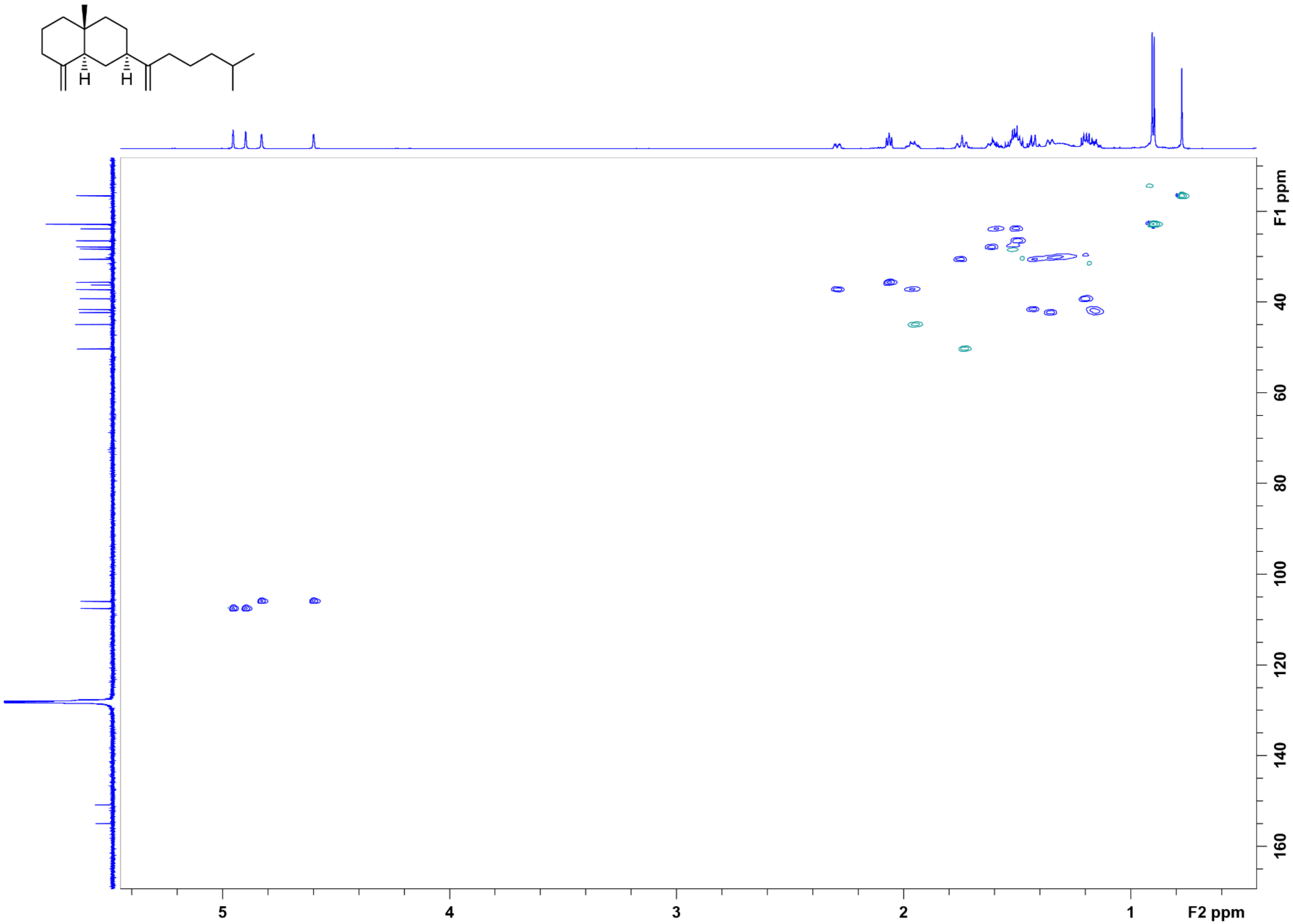

Figure S112. HSQC spectrum (C_6D_6) of **11**.

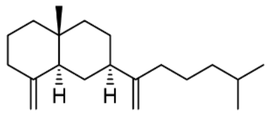

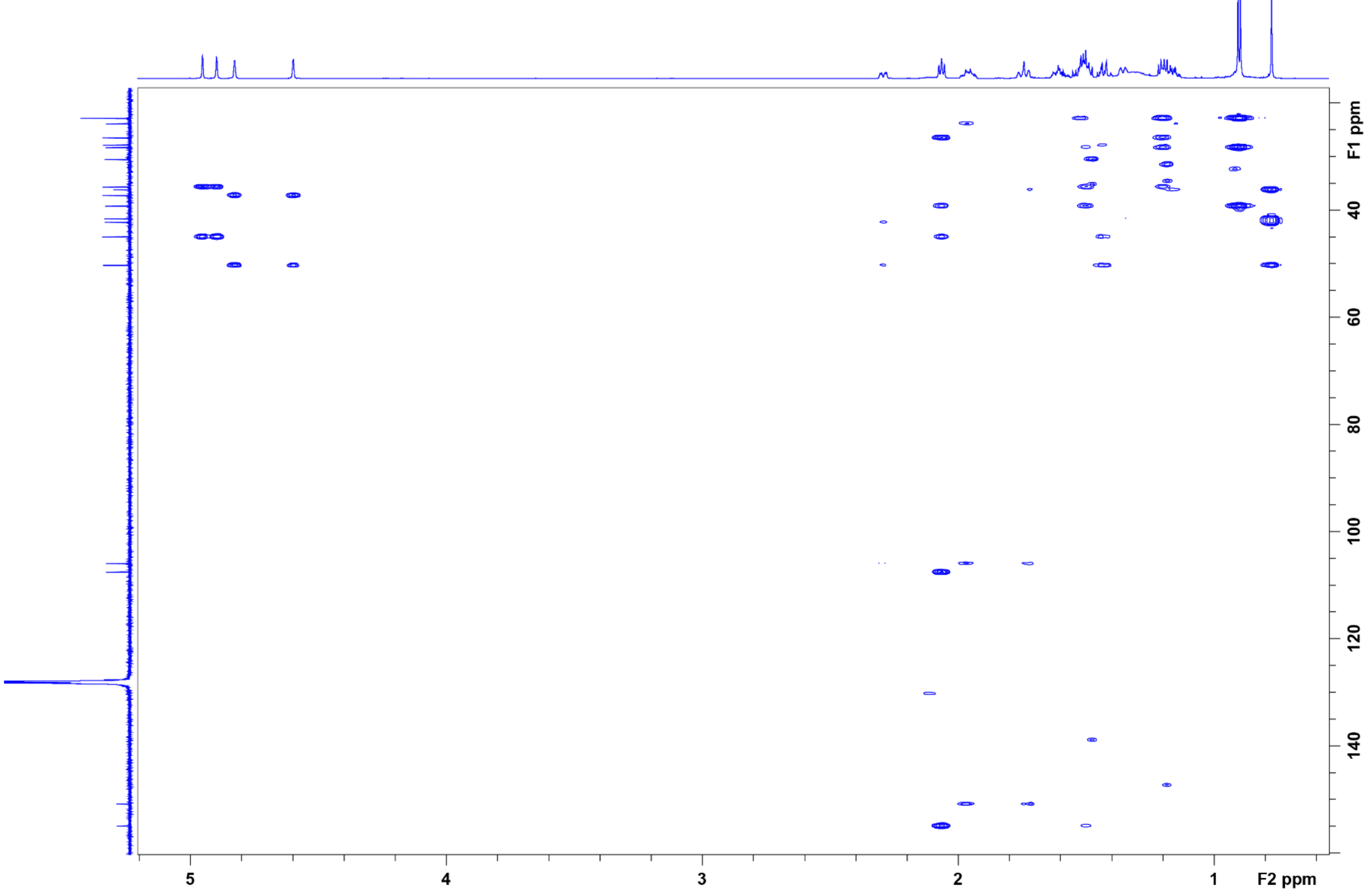

Figure S113. HMBC spectrum (C_6D_6) of **11**.

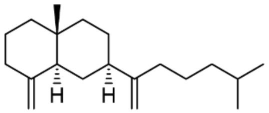

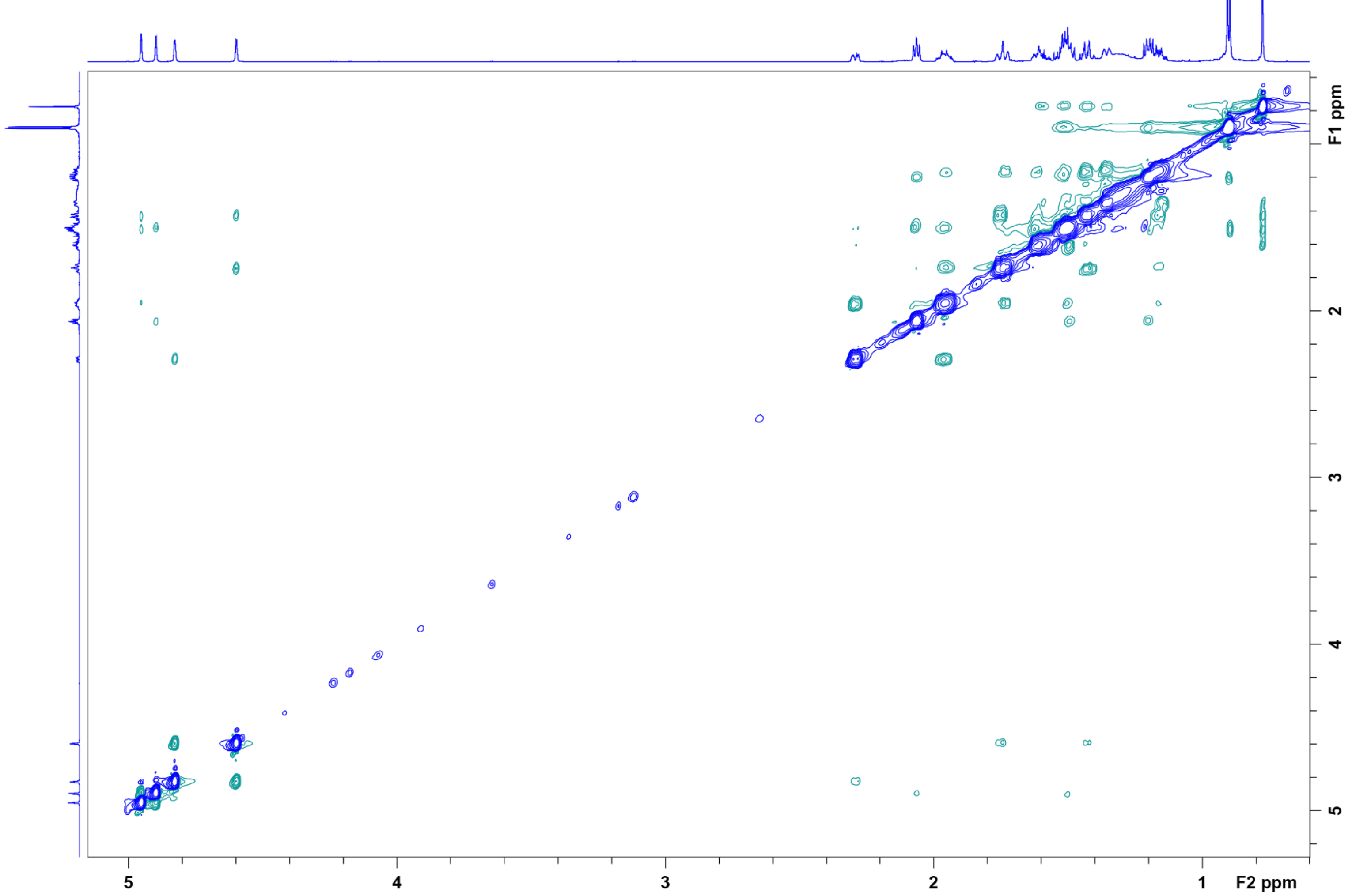

Figure S114. NOESY spectrum (C_6D_6) of **11**.

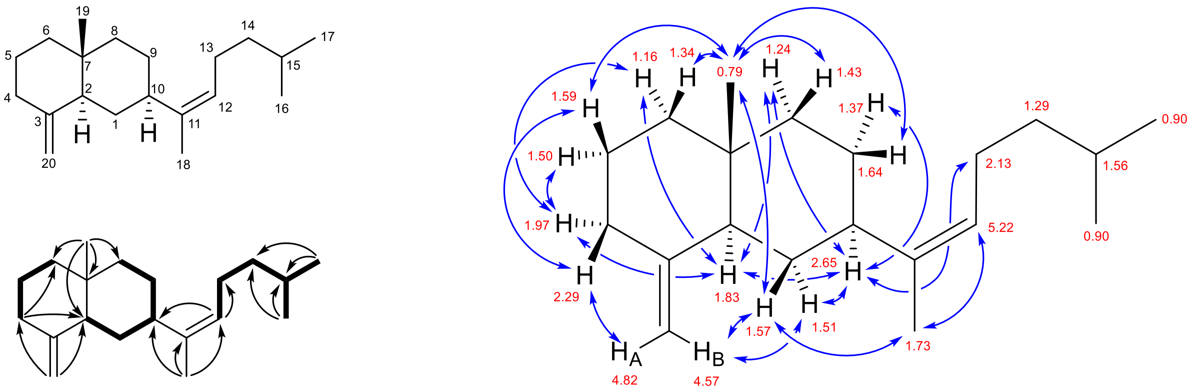

Figure S115. Structure elucidation of (*Z*)-12-isopentenyl-β-selinene (**13**). Bold: ^{1}H,^{1}H-COSY correlations, single headed arrows: HMBC correlations, and blue double headed arrows: NOESY correlations. Carbon numbering follows 14,15-dihydro-GGPP numbering to indicate the origin of each carbon.

Table S18. NMR data of (*Z*)-12-isopentenyl-β-selinene (**13**) in C_6D_6 recorded at 298 K.

C[a]	type	^{13}C[b]	^{1}H[b]
1	CH_2	29.03	1.57 (m)
			1.51 (m)
2	CH	50.09	1.83 (br d, *J* = 12.0)
3	C	150.85	–
4	CH_2	37.24	2.29 (dddd, *J* = 13.0, 3.8, 1.9, 1.8)
			1.97 (ddd, *J* = 13.5, 13.4, 5.6)
5	CH_2	23.86	1.59 (m)
			1.50 (m)
6	CH_2	42.29	1.34 (m)
			1.16 (m)
7	C	36.02	–
8	CH_2	41.40	1.43 (ddd, *J* = 12.9, 3.9, 2.6)
			1.24 (m)
9	CH_2	26.30	1.64 (dddd, *J* = 13.1, 13.0, 12.9, 3.7)
			1.37 (m)
10	CH	40.42	2.26 (tt, *J* = 12.3, 4.0)
11	C	139.33	–
12	CH	125.47	5.22 (t, *J* = 7.3)
13	CH_2	25.59	2.13 (m, 2H)
14	CH_2	39.95	1.29 (m, 2H)
15	CH	27.92	–
16	CH_3	22.79	0.90 (d, *J* = 6.7)
17	CH_3	22.77	0.90 (d, *J* = 6.7)
18	CH_3	20.05	1.73 (d, *J* = 1.2)
19	CH_3	16.57	0.79 (s)
20	CH_2	105.99	4.82 (br s, H_A)
			4.57 (br s, H_B)

[a] Carbon numbering as shown in Figure S115 indicates the origin of each carbon from 14,15-dihydro-GGPP by same number. [b] Chemical shifts δ in ppm, multiplicity: s = singlet, d = doublet, t = triplet, m = multiplet, br = broad, coupling constants *J* are given in Hertz.

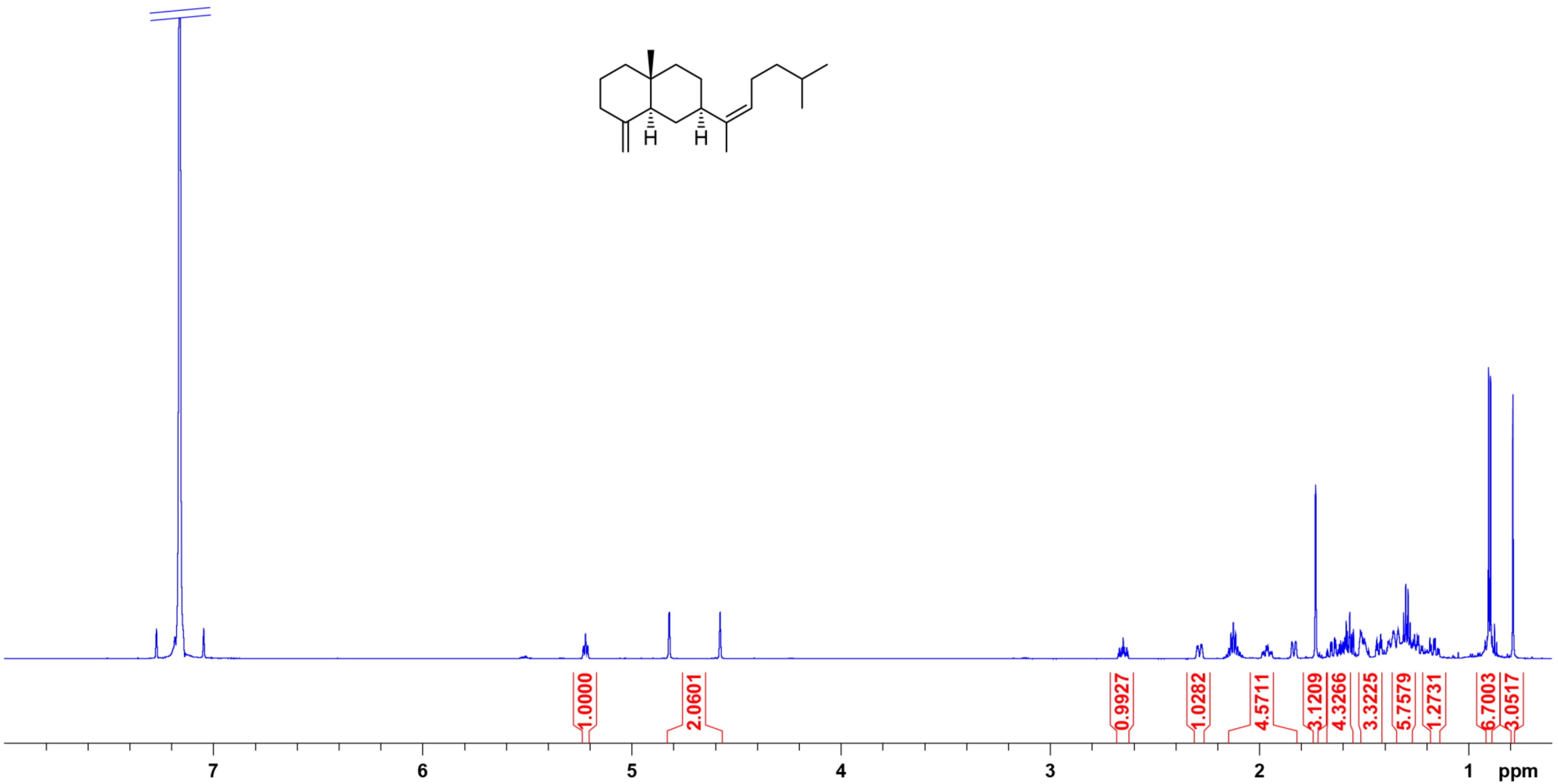

Figure S116. ^{1}H-NMR spectrum of **13** (700 MHz, C_6D_6).

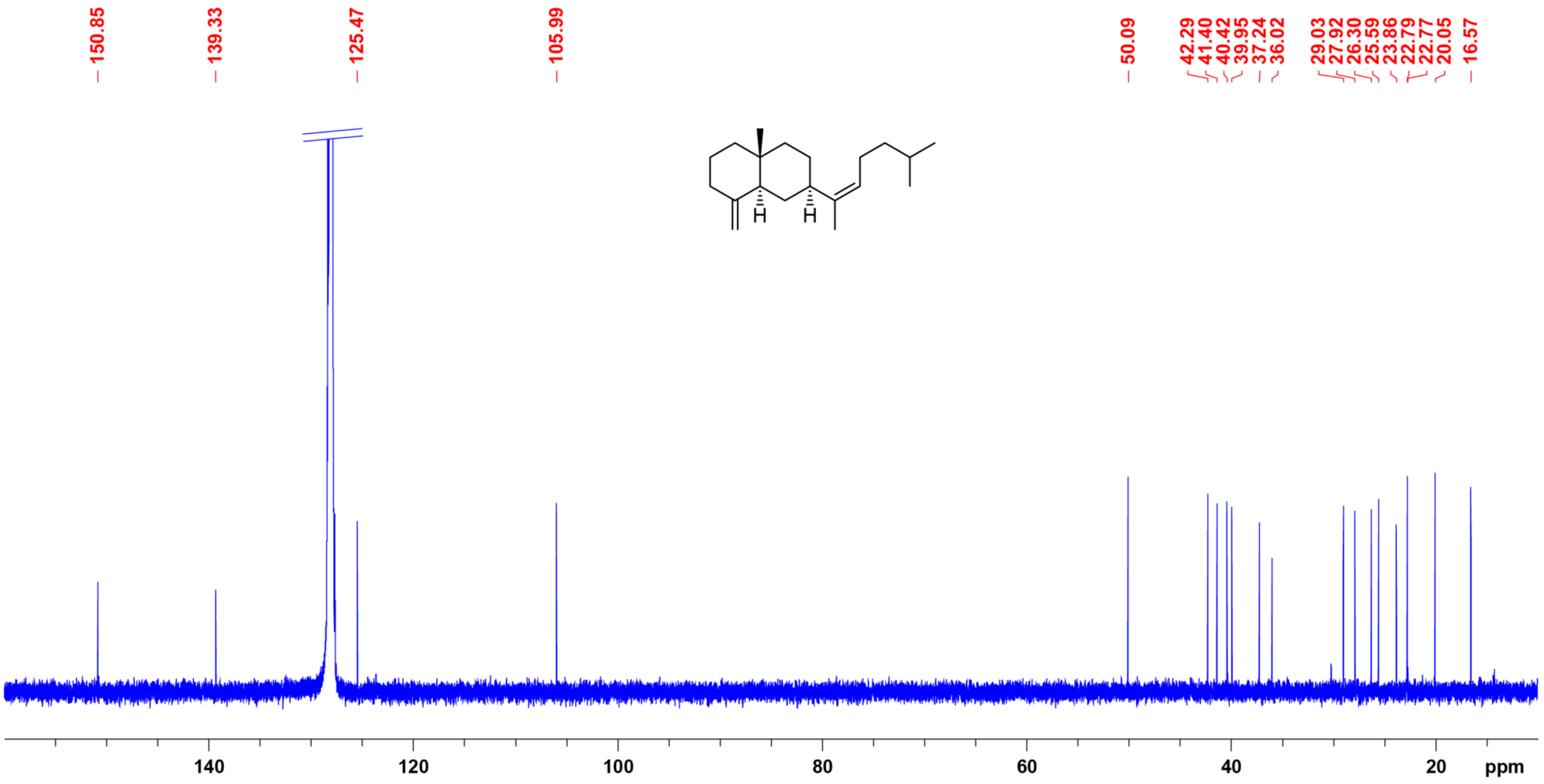

Figure S117. ^{13}C-NMR spectrum of **13** (176 MHz, C_6D_6).

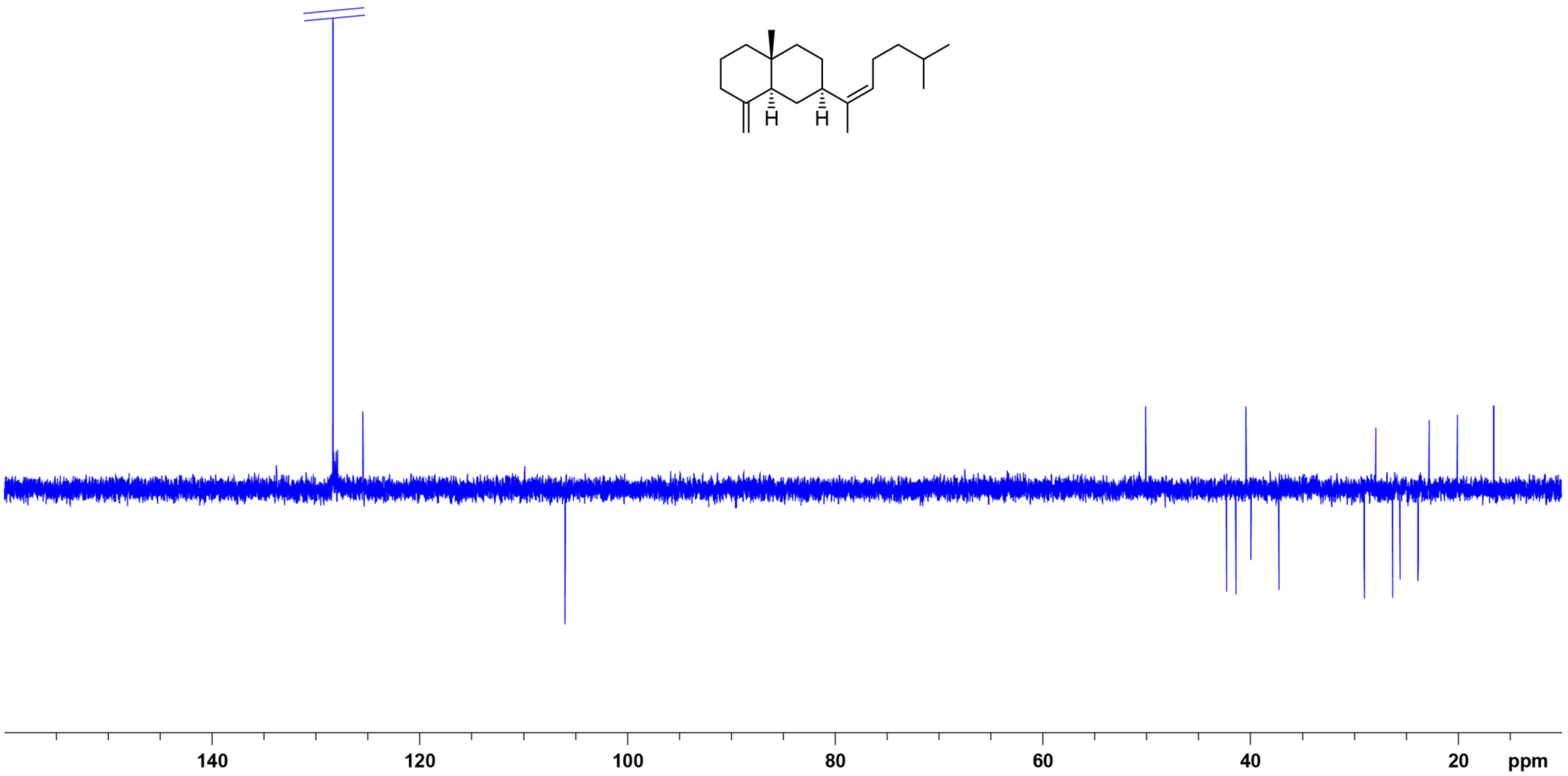

Figure S118. ^{13}C-DEPT135 spectrum of **13** (176 MHz, C_6D_6).

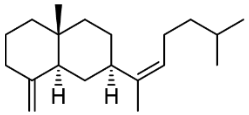

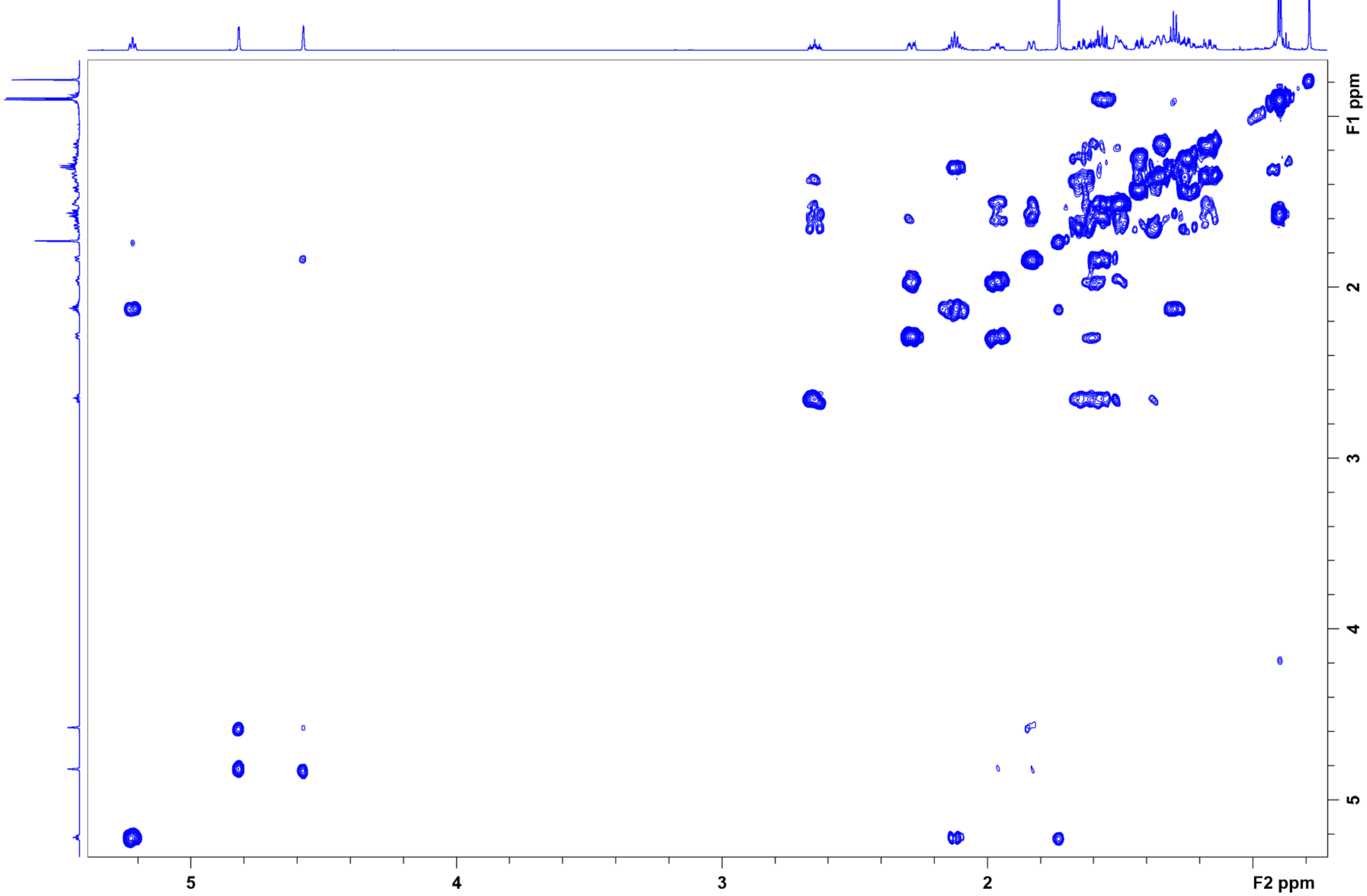

Figure S119. $^{1}H,^{1}H$-COSY spectrum (C_6D_6) of **13**.

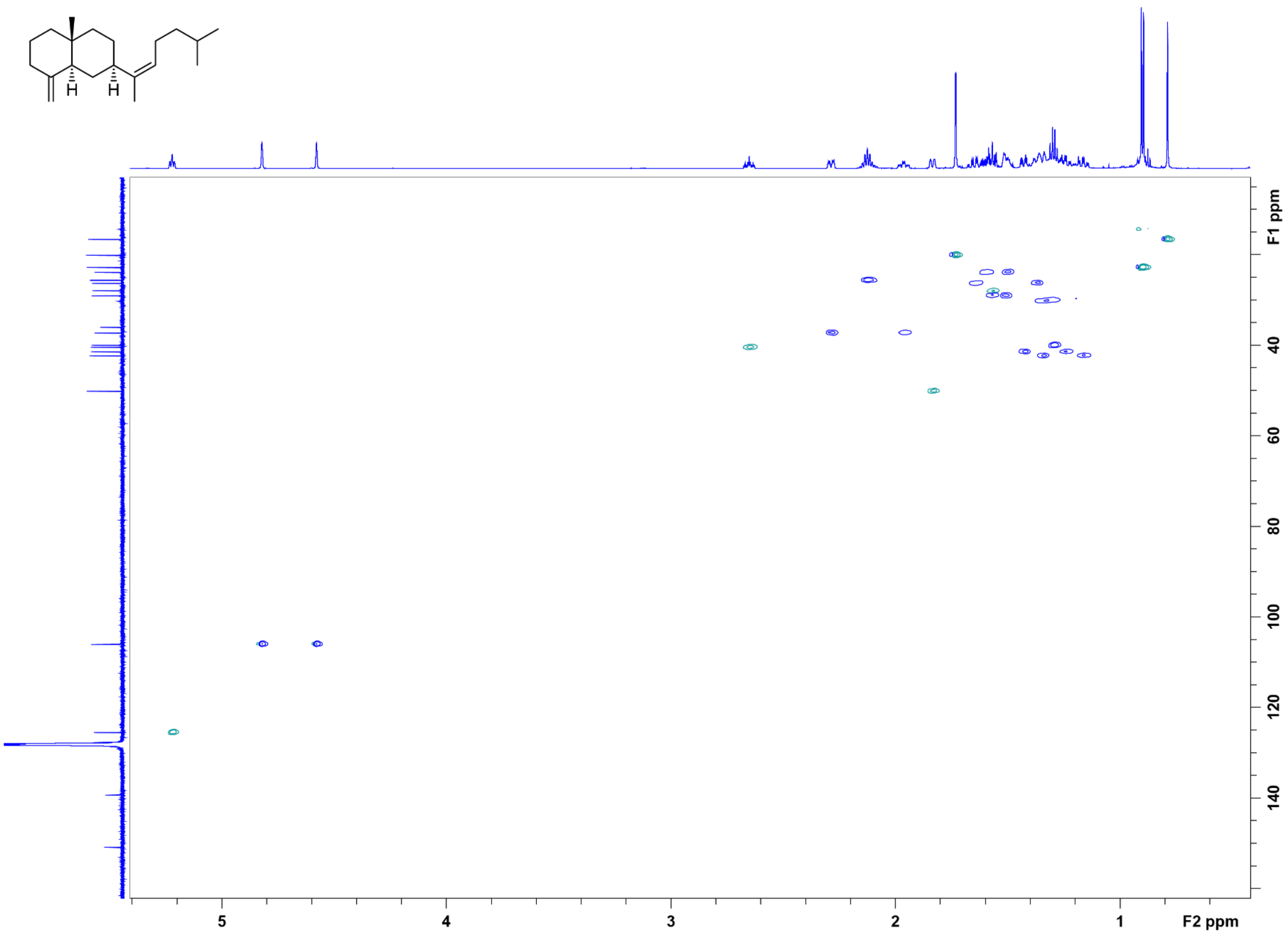

Figure S120. HSQC spectrum (C_6D_6) of **13**.

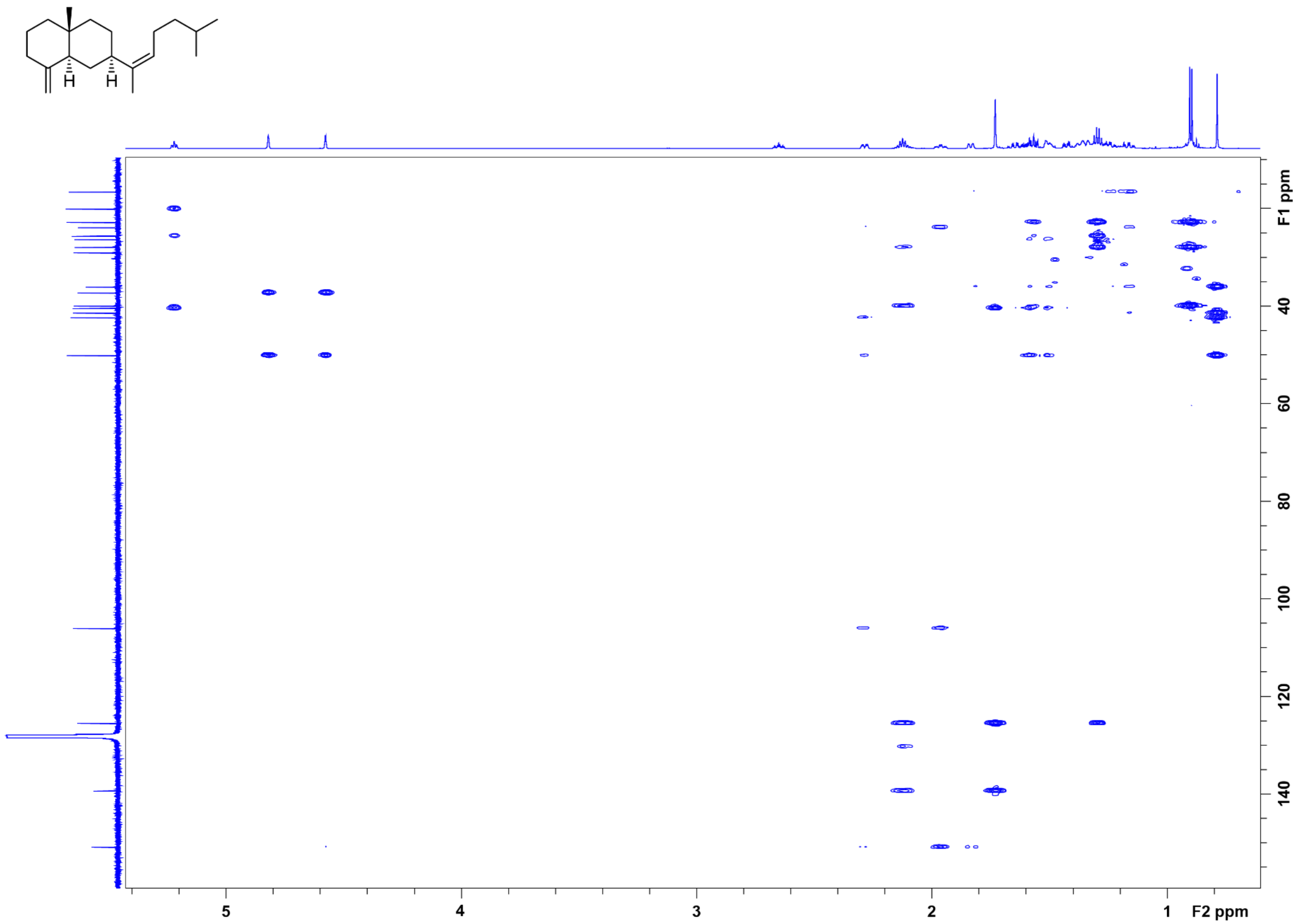

Figure S121. HMBC spectrum (C_6D_6) of **13**.

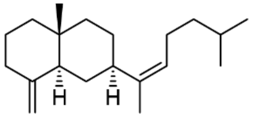

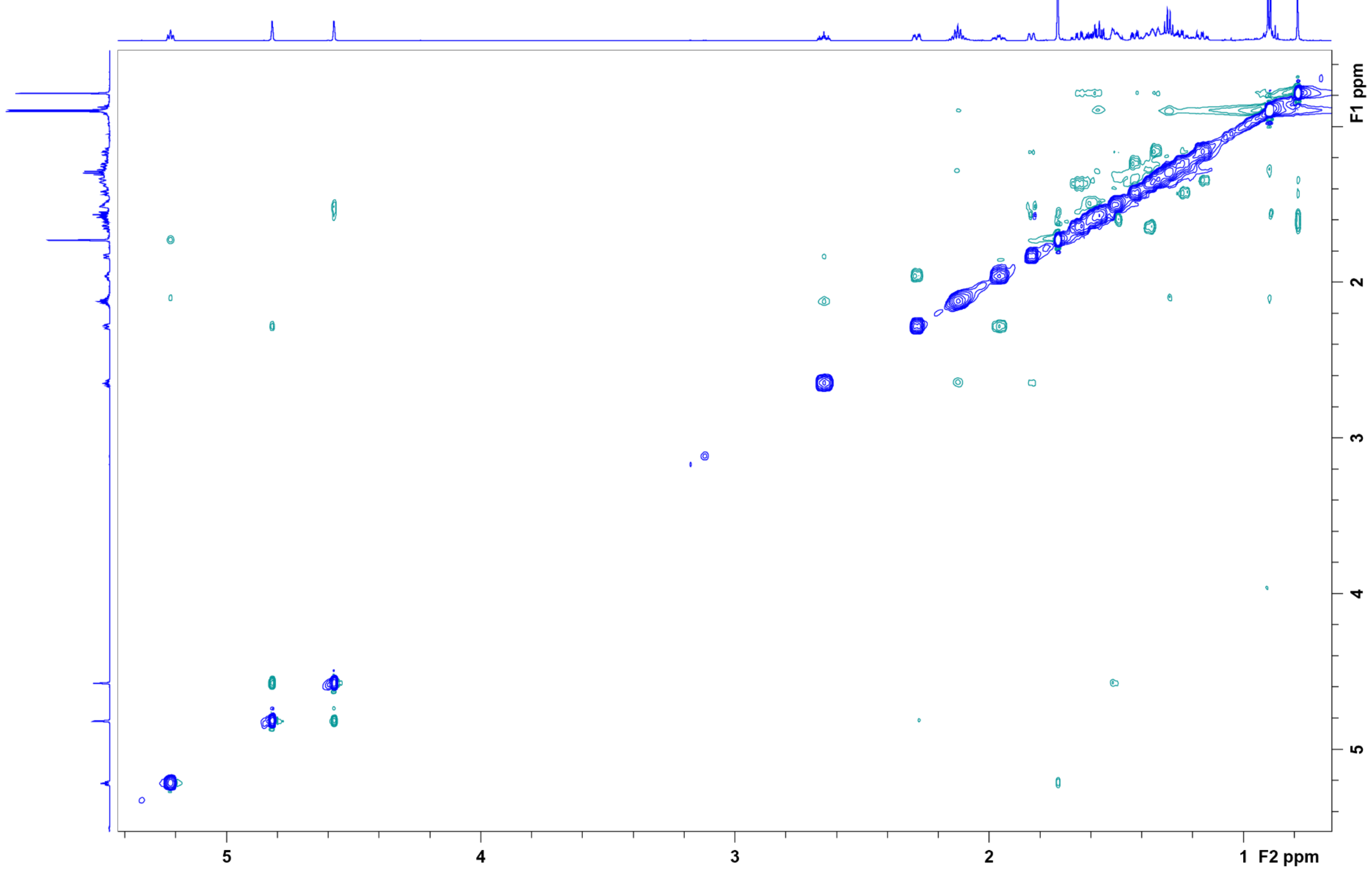

Figure S122. NOESY spectrum (C_6D_6) of **13**.

Synthesis of 20-*nor*-GGPP

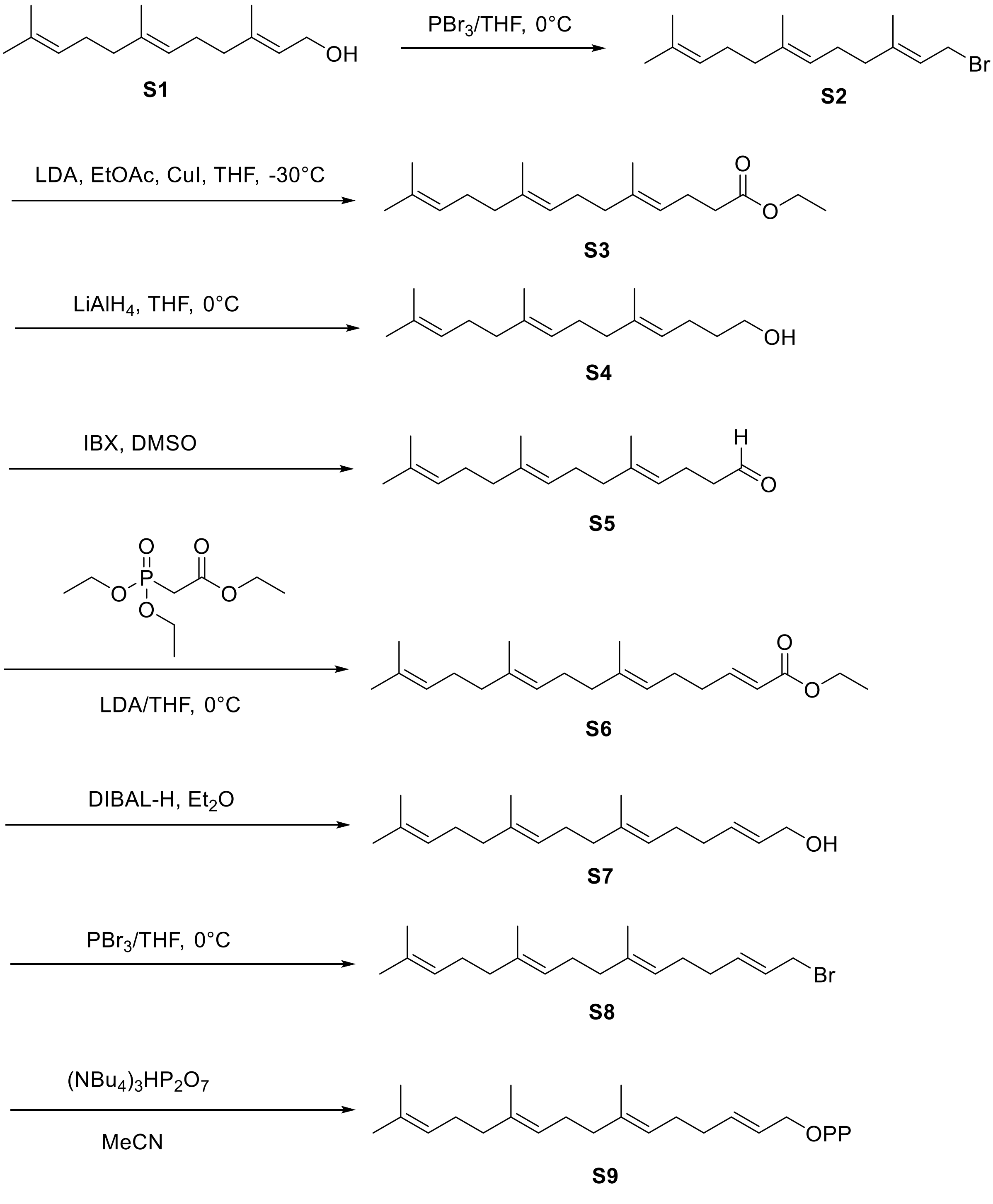

Scheme S1. Synthesis of 20-*nor*-GGPP.

Synthesis of ethyl (4*E*,8*E*)-5,9,13-trimethyltetradeca-4,8,12-trienoate (S3)

Farnesol **S1** (2.49 mL, 10 mmol, 1.0 eq) was dissolved in dry THF (30 mL) and the solution was cooled to 0 °C. PBr_3 (0.38 mL, 4 mmol, 1.2 eq.) was added dropwise. The reaction mixture was stirred for 1 h at 0 °C and then poured onto ice-water. The aqueous phase was extracted three times with ethyl acetate. The combined organic layers were dried with $MgSO_4$ and concentrated under reduced pressure. Farnesyl bromide **S2** was obtained as pale-yellow oil and immediately used in the next step without purification.

To a cooled solution (0°C) of diisopropylamine (2.89 mL, 20.5 mmol, 2.05 eq.) in dry THF (25 mL) was added *n*-BuLi (1.6 M in hexane, 12.8 mL, 20.5 mmol, 2.05 eq.). The reaction mixture was stirred for 1 h at 0 °C. In a second flask CuI (7.62 g, 40 mmol, 4.0 eq.) and ethyl acetate (2.00 mL, 20.5 mmol, 2.05 eq.) were dissolved in dry THF (70 mL) and the mixture was cooled to –110 °C. The freshly prepared LDA was added and the reaction mixture was allowed to warm to –50 °C and further stirred for 1.5 h. The freshly prepared farnesyl bromide **S2** in dry THF (10 mL) was added dropwise and the reaction mixture was allowed to warm to –30 °C and further stirred overnight at –30 °C. The reaction mixture was then hydrolysed by the addition of a saturated aqueous NH_4Cl solution, followed by extraction with ethyl acetate (3 x 70 mL). The combined organic layers were dried with $MgSO_4$ and concentrated under reduced pressure. The crude product was purified by column chromatography on silica gel with cyclohexane/ethyl acetate (25:1) yielding the ester **S3** (1.68 g, 5.76 mmol, 58%) as a colourless oil.

TLC (cyclohexane/ ethyl acetate, 20:1): R_f = 0.6. GC (HP5-MS): *I* = 2019. ^{1}H-NMR (500 MHz, $CDCl_3$): δ = 5.09 (m, 3H), 4.12 (qd, $^3J_{H,H}$ = 7.1 Hz, 2H), 2.30 (br s, 2H), 2.31 (br s, 2H), 2.06 (m, 2H), 2.10-2.01 (m, 4H), 2.00-1.94 (m, 4H), 1.68 (d, $^4J_{H,H}$ = 1.3 Hz, 3H), 1.62 (d, $^4J_{H,H}$ = 1.5 Hz, 3H), 1.60 (s, 3H), 1.59 (s, 3H) ppm. ^{13}C-NMR (125 MHz, $CDCl_3$): δ = 173.60 (C_q), 136.77 (C_q), 135.19 (C_q), 131.42 (C_q), 124.52 (CH), 124.20 (CH), 122.48 (CH), 60.35 (CH_2), 39.85 (CH_2), 39.79 (CH_2), 34.70 (CH_2), 26.90 (CH_2), 26.69 (CH_2), 25.84 (CH_3), 23.72 (CH_2), 17.82 (CH_3), 16.15 (CH_3), 16.14 (CH_3), 14.40 (CH_3) ppm. MS (EI, 70 eV): *m/z* (%) = 292 (4), 277 (1), 249 (5), 236 (2), 223 (6), 208 (11), 191 (2), 181 (7), 177 (14), 163 (5), 155 (10), 149 (13), 135 (40), 121 (27), 113 (22), 109 (37), 95 (27), 93 (38), 81 (100), 69 (99), 67 (52), 55 (24), 41 (84). HRMS (APCI): *m/z* = 293.2476 (calc. for $[C_{19}H_{33}O_2]^+$ 293.2475).

Synthesis of (4*E*,8*E*)-5,9,13-trimethyltetradeca-4,8,12-trien-1-ol (S4)

To a stirred suspension of $LiAlH_4$ (2.4 eq.) in dry THF (1 mL $mmol^{-1}$) at 0 °C were added dropwise the solutions of the esters **S3** (1.0 eq.) in dry THF (1 mL $mmol^{-1}$). After 2 h stirring at 0 °C, the reaction mixture was allowed to warm to room temperature and stirring was continued for 1 h. The reaction mixture was hydrolysed by the dropwise addition of water (1 mL $mmol^{-1}$) and stirred for another 15 min. The precipitate was filtered off and thoroughly washed with Et_2O. The combined organic layers were dried with MgSO4 and concentrated under reduced pressure. The residue was purified by column chromatography on silica gel with cyclohexane/ethyl acetate (10:1) to give the products **S4** (1.19 g, 4.76 mmol, 83%) as a colourless oil.

TLC (cyclohexane/ ethyl acetate, 12:1): R_f = 0.5. GC (HP5-MS): *I* = 1908. ^{1}H-NMR (500 MHz, $CDCl_3$): δ = 5.15 (tt, $^3J_{H,H}$ = 7.1, $^4J_{H,H}$ = 1.3 Hz, 1H), 5.09 (m, 2H), 3.64 (t, $^3J_{H,H}$ = 6.5 Hz, 2H), 2.10-2.03 (m, 4H), 2.01-1.95 (m, 4H), 1.68 (s, 3H), 1.63 (m, 2H), 1.62 (s, 3H), 1.60 (s, 6H) ppm. ^{13}C-NMR (125 MHz, $CDCl_3$): δ = 135.99 (C_q), 135.20 (C_q), 131.42 (C_q), 124.53 (CH), 124.26 (CH), 123.89 (CH), 62.90 (CH_2), 39.86 (2x CH_2), 32.91 (CH_2), 26.90 (CH_2), 26.71 (CH_2), 25.83 (CH_3), 24.41 (CH_2), 17.82 (CH_3), 16.15 (CH_3), 16.13 (CH_3) ppm. MS (EI, 70 eV): *m/z* (%) = 250 (2), 235 (1), 219 (1), 207 (2), 194 (1), 181 (3), 166 (4), 149 (2), 136 (12), 121 (12), 107 (10), 95 (42), 81 (37), 69 (100), 55 (18), 41 (45). HRMS (APCI): *m/z* = 251.2368 (calc. for $[C_{17}H_{31}O]^+$ 251.2369).

Synthesis of (4*E*,8*E*)-5,9,13-trimethyltetradeca-4,8,12-trien-1-al (S5)

The alcohol **S4** (1.193 mg, 4.76 mmol, 1.0 eq.) was dissolved in dry DMSO (22 mL) and IBX (1.867 g, 6.67 mmol, 1.4 eq.) was added in one portion. The reaction mixture was stirred for 2 h at room temperature. The mixture was hydrolysed with saturated aqueous $NaHCO_3$ solution and extracted three times with diethyl ether (3 x 60 mL). The combined organic layers were dried with $MgSO_4$ and concentrated under reduced pressure. Column chromatography with cyclohexane/ethyl acetate (15:1) yielded the desired aldehyde **S5** (1.04 g, 4.18 mmol, 88%) as colourless oil.

TLC (cyclohexane/ ethyl acetate, 15:1): R_f = 0.4. GC (HP5-MS): *I* = 1850. ^{1}H-NMR (500 MHz, C_6D_6): δ = 9.33 (s, 1H), 5.23 (m, 2H), 5.02 (tq, $^3J_{H,H}$ = 7.2, $^4J_{H,H}$ = 1.3 Hz, 1H), 2.17 (m, 2H), 2.13-2.06 (m, 6H), 2.00 (m, 2H), 1.90 (td, $^3J_{H,H}$ = 7.4, $^4J_{H,H}$ = 1.9 Hz, 2H), 1.68 (d, $^4J_{H,H}$ = 1.4, 3H), 1.58 (d, $^4J_{H,H}$ = 1.3 Hz, 3H), 1.57 (d, $^4J_{H,H}$ = 1.3 Hz, 3H), 1.48 (d, $^4J_{H,H}$ = 1.4 Hz, 3H) ppm. ^{13}C-NMR (125 MHz, C_6D_6): δ = 204.44 (CH), 136.38 (C_q), 135.19 (C_q), 131.20 (C_q), 124.94 (CH), 124.59 (CH), 122.98 (CH), 43.90 (CH_2), 40.22 (CH_2), 40.02 (CH_2), 27.25 (CH_2), 26.93 (CH_2), 25.88 (CH_3), 21.07 (CH_2), 17.77 (CH_3), 16.13 (CH_3), 16.01 (CH_3) ppm. MS (EI, 70 eV): *m/z* (%) = 248 (2), 233 (1), 215 (1), 205 (2), 187 (1), 177 (3), 161 (4), 147 (2), 136 (24), 121 (9), 107 (7), 93 (25), 81 (41), 69 (100), 55 (26), 41 (53). HRMS (APCI): *m/z* = 249.2212 (calc. for $[C_{17}H_{29}O]^+$ 249.2213).

Synthesis of ethyl (2*E*,6*E*,10*E*)-7,11,15-trimethylhexadeca-2,6,10,14-tetraenoate S6

Diisopropylamine (0.62 mL, 4.39 mmol) was dissolved in dry THF (2 mL $mmol^{-1}$) and the solution was cooled to 0 °C. After addition of *n*-BuLi (1.6 M in THF, 2.74 mL, 4.39 mmol) the reaction mixture was stirred for 1 h at 0 °C and then cooled to -78°C. Triethyl phosphonoacetate (1 eq.) in dry THF (2 mL $mmol^{-1}$) was added, followed by the addition of the aldehyde **S5** (1 eq.) in dry THF (2 mL $mmol^{-1}$). The reaction mixture was stirred for 45 min at -78 °C and then warmed to room temperature and stirred overnight. The reaction was quenched with H_2O (4 mL $mmol^{-1}$) and extracted three times with ethyl acetate. The combined organic layers were dried over $MgSO_4$ and concentrated under reduced pressure. Purification by column chromatography on silica gel with cyclohexane/diethyl ether (60:1) yielded **S6** (0.681 g, 2.39 mmol, 57%) as a colourless oil.

TLC (cyclohexane/ ethyl acetate, 20:1): R_f = 0.6. GC (HP5-MS): *I* = 2254. ^{1}H-NMR (500 MHz, $CDCl_3$): δ = 6.96 (dt, $^3J_{H,H}$ = 15.7, $^3J_{H,H}$ = 6.8 Hz, 1H), 5.82 (dt, $^3J_{H,H}$ = 15.7, $^4J_{H,H}$ = 1.5 Hz, 1H), 5.11 (m, 3H), 4.18 (q, $^3J_{H,H}$ = 7.1 Hz, 2H), 2.10-2.03 (m, 4H), 2.02-1.95 (m, 4H), 1.68 (d, $^3J_{H,H}$ = 1.4, 3H), 1.60 (s, 9H), 1.28 (t, $^3J_{H,H}$ = 7.1 Hz, 3H) ppm. ^{13}C-NMR (125 MHz, $CDCl_3$): δ = 166.89 (C_q), 149.09 (CH), 136.56 (C_q), 135.22 (C_q), 131.42 (C_q), 124.53 (CH), 124.23 (CH), 122.91 (CH), 121.56 (CH), 60.27 (CH_2), 39.86 (CH_2), 39.81 (CH_2), 32.62 (CH_2), 26.91 (CH_2), 26.73 (CH_2), 26.65 (CH_2), 25.84 (CH_3), 17.83 (CH_3), 16.23 (CH_3), 16.15 (CH_3), 14.43 (CH_3) ppm. MS (EI, 70 eV): *m/z* (%) = 318 (2), 303 (1), 275 (4), 261 (1), 245 (2), 229 (1), 205 (4), 190 (2), 175 (10), 161 (8), 149 (9), 136 (43), 121 (28), 114 (47), 107 (39), 93 (36), 81 (88), 69 (100), 55 (25), 41 (72). HRMS (APCI): *m/z* = 319.2630 (calc. for $[C_{21}H_{35}O_2]^+$ 319.2632).

Synthesis of 20-*nor*-geranylgeraniol (S7)

The ester **S6** (0.628 g, 2.39 mmol) was dissolved in dry diethyl ether (35 mL) and the solution was cooled to 0 °C. After addition of DIBAl-H (1.0 M in hexane, 5.25 mL, 5.25 mmol) the reaction mixture was stirred for 1 h at 0 °C. Saturated sodium potassium tartrate solution was then added to the reaction mixture and further stirred at room temperature for 30 min. Ethyl acetate (3 x 30 mL) was added for extraction and the combined organic layers were dried with $MgSO_4$ and concentrated under reduced pressure. The crude product was purified by column chromatography on silica gel with cyclohexane/ethyl acetate (5:1) yielding the alcohol **S7** (0.54 g, 1.94 mmol, 81%) as a colourless oil.

TLC (cyclohexane/ ethyl acetate, 7:3): R_f = 0.6. GC (HP5-MS): *I* = 2128. ^{1}H-NMR (500 MHz, $CDCl_3$): δ = 5.73-5.63 (m, 2H), 5.15-5.09 (m, 3H), 4.09 (br d, $^3J_{H,H}$ = 5.4, 2H), 2.10-2.04 (m,

8H), 2.01-1.96 (m, 4H), 1.68 (d, $^4J_{H,H}$ = 1.5, 3H), 1.60 (d, $^4J_{H,H}$ = 1.5, 9H) ppm. ^{13}C-NMR (125 MHz, $CDCl_3$): δ = 135.76 (C_q), 135.14 (C_q), 133.28 (CH), 131.44 (C_q), 129.21 (CH), 124.54 (CH), 124.33 (CH), 123.79 (CH), 64.01 (CH_2), 39.88 (CH_2), 39.85 (CH_2), 32.60 (CH_2), 27.75 (CH_2), 26.92 (CH_2), 26.77 (CH_2), 25.84 (CH_3), 17.83 (CH_3), 16.22 (CH_3), 16.16 (CH_3) ppm. MS (EI, 70 eV): *m/z* (%) = 207 (11), 191 (3), 175 (3), 161 (3), 147 (6), 136 (19), 121 (17), 107 (19), 93 (28), 81 (61), 69 (100), 55 (22), 41 (61). APCI-(+)-HR-MS: calc. $[C_{19}H_{33}O]^+$ *m/z* = 277.2525; found: *m/z* = 277.2526.

Synthesis of trisammonium 20-*nor*-GGPP (S9)

The alcohol (*E*)-**S7** (0.54 g, 1.94 mmol) was dissolved in dry THF (15 mL) and the solutions were cooled to 0 °C. PBr_3 (0.07 mL, 0.78 mmol) was added dropwise. The reaction mixture was stirred for 1 h at 0 °C and then poured onto ice-water. The aqueous phase was extracted three times with petrol ether. The combined organic layers were dried with $MgSO_4$ and concentrated under reduced pressure. The bromide **S8** obtained without purification was immediately dissolved in dry MeCN (5 mL). After addition of $(n\text{-}Bu_4N)_3P_2O_7H$ (2.1 g, 2.3 mmol) the reaction mixture was stirred over night at room temperature and then concentrated under reduced pressure. The residue was loaded onto an ion exchange resin column (DOWEX® 50W-X8, 100-200 mesh, NH_4^+ form). The product was eluted with two column volumes of freshly prepared ion exchange buffer (0.025 M NH_4HCO_3 in 2% iPrOH/H_2O) followed by lyophilisation of the eluate to yield **S9**.

^{1}H-NMR (500 MHz, D_2O): δ = 5.81-5.52 (m, 3H), 5.09-5.07 (m, 2H), 4.33 (m, 2H), 2.04-1.95 (m, 8H), 1.93-1.87 (m, 4H), 1.89 (d, br s 3H), 1.52 (d, br s, 9H) ppm. ^{13}C-NMR (125 MHz, D_2O): δ = 135.27 (C_q), 134.77 (C_q), 134.45 (CH), 134.36 (C_q), 130.62 (CH), 126.21 (CH), 124.49 (CH), 124.14 (CH), 66.43 (CH_2), 39.82 (CH_2), 39.74 (CH_2), 32.48 (CH_2), 27.56 (CH_2), 26.79 (CH_2), 25.42 (CH_2), 23.32 (CH_3), 19.30 (CH_3), 17.40 (CH_3), 15.78 (CH_3) ppm. ^{31}P-NMR (D_2O, 203 MHz): –6.17 (d, $^2J_{P,P}$ = 20.5 Hz), –10.16 (d, $^2J_{P,P}$ = 20.5 Hz) ppm. ESI-(–)-HR-MS: calc. $[C_{19}H_{33}O_7P_2]^-$ *m/z* = 435.1712; found: *m/z* = 435.1707.

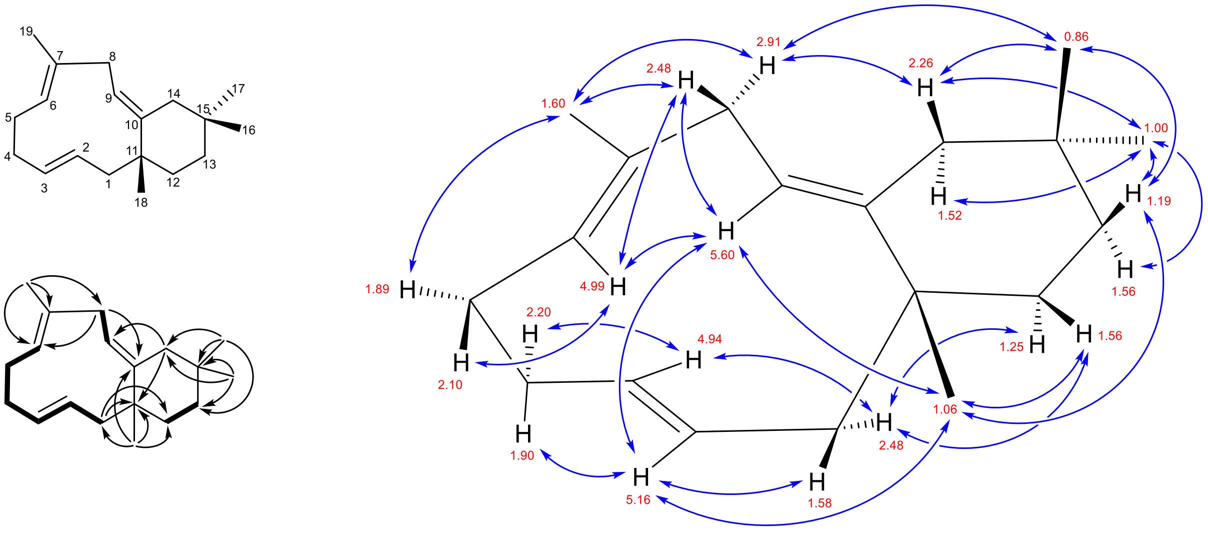

Figure S123. Structure elucidation of 20-*nor*-chryseojoostene A (**1a**). Bold: $^{1}H,^{1}H$-COSY correlations, single headed arrows: HMBC correlations, and blue double headed arrows: NOESY correlations. Carbon numbering follows 20-*nor*-GGPP numbering to indicate the origin of each carbon.

Table S19. NMR data of 20-*nor*-chryseojoostene A (**1a**) in C_6D_6 recorded at 298 K.

C[a]	type	^{13}C[b]	^{1}H[b]
1	CH_2	41.28	2.48 (m)
			1.58 (m)
2	CH	129.56	5.16 (dddd, *J* = 14.9, 11.4, 3.5, 1.4)
3	CH	129.20	4.94 (m)
4	CH_2	33.18	2.20 (m)
			1.90 (m)
5	CH_2	25.53	2.10 (m)
			1.89 (m)
6	CH	126.86	4.99 (br t, *J* = 7.8)
7	C	138.56	–
8	CH_2	35.66	2.91 (dd, *J* = 13.2, 10.0)
			2.48 (m)
9	CH	125.66	5.60 (ddd, *J* = 9.8, 6.6, 2.1)
10	C	140.62	–
11	C	39.80	–
12	CH_2	36.62	1.56 (m)
			1.25 (m)
13	CH_2	35.36	1.56 (m)
			1.19 (m)
14	CH_2	40.03	2.26 (dd, *J* = 13.9, 2.5)
			1.52 (m)
15	C	32.03	–
16	CH_3	25.09	0.86 (s)
17	CH_3	32.90	1.00 (s)
18	CH_3	25.84	1.06 (s)
19	CH_3	18.78	1.60 (br s)

[a] Carbon numbering as shown in Figure S123 indicates the origin of each carbon from 20-*nor*-GGPP by same number. [b] Chemical shifts δ in ppm, multiplicity: s = singlet, d = doublet, t = triplet, m = multiplet, br = broad, coupling constants *J* are given in Hertz.

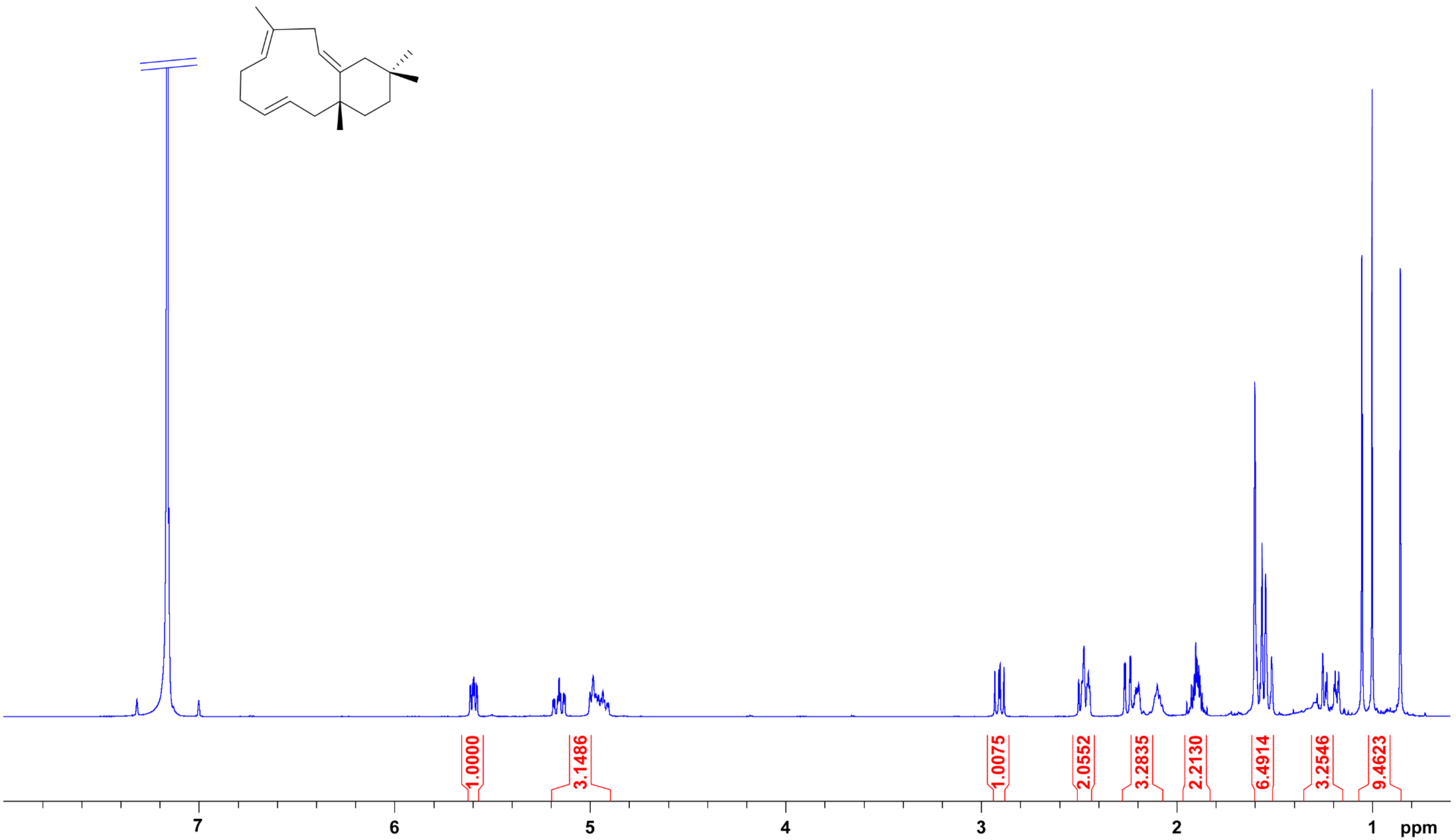

Figure S124. ^{1}H-NMR spectrum of **1a** (700 MHz, C_6D_6).

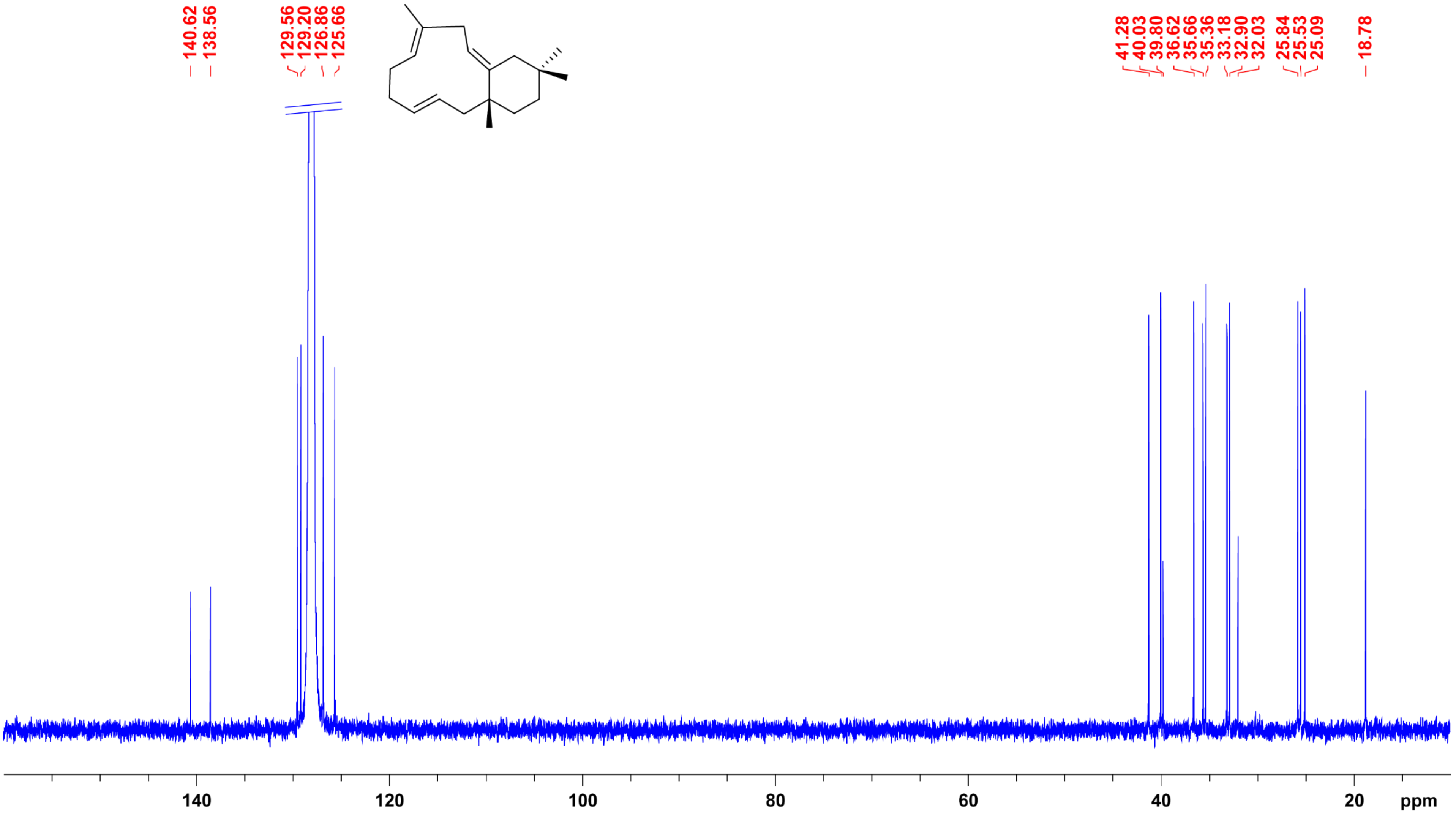

Figure S125. ^{13}C-NMR spectrum of **1a** (176 MHz, C_6D_6).

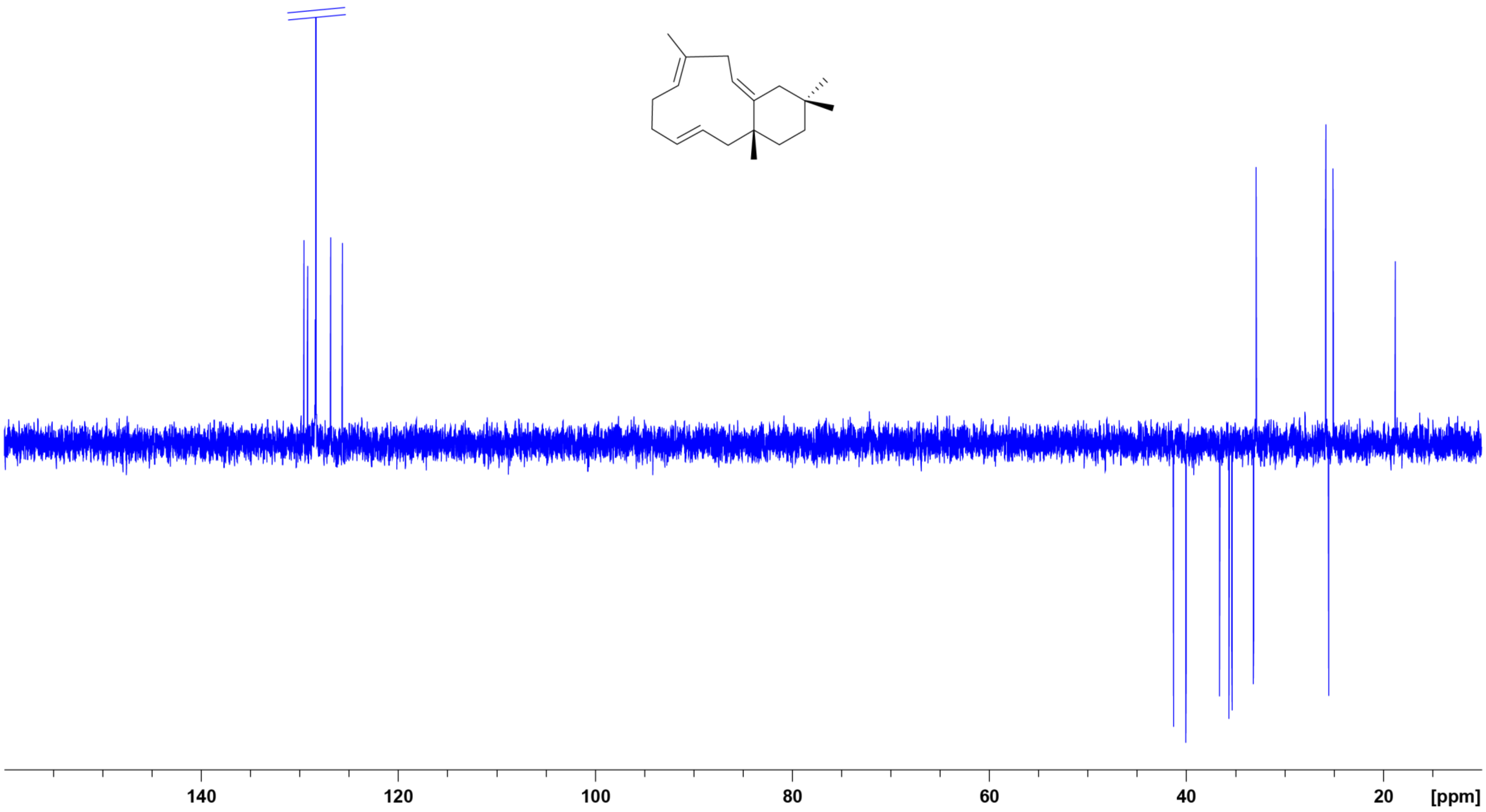

Figure S126. ^{13}C-DEPT135 spectrum of **1a** (176 MHz, C_6D_6).

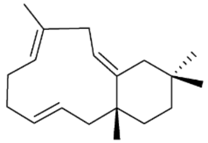

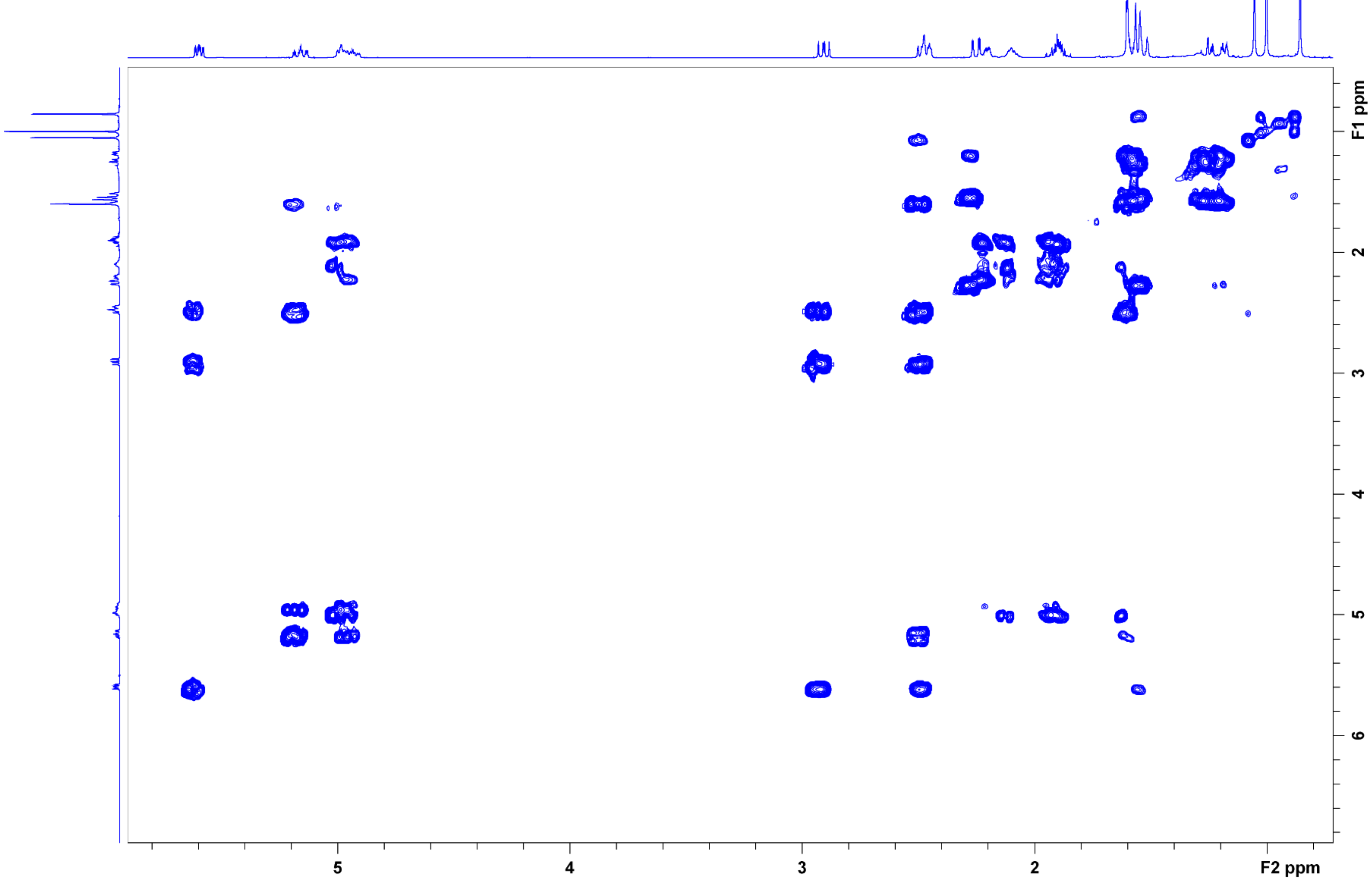

Figure S127. ^{1}H,^{1}H-COSY spectrum (C_6D_6) of **1a**.

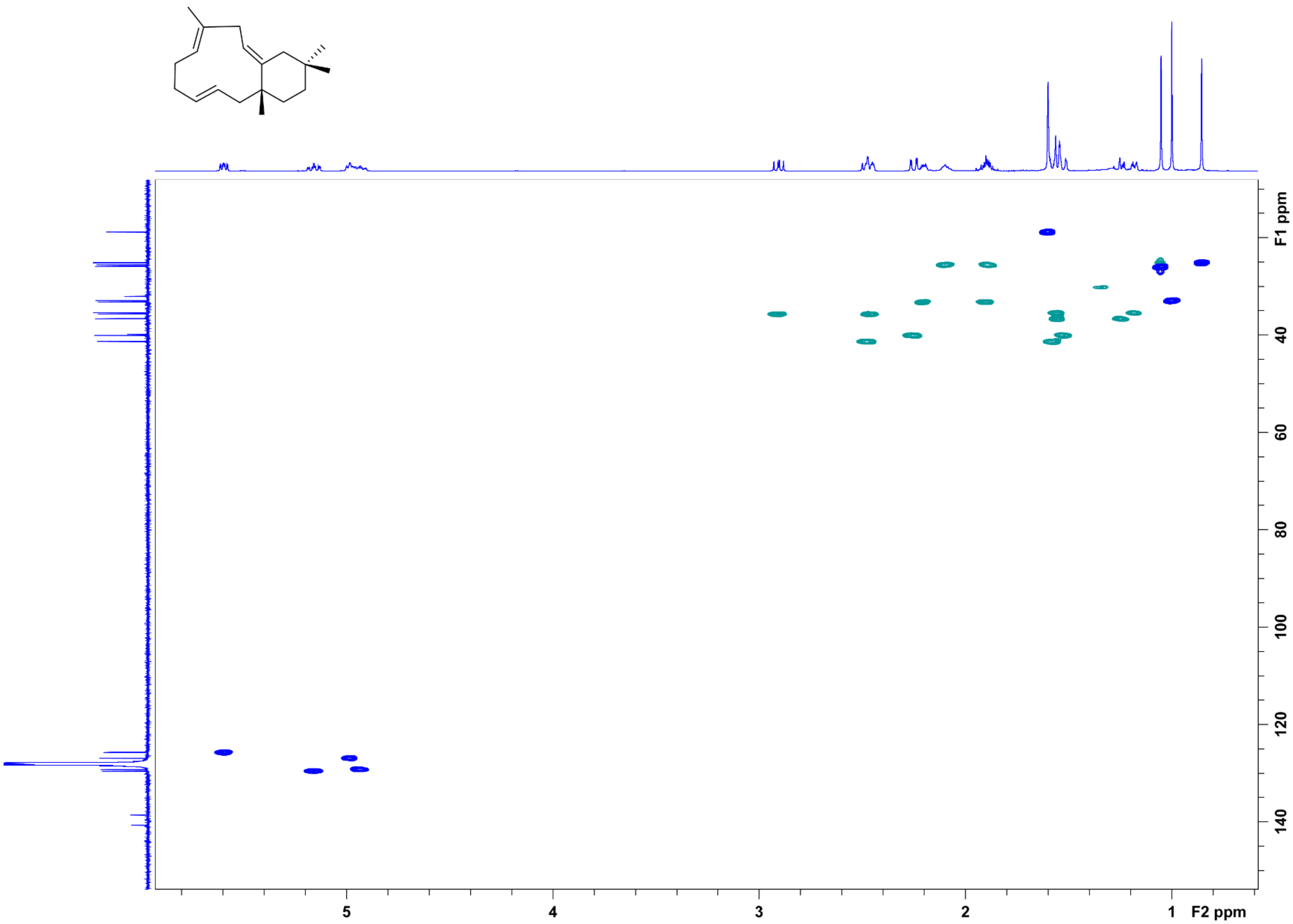

Figure S128. HSQC spectrum (C_6D_6) of **1a**.

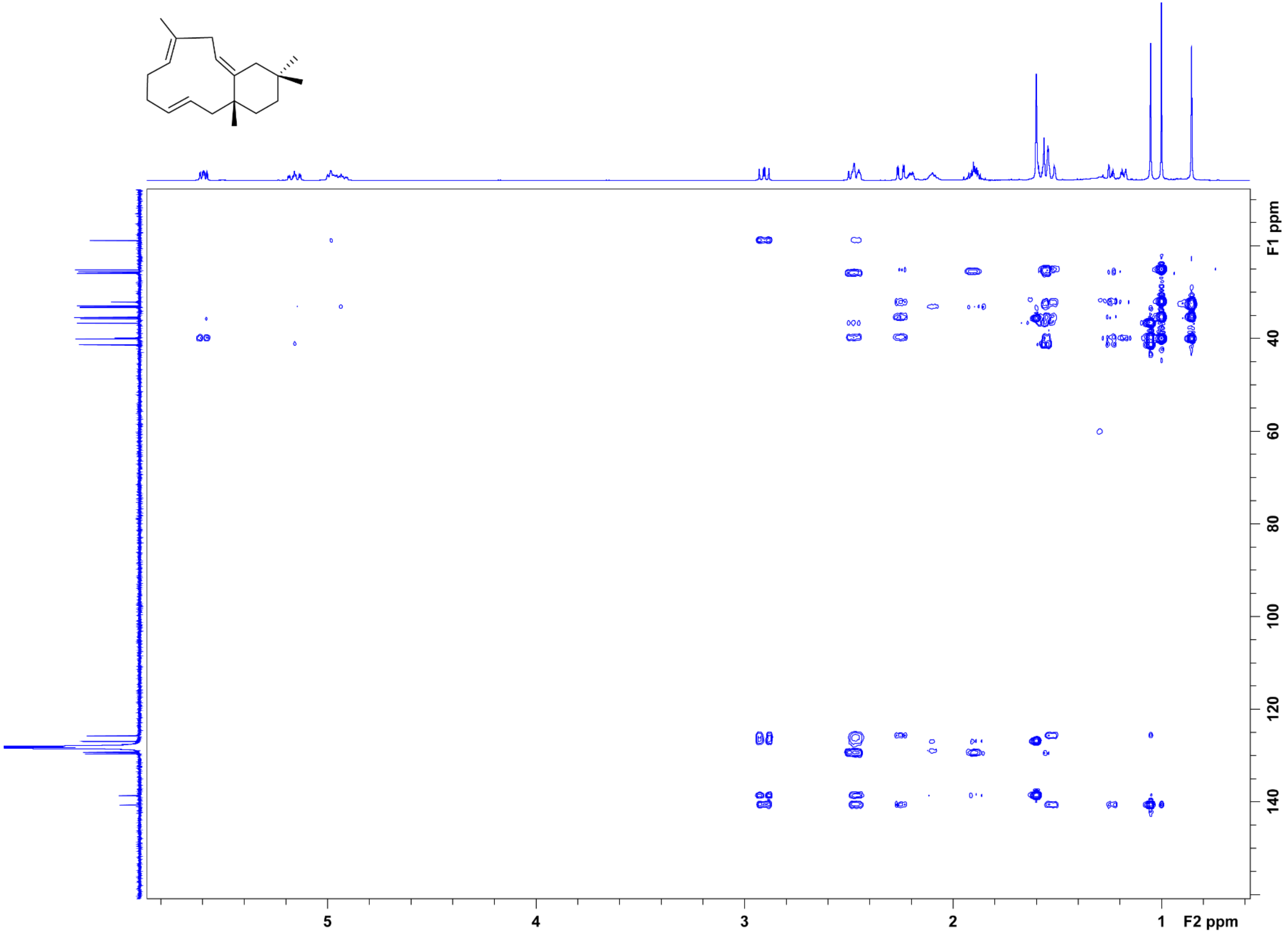

Figure S129. HMBC spectrum (C_6D_6) of **1a**.

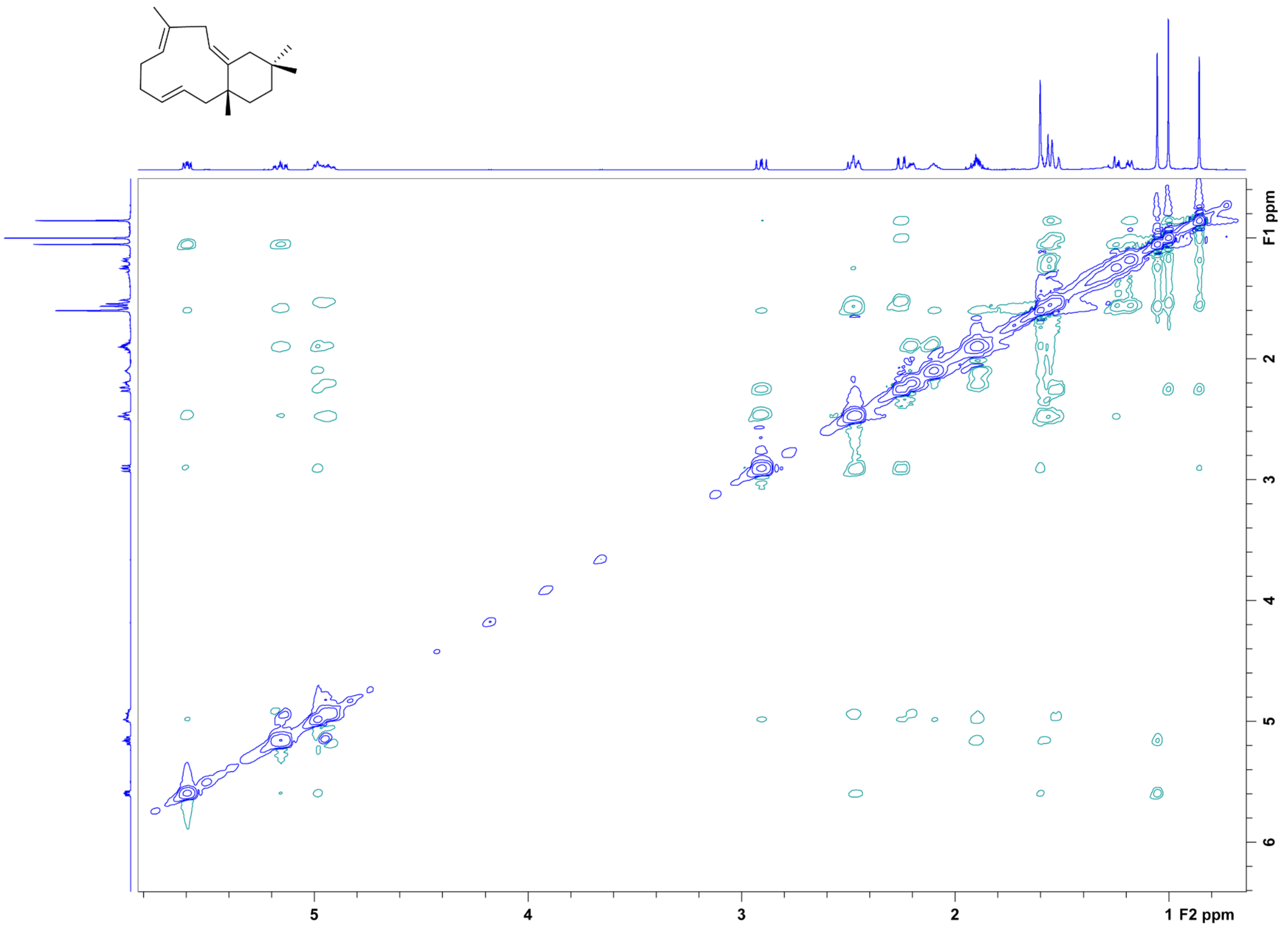

Figure S130. NOESY spectrum (C_6D_6) of **1a**.

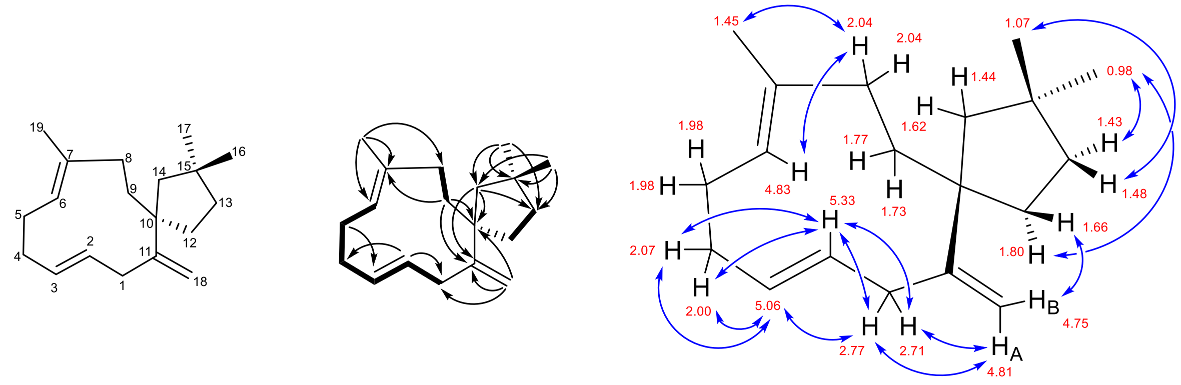

Figure S131. Structure elucidation of 20-*nor*-chryseojoostene C (**3a**). Bold: $^{1}H,^{1}H$-COSY correlations, single headed arrows: HMBC correlations, and blue double headed arrows: NOESY correlations. Carbon numbering follows 20-*nor*-GGPP numbering to indicate the origin of each carbon.

Table S20. NMR data of 20-*nor*-chryseojoostene C (**3a**) in C_6D_6 recorded at 298 K.

C[a]	type	^{13}C[b]	^{1}H[b]
1	CH_2	38.75	2.77 (dd, *J* = 13.2, 7.5)
			2.71 (dd, *J* = 13.2, 7.9)
2	CH	132.97	5.34 (ddd, *J* = 15.3, 7.6, 7.5)
3	CH	128.41	5.06 (ddd, *J* = 13.5, 7.6, 7.5)
4	CH_2	32.91	2.07 (m)
			2.00 (m)
5	CH_2	26.24	1.98 (m, 2H)
6	CH	126.97	4.83 (t, *J* = 7.6)
7	C	135.93*	–
8	CH_2	35.62*	2.04 (m, 2H)
9	CH_2	38.95*	1.77 (ddd, *J* = 14.4, 6.3, 6.2)
			1.73 (ddd, *J* = 14.4, 6.3, 6.2)
10	C	52.29	–
11	C	156.90	–
12	CH_2	36.44*	1.80 (ddd, *J* = 12.5, 10.7, 7.2)
			1.66 (ddd, *J* = 12.5, 6.5, 4.3)
13	CH_2	40.44	1.48 (ddd, *J* = 12.5, 10.8, 6.4)
			1.43 (ddd, *J* = 12.4, 6.8, 3.5)
14	CH_2	54.73*	1.62 (d, *J* = 13.4)
			1.44 (d, *J* = 13.4)
15	C	37.94	–
16	CH_3	31.82	1.07 (s)
17	CH_3	31.85	0.98 (s)
18	CH_2	110.62	4.81 (br s)
			4.75 (br s)
19	CH_3	17.82*	1.45 (br s)

[a] Carbon numbering as shown in Figure S131 indicates the origin of each carbon from 20-*nor*-GGPP by same number. [b] Chemical shifts δ in ppm, multiplicity: s = singlet, d = doublet, t = triplet, m = multiplet, br = broad, coupling constants *J* are given in Hertz. Asterisks indicate carbons that show peak broadening in the ^{13}C-NMR.

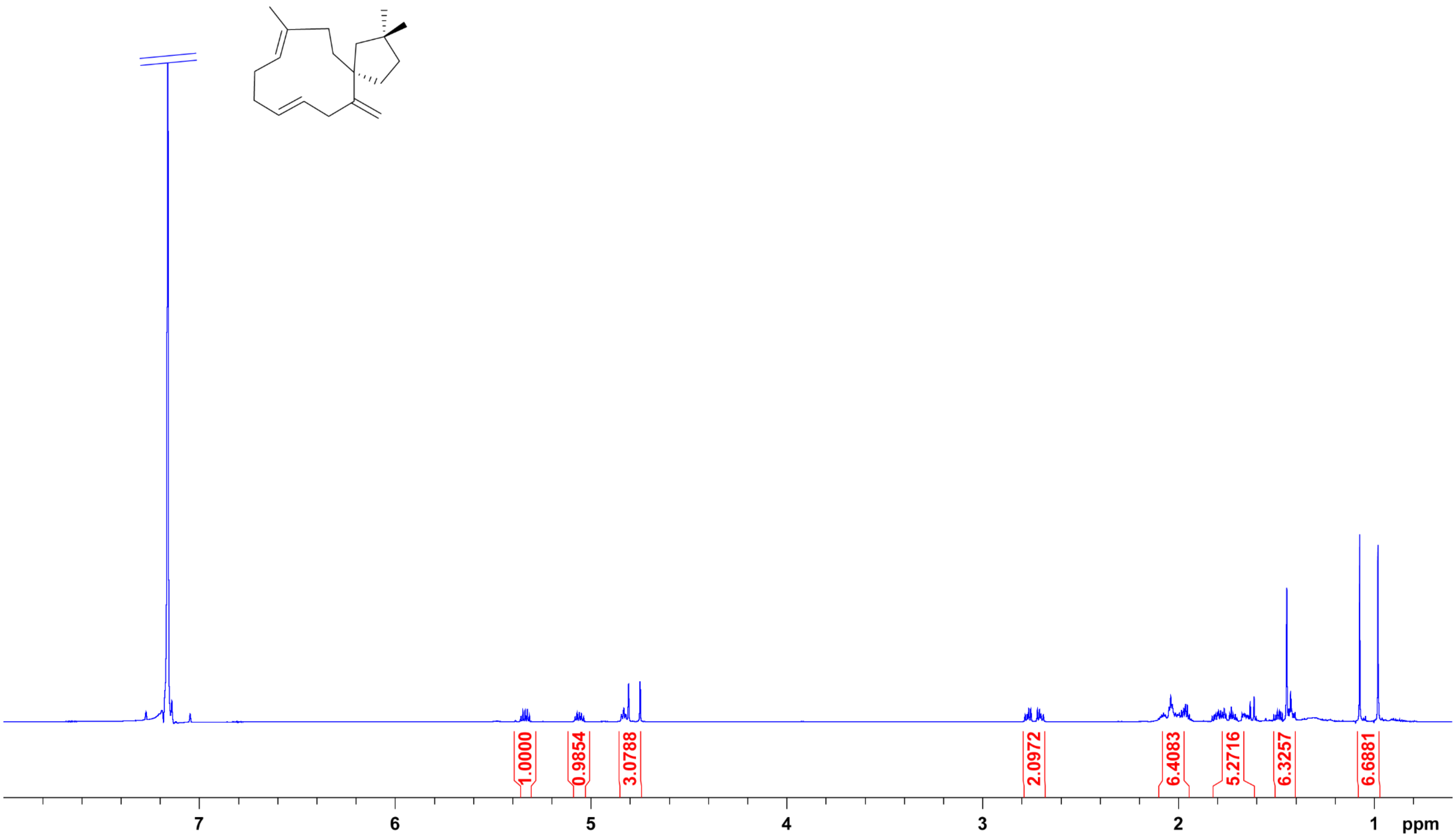

Figure S132. ^{1}H-NMR spectrum of **3a** (500 MHz, C_6D_6).

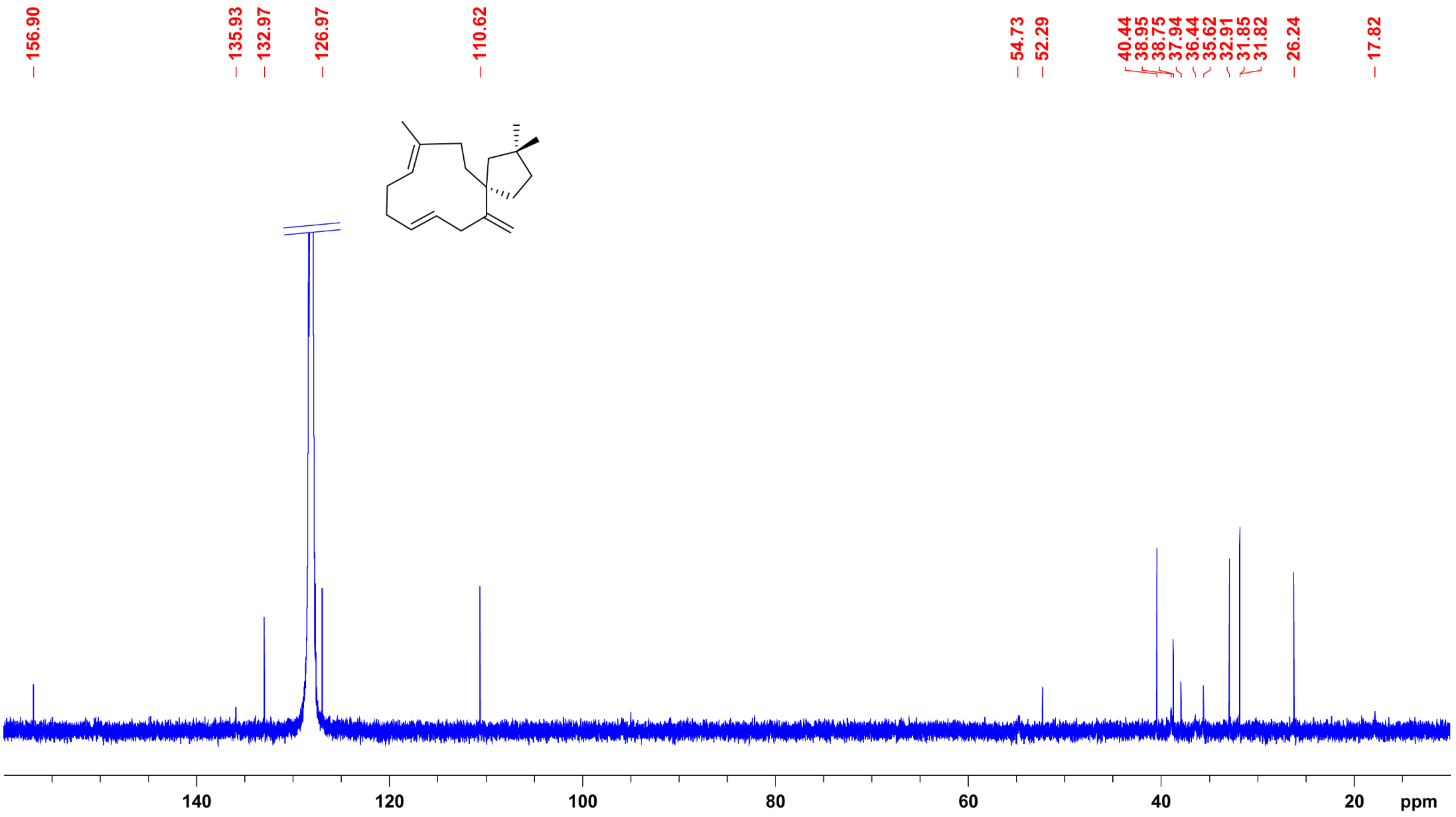

Figure S133. ^{13}C-NMR spectrum of **3a** (125 MHz, C_6D_6).

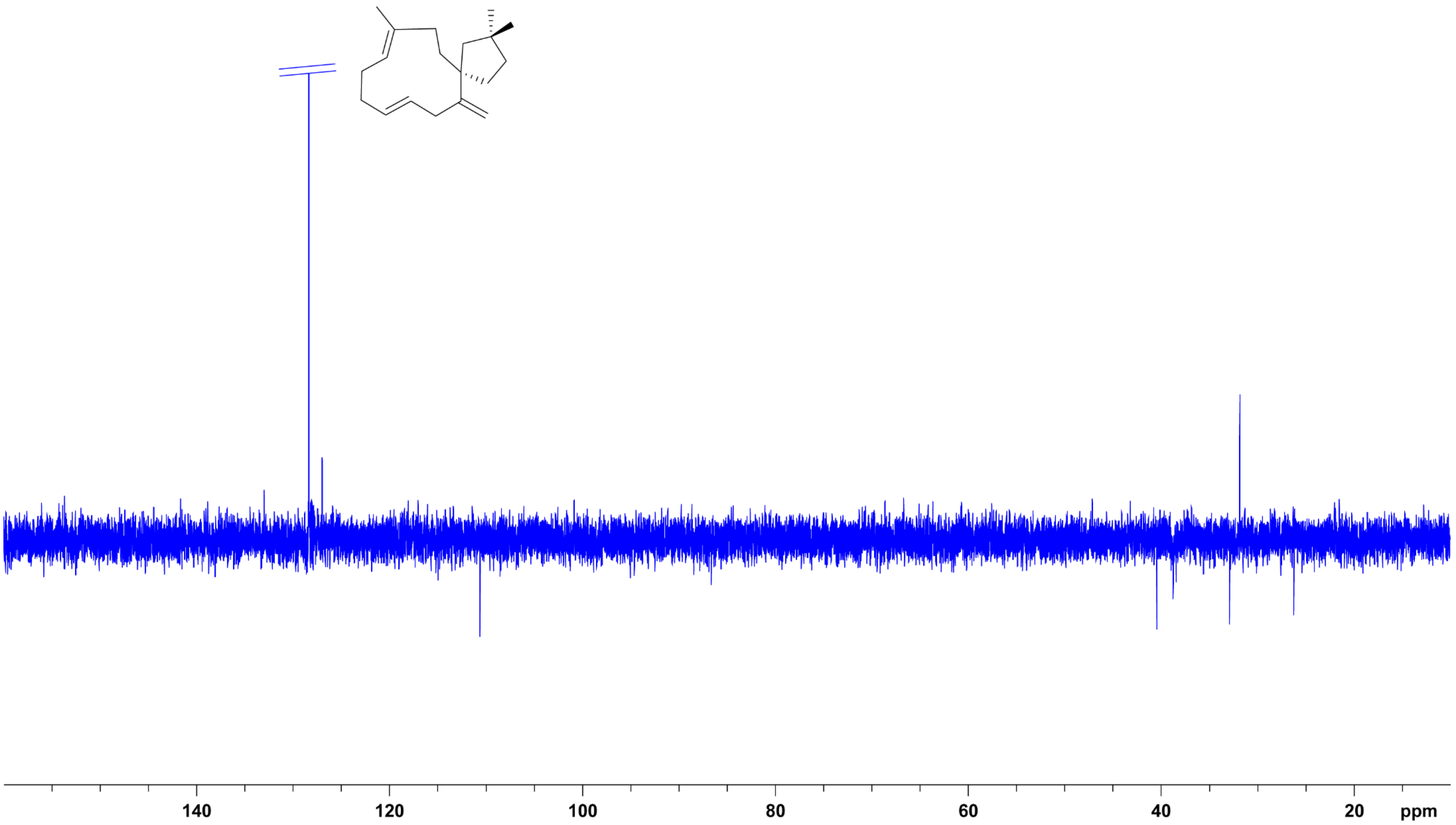

Figure S134. ^{13}C-DEPT135 spectrum of **3a** (125 MHz, C_6D_6).

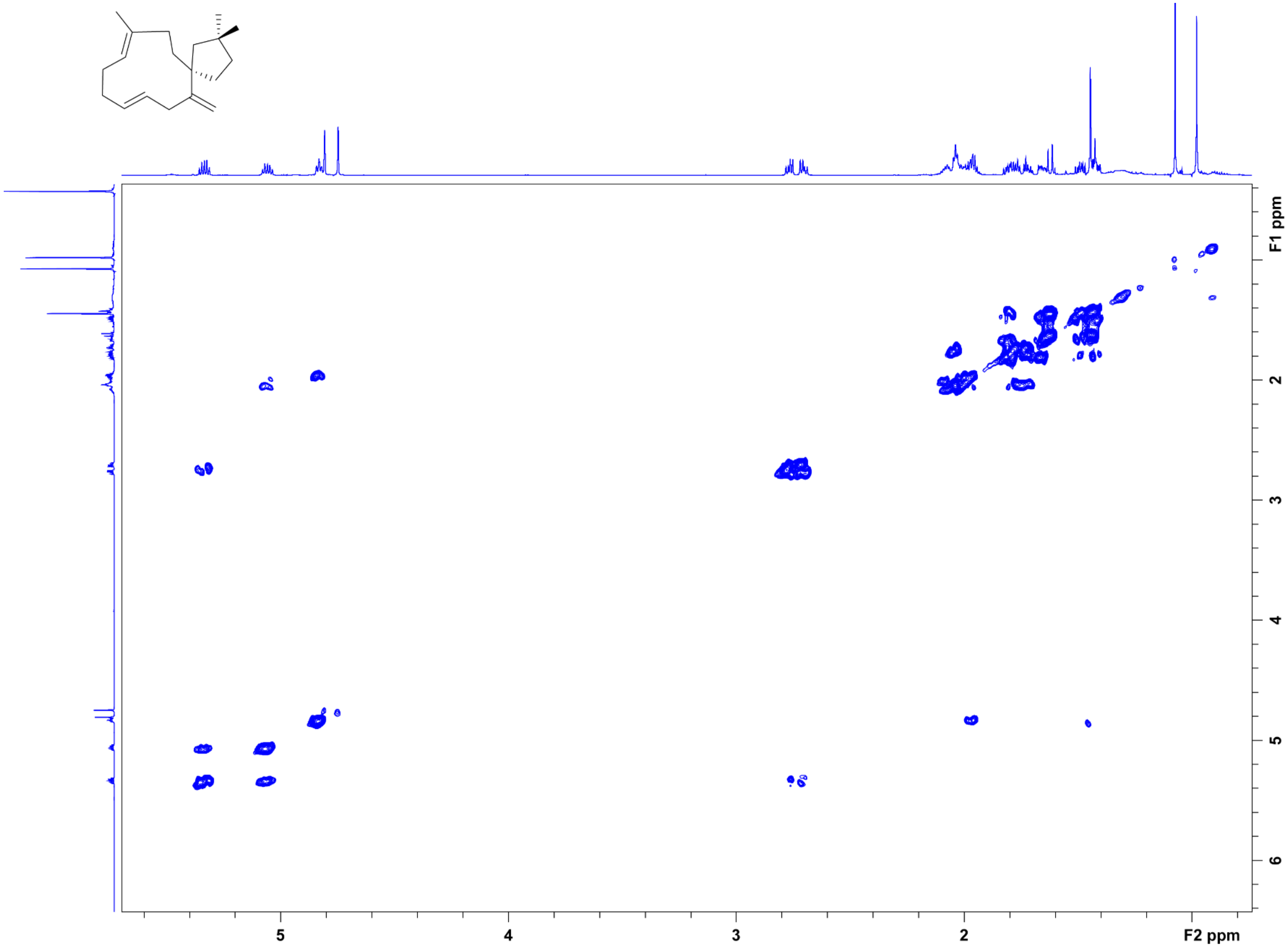

Figure S135. ${}^{1}H,{}^{1}H$-COSY spectrum (C_6D_6) of **3a**.

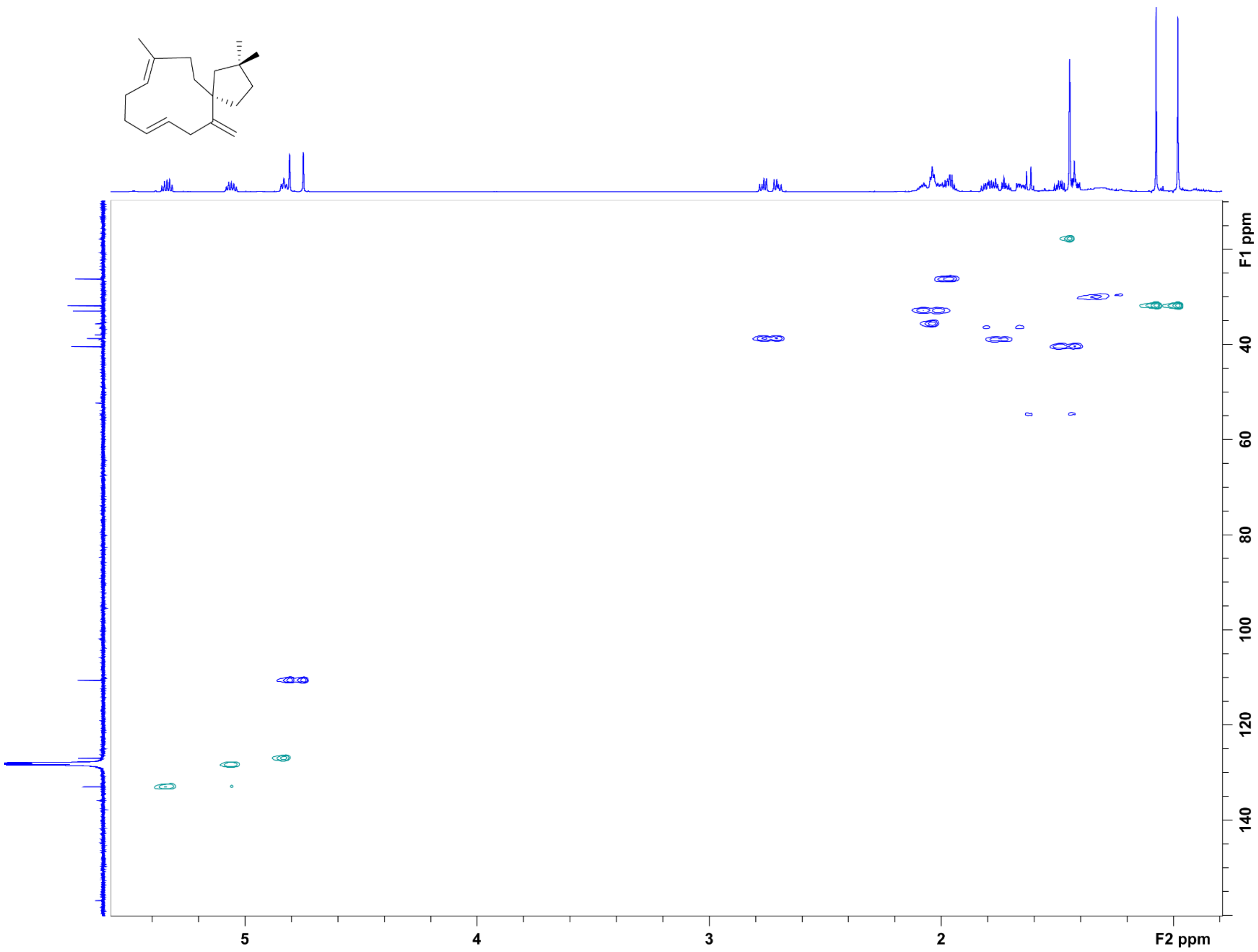

Figure S136. HSQC spectrum (C_6D_6) of **3a**.

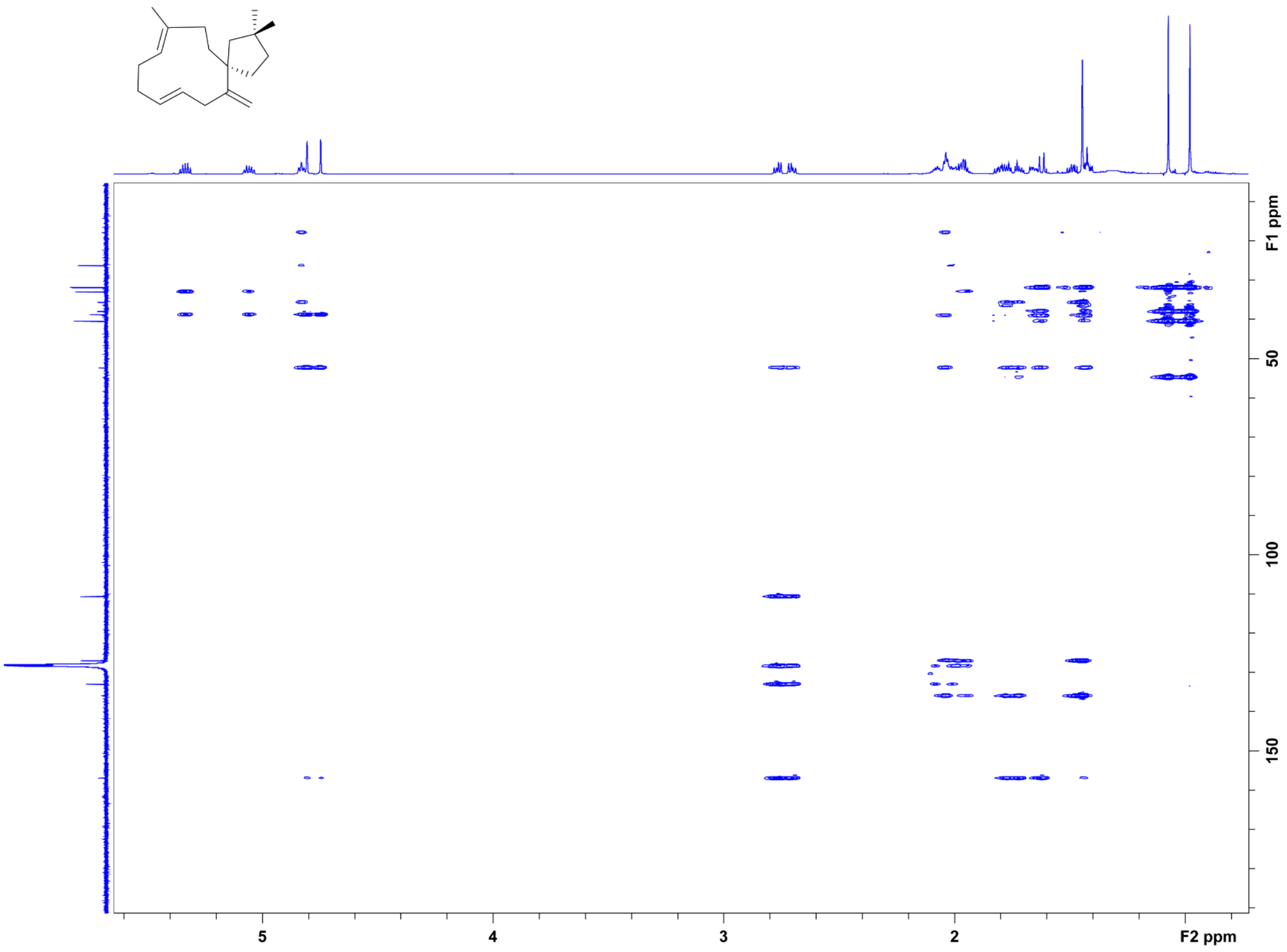

Figure S137. HMBC spectrum (C_6D_6) of **3a**.

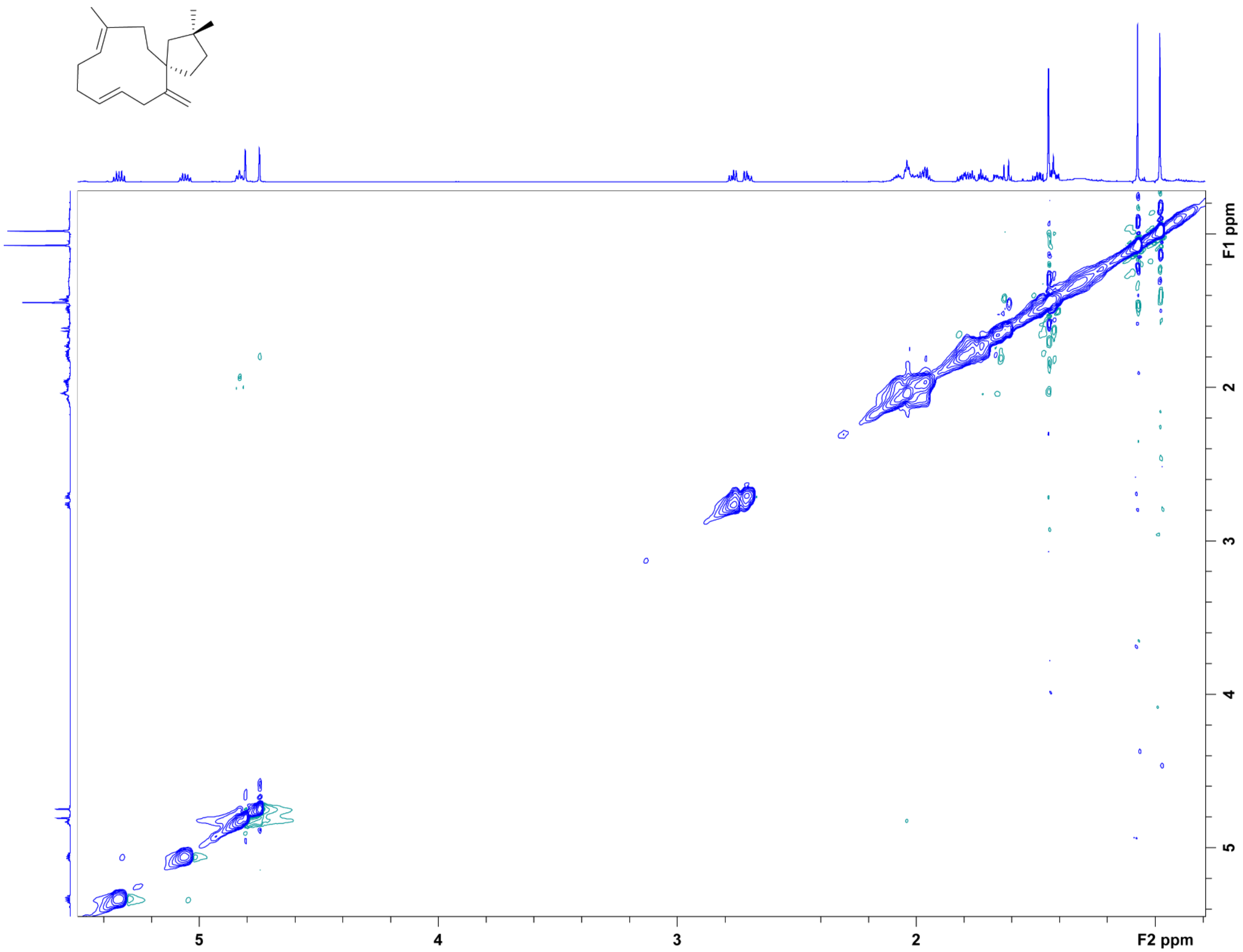

Figure S138. NOESY spectrum (C_6D_6) of **3a**.

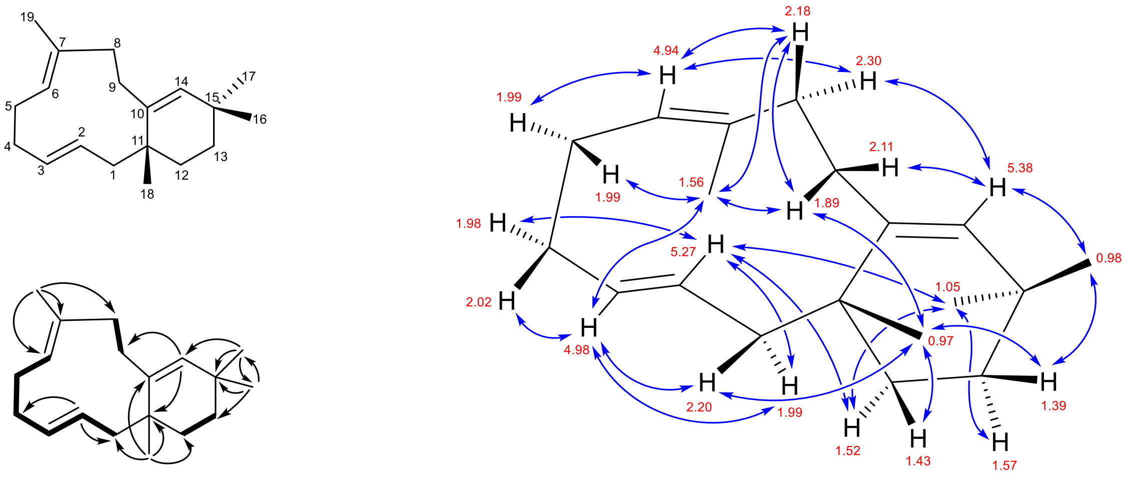

Figure S139. Structure elucidation of 20-*nor*-chryseojoostene D (**4a**). Bold: ^{1}H,^{1}H-COSY correlations, single headed arrows: HMBC correlations, and blue double headed arrows: NOESY correlations. Carbon numbering follows 20-*nor*-GGPP numbering to indicate the origin of each carbon.

Table S21. NMR data of 20-*nor*-chryseojoostene D (**4a**) in C_6D_6 recorded at 298 K.

C[a]	type	^{13}C[b]	^{1}H[b]
1	CH_2	44.82	2.20 (m)
			1.99 (m)
2	CH	130.38	5.27 (ddd, *J* = 14.8, 7.2, 6.2)
3	CH	128.80	4.98 (dddd, *J* = 14.6, 7.6, 1.3, 1.2)
4	CH_2	33.59	2.02 (m)
			1.98 (m)
5	CH_2	26.68	1.99 (m, 2H)
6	CH	124.81	4.94 (br t, *J* = 7.3)
7	C	138.56	–
8	CH_2	36.23	2.30 (ddd, *J* = 12.7, 12.6, 2.0)
			2.18 (m)
9	CH_2	33.03*	2.11 (dddd, *J* = 15.4, 5.8, 2.2, 2.1)
			1.89 (ddd, *J* = 15.4, 12.2, 2.3)
10	C	139.49	–
11	C	38.82	–
12	CH_2	36.70	1.52 (m)
			1.43 (m)
13	CH_2	33.90	1.57 (m)
			1.39 (m)
14	CH	133.24	5.38 (d, *J* = 2.4)
15	C	32.49	–
16	CH_3	30.44	098 (s)
17	CH_3	30.33	1.05 (s)
18	CH_3	26.33	0.97 (s)
19	CH_3	20.04	1.56 (d, *J* = 1.4)

[a] Carbon numbering as shown in Figure S139 indicates the origin of each carbon from 20-*nor*-GGPP by same number. [b] Chemical shifts δ in ppm, multiplicity: s = singlet, d = doublet, t = triplet, m = multiplet, br = broad, coupling constants *J* are given in Hertz. Asterisks indicate carbons that show peak broadening in the ^{13}C-NMR.

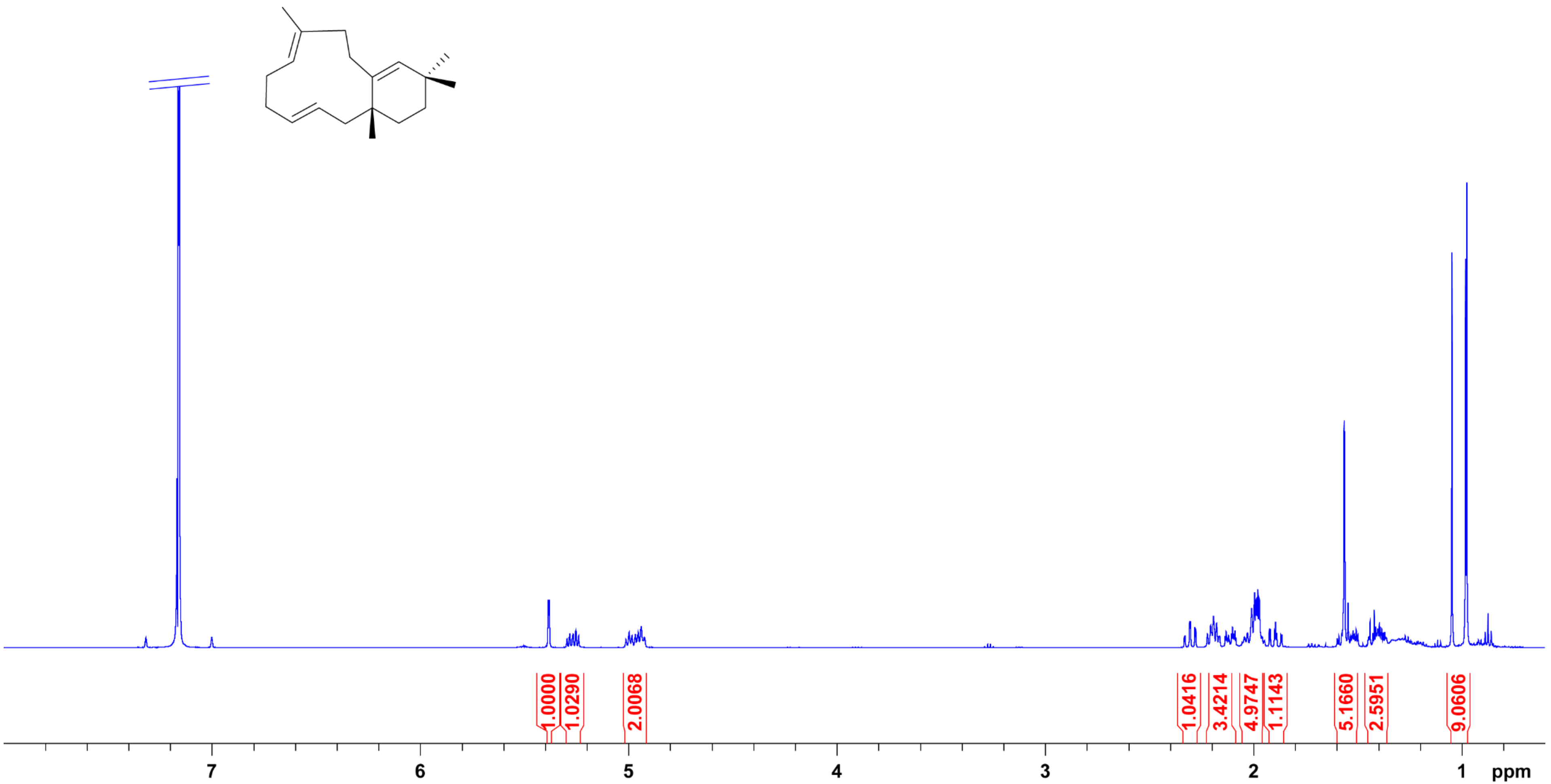

Figure S140. ^{1}H-NMR spectrum of **4a** (700 MHz, C_6D_6).

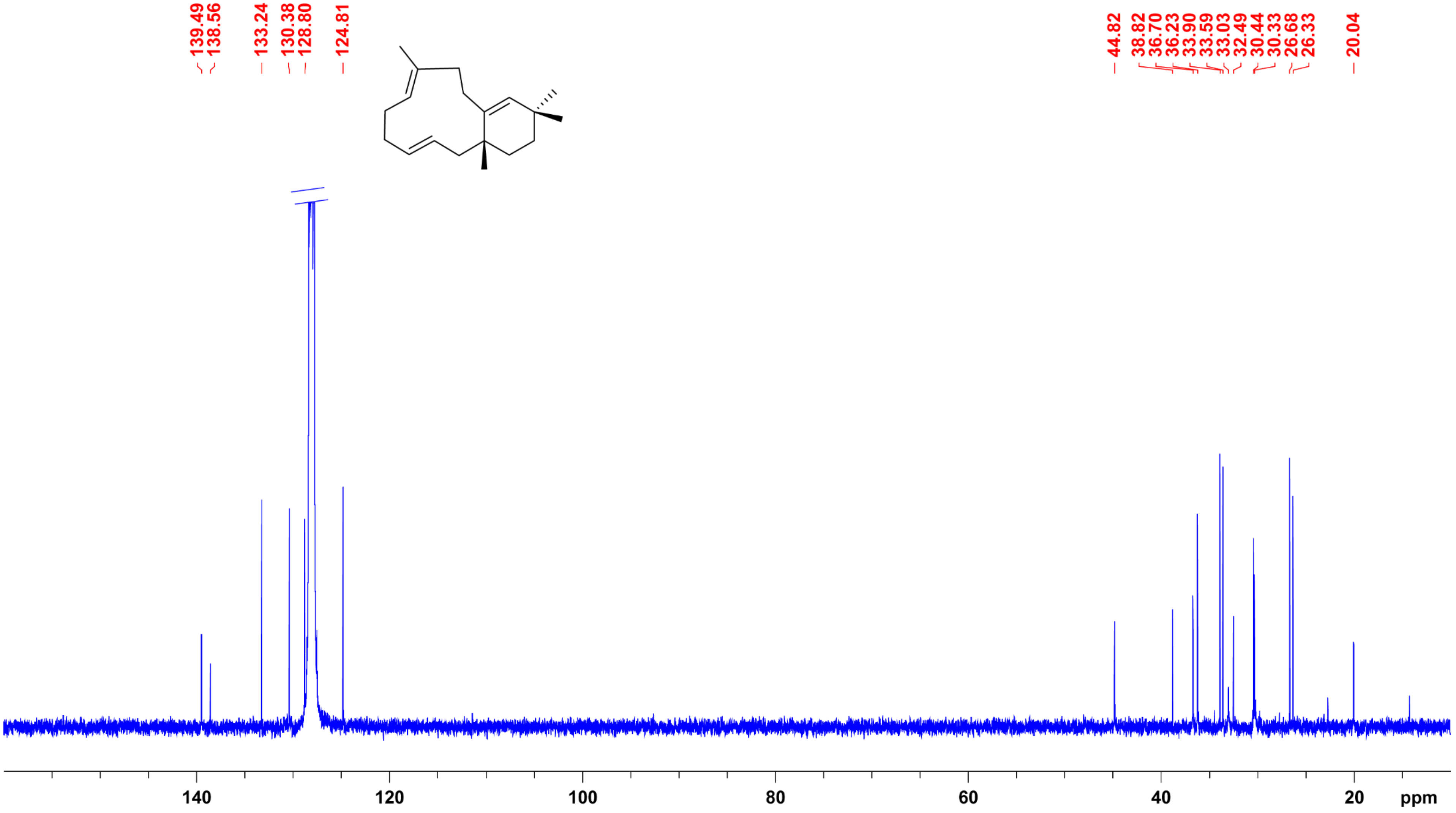

Figure S141. ^{13}C-NMR spectrum of **4a** (176 MHz, C_6D_6).

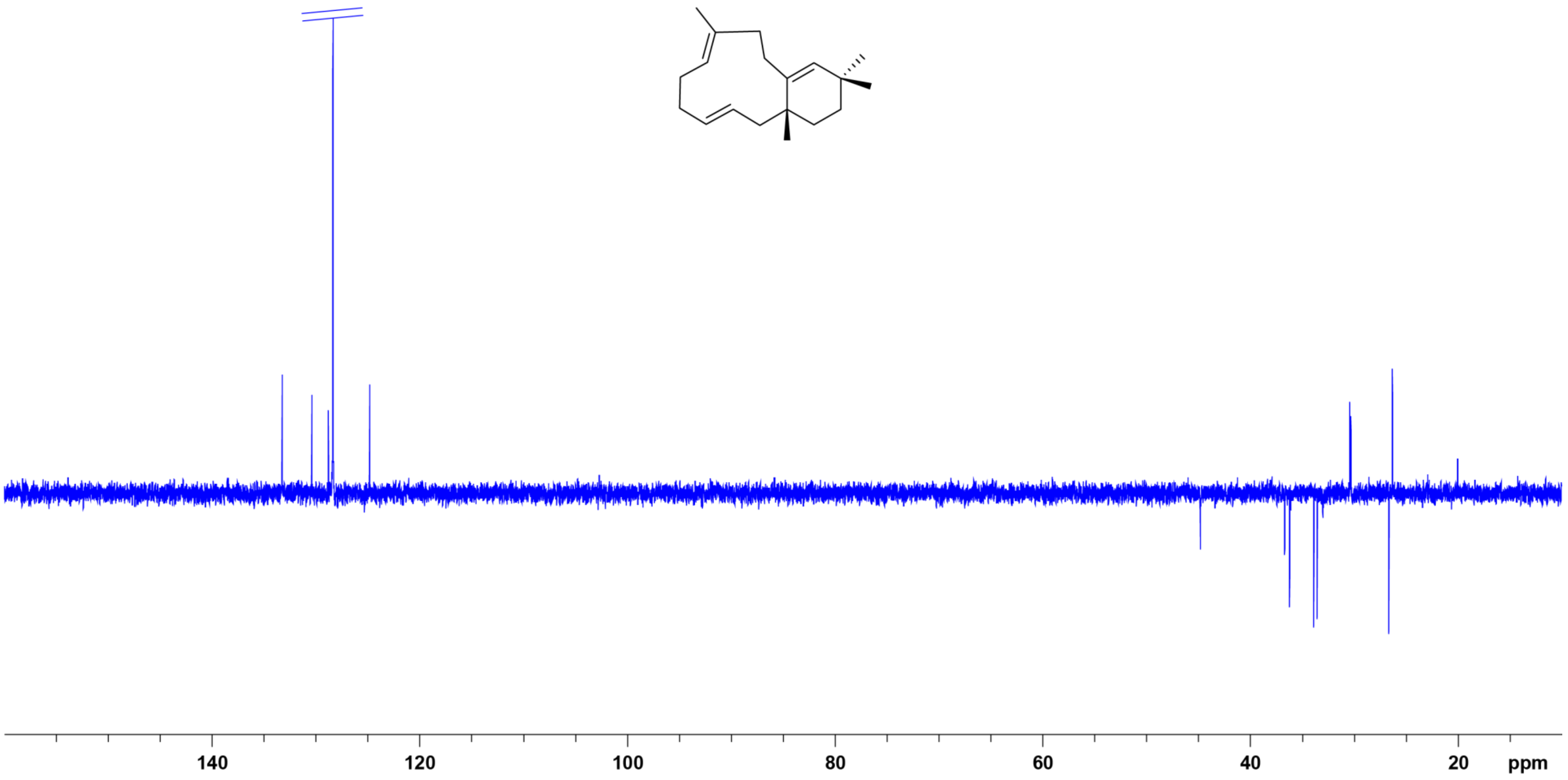

Figure S142. ^{13}C-DEPT135 spectrum of **4a** (176 MHz, C_6D_6).

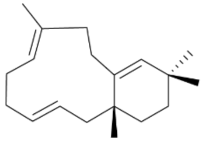

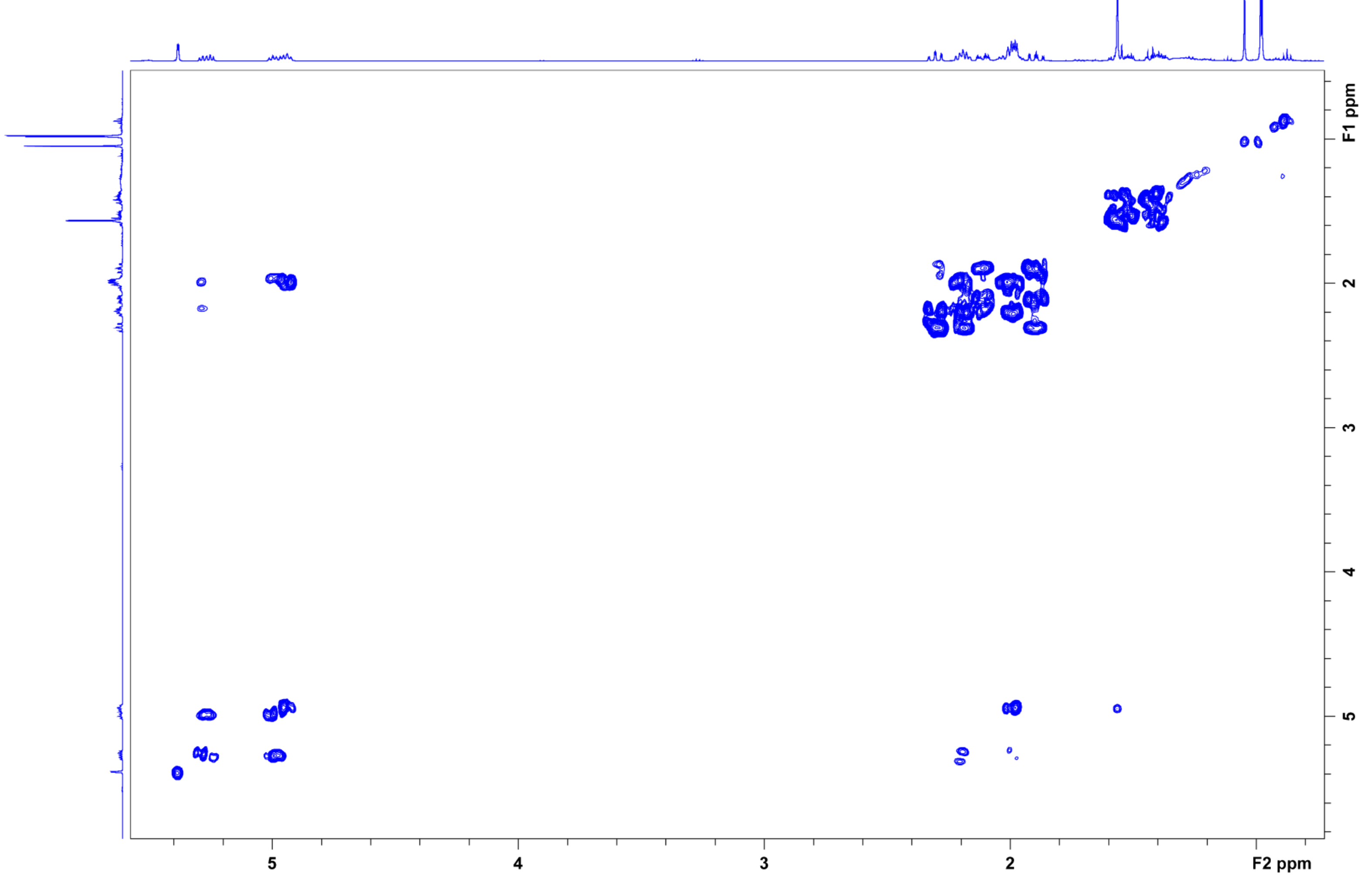

Figure S143. $^{1}H,^{1}H$-COSY spectrum (C_6D_6) of **4a**.

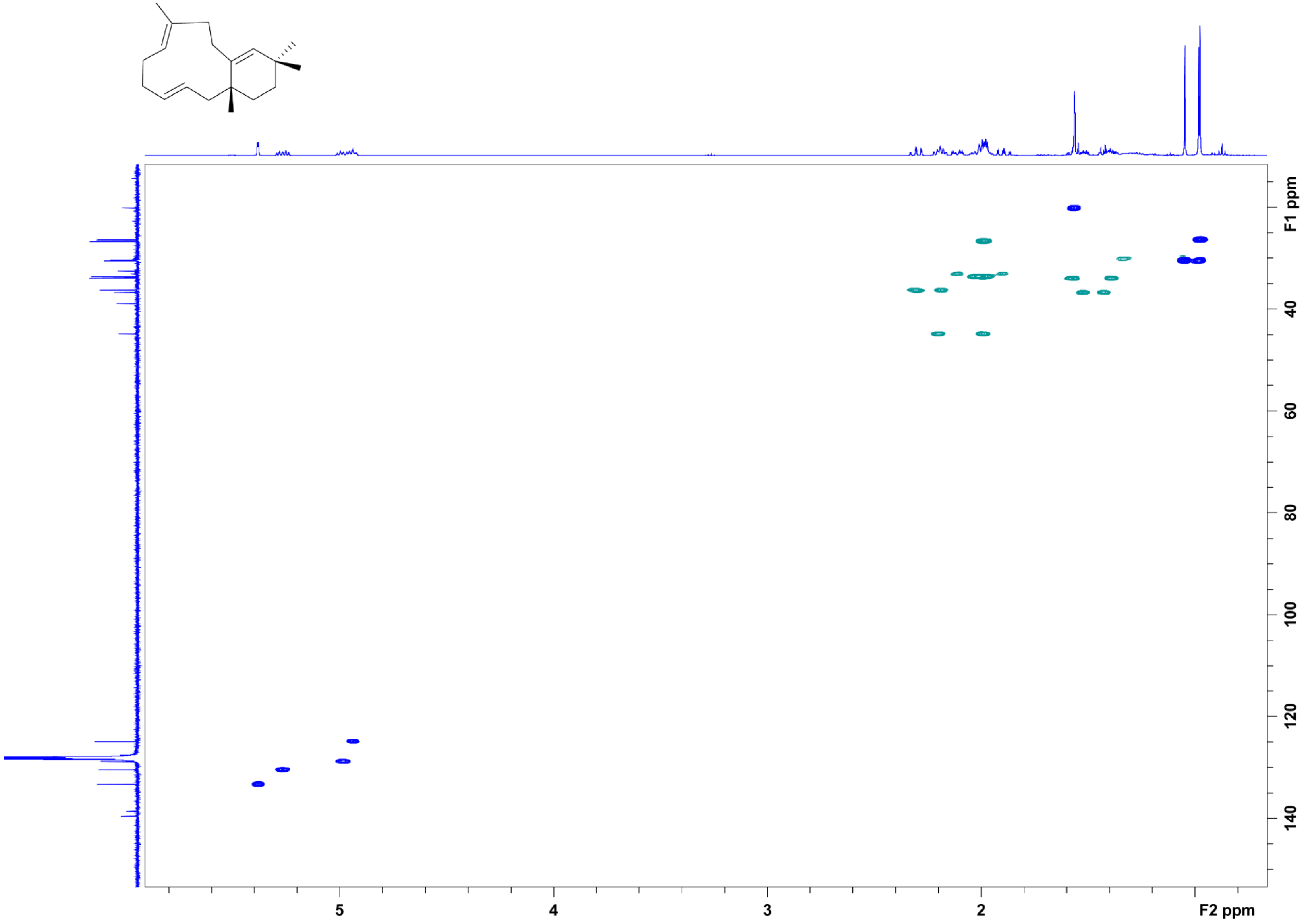

Figure S144. HSQC spectrum (C_6D_6) of **4a**.

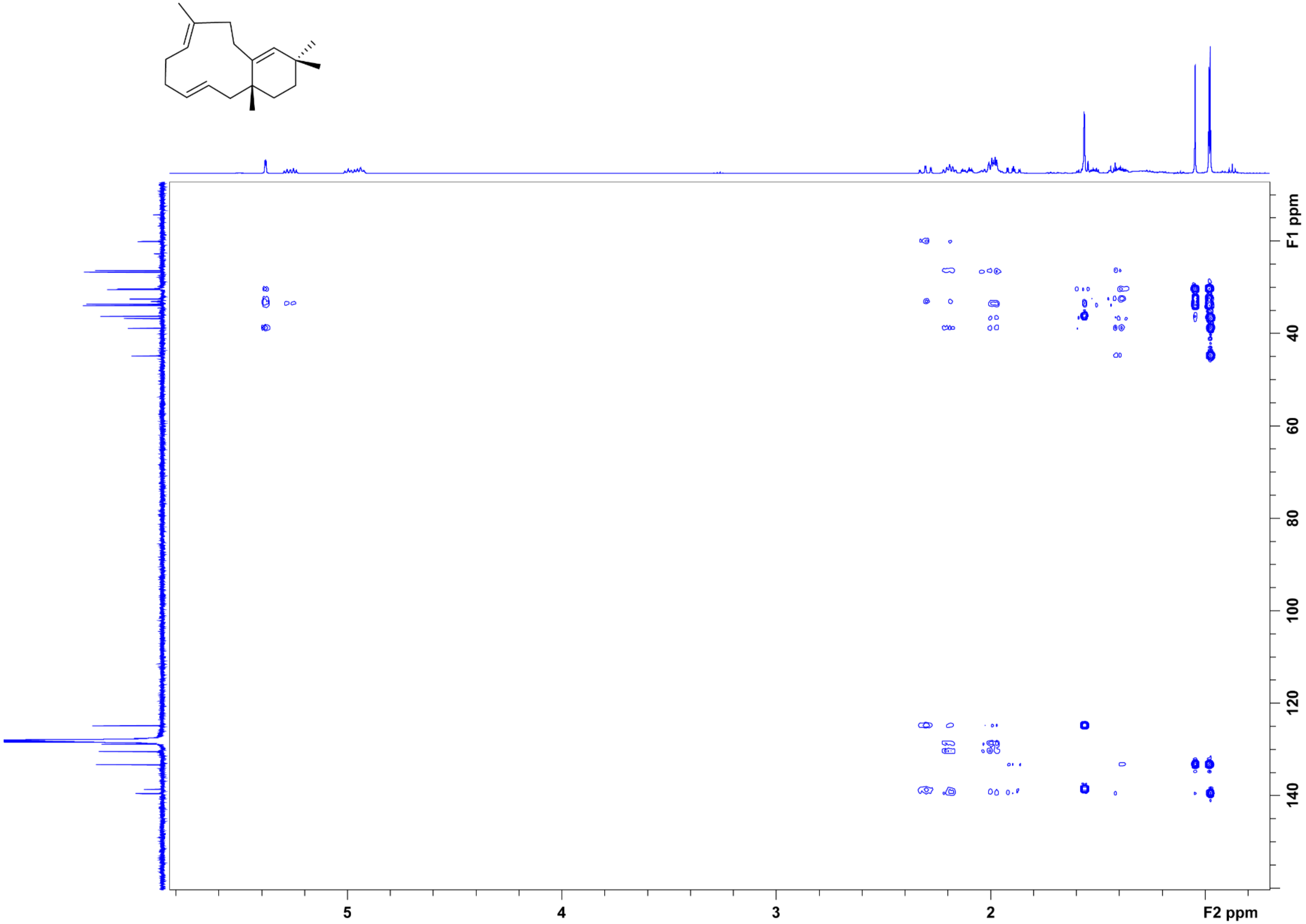

Figure S145. HMBC spectrum (C_6D_6) of **4a**.

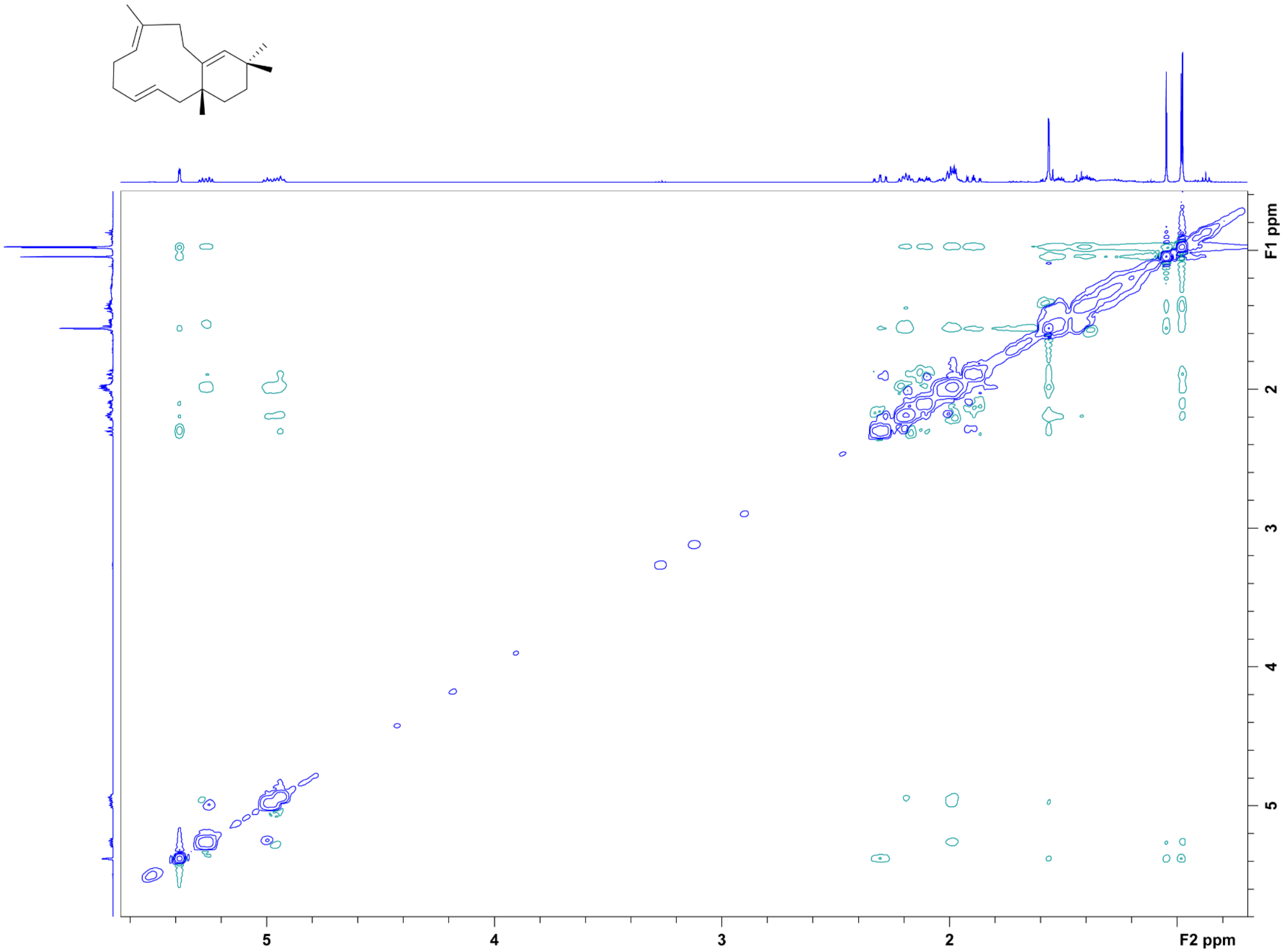

Figure S146. NOESY spectrum (C_6D_6) of **4a**.

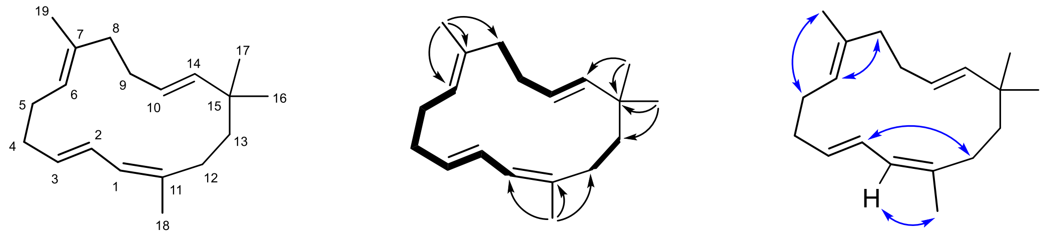

Figure S147. Structure elucidation of macrojoostene (**14**). Bold: $^1H,^1H$-COSY correlations, single headed arrows: HMBC correlations, and blue double headed arrows: NOESY correlations. Carbon numbering follows 20-*nor*-GGPP numbering to indicate the origin of each carbon.

Table S22. NMR data of macrojoostene (**14**) in C_6D_6 recorded at 298 K.

C[a]	type	^{13}C[b]	^{1}H[b]
1	CH	126.22	5.91 (br d, *J* = 10.4)
2	CH	126.99	6.49 (ddt, *J* = 15.4, 10.4, 1.6)
3	CH	130.99	5.72 (dt, *J* = 15.5, 4.8)
4	CH_2	30.52	2.16 (m, 2H)
5	CH_2	26.29	2.23 (m,2H)
6	CH	126.89	5.25 (tsext, *J* = 6.0, 1.3)
7	C	133.89	–
8	CH_2	38.65	2.13 (m, 2H)
9	CH_2	30.11	2.22 (m, 2H)
10	CH	126.57	5.38 (br d, *J* = 15.8)
11	C	137.37	–
12	CH_2	28.46	2.18 (m, 2H)
13	CH_2	40.70	1.45 (m, 2H)
14	CH	139.89	5.40 (d, *J* = 15.8)
15	C	36.38	–
16	CH_3	29.05	0.98 (s)
17	CH_3	29.05	0.98 (s)
18	CH_3	23.48	1.74 (br s)
19	CH_3	16.03	1.57 (q, *J* = 1.1)

[a] Carbon numbering as shown in Figure S147 indicates the origin of each carbon from 20-*nor*-GGPP by same number. [b] Chemical shifts δ in ppm, multiplicity: s = singlet, d = doublet, t = triplet, q = quartet, sext = sextet, m = multiplet, br = broad, coupling constants *J* are given in Hertz.

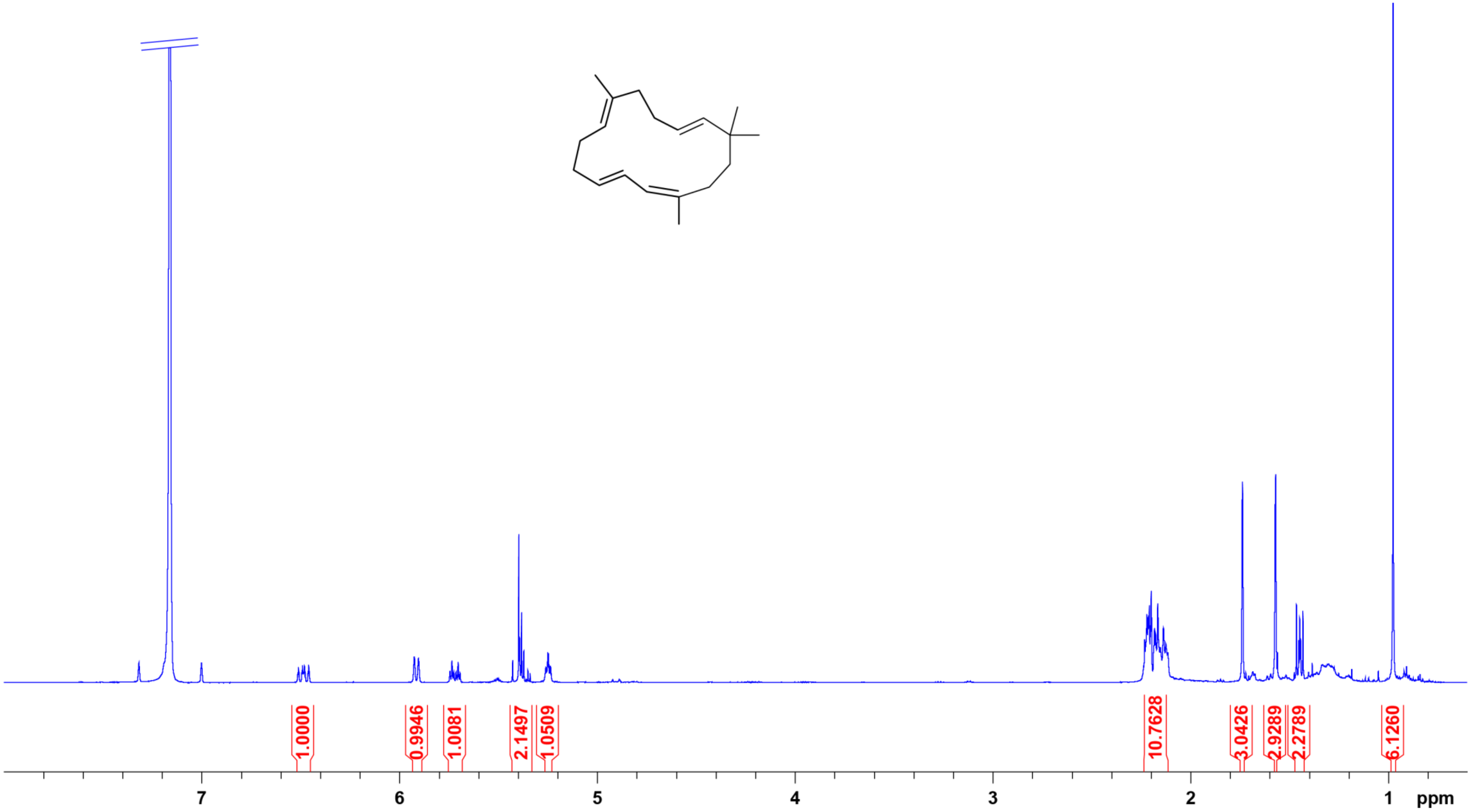

Figure S148. ^{1}H-NMR spectrum of **14** (700 MHz, C_6D_6).

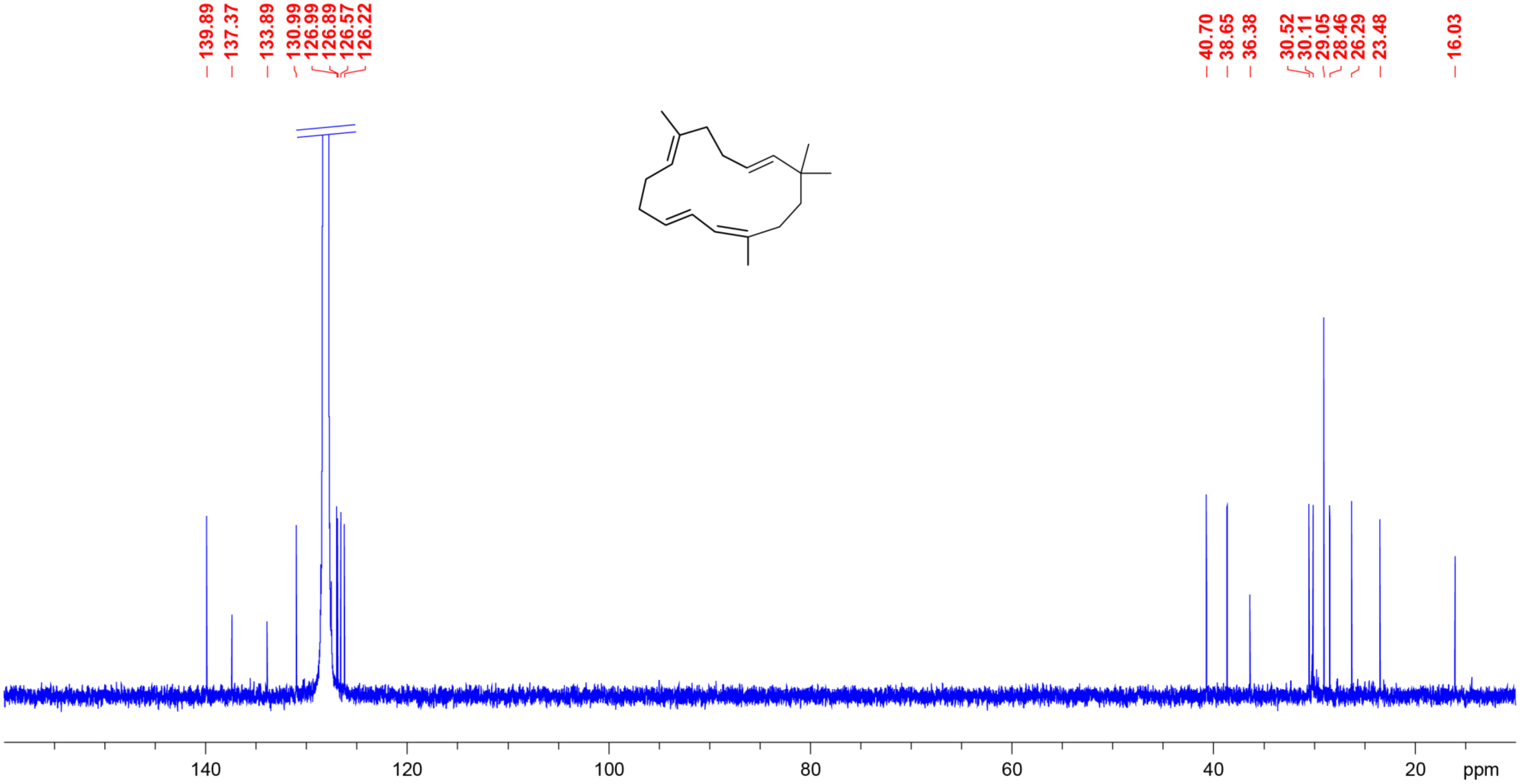

Figure S149. ^{13}C-NMR spectrum of **14** (176 MHz, C_6D_6).

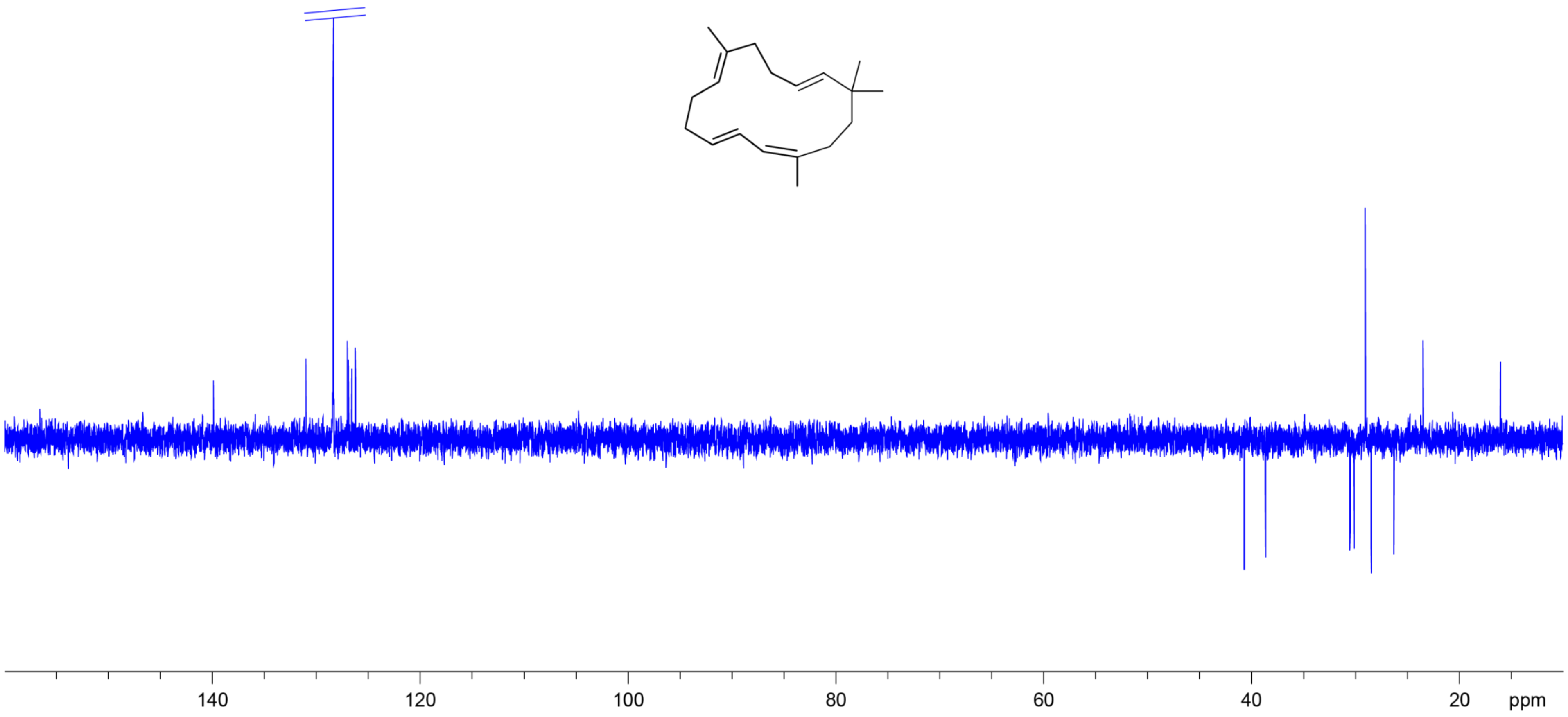

Figure S150. ^{13}C-DEPT135 spectrum of **14** (176 MHz, C_6D_6).

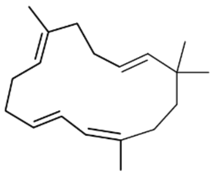

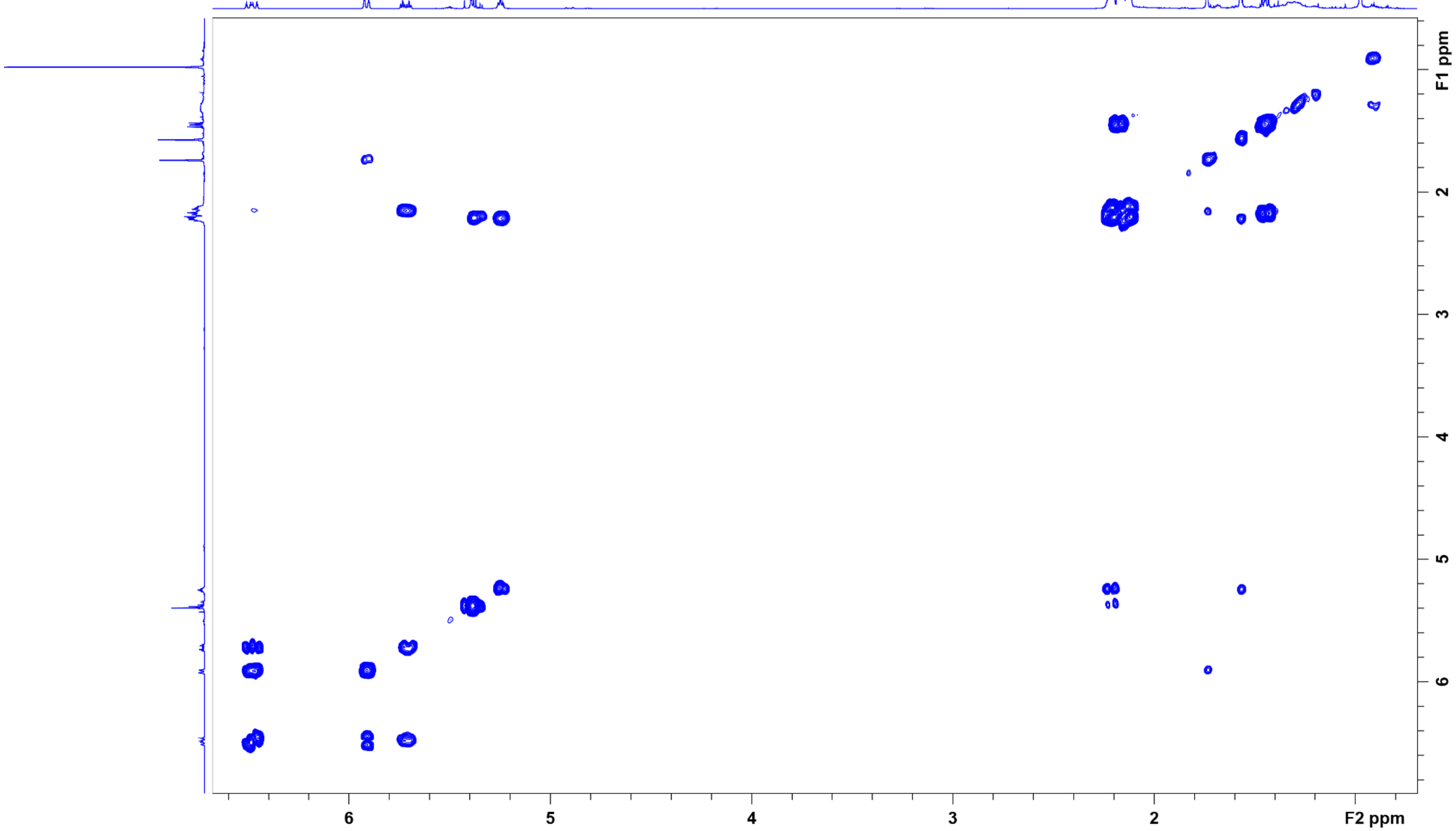

Figure S151. $^{1}H,^{1}H$-COSY spectrum (C_6D_6) of **14**.

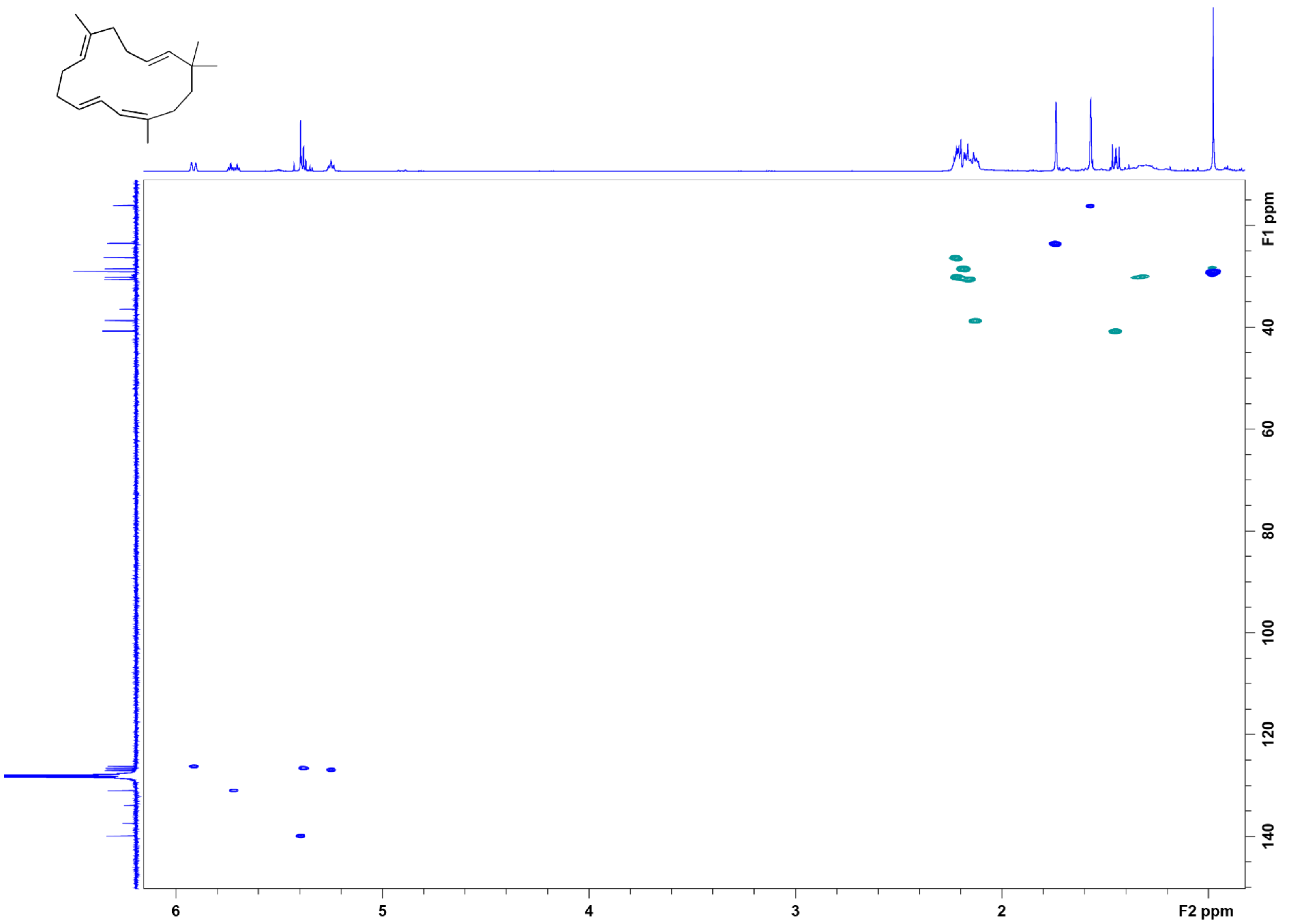

Figure S152. HSQC spectrum (C_6D_6) of **14**.

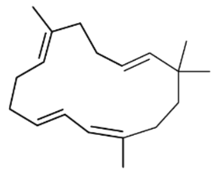

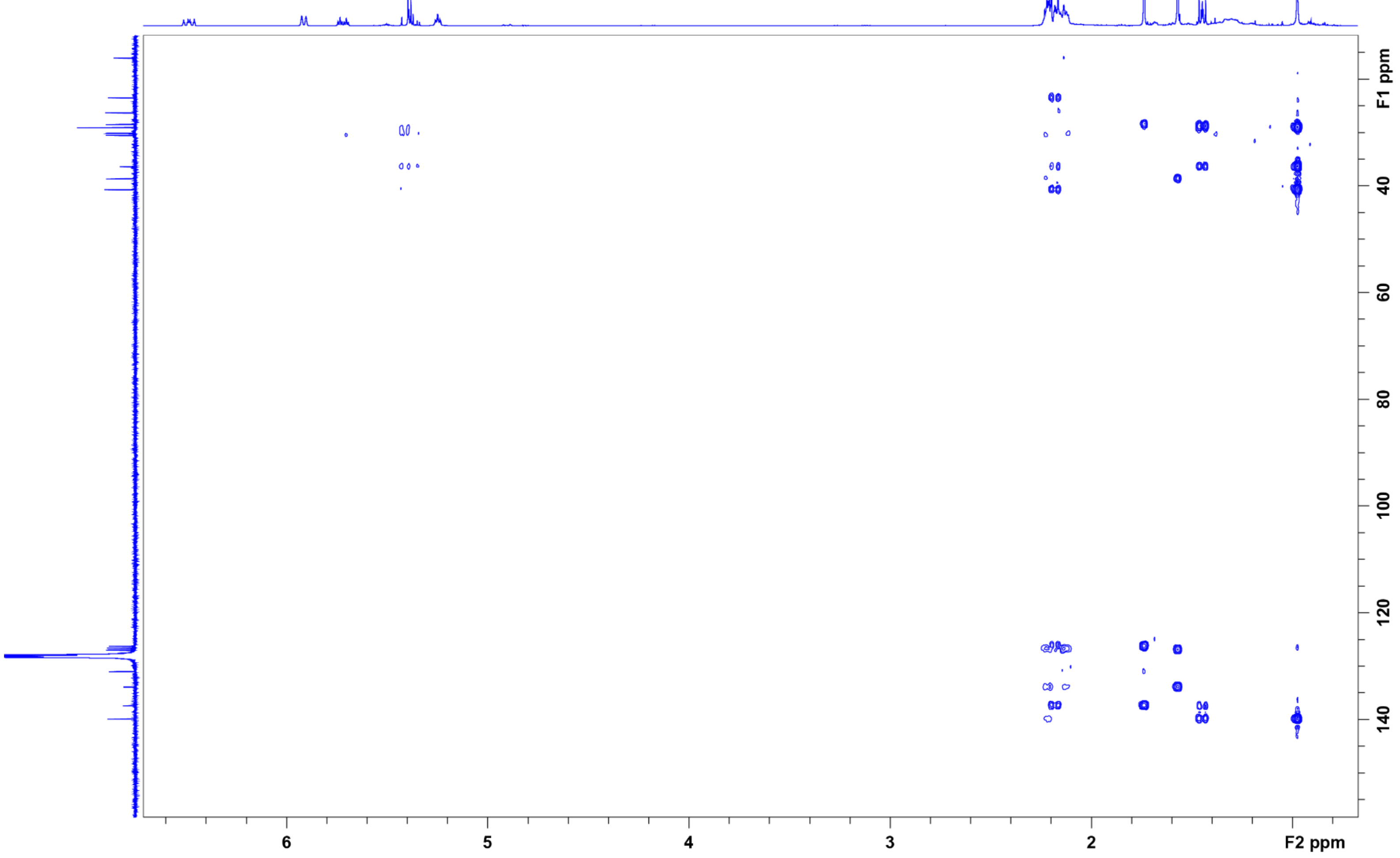

Figure S153. HMBC spectrum (C_6D_6) of **14**.

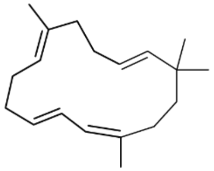

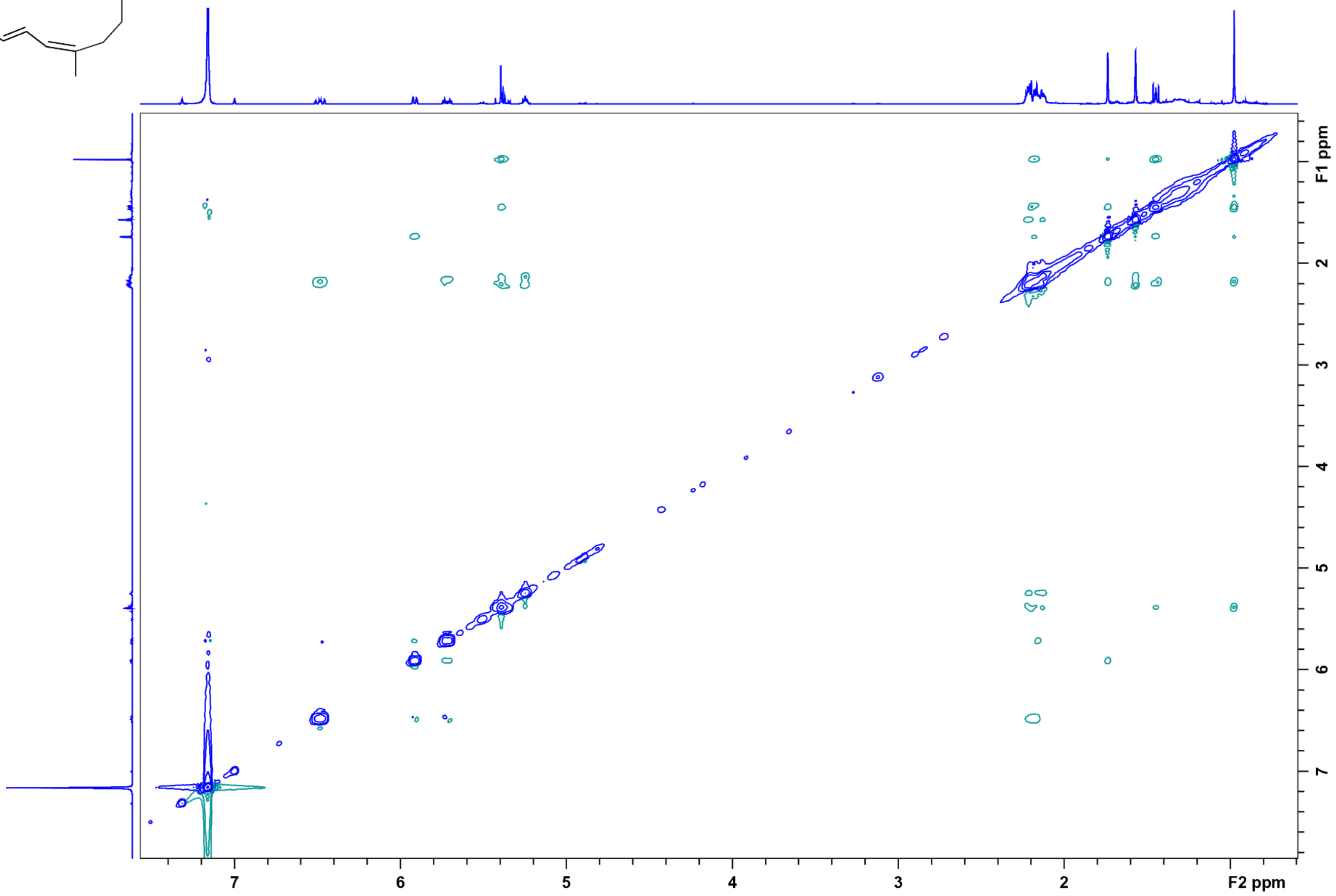

Figure S154. NOESY spectrum (C_6D_6) of **14**.

Cartesian coordinates of computed structures (Scheme 1 of main text, Table S7)

Gibbs energies (G"..." in Hartree) and imaginary frequencies of TS (T-"..." in cm^{-1}), mPW1PW91/6-311+G(d,p)//B97D3/6-31G(d,p)-sp-density-fitting, 1 bar, 298.15 K.

**A**	G781.678830		
C	3.106601	0.659635	1.357690
C	2.743998	1.642474	0.175021
C	1.741195	1.059516	-0.798435
C	0.330857	-1.971084	1.467778
C	1.565211	-1.280120	1.329583
C	1.865470	-0.033861	1.843324
H	3.827290	-0.082281	0.991054
H	2.338287	2.569498	0.602705
C	1.944944	0.024498	-1.640476
C	-1.805263	-1.927266	0.067988
H	2.312758	-1.739705	0.681697
C	-0.639821	-1.317671	-0.387968
C	0.776384	-0.612029	-2.371164
H	1.096603	-0.988377	-3.354101
C	0.976889	0.741014	2.765774
H	0.636360	1.661912	2.266610
H	0.097712	0.193853	3.119770
H	1.552305	1.071234	3.643771
H	3.585203	1.238301	2.160422
H	3.689502	1.912046	-0.317618
C	3.286569	-0.633764	-1.860395
H	3.540676	-0.619396	-2.931412
H	4.106389	-0.149202	-1.318897
H	3.271754	-1.697440	-1.569074
H	0.728896	1.474958	-0.752450
H	-0.021880	0.123649	-2.549003
H	0.309610	-3.035207	1.237243
H	-0.388161	-1.647252	2.217655
H	-0.530351	-0.257636	-0.165142
C	0.179469	-1.808651	-1.569952
H	-0.465216	-2.403814	-2.233416
H	0.991532	-2.483201	-1.256835
C	-2.137785	-3.350296	-0.274193
H	-2.680031	-3.845789	0.542320
H	-2.814135	-3.355561	-1.146583
H	-1.258192	-3.945327	-0.543641
C	-2.817041	-1.194934	0.909479
H	-3.800019	-1.407121	0.448448
H	-2.877915	-1.693066	1.895216
C	-2.647260	0.324994	1.079614
H	-3.484456	0.675156	1.706760
H	-1.734685	0.535446	1.657407
C	-2.625412	1.062019	-0.244398
H	-3.120409	0.551686	-1.078581
C	-2.081236	2.272219	-0.491502
C	-2.128113	2.877554	-1.876040
H	-1.111537	3.096367	-2.241648
H	-2.621421	2.219418	-2.602392

H	-2.665024	3.839095	-1.866313
C	-1.425832	3.135561	0.562835
H	-0.399847	3.411542	0.269087
H	-1.974591	4.085054	0.663547
H	-1.395951	2.672322	1.556010

A-B1-TS G781.668670, T-155

C	3.575679	0.183963	-0.813735
C	3.077190	-1.255484	-1.186589
C	1.765379	-1.625073	-0.524153
C	0.819970	2.455275	0.666577
C	1.948716	1.576466	0.440129
C	2.438823	1.177165	-0.770900
H	4.067775	0.154175	0.166926
H	2.955919	-1.324395	-2.276385
C	1.544630	-1.799165	0.797026
C	-1.099711	1.711899	0.769042
H	2.436292	1.173214	1.330503
C	-0.709407	0.355148	0.646620
C	0.127242	-1.948789	1.320348
H	0.096831	-2.639657	2.175879
C	1.843222	1.576157	-2.093131
H	1.382911	0.699802	-2.578278
H	1.077435	2.356352	-2.019951
H	2.627993	1.924689	-2.780429
H	4.331356	0.493463	-1.552107
H	3.876204	-1.964052	-0.921379
C	2.629734	-1.785715	1.846059
H	2.585777	-2.707619	2.446016
H	3.638010	-1.708601	1.426265
H	2.497284	-0.953620	2.557869
H	0.900621	-1.706036	-1.191243
H	-0.525831	-2.365096	0.542253
H	0.774659	2.894874	1.663926
H	0.576149	3.160715	-0.126510
H	-0.335641	0.041125	-0.328386
C	-0.455340	-0.585858	1.775300
H	-1.389955	-0.714725	2.347448
H	0.237299	-0.099199	2.486158
C	-1.604197	2.212929	2.113391
H	-1.629269	3.310264	2.133102
H	-2.634887	1.862310	2.280077
H	-0.992099	1.867596	2.955423
C	-1.856182	2.294303	-0.448960
H	-2.698037	2.878426	-0.047802
H	-1.256279	3.003403	-1.034457
C	-2.403669	1.178010	-1.372809
H	-3.215806	1.595834	-1.989130
H	-1.628117	0.844758	-2.076728
C	-2.915025	0.032081	-0.539788
H	-3.549332	0.318481	0.306816
C	-2.788712	-1.307656	-0.781052
C	-3.467914	-2.306291	0.121164
H	-2.758051	-3.063260	0.487250

H	-3.954414	-1.832418	0.982047
H	-4.235852	-2.859575	-0.443337
C	-2.065364	-1.884659	-1.971096
H	-1.476783	-2.770023	-1.691623
H	-2.803673	-2.230799	-2.712730
H	-1.408890	-1.168647	-2.478151

B1 G781.715347

C	-3.388128	-0.671431	-0.610407
C	-3.330833	0.874341	-0.848831
C	-2.043335	1.481841	-0.341782
C	0.076930	-2.436971	-0.097938
C	-1.312704	-1.878696	0.025651
C	-2.080223	-1.355729	-0.956169
H	-3.630999	-0.860963	0.444201
H	-3.431412	1.085347	-1.922814
C	-1.677832	1.626129	0.949453
C	1.154362	-1.681169	0.763297
H	-1.721118	-1.843446	1.041568
C	0.977216	-0.108614	0.533013
C	-0.256420	2.015686	1.311138
H	-0.250410	2.736844	2.143834
C	-1.704342	-1.328551	-2.420700
H	-1.575536	-0.293696	-2.778941
H	-0.791025	-1.887090	-2.655674
H	-2.515347	-1.757929	-3.028327
H	-4.213815	-1.083399	-1.213433
H	-4.207874	1.328277	-0.363283
C	-2.590221	1.323465	2.114739
H	-2.545073	2.128470	2.863870
H	-3.635599	1.202072	1.811849
H	-2.289145	0.398234	2.633447
H	-1.302627	1.748416	-1.104513
H	0.215997	2.519314	0.448980
H	0.101862	-3.491326	0.227596
H	0.400258	-2.431976	-1.148747
H	0.204583	0.016407	-0.238991
C	0.586984	0.780108	1.718836
H	1.480829	1.086051	2.286124
H	-0.009798	0.185410	2.422636
C	1.044577	-2.096290	2.241550
H	1.300828	-3.160470	2.335613
H	1.736442	-1.530727	2.880195
H	0.029379	-1.969080	2.637979
C	2.563208	-2.020988	0.216500
H	3.311399	-1.953584	1.021613
H	2.616672	-3.038837	-0.193111
C	2.856153	-0.954629	-0.843800
H	3.910652	-0.891374	-1.142660
H	2.257041	-1.128398	-1.748297
C	2.403979	0.335733	-0.113741
H	3.080818	0.544277	0.729121
C	2.195536	1.529199	-0.907531
C	2.746370	2.824606	-0.441406

H	2.270929	3.701011	-0.893818
H	2.758852	2.895923	0.653625
H	3.812282	2.826008	-0.749028
C	1.522547	1.481724	-2.227389
H	0.865216	2.348982	-2.373182
H	2.316257	1.583699	-2.993637
H	0.986541	0.548986	-2.420693

B2 G781.709552

C	2.862066	1.950533	-0.225494
C	3.373091	0.562201	-0.678367
C	2.279609	-0.239695	-1.340145
C	-0.124959	0.682307	2.028790
C	1.174713	0.871963	1.296173
C	1.622918	1.979756	0.659663
H	3.682805	2.498153	0.269790
H	4.208959	0.729291	-1.378617
C	1.941560	-1.531846	-1.123594
C	-0.846830	-0.664411	1.635634
H	1.779339	-0.032760	1.219212
C	-0.837902	-0.842378	0.057312
C	0.642171	-2.086050	-1.673900
H	0.797541	-3.086319	-2.109693
C	0.981984	3.346665	0.776689
H	1.685209	4.042193	1.261820
H	0.765195	3.781336	-0.212196
H	0.062799	3.354415	1.374246
H	2.630642	2.546842	-1.127311
H	3.796134	0.018597	0.177631
C	2.752066	-2.485923	-0.277198
H	2.135159	-2.997583	0.476496
H	3.173382	-3.279137	-0.915123
H	3.589130	-2.005846	0.240467
H	1.655848	0.332467	-2.040803
H	0.262576	-1.439680	-2.482509
H	0.031471	0.657665	3.121103
H	-0.791500	1.537729	1.844522
H	-0.091562	-0.131489	-0.320517
C	-0.440101	-2.214140	-0.559541
H	-1.318494	-2.725170	-0.983519
H	-0.061927	-2.887480	0.217495
C	-0.205782	-1.817557	2.424825
H	-0.291201	-1.620411	3.502109
H	-0.700360	-2.777543	2.227387
H	0.862256	-1.930852	2.197696
C	-2.349187	-0.538109	1.981064
H	-2.814927	-1.535165	2.018828
H	-2.520243	-0.043784	2.948831
C	-2.988146	0.264260	0.839162
H	-4.074110	0.158340	0.747335
H	-2.748709	1.333399	0.917283
C	-2.244746	-0.408205	-0.434182
H	-2.880298	-1.271541	-0.691700
C	-2.371392	0.629774	-1.441601

C	-3.622426	0.751376	-2.226739
H	-3.368844	0.627304	-3.296100
H	-4.384178	0.014066	-1.956186
H	-4.028795	1.772858	-2.153572
C	-1.289297	1.606943	-1.682429
H	-0.568155	1.115338	-2.365218
H	-1.635016	2.526593	-2.166120
H	-0.696589	1.813401	-0.779801

B2-C1-TS G781.691576, T-219

C	2.767642	2.173511	-0.324132
C	3.451090	0.810576	-0.584222
C	2.508381	-0.178821	-1.226855
C	-0.316758	0.857193	1.758236
C	1.030523	1.082357	1.130006
C	1.455569	2.170262	0.447426
H	3.483570	2.847606	0.177322
H	4.320128	0.999905	-1.236674
C	2.327291	-1.475851	-0.899096
C	-0.903054	-0.575445	1.480918
H	1.702333	0.225504	1.187372
C	-0.644436	-1.002138	-0.091060
C	1.147210	-2.253327	-1.456278
H	1.450196	-3.271869	-1.746903
C	0.700046	3.478678	0.370896
H	1.330079	4.290807	0.766251
H	0.472520	3.751738	-0.671833
H	-0.232198	3.485502	0.947224
H	2.562731	2.654635	-1.297935
H	3.856500	0.411332	0.356124
C	3.183572	-2.226570	0.095983
H	2.594898	-2.619400	0.940972
H	3.638714	-3.103912	-0.389776
H	3.997689	-1.622845	0.509489
H	1.853422	0.241943	-2.001245
H	0.750425	-1.762041	-2.358666
H	-0.259071	0.942983	2.857502
H	-1.028159	1.631178	1.440008
H	0.039271	-0.213014	-0.450592
C	0.017636	-2.378621	-0.392897
H	-0.738567	-3.097126	-0.746886
H	0.439834	-2.818727	0.515059
C	-0.269234	-1.574198	2.460078
H	-0.510279	-1.265701	3.486840
H	-0.651964	-2.593231	2.324423
H	0.824285	-1.597587	2.377078
C	-2.437095	-0.561252	1.687471
H	-2.803296	-1.596012	1.752292
H	-2.669936	-0.080579	2.650814
C	-3.217302	0.188175	0.602136
H	-4.235980	-0.186114	0.483682
H	-3.214419	1.272304	0.756277
C	-1.941043	-0.886072	-0.772710
H	-2.510425	-1.815057	-0.922689

C	-2.627843	0.308155	-1.110503
C	-3.875839	0.201741	-1.985282
H	-3.582618	0.281483	-3.040819
H	-4.406731	-0.747551	-1.843006
H	-4.565896	1.027853	-1.771854
C	-1.846172	1.598904	-1.325930
H	-1.557064	1.645818	-2.385329
H	-2.475593	2.473707	-1.121258
H	-0.935511	1.662922	-0.728364

**C1**	G781.701384		
C	2.842295	2.199722	-0.121382
C	3.568489	0.860547	-0.460692
C	2.665847	-0.055750	-1.235731
C	-0.297318	0.956775	1.908722
C	1.102257	1.252294	1.434714
C	1.407402	2.043101	0.367542
H	3.436700	2.729909	0.639429
H	4.474652	1.112095	-1.036137
C	2.389557	-1.343512	-0.923232
C	-0.819417	-0.466976	1.480170
H	1.922208	0.760824	1.967016
C	-0.418693	-0.677828	-0.024617
C	1.211766	-2.040016	-1.563671
H	1.459827	-3.072638	-1.857473
C	0.321098	2.716901	-0.434388
H	0.724300	3.430613	-1.161189
H	-0.258991	1.964803	-1.016982
H	-0.411507	3.239977	0.193814
H	2.844839	2.840238	-1.014452
H	3.910454	0.375071	0.463255
C	3.107725	-2.088334	0.186382
H	2.848505	-1.695366	1.181947
H	2.853763	-3.155842	0.181183
H	4.197366	-2.004526	0.086429
H	2.123475	0.398538	-2.074664
H	0.896814	-1.513060	-2.477784
H	-0.349000	1.013456	3.006711
H	-0.981157	1.727019	1.532903
H	0.562496	-0.098657	-0.167227
C	0.042238	-2.114666	-0.554145
H	-0.806382	-2.659634	-0.988778
H	0.351667	-2.676505	0.333908
C	-0.190516	-1.522157	2.410891
H	-0.432308	-1.277369	3.453455
H	-0.582903	-2.529090	2.217043
H	0.903551	-1.558075	2.326483
C	-2.360310	-0.517565	1.639583
H	-2.700798	-1.550855	1.460458
H	-2.619488	-0.290708	2.684171
C	-3.106552	0.451302	0.711152
H	-4.192816	0.327600	0.825496
H	-2.894436	1.492722	0.993497
C	-1.334103	-0.142159	-0.988200

H	-1.020296	-0.203821	-2.039539
C	-2.737939	0.261973	-0.776667
C	-3.486204	-1.014731	-1.360820
H	-3.231889	-1.199306	-2.411381
H	-3.281725	-1.917629	-0.775581
H	-4.559746	-0.790698	-1.298715
C	-3.144221	1.479459	-1.641156
H	-2.899123	1.326394	-2.700739
H	-4.227666	1.634529	-1.560225
H	-2.640791	2.389697	-1.293064

C2 G781.695110

C	-3.325775	-1.769124	-0.454562
C	-3.714596	-0.260651	-0.433222
C	-2.640443	0.578350	-1.077940
C	0.062311	-1.490270	1.536047
C	-1.378518	-1.522613	1.095981
C	-1.878567	-2.047181	-0.044126
H	-4.021201	-2.328592	0.190965
H	-4.677755	-0.152413	-0.959574
C	-2.130370	1.758489	-0.660535
C	0.698285	-0.059808	1.438031
H	-2.071358	-0.971996	1.738058
C	0.535391	0.471542	-0.164766
C	-0.855924	2.287527	-1.295156
H	-0.904911	3.377827	-1.444103
C	-1.082169	-2.867513	-1.031076
H	-1.620539	-3.795885	-1.273740
H	-0.969848	-2.326464	-1.986516
H	-0.085651	-3.155115	-0.672401
H	-3.478784	-2.156401	-1.473326
H	-3.895336	0.068628	0.600267
C	-2.708635	2.590930	0.460750
H	-1.925215	3.036275	1.091032
H	-3.278803	3.434060	0.039034
H	-3.394063	2.031284	1.108445
H	-2.174394	0.125367	-1.963208
H	-0.708704	1.830972	-2.285978
H	0.138432	-1.775920	2.598573
H	0.666198	-2.219526	0.979931
H	-0.438423	0.010120	-0.397353
C	0.409956	1.996394	-0.421822
H	1.280680	2.387617	-0.957248
H	0.343845	2.560122	0.514977
C	-0.066616	0.904652	2.353672
H	-0.108791	0.461272	3.359241
H	0.430408	1.876542	2.443547
H	-1.094108	1.062636	2.016006
C	2.201322	-0.060189	1.789107
H	2.543406	0.975042	1.930136
H	2.333629	-0.558928	2.762734
C	3.081917	-0.763808	0.767038
H	4.154972	-0.660088	0.987341
H	2.865287	-1.835798	0.688552

C	1.580120	-0.285438	-0.807141
H	1.304099	-1.298107	-1.128420
C	3.016248	-0.075205	-0.754917
C	3.602993	1.342290	-0.689392
H	3.499801	1.834383	-1.664780
H	3.134923	1.977093	0.067503
H	4.674738	1.275073	-0.464258
C	3.822801	-0.977987	-1.705849
H	3.801463	-0.565603	-2.722842
H	4.870479	-1.024009	-1.384378
H	3.427140	-2.001785	-1.738952

C2-D-TS G781.704203, T-926

C	2.027532	2.440452	0.018713
C	3.074032	1.435374	-0.517564
C	2.424157	0.261171	-1.203516
C	-0.268764	0.019219	2.203170
C	0.817615	0.655930	1.362039
C	0.917505	1.959949	0.935788
H	2.553989	3.261210	0.536162
H	3.722299	1.993634	-1.212686
C	2.595471	-1.044276	-0.903239
C	-1.163162	-0.986729	1.348254
H	1.696840	0.030466	1.205653
C	-0.505191	-1.153838	-0.060541
C	1.587559	-2.074324	-1.375963
H	2.068982	-3.046701	-1.556510
C	-0.059874	3.042183	1.310857
H	-0.533249	3.487376	0.421998
H	-0.842073	2.714383	2.000691
H	0.490873	3.862326	1.797240
H	1.529403	2.936183	-0.836204
H	3.716762	1.102350	0.308124
C	3.656478	-1.569424	0.038626
H	3.223627	-2.006298	0.954041
H	4.213433	-2.381968	-0.451831
H	4.384521	-0.811099	0.342855
H	1.656269	0.521837	-1.943260
H	1.128238	-1.773827	-2.328610
H	0.210805	-0.536203	3.021435
H	-0.921197	0.762377	2.678107
H	0.213393	0.006080	-0.058420
C	0.510922	-2.287945	-0.274372
H	-0.071217	-3.197040	-0.491751
H	1.019147	-2.483689	0.677098
C	-1.269711	-2.337737	2.090108
H	-1.806535	-2.181912	3.035809
H	-1.831719	-3.078562	1.506040
H	-0.290334	-2.763114	2.339759
C	-2.601750	-0.429085	1.173971
H	-3.252245	-1.243070	0.814552
H	-2.986008	-0.145842	2.164304
C	-2.689044	0.760074	0.214305
H	-3.716784	1.147871	0.173780

H	-2.065110	1.584190	0.582920
C	-1.090327	-0.575963	-1.184900
H	-0.648385	-0.808379	-2.160507
C	-2.231642	0.385005	-1.213511
C	-3.367049	-0.356110	-1.992355
H	-3.039361	-0.663962	-2.993943
H	-3.718333	-1.244380	-1.454028
H	-4.211118	0.336642	-2.108451
C	-1.818325	1.643851	-2.020350
H	-1.551876	1.391301	-3.055524
H	-2.659840	2.348746	-2.047709
H	-0.961772	2.152413	-1.558250

**D**	G781.705021		
C	2.001237	2.437806	0.132487
C	3.071659	1.445778	-0.393592
C	2.465794	0.299113	-1.167172
C	-0.338815	-0.126261	2.089637
C	0.721838	0.597443	1.301779
C	0.868305	1.936807	1.011895
H	2.510453	3.250254	0.678102
H	3.753319	2.032760	-1.030169
C	2.576878	-1.016183	-0.874692
C	-1.226381	-1.161965	1.208432
H	1.578187	-0.025492	1.052772
C	-0.580332	-1.288423	-0.169554
C	1.605946	-2.026219	-1.459704
H	2.112632	-2.979153	-1.671712
C	-0.064366	3.010109	1.498822
H	-0.408380	3.660847	0.682344
H	-0.931955	2.628768	2.044408
H	0.497387	3.660320	2.188892
H	1.518376	2.945275	-0.723904
H	3.669440	1.080076	0.451891
C	3.535514	-1.573687	0.157656
H	3.021424	-1.912960	1.073182
H	4.045753	-2.458620	-0.250191
H	4.308169	-0.859751	0.459019
H	1.781799	0.593262	-1.971412
H	1.185081	-1.677670	-2.412400
H	0.169452	-0.713925	2.866318
H	-1.030622	0.553239	2.602164
H	0.130058	0.330937	-0.169820
C	0.475255	-2.340938	-0.430936
H	-0.089878	-3.224838	-0.781429
H	0.925003	-2.656249	0.519175
C	-1.311079	-2.504224	1.966231
H	-1.824966	-2.345662	2.923384
H	-1.885775	-3.247781	1.398224
H	-0.325158	-2.928513	2.192349
C	-2.664555	-0.591485	1.047027
H	-3.293800	-1.365024	0.579449
H	-3.081969	-0.422516	2.050255
C	-2.722637	0.701442	0.228998

H	-3.762039	1.049881	0.143921
H	-2.177781	1.497334	0.754798
C	-0.876665	-0.341484	-1.141590
H	-0.342706	-0.414477	-2.092819
C	-2.121496	0.535851	-1.184569
C	-3.120100	-0.193171	-2.127654
H	-2.687276	-0.349892	-3.125223
H	-3.415192	-1.172397	-1.729328
H	-4.023936	0.419368	-2.245607
C	-1.772084	1.910709	-1.794389
H	-1.374003	1.806011	-2.813517
H	-2.670206	2.539963	-1.848139
H	-1.024409	2.440508	-1.189656

**D**	G781.705021		
C	2.001237	2.437806	0.132487
C	3.071659	1.445778	-0.393592
C	2.465794	0.299113	-1.167172
C	-0.338815	-0.126261	2.089637
C	0.721838	0.597443	1.301779
C	0.868305	1.936807	1.011895
H	2.510453	3.250254	0.678102
H	3.753319	2.032760	-1.030169
C	2.576878	-1.016183	-0.874692
C	-1.226381	-1.161965	1.208432
H	1.578187	-0.025492	1.052772
C	-0.580332	-1.288423	-0.169554
C	1.605946	-2.026219	-1.459704
H	2.112632	-2.979153	-1.671712
C	-0.064366	3.010109	1.498822
H	-0.408380	3.660847	0.682344
H	-0.931955	2.628768	2.044408
H	0.497387	3.660320	2.188892
H	1.518376	2.945275	-0.723904
H	3.669440	1.080076	0.451891
C	3.535514	-1.573687	0.157656
H	3.021424	-1.912960	1.073182
H	4.045753	-2.458620	-0.250191
H	4.308169	-0.859751	0.459019
H	1.781799	0.593262	-1.971412
H	1.185081	-1.677670	-2.412400
H	0.169452	-0.713925	2.866318
H	-1.030622	0.553239	2.602164
H	0.130058	0.330937	-0.169820
C	0.475255	-2.340938	-0.430936
H	-0.089878	-3.224838	-0.781429
H	0.925003	-2.656249	0.519175
C	-1.311079	-2.504224	1.966231
H	-1.824966	-2.345662	2.923384
H	-1.885775	-3.247781	1.398224
H	-0.325158	-2.928513	2.192349
C	-2.664555	-0.591485	1.047027
H	-3.293800	-1.365024	0.579449
H	-3.081969	-0.422516	2.050255

C	-2.722637	0.701442	0.228998
H	-3.762039	1.049881	0.143921
H	-2.177781	1.497334	0.754798
C	-0.876665	-0.341484	-1.141590
H	-0.342706	-0.414477	-2.092819
C	-2.121496	0.535851	-1.184569
C	-3.120100	-0.193171	-2.127654
H	-2.687276	-0.349892	-3.125223
H	-3.415192	-1.172397	-1.729328
H	-4.023936	0.419368	-2.245607
C	-1.772084	1.910709	-1.794389
H	-1.374003	1.806011	-2.813517
H	-2.670206	2.539963	-1.848139
H	-1.024409	2.440508	-1.189656

D-E-TS G781.706629, T-955

C	2.020410	2.436204	0.117066
C	3.085099	1.429857	-0.396141
C	2.475467	0.286075	-1.171409
C	-0.327069	-0.106357	2.079134
C	0.733849	0.616862	1.301399
C	0.879338	1.947803	0.995875
H	2.536986	3.246110	0.659329
H	3.778417	2.006100	-1.029875
C	2.565254	-1.029482	-0.871631
C	-1.218854	-1.162268	1.207063
H	1.572370	-0.016837	1.024825
C	-0.586411	-1.294222	-0.168432
C	1.592181	-2.029982	-1.469769
H	2.093992	-2.984584	-1.686243
C	-0.048317	3.030968	1.474355
H	-0.393183	3.673131	0.651422
H	-0.917005	2.657419	2.024307
H	0.514069	3.687890	2.157129
H	1.547678	2.941777	-0.745854
H	3.671651	1.061438	0.456056
C	3.500181	-1.595013	0.178203
H	2.969168	-1.910695	1.092645
H	3.996582	-2.495494	-0.212241
H	4.283666	-0.892902	0.479192
H	1.806729	0.584428	-1.986853
H	1.177465	-1.671731	-2.421453
H	0.176973	-0.696437	2.856651
H	-1.027029	0.569329	2.585312
H	0.094475	0.366968	-0.238851
C	0.453201	-2.351156	-0.449954
H	-0.129233	-3.212813	-0.830126
H	0.891720	-2.704911	0.491533
C	-1.287554	-2.495007	1.982719
H	-1.793540	-2.326748	2.942249
H	-1.863048	-3.249253	1.429792
H	-0.296964	-2.910051	2.205360
C	-2.660874	-0.594249	1.057583
H	-3.292777	-1.370074	0.597647

H	-3.067654	-0.426492	2.065247
C	-2.729331	0.698601	0.240474
H	-3.772056	1.037391	0.156460
H	-2.190959	1.498682	0.766872
C	-0.855845	-0.307741	-1.121771
H	-0.335024	-0.389616	-2.079556
C	-2.128087	0.539747	-1.173194
C	-3.108252	-0.219034	-2.109777
H	-2.673507	-0.367276	-3.107879
H	-3.375077	-1.205302	-1.708131
H	-4.029676	0.366476	-2.228547
C	-1.811626	1.918604	-1.789240
H	-1.408506	1.817965	-2.806846
H	-2.724591	2.525475	-1.849447
H	-1.078664	2.469388	-1.185441

**E**	G781.726404		
C	3.105330	1.927624	-0.399607
C	3.624277	0.479078	-0.577661
C	2.552377	-0.384630	-1.188897
C	-0.106560	1.036575	1.705932
C	1.277259	1.116941	1.112230
C	1.749865	2.070930	0.282632
H	3.859218	2.528396	0.136761
H	4.513778	0.516287	-1.227991
C	2.143130	-1.610502	-0.804470
C	-0.748298	-0.376684	1.447445
H	1.924321	0.269474	1.344590
C	-0.697288	-0.781569	0.014975
C	0.889193	-2.186849	-1.426775
H	1.002436	-3.251929	-1.681047
C	0.999764	3.329874	-0.089598
H	0.716915	3.318525	-1.156148
H	0.097795	3.508160	0.507654
H	1.650842	4.208131	0.038136
H	3.022501	2.392066	-1.397489
H	3.966992	0.068609	0.382648
C	2.824598	-2.464314	0.239200
H	2.106770	-2.927240	0.933325
H	3.358313	-3.296879	-0.246179
H	3.560717	-1.912241	0.834273
H	1.987810	0.097506	-1.997142
H	0.626180	-1.652286	-2.348753
H	-0.097395	1.165863	2.801198
H	-0.748173	1.830123	1.311063
H	-0.784667	1.171009	-0.816319
C	-0.359916	-2.149021	-0.440363
H	-1.208025	-2.516824	-1.043061
H	-0.189181	-2.839185	0.389313
C	-0.186557	-1.402643	2.449237
H	-0.341034	-1.025336	3.467252
H	-0.691100	-2.374574	2.383085
H	0.889415	-1.558238	2.311664
C	-2.339807	-0.279783	1.678365

H	-2.761498	-1.293797	1.630858
H	-2.442344	0.056343	2.720970
C	-3.056539	0.662032	0.714153
H	-4.138823	0.608428	0.910800
H	-2.763681	1.704181	0.906029
C	-1.209702	0.173652	-0.971924
H	-1.025953	-0.147947	-2.002977
C	-2.810695	0.323525	-0.772193
C	-3.527586	-0.973533	-1.190470
H	-3.247163	-1.271699	-2.210362
H	-3.323809	-1.818263	-0.519810
H	-4.612985	-0.808620	-1.184493
C	-3.252912	1.479647	-1.686882
H	-3.052257	1.254057	-2.742640
H	-4.334788	1.640063	-1.580273
H	-2.740721	2.415742	-1.426912

**E**	G781.726397		
C	3.104819	1.927844	-0.399726
C	3.623712	0.479306	-0.577951
C	2.551775	-0.384460	-1.189073
C	-0.106532	1.036336	1.706293
C	1.277254	1.117030	1.112579
C	1.749564	2.071093	0.282915
H	3.858870	2.528625	0.136403
H	4.513088	0.516518	-1.228454
C	2.142710	-1.610395	-0.804642
C	-0.748010	-0.376981	1.447523
H	1.924441	0.269607	1.344749
C	-0.697004	-0.781777	0.015064
C	0.888849	-2.187077	-1.426928
H	1.002395	-3.252110	-1.681287
C	0.999189	3.329931	-0.089166
H	0.715278	3.318142	-1.155440
H	0.097818	3.508515	0.508900
H	1.650443	4.208200	0.037523
H	3.021664	2.392304	-1.397576
H	3.966639	0.068806	0.382271
C	2.824454	-2.464089	0.238958
H	2.106768	-2.927589	0.932841
H	3.358726	-3.296233	-0.246532
H	3.560160	-1.911750	0.834297
H	1.987173	0.097624	-1.997327
H	0.625700	-1.652563	-2.348897
H	-0.097376	1.165419	2.801583
H	-0.748246	1.829869	1.311559
H	-0.783983	1.170834	-0.816083
C	-0.359948	-2.149387	-0.440348
H	-1.208365	-2.517183	-1.042603
H	-0.188975	-2.839462	0.389352
C	-0.186342	-1.403000	2.449303
H	-0.340853	-1.025729	3.467325
H	-0.690891	-2.374919	2.383084
H	0.889632	-1.558582	2.311744

C	-2.339643	-0.280334	1.678170
H	-2.761236	-1.294367	1.630231
H	-2.442319	0.055387	2.720895
C	-3.056236	0.661795	0.714179
H	-4.138544	0.608122	0.910669
H	-2.763398	1.703877	0.906437
C	-1.209217	0.173580	-0.971804
H	-1.025457	-0.147964	-2.002865
C	-2.810195	0.323718	-0.772235
C	-3.527303	-0.973065	-1.191016
H	-3.246695	-1.271060	-2.210905
H	-3.323929	-1.818004	-0.520496
H	-4.612661	-0.807873	-1.185274
C	-3.252053	1.480213	-1.686637
H	-3.051308	1.254898	-2.742437
H	-4.333907	1.640828	-1.580118
H	-2.739692	2.416118	-1.426318

E-F-TS G781.710192, T-771

C	2.986813	2.026121	-0.485937
C	3.588782	0.601048	-0.515164
C	2.564547	-0.382133	-1.014334
C	-0.311710	1.290202	1.569409
C	1.094205	1.299218	1.021645
C	1.593491	2.174863	0.120854
H	3.687719	2.710352	0.020980
H	4.480502	0.616226	-1.164130
C	2.432268	-1.689272	-0.690731
C	-1.017143	-0.126094	1.541982
H	1.745044	0.495916	1.377686
C	-0.647009	-0.785732	0.201856
C	1.087490	-2.351808	-1.036267
H	1.142230	-3.447676	-1.020359
C	0.840328	3.376468	-0.407877
H	0.589539	3.246230	-1.474100
H	-0.081264	3.604689	0.138117
H	1.479549	4.270968	-0.355418
H	2.928181	2.392887	-1.525121
H	3.943420	0.301205	0.482734
C	3.404975	-2.495077	0.131543
H	2.928494	-2.964869	1.007008
H	3.816650	-3.317539	-0.473867
H	4.247119	-1.892134	0.488093
H	1.780389	0.048255	-1.646385
H	0.688727	-2.019011	-1.999814
H	-0.333879	1.606091	2.626489
H	-0.934769	2.005911	1.023260
H	-0.801711	0.838069	-1.105617
C	0.328528	-1.814988	0.144400
H	-1.002173	-2.056860	0.238432
H	0.684818	-2.175847	1.111536
C	-0.562573	-0.937920	2.771186
H	-0.954804	-0.456578	3.675201
H	-0.952503	-1.966776	2.759162

H	0.529993	-0.975596	2.877511
C	-2.562666	0.058866	1.602422
H	-3.021053	-0.922908	1.802848
H	-2.791191	0.684524	2.477797
C	-3.182558	0.681837	0.342678
H	-4.277675	0.689712	0.446296
H	-2.880029	1.737549	0.257128
C	-1.247132	-0.167730	-1.034129
H	-0.947167	-0.709774	-1.940540
C	-2.799750	-0.048189	-0.967420
C	-3.431746	-1.456017	-1.029084
H	-3.136699	-1.981999	-1.948258
H	-3.154230	-2.092125	-0.173666
H	-4.527160	-1.384166	-1.022409
C	-3.272765	0.773875	-2.181754
H	-3.007020	0.279566	-3.127011
H	-4.364382	0.893457	-2.163541
H	-2.822921	1.777010	-2.181714

**F**	G781.717481		
C	2.841848	2.160137	-0.454238
C	3.455294	0.746062	-0.539098
C	2.434499	-0.250184	-0.992569
C	-0.323715	1.147089	1.657949
C	1.061082	1.280843	1.090699
C	1.473244	2.224156	0.213921
H	3.554178	2.839239	0.042044
H	4.301759	0.756431	-1.249351
C	2.431114	-1.597182	-0.735527
C	-0.963642	-0.283380	1.549734
H	1.775674	0.515646	1.407707
C	-0.672701	-1.001107	0.181078
C	1.170982	-2.380717	-1.153526
H	1.336427	-3.463926	-1.138373
C	0.632821	3.393742	-0.247795
H	0.334043	3.274720	-1.303152
H	-0.270682	3.554746	0.348862
H	1.224561	4.320822	-0.202327
H	2.731046	2.552310	-1.479436
H	3.884737	0.430690	0.423759
C	3.498722	-2.339369	0.024884
H	3.073225	-3.123094	0.666447
H	4.164556	-2.846515	-0.690703
H	4.115163	-1.681071	0.647356
H	1.606818	0.144642	-1.587660
H	0.731924	-2.058040	-2.100976
H	-0.332487	1.404438	2.731403
H	-0.984913	1.872397	1.174186
H	-0.694408	0.764497	-1.036328
C	0.534054	-1.833551	0.057827
H	-1.315658	-1.935325	0.239864
H	0.910632	-2.243239	0.999205
C	-0.496836	-1.141622	2.742658
H	-0.900805	-0.726846	3.674862

H	-0.852432	-2.180832	2.668934
H	0.597545	-1.155390	2.850727
C	-2.512567	-0.129740	1.614854
H	-2.958776	-1.131924	1.726557
H	-2.768793	0.420341	2.533094
C	-3.128067	0.588393	0.400343
H	-4.224263	0.583331	0.495950
H	-2.835066	1.649976	0.410333
C	-1.194809	-0.215481	-1.028572
H	-0.935378	-0.716499	-1.973231
C	-2.736267	-0.016923	-0.973750
C	-3.458216	-1.364075	-1.201760
H	-3.155906	-1.818454	-2.156253
H	-3.267259	-2.098191	-0.404156
H	-4.545255	-1.212360	-1.232300
C	-3.140200	0.959488	-2.096660
H	-2.891770	0.552811	-3.087742
H	-4.221952	1.149741	-2.076470
H	-2.626329	1.925475	-1.985538

F G781.717484

C	2.841919	2.160058	-0.454314
C	3.455289	0.745969	-0.539075
C	2.434410	-0.250256	-0.992460
C	-0.323717	1.147203	1.657878
C	1.061070	1.280963	1.090622
C	1.473269	2.224192	0.213770
H	3.554251	2.839143	0.041989
H	4.301772	0.756244	-1.249295
C	2.431049	-1.597225	-0.735413
C	-0.963635	-0.283277	1.549735
H	1.775649	0.515752	1.407630
C	-0.672740	-1.001078	0.181137
C	1.170892	-2.380845	-1.153420
H	1.336530	-3.464024	-1.138157
C	0.633027	3.393870	-0.247940
H	0.335978	3.275915	-1.303890
H	-0.271479	3.553896	0.347464
H	1.224300	4.321168	-0.200487
H	2.731194	2.552225	-1.479524
H	3.884678	0.430591	0.423814
C	3.498685	-2.339520	0.024835
H	3.073128	-3.122613	0.667133
H	4.163832	-2.847440	-0.690833
H	4.115815	-1.681177	0.646580
H	1.606665	0.144590	-1.587444
H	0.731841	-2.058223	-2.100876
H	-0.332515	1.404614	2.731312
H	-0.984934	1.872467	1.174073
H	-0.694421	0.764476	-1.036386
C	0.533993	-1.833697	0.057924
H	-1.315745	-1.935257	0.239999
H	0.910468	-2.243400	0.999328
C	-0.496829	-1.141457	2.742713

H	-0.900862	-0.726669	3.674879
H	-0.852364	-2.180688	2.668992
H	0.597551	-1.155149	2.850803
C	-2.512576	-0.129592	1.614870
H	-2.958801	-1.131760	1.726649
H	-2.768767	0.420557	2.533078
C	-3.128071	0.588453	0.400306
H	-4.224268	0.583405	0.495919
H	-2.835076	1.650039	0.410214
C	-1.194791	-0.215522	-1.028551
H	-0.935382	-0.716573	-1.973196
C	-2.736274	-0.016951	-0.973749
C	-3.458215	-1.364116	-1.201695
H	-3.155939	-1.818516	-2.156189
H	-3.267231	-2.098222	-0.404087
H	-4.545258	-1.212414	-1.232196
C	-3.140188	0.959389	-2.096723
H	-2.891752	0.552643	-3.087776
H	-4.221941	1.149639	-2.076559
H	-2.626317	1.925383	-1.985668

F-G-TS G781.719672, T-133

C	2.838061	2.153513	-0.466234
C	3.401361	0.724819	-0.590783
C	2.331965	-0.226115	-1.019708
C	-0.261107	1.068806	1.698009
C	1.109418	1.229037	1.104471
C	1.500024	2.212193	0.259698
H	3.583537	2.812959	0.006661
H	4.213517	0.702115	-1.341737
C	2.295907	-1.575196	-0.736672
C	-0.909775	-0.351981	1.537051
H	1.831625	0.454694	1.377289
C	-0.664352	-1.032459	0.139492
C	1.115339	-2.400214	-1.283235
H	1.339233	-3.469901	-1.318030
C	0.652879	3.404011	-0.121211
H	0.298274	3.320116	-1.162807
H	-0.216329	3.554886	0.526495
H	1.258600	4.322144	-0.079628
H	2.686912	2.559976	-1.480671
H	3.863509	0.382210	0.346468
C	3.371797	-2.291406	0.048941
H	3.024733	-3.267698	0.407674
H	4.242212	-2.472451	-0.598730
H	3.716017	-1.710416	0.914005
H	1.530177	0.183841	-1.638757
H	0.681449	-2.028133	-2.213255
H	-0.239419	1.279003	2.781510
H	-0.929267	1.819235	1.265058
H	-0.658322	0.794809	-1.002829
C	0.547043	-1.882149	-0.039395
H	-1.342994	-1.929893	0.171493
H	0.873634	-2.390210	0.871147

C	-0.433842	-1.259948	2.688944
H	-0.808913	-0.873425	3.645296
H	-0.809370	-2.289427	2.585029
H	0.662636	-1.297207	2.769856
C	-2.455995	-0.181832	1.634659
H	-2.912664	-1.182896	1.706681
H	-2.693572	0.329375	2.580164
C	-3.079434	0.598399	0.462373
H	-4.174350	0.600782	0.572536
H	-2.775502	1.655556	0.518965
C	-1.175942	-0.175610	-1.026350
H	-0.943870	-0.639869	-1.997224
C	-2.712979	0.056043	-0.945213
C	-3.472820	-1.258251	-1.235764
H	-3.183881	-1.674265	-2.211742
H	-3.302903	-2.034266	-0.474957
H	-4.554887	-1.072931	-1.258434
C	-3.103960	1.098910	-2.011626
H	-2.876273	0.737734	-3.025158
H	-4.180297	1.314935	-1.969463
H	-2.564777	2.045191	-1.857578

**G**	G781.718785		
C	3.062078	2.021053	-0.479738
C	3.429254	0.542157	-0.680834
C	2.162078	-0.208868	-0.948411
C	-0.087827	0.950704	1.598901
C	1.302349	1.060870	1.037560
C	1.754246	2.116181	0.290322
H	3.873091	2.593038	-0.002178
H	4.111328	0.404762	-1.540303
C	1.950931	-1.566703	-0.623266
C	-0.832667	-0.423278	1.469900
H	2.014494	0.294174	1.351660
C	-0.770843	-1.095151	0.052166
C	0.951795	-2.347386	-1.555231
H	1.275453	-3.371024	-1.747016
C	0.960928	3.370126	0.038091
H	0.462304	3.321844	-0.945898
H	0.200020	3.569176	0.798367
H	1.638425	4.235342	-0.008219
H	2.900867	2.491870	-1.463840
H	3.951132	0.123730	0.189619
C	3.036683	-2.352242	0.097255
H	2.731228	-3.396512	0.230492
H	3.976246	-2.349217	-0.470895
H	3.238985	-1.939215	1.095961
H	1.434992	0.271715	-1.606697
H	0.606514	-1.806638	-2.435496
H	-0.054195	1.183989	2.678237
H	-0.707237	1.729627	1.146012
H	-0.661791	0.786925	-1.041618
C	0.437508	-1.980895	-0.238872
H	-1.536637	-1.895857	0.104808

H	0.553941	-2.761712	0.517573
C	-0.328774	-1.384128	2.564371
H	-0.554654	-0.978471	3.559627
H	-0.816455	-2.367438	2.493080
H	0.758861	-1.541050	2.516409
C	-2.345922	-0.128426	1.706949
H	-2.873600	-1.092736	1.781570
H	-2.469437	0.363361	2.684559
C	-2.994252	0.742568	0.612925
H	-4.076435	0.809948	0.801410
H	-2.621504	1.776615	0.693871
C	-1.265461	-0.135274	-1.040148
H	-1.156922	-0.584850	-2.039556
C	-2.764771	0.243574	-0.840480
C	-3.673596	-0.971311	-1.140125
H	-3.479625	-1.370312	-2.146181
H	-3.546569	-1.792853	-0.421794
H	-4.729324	-0.671741	-1.097214
C	-3.118503	1.374552	-1.827476
H	-2.991397	1.047693	-2.869855
H	-4.163444	1.690612	-1.702875
H	-2.480061	2.256465	-1.666775

**G**	G781.718782		
C	3.061452	2.021765	-0.479312
C	3.429184	0.543015	-0.680813
C	2.162235	-0.208372	-0.948448
C	-0.087872	0.950104	1.599521
C	1.302201	1.060770	1.038021
C	1.753588	2.116283	0.290777
H	3.872273	2.593901	-0.001606
H	4.111164	0.406202	-1.540454
C	1.951397	-1.566293	-0.623485
C	-0.832651	-0.423867	1.469860
H	2.014725	0.294399	1.352080
C	-0.770629	-1.095255	0.051920
C	0.952346	-2.346954	-1.555666
H	1.276105	-3.370553	-1.747501
C	0.959623	3.369814	0.038519
H	0.459757	3.320528	-0.944804
H	0.199506	3.569157	0.799530
H	1.636712	4.235242	-0.009322
H	2.900100	2.492738	-1.463315
H	3.951382	0.124556	0.189424
C	3.037253	-2.351739	0.096955
H	2.731823	-3.395994	0.230396
H	3.976702	-2.348770	-0.471378
H	3.239720	-1.938560	1.095567
H	1.434990	0.272146	-1.606614
H	0.607248	-1.806091	-2.435933
H	-0.054049	1.182714	2.678984
H	-0.707431	1.729258	1.147225
H	-0.661688	0.787129	-1.041292
C	0.437866	-1.980696	-0.239345

H	-1.536280	-1.896116	0.104242
H	0.554431	-2.761660	0.516943
C	-0.328823	-1.385101	2.564050
H	-0.554859	-0.979834	3.559427
H	-0.816412	-2.368423	2.492330
H	0.758833	-1.541886	2.516156
C	-2.345944	-0.129176	1.706840
H	-2.873560	-1.093541	1.781138
H	-2.469572	0.362316	2.684585
C	-2.994224	0.742097	0.613010
H	-4.076419	0.809420	0.801445
H	-2.621483	1.776120	0.694248
C	-1.265284	-0.135123	-1.040169
H	-1.156573	-0.584413	-2.039685
C	-2.764632	0.243525	-0.840523
C	-3.673342	-0.971334	-1.140583
H	-3.479294	-1.370003	-2.146757
H	-3.546282	-1.793095	-0.422510
H	-4.729102	-0.671871	-1.097625
C	-3.118391	1.374766	-1.827205
H	-2.991189	1.048224	-2.869676
H	-4.163365	1.690705	-1.702578
H	-2.480029	2.256680	-1.666205

G-H-TS G781.722066, T-335

C	3.407195	1.692053	-0.671967
C	3.465579	0.166549	-0.838818
C	2.014948	-0.302703	-0.805645
C	0.095331	1.044374	1.218328
C	1.530538	0.899353	0.755481
C	2.178066	1.988945	0.144401
H	4.316725	2.143994	-0.242701
H	3.952518	-0.138829	-1.776899
C	1.678486	-1.683470	-0.449960
C	-0.801151	-0.216556	1.412143
H	2.175347	0.214152	1.320267
C	-0.959074	-1.101649	0.131038
C	0.758417	-2.420235	-1.464580
H	1.031218	-3.455857	-1.670533
C	1.571651	3.346459	0.035983
H	0.829797	3.347860	-0.784359
H	1.038234	3.646231	0.946050
H	2.328382	4.096099	-0.226957
H	3.267943	2.190851	-1.647722
H	4.032159	-0.293426	-0.019949
C	2.736590	-2.499593	0.282833
H	2.362681	-3.510423	0.483439
H	3.660233	-2.597861	-0.303080
H	2.997145	-2.053940	1.255840
H	1.379498	0.139925	-1.580186
H	0.449688	-1.853753	-2.342358
H	0.113167	1.569357	2.190547
H	-0.409435	1.727112	0.525989
H	-0.801097	0.527642	-1.321134

C	0.208388	-2.055142	-0.136985
H	-1.765449	-1.813369	0.386089
H	0.208831	-2.886240	0.573262
C	-0.286598	-1.044682	2.604270
H	-0.318058	-0.451976	3.529100
H	-0.908802	-1.936965	2.761550
H	0.748681	-1.386595	2.464067
C	-2.231584	0.313136	1.738707
H	-2.853865	-0.551503	2.016805
H	-2.188668	0.956655	2.631930
C	-2.894136	1.081696	0.580194
H	-3.929323	1.330871	0.858995
H	-2.391557	2.053750	0.440935
C	-1.497211	-0.287482	-1.056252
H	-1.568608	-0.928557	-1.948058
C	-2.904196	0.319502	-0.773380
C	-3.974284	-0.797147	-0.746344
H	-3.964712	-1.373051	-1.682596
H	-3.836428	-1.503628	0.083059
H	-4.975176	-0.359094	-0.632364
C	-3.254292	1.303512	-1.908165
H	-3.280972	0.792713	-2.881606
H	-4.240624	1.759132	-1.743226
H	-2.515441	2.117190	-1.971891

**H**	G781.744560		
C	-3.461237	-1.087603	-1.340745
C	-3.212352	0.432792	-1.280777
C	-1.780351	0.533319	-0.702779
C	-0.280565	-1.278861	0.557651
C	-1.689331	-0.694041	0.259782
C	-2.656156	-1.668521	-0.252418
H	-4.497995	-1.453161	-1.380795
H	-3.317277	0.915266	-2.258304
C	-1.406093	1.850539	-0.031250
C	0.738776	-0.350063	1.274189
H	-2.151427	-0.395287	1.238181
C	1.122232	0.938669	0.473482
C	-0.444706	2.762891	-0.767903
H	-0.593955	3.840638	-0.696057
C	-2.753763	-3.071067	0.179218
H	-1.959971	-3.622757	-0.363826
H	-2.513393	-3.197341	1.243342
H	-3.711879	-3.535107	-0.077696
H	-2.973748	-1.516809	-2.250420
H	-3.948462	0.885200	-0.603076
C	-2.497272	2.494181	0.816547
H	-2.096501	3.358322	1.360134
H	-3.337094	2.855788	0.205769
H	-2.907460	1.802218	1.571572
H	-1.070591	0.330288	-1.524779
H	-0.088324	2.432280	-1.743851
H	-0.391073	-2.178843	1.181173
H	0.121815	-1.615543	-0.408027

H	1.056632	0.088180	-1.544964
C	0.025360	2.016490	0.457421
H	1.923700	1.402682	1.074245
H	0.072827	2.643859	1.351470
C	0.201860	0.010046	2.670272
H	0.028686	-0.893274	3.273420
H	0.920677	0.639447	3.212142
H	-0.741109	0.574653	2.621810
C	2.048748	-1.186573	1.438413
H	2.725505	-0.611593	2.088841
H	1.823082	-2.117812	1.982944
C	2.755344	-1.510270	0.112691
H	3.696805	-2.039665	0.326097
H	2.146726	-2.219320	-0.474474
C	1.771921	0.605932	-0.882362
H	2.027778	1.544885	-1.395588
C	3.056239	-0.261699	-0.757480
C	4.209789	0.559897	-0.136269
H	4.413612	1.458480	-0.735341
H	4.000592	0.886169	0.890982
H	5.130534	-0.038943	-0.105352
C	3.487235	-0.716611	-2.166937
H	3.705440	0.147378	-2.810940
H	4.393792	-1.336474	-2.120486
H	2.697985	-1.310477	-2.652626

**C3**	G781.708043		
C	3.091212	1.505906	0.577287
C	3.361816	1.170262	-0.931344
C	2.416211	0.133461	-1.486352
C	-0.432359	0.679670	2.091342
C	1.034393	0.756642	1.772790
C	1.622856	1.625232	0.899741
H	3.546070	0.737043	1.215285
H	3.278328	2.087422	-1.529810
C	2.325702	-1.158762	-1.096226
C	-1.095812	-0.603465	1.468204
H	1.681974	0.020276	2.258499
C	-0.524585	-0.759530	0.008062
C	1.163751	-2.011238	-1.565732
H	1.499293	-3.030690	-1.813053
C	0.834818	2.668923	0.144582
H	0.287704	2.207238	-0.701279
H	0.088380	3.169829	0.772801
H	1.490100	3.434870	-0.286485
H	3.597079	2.458226	0.805459
H	4.409498	0.842382	-1.010902
C	3.268376	-1.794261	-0.099942
H	3.496585	-2.830465	-0.387875
H	4.216684	-1.251561	-0.015478
H	2.826023	-1.838665	0.908407
H	1.693576	0.485871	-2.230708
H	0.727526	-1.595505	-2.486558
H	-0.593416	0.654362	3.180128

H	-0.942684	1.577877	1.722866
H	0.452689	-0.110583	-0.005945
C	0.094511	-2.140110	-0.456596
H	-0.717490	-2.807830	-0.777047
H	0.552578	-2.597276	0.425785
C	-0.771153	-1.819981	2.358844
H	0.308081	-1.967619	2.495673
H	-1.207246	-1.664601	3.354504
H	-1.195152	-2.748540	1.954753
C	-2.635396	-0.421292	1.433065
H	-3.102020	-1.377963	1.145843
H	-2.986748	-0.207017	2.452945
C	-3.100978	0.695267	0.489894
H	-4.198195	0.759333	0.484267
H	-2.746144	1.672701	0.848440
C	-1.274475	-0.142112	-1.033001
H	-0.863413	-0.231004	-2.046959
C	-2.602783	0.492801	-0.956029
C	-3.479997	-0.611621	-1.688057
H	-3.154571	-0.777319	-2.721910
H	-3.477215	-1.567290	-1.152382
H	-4.507049	-0.222217	-1.705524
C	-2.682781	1.793783	-1.791819
H	-2.349293	1.635475	-2.826172
H	-3.723298	2.142318	-1.818299
H	-2.068197	2.583464	-1.341533

C3-I-TS G781.705941, T1367

C	2.931591	1.519805	0.704143
C	3.102350	1.348830	-0.850812
C	2.247615	0.236104	-1.400071
C	-0.489733	0.248901	2.197791
C	0.900498	0.418739	1.637279
C	1.492797	1.564455	1.145681
H	3.443790	0.690525	1.206718
H	2.845863	2.293215	-1.349733
C	2.377164	-1.081243	-1.122749
C	-1.300067	-0.833407	1.378783
H	1.572942	-0.431870	1.792682
C	-0.625066	-0.966737	-0.032305
C	1.273865	-2.053088	-1.498431
H	1.690746	-3.051449	-1.696385
C	0.767205	2.861276	0.936619
H	0.957544	3.250138	-0.075195
H	-0.313006	2.798135	1.095434
H	1.165723	3.617595	1.631403
H	3.434045	2.453402	0.999583
H	4.173092	1.178645	-1.035505
C	3.531527	-1.666581	-0.343825
H	3.969013	-2.508547	-0.901299
H	4.332513	-0.945769	-0.148715
H	3.206370	-2.079793	0.625071
H	1.388725	0.545803	-2.004185
H	0.773102	-1.741226	-2.426155

H	-0.423370	-0.066251	3.249349
H	-1.025195	1.205557	2.198664
H	0.369793	-0.008493	0.127432
C	0.253985	-2.204040	-0.339348
H	-0.424298	-3.044402	-0.551817
H	0.799983	-2.479565	0.570466
C	-1.296090	-2.171929	2.148288
H	-0.285753	-2.494099	2.430854
H	-1.869131	-2.051189	3.077397
H	-1.766284	-2.978512	1.570805
C	-2.769931	-0.364844	1.214537
H	-3.367496	-1.199632	0.813413
H	-3.177736	-0.146161	2.212380
C	-2.917550	0.864464	0.311229
H	-3.971554	1.168740	0.248155
H	-2.386340	1.722744	0.751292
C	-1.203083	-0.309185	-1.116200
H	-0.770084	-0.489296	-2.106142
C	-2.376861	0.609344	-1.113263
C	-3.447540	-0.140916	-1.979930
H	-3.075625	-0.355319	-2.990183
H	-3.760079	-1.084072	-1.515997
H	-4.327075	0.510281	-2.070981
C	-2.015185	1.936967	-1.829036
H	-1.670368	1.760880	-2.857129
H	-2.904833	2.579001	-1.875281
H	-1.229980	2.479491	-1.286680

I G781.720223

C	3.319252	1.449202	0.022235
C	3.198267	0.623037	-1.382418
C	2.164881	-0.455364	-1.403811
C	-0.289129	1.231407	1.513901
C	1.140213	0.932012	1.083169
C	1.978737	1.898320	0.417760
H	3.757050	0.768699	0.760343
H	3.011452	1.352572	-2.179078
C	2.206063	-1.622008	-0.694454
C	-1.202988	-0.043566	1.554019
H	1.707598	0.294763	1.777001
C	-0.878760	-0.968177	0.354363
C	0.998426	-2.530567	-0.652037
H	1.326581	-3.570758	-0.515891
C	1.416230	3.146803	-0.161201
H	2.155732	3.725095	-0.723478
H	0.568171	2.901187	-0.824627
H	0.985266	3.774453	0.634782
H	4.002602	2.282985	-0.175741
H	4.214078	0.228797	-1.519432
C	3.372170	-2.026846	0.168117
H	3.686934	-3.048481	-0.091295
H	4.245586	-1.373303	0.073584
H	3.081863	-2.062786	1.230867
H	1.268714	-0.266598	-2.001249

H	0.446278	-2.489242	-1.598965
H	-0.290657	1.718630	2.501640
H	-0.721440	1.950123	0.808308
H	1.077093	0.214488	0.167158
C	0.058930	-2.166531	0.542019
H	-0.560510	-3.052214	0.756603
H	0.679107	-2.032714	1.437953
C	-1.026095	-0.757999	2.913831
H	0.023990	-0.977284	3.151859
H	-1.412573	-0.119039	3.719427
H	-1.581215	-1.704873	2.943256
C	-2.683092	0.414714	1.432140
H	-3.326238	-0.464558	1.594991
H	-2.909482	1.122170	2.245335
C	-3.009946	1.055895	0.078412
H	-4.073389	1.333959	0.045676
H	-2.460373	2.005099	-0.030626
C	-1.534260	-0.808681	-0.818838
H	-1.299682	-1.488508	-1.646518
C	-2.681905	0.130982	-1.122587
C	-3.911367	-0.761978	-1.453720
H	-3.691012	-1.459788	-2.273707
H	-4.219751	-1.352917	-0.581117
H	-4.758721	-0.134371	-1.762878
C	-2.333953	0.980476	-2.369123
H	-2.129346	0.345799	-3.243299
H	-3.168698	1.647590	-2.626048
H	-1.445048	1.604231	-2.185504

E G781.726401

C	3.105075	1.927604	-0.399877
C	3.623910	0.479039	-0.577866
C	2.551934	-0.384739	-1.188916
C	-0.106514	1.036587	1.706082
C	1.277206	1.117006	1.112204
C	1.749728	2.071009	0.282577
H	3.859093	2.528404	0.136279
H	4.513349	0.516112	-1.228290
C	2.142851	-1.610637	-0.804404
C	-0.748336	-0.376641	1.447587
H	1.924235	0.269442	1.344301
C	-0.697111	-0.781615	0.015128
C	0.888912	-2.187253	-1.426614
H	1.002312	-3.252347	-1.680767
C	0.999779	3.330137	-0.089293
H	0.718135	3.319770	-1.156156
H	0.097117	3.507725	0.507125
H	1.650560	4.208394	0.040050
H	3.022078	2.391961	-1.397787
H	3.966705	0.068651	0.382453
C	2.824503	-2.464341	0.239231
H	2.106823	-2.926993	0.933696
H	3.357896	-3.297110	-0.246154
H	3.560920	-1.912274	0.833935

H	1.987298	0.097301	-1.997170
H	0.625871	-1.652864	-2.348686
H	-0.097232	1.165776	2.801361
H	-0.748171	1.830178	1.311368
H	-0.784242	1.170939	-0.816218
C	-0.359903	-2.149219	-0.440096
H	-1.208309	-2.517033	-1.042402
H	-0.189117	-2.839248	0.389684
C	-0.186816	-1.402596	2.449489
H	-0.341425	-1.025246	3.467471
H	-0.691393	-2.374503	2.383301
H	0.889175	-1.558232	2.312087
C	-2.339866	-0.279571	1.678292
H	-2.761679	-1.293543	1.630862
H	-2.442448	0.056679	2.720853
C	-3.056377	0.662215	0.713903
H	-4.138699	0.608724	0.910383
H	-2.763475	1.704363	0.905716
C	-1.209243	0.173585	-0.971882
H	-1.025420	-0.148041	-2.002904
C	-2.810356	0.323565	-0.772366
C	-3.527249	-0.973482	-1.190620
H	-3.246620	-1.271825	-2.210404
H	-3.323729	-1.818131	-0.519780
H	-4.612634	-0.808461	-1.184924
C	-3.252320	1.479628	-1.687257
H	-3.051517	1.253903	-2.742956
H	-4.334201	1.640132	-1.580835
H	-2.740100	2.415710	-1.427304

E-J-TS G781.712808, T-271

C	2.990430	2.027784	-0.434242
C	3.445889	0.578110	-0.760028
C	2.302001	-0.250223	-1.291978
C	0.046840	0.873885	1.896919
C	1.395006	1.156837	1.261110
C	1.696498	2.121867	0.367318
H	3.806261	2.566247	0.075895
H	4.251650	0.642945	-1.510488
C	1.958057	-1.515972	-0.971366
C	-0.255888	-0.554462	1.444601
H	2.147772	0.389919	1.451198
C	-0.746353	-0.846816	0.141842
C	0.683030	-2.113886	-1.543644
H	0.855438	-3.144153	-1.893227
C	0.815730	3.303686	0.043597
H	0.476178	3.272709	-1.004415
H	-0.062948	3.395086	0.694140
H	1.393766	4.234948	0.146859
H	2.830731	2.568505	-1.381692
H	3.900635	0.108360	0.123996
C	2.775226	-2.427492	-0.083108
H	2.140228	-3.054496	0.559251
H	3.363292	-3.124313	-0.701559

H	3.486833	-1.890819	0.556230
H	1.652201	0.277153	-2.001291
H	0.357618	-1.537164	-2.420219
H	0.075175	0.908378	2.996554
H	-0.705150	1.592005	1.558059
H	-0.710142	1.197224	-0.543805
C	-0.502890	-2.196935	-0.520987
H	-1.399571	-2.501970	-1.076946
H	-0.295420	-2.982306	0.214535
C	0.303159	-1.615908	2.361947
H	0.067104	-1.373770	3.405829
H	-0.049844	-2.626621	2.142979
H	1.397908	-1.615628	2.272782
C	-2.302523	-0.898559	1.396698
H	-2.588668	-1.892422	1.041963
H	-2.178444	-0.929592	2.483524
C	-3.077930	0.278542	0.863679
H	-4.150803	0.089804	1.033111
H	-2.829468	1.184063	1.435702
C	-1.231419	0.263798	-0.774830
H	-0.986240	0.013736	-1.813198
C	-2.780965	0.474422	-0.643440
C	-3.556559	-0.562027	-1.485777
H	-3.267513	-0.498089	-2.543349
H	-3.390131	-1.596875	-1.157375
H	-4.636101	-0.370502	-1.422552
C	-3.155572	1.892638	-1.107414
H	-2.900909	2.037671	-2.166159
H	-4.234949	2.065644	-0.999571
H	-2.627085	2.658596	-0.522154

**J**	G781.717261		
C	3.012954	2.056534	-0.423251
C	3.438829	0.607940	-0.814927
C	2.269627	-0.199034	-1.310389
C	0.124278	0.837584	1.941218
C	1.454763	1.157051	1.274507
C	1.706864	2.120615	0.355448
H	3.830784	2.535533	0.139465
H	4.201158	0.697716	-1.607508
C	1.925215	-1.469285	-0.996148
C	-0.069297	-0.558207	1.420172
H	2.252773	0.447483	1.494249
C	-0.899986	-0.892914	0.251989
C	0.636100	-2.042482	-1.549906
H	0.813677	-3.051491	-1.956660
C	0.772507	3.242260	-0.008046
H	0.430818	3.149032	-1.051323
H	-0.107558	3.318079	0.641203
H	1.311928	4.200178	0.049872
H	2.889147	2.659699	-1.336294
H	3.941976	0.111169	0.026916
C	2.786227	-2.405097	-0.177515
H	2.184474	-3.109006	0.413256

H	3.407002	-3.021637	-0.846764
H	3.475139	-1.884483	0.500223
H	1.591904	0.344013	-1.979974
H	0.293436	-1.428326	-2.394245
H	0.189047	0.846357	3.038979
H	-0.658535	1.528038	1.616006
H	-0.761766	1.185026	-0.443559
C	-0.523893	-2.185612	-0.524992
H	-1.418745	-2.493887	-1.083595
H	-0.310537	-3.012061	0.166856
C	0.545278	-1.623173	2.271201
H	0.287890	-1.449279	3.325993
H	0.273731	-2.639742	1.984815
H	1.640969	-1.540907	2.214862
C	-2.270648	-1.164756	1.145036
H	-2.686988	-2.089203	0.723966
H	-2.102011	-1.363165	2.212224
C	-3.141216	0.066231	0.901506
H	-4.201861	-0.171202	1.068995
H	-2.880378	0.864353	1.614696
C	-1.316809	0.275084	-0.686946
H	-1.051458	0.034131	-1.722867
C	-2.850707	0.519083	-0.549112
C	-3.633593	-0.354520	-1.554921
H	-3.370358	-0.086833	-2.587501
H	-3.432935	-1.427997	-1.431657
H	-4.715388	-0.206158	-1.434960
C	-3.203963	1.998826	-0.774030
H	-2.930406	2.319742	-1.788947
H	-4.282692	2.167950	-0.652898
H	-2.679425	2.650104	-0.059181

Cartesian coordinates of computed structures (Scheme 2 of main text, Table S16)

Gibbs energies (G"..." in Hartree) and imaginary frequencies of TS (T-"..." in cm^{-1}), mPW1PW91/6-311+G(d,p)//B97D3/6-31G(d,p)-sp-density-fitting, 1 bar, 298.15 K.

$5^{+\bullet}$ G781.088383

C	-3.632277	-1.166789	-0.389691
C	-3.464513	0.051916	-1.409060
C	-2.252289	0.874814	-1.142206
C	0.099506	-1.636613	0.581564
C	-1.392786	-1.338297	0.784695
C	-2.351307	-1.933442	-0.218440
H	-3.954704	-0.744681	0.573471
H	-3.399436	-0.376187	-2.417573
C	-2.123385	1.905954	-0.242009
C	1.067679	-0.637591	1.293530
H	-1.731311	-1.639408	1.792224
C	1.019188	0.739547	0.594939
C	-0.783982	2.565501	-0.069501
H	-0.921470	3.606557	0.251101
C	-2.084444	-3.041746	-0.955552
H	-2.814783	-3.435240	-1.663511
H	-1.151501	-3.594712	-0.853537
H	-4.440632	-1.797363	-0.781506
H	-4.388077	0.643573	-1.345655
C	-3.237698	2.377237	0.649254
H	-3.496805	3.419696	0.403418
H	-4.147742	1.773485	0.578539
H	-2.911844	2.388551	1.701123
H	-1.337395	0.566932	-1.660748
H	-0.225697	2.577929	-1.012207
H	0.334171	-2.654014	0.932583
H	0.307912	-1.622600	-0.497925
C	0.094687	1.849795	1.050157
H	0.725912	2.652079	1.466967
H	-0.559057	1.526398	1.867399
C	0.735629	-0.535379	2.799860
H	-0.293903	-0.214615	2.999215
H	0.864322	-1.520628	3.268494
H	1.411390	0.167194	3.305797
C	2.521796	-1.183008	1.165569
H	3.186689	-0.549655	1.774961
H	2.555687	-2.187628	1.612371
C	3.026685	-1.226697	-0.278214
H	4.041519	-1.647950	-0.312715
H	2.398165	-1.902897	-0.877823
C	1.909989	1.033601	-0.410237
H	1.847479	2.025528	-0.875890
C	3.034915	0.181065	-0.923120
C	4.361043	0.928326	-0.572373
H	4.368741	1.946938	-0.983332
H	4.507811	0.995422	0.513022
H	5.207890	0.379301	-1.005834
C	2.925985	0.079003	-2.468366

H	2.971968	1.069658	-2.941647
H	3.760667	-0.520536	-2.856814
H	1.986634	-0.406143	-2.768445
H	-1.549131	-0.248179	0.769688

$5^{+\bullet}$-$L^{+\bullet}$-TS G781.057613, T1204

C	2.827502	1.623432	0.785918
C	3.104265	1.289919	-0.724231
C	2.299177	0.153950	-1.316468
C	-0.524932	-0.049757	2.009651
C	0.705802	0.389624	1.271835
C	1.370714	1.661382	1.232910
H	3.338591	0.873586	1.407790
H	2.905372	2.193034	-1.317326
C	2.315826	-1.157412	-0.969156
C	-1.502746	-1.040188	1.161402
H	1.389113	-0.442065	1.102729
C	-0.864062	-1.234227	-0.198573
C	1.259561	-2.116516	-1.498330
H	1.709241	-3.103380	-1.682473
C	0.756464	2.846968	1.581469
H	1.293046	3.793489	1.528852
H	-0.270827	2.881735	1.942211
H	3.302924	2.589303	1.002704
H	4.185172	1.097794	-0.808335
C	3.308882	-1.745556	0.007219
H	3.805293	-2.616931	-0.446320
H	4.088180	-1.035482	0.303373
H	2.828102	-2.119010	0.927252
H	1.587715	0.454633	-2.089005
H	0.860211	-1.774156	-2.462529
H	-0.181819	-0.631947	2.878386
H	-1.116626	0.786920	2.399152
C	0.081772	-2.370052	-0.490365
H	-0.539829	-3.185274	-0.907496
H	0.472824	-2.777239	0.452564
C	-1.710909	-2.349166	1.946529
H	-0.766833	-2.859109	2.177601
H	-2.204936	-2.128409	2.901543
H	-2.352630	-3.046974	1.392193
C	-2.870786	-0.315700	0.975888
H	-3.552435	-0.989333	0.433491
H	-3.314907	-0.163978	1.970510
C	-2.746242	1.026088	0.246552
H	-3.742609	1.464942	0.094026
H	-2.197721	1.742174	0.876927
C	-0.896500	-0.124188	-1.056287
H	-0.361133	-0.218216	-2.002692
C	-2.029160	0.903205	-1.116418
C	-3.009088	0.380670	-2.202210
H	-2.504710	0.251035	-3.169824
H	-3.451741	-0.583721	-1.919413
H	-3.821781	1.105911	-2.342583
C	-1.460682	2.269394	-1.555853

H	-0.977529	2.201552	-2.541167
H	-2.268847	3.008883	-1.631173
H	-0.720430	2.646123	-0.836082
H	0.092406	0.332903	-0.183783

**L**$^{+\bullet}$	G781.077208		
C	-3.584642	-1.299476	-0.040425
C	-3.630019	-0.316208	-1.250984
C	-2.410012	0.571884	-1.380051
C	0.065007	-1.431515	1.349336
C	-1.384562	-1.288091	1.168025
C	-2.182895	-1.860780	0.149501
H	-3.889364	-0.771276	0.873574
H	-3.725863	-0.902586	-2.175628
C	-2.071343	1.620946	-0.600402
C	0.937022	-0.044586	1.443897
H	-1.902492	-0.638869	1.878749
C	0.857476	0.752205	0.197382
C	-0.794819	2.390494	-0.899803
H	-0.993499	3.473912	-0.892768
C	-1.728572	-2.866236	-0.684815
H	-2.379346	-3.290510	-1.448912
H	-0.733792	-3.301380	-0.596578
H	-4.311546	-2.106909	-0.204211
H	-4.547970	0.285574	-1.170710
C	-2.908796	2.129805	0.549330
H	-3.128899	3.200785	0.420058
H	-3.863588	1.602115	0.647037
H	-2.381731	2.041791	1.513374
H	-1.724364	0.322414	-2.196339
H	-0.430391	2.142703	-1.905507
H	0.266892	-1.857492	2.346491
H	0.521445	-2.088512	0.603091
C	0.340013	2.149121	0.146703
H	1.199121	2.776528	-0.165702
H	0.024739	2.511724	1.129620
C	0.525454	0.701736	2.728075
H	-0.524719	1.016869	2.720836
H	0.669162	0.044624	3.595297
H	1.146860	1.592264	2.886249
C	2.437135	-0.528482	1.603873
H	3.018855	0.341203	1.941445
H	2.450942	-1.254177	2.431264
C	3.065574	-1.128801	0.345990
H	4.132565	-1.318360	0.540594
H	2.620736	-2.111556	0.122488
C	1.399614	0.148243	-1.046257
H	1.268154	0.813973	-1.908111
C	2.931145	-0.219786	-0.895382
C	3.754369	1.076164	-0.742826
H	3.613360	1.733521	-1.612185
H	3.482039	1.649047	0.154661
H	4.824635	0.840646	-0.673936
C	3.375070	-0.970107	-2.163084

H	3.250595	-0.346747	-3.059218
H	4.437753	-1.240181	-2.090873
H	2.798455	-1.895139	-2.304810
H	0.857937	-0.785175	-1.267849

L$^{+\bullet}$ G781.077207

C	-3.584473	-1.299305	-0.040608
C	-3.629614	-0.316208	-1.251270
C	-2.409670	0.572020	-1.380092
C	0.065064	-1.431806	1.349334
C	-1.384510	-1.288470	1.168112
C	-2.182814	-1.860839	0.149350
H	-3.889060	-0.770931	0.873330
H	-3.725136	-0.902707	-2.175874
C	-2.071262	1.621067	-0.600314
C	0.936942	-0.044770	1.443944
H	-1.902502	-0.639548	1.879067
C	0.857353	0.752215	0.197579
C	-0.794767	2.390792	-0.899407
H	-0.993518	3.474191	-0.892100
C	-1.728529	-2.866223	-0.685045
H	-2.379266	-3.290316	-1.449275
H	-0.733824	-3.301516	-0.596720
H	-4.311543	-2.106615	-0.204282
H	-4.547658	0.285489	-1.171354
C	-2.908946	2.129720	0.549348
H	-3.128943	3.200743	0.420248
H	-3.863804	1.602089	0.646720
H	-2.382106	2.041462	1.513493
H	-1.723900	0.322732	-2.196331
H	-0.430223	2.143302	-1.905143
H	0.267068	-1.857811	2.346439
H	0.521514	-2.088659	0.602975
C	0.339906	2.149161	0.147194
H	1.199070	2.776677	-0.164830
H	0.024416	2.511464	1.130151
C	0.525437	0.701317	2.728280
H	-0.524771	1.016336	2.721210
H	0.669319	0.044084	3.595384
H	1.146763	1.591883	2.886518
C	2.437157	-0.528598	1.603751
H	3.018823	0.341118	1.941332
H	2.451041	-1.254334	2.431105
C	3.065488	-1.128791	0.345777
H	4.132493	-1.318379	0.540272
H	2.620629	-2.111526	0.122217
C	1.399523	0.148463	-1.046197
H	1.268010	0.814378	-1.907905
C	2.930949	-0.219648	-0.895499
C	3.754259	1.076253	-0.742861
H	3.613231	1.733703	-1.612146
H	3.482004	1.649066	0.154696
H	4.824516	0.840675	-0.674049
C	3.374802	-0.969855	-2.163302

H	3.250300	-0.346391	-3.059360
H	4.437475	-1.239969	-2.091161
H	2.798143	-1.894848	-2.305105
H	0.857683	-0.784821	-1.267963

L$^{+\bullet}$-M$^{+\bullet}$-TS G781.072939, T-421

C	3.451826	1.402212	0.185033
C	3.515833	0.717181	-1.213602
C	2.387984	-0.259684	-1.461891
C	-0.062670	1.071107	1.816727
C	1.324154	0.988278	1.482484
C	2.030094	1.807167	0.546517
H	3.835582	0.713336	0.949546
H	3.483324	1.494485	-1.989870
C	2.188226	-1.443609	-0.845491
C	-1.230774	-0.410738	1.453790
H	1.915278	0.228959	2.001660
C	-0.857212	-0.876576	0.143794
C	0.945067	-2.255632	-1.163859
H	1.204801	-3.320659	-1.266444
C	1.482154	2.939506	-0.017288
H	2.059969	3.547317	-0.713021
H	0.479989	3.293585	0.220812
H	4.110916	2.281527	0.184452
H	4.496775	0.226618	-1.309105
C	3.135522	-2.045638	0.166904
H	3.396256	-3.075771	-0.120070
H	4.069486	-1.482366	0.266484
H	2.679721	-2.113809	1.168595
H	1.641728	0.047857	-2.200987
H	0.520019	-1.944341	-2.127135
H	-0.258685	0.973570	2.888531
H	-0.617027	1.891779	1.360443
C	-0.122043	-2.166880	-0.042182
H	-0.933958	-2.888935	-0.281506
H	0.300764	-2.525799	0.903601
C	-1.014158	-1.353583	2.640501
H	0.039253	-1.618831	2.790883
H	-1.374815	-0.880260	3.562385
H	-1.584051	-2.283828	2.506439
C	-2.605819	0.298408	1.533950
H	-3.345612	-0.503994	1.689658
H	-2.637193	0.911686	2.446389
C	-2.991433	1.130409	0.307218
H	-4.052087	1.412426	0.385307
H	-2.427667	2.077773	0.293733
C	-1.256623	-0.075133	-1.051394
H	-1.051072	-0.625997	-1.977916
C	-2.747188	0.390124	-1.024651
C	-3.659860	-0.849133	-1.153284
H	-3.479015	-1.368403	-2.104704
H	-3.495809	-1.576238	-0.344376
H	-4.717471	-0.554558	-1.128315
C	-2.992461	1.341270	-2.209588

H	-2.793269	0.842103	-3.168184
H	-4.037872	1.678908	-2.221043
H	-2.349799	2.231527	-2.148474
H	-0.602170	0.815999	-1.079895

**$M^{+\bullet}$**	G781.079026		
C	-3.383912	-1.407918	0.639365
C	-3.412570	-1.088383	-0.897746
C	-2.327224	-0.160959	-1.381916
C	-0.229098	-0.005087	2.318932
C	-1.457921	-0.218450	1.761499
C	-1.985099	-1.469402	1.220779
H	-3.954937	-0.639883	1.179383
H	-3.320648	-2.035257	-1.446315
C	-2.178599	1.163168	-1.124469
C	1.864882	1.207827	0.907302
H	-2.157745	0.618875	1.757744
C	1.012809	1.028141	-0.185375
C	-0.983437	1.909134	-1.686511
H	-1.311659	2.896931	-2.045177
C	-1.283992	-2.641186	1.233050
H	-1.713652	-3.553702	0.820232
H	-0.290671	-2.726694	1.672377
H	-3.902797	-2.362253	0.798583
H	-4.410818	-0.687186	-1.131335
C	-3.175928	1.990408	-0.349996
H	-3.632233	2.738170	-1.017891
H	-3.987050	1.398456	0.086882
H	-2.695291	2.562467	0.458763
H	-1.566229	-0.623745	-2.014715
H	-0.580417	1.382881	-2.561799
H	-0.015775	0.936727	2.817922
H	0.509250	-0.796128	2.426306
C	0.149586	2.178508	-0.657973
H	0.844208	2.901363	-1.128353
H	-0.260655	2.716183	0.209719
C	1.957895	2.516531	1.647712
H	0.984234	2.993892	1.813350
H	2.456053	2.389004	2.616206
H	2.561100	3.233560	1.065357
C	2.898415	0.177333	1.280963
H	3.882658	0.625135	1.044175
H	2.913617	0.069449	2.378924
C	2.756332	-1.186878	0.597038
H	3.696852	-1.747623	0.696207
H	1.986739	-1.784592	1.113131
C	1.052891	-0.245597	-0.975949
H	0.815858	-0.039165	-2.029828
C	2.367441	-1.059898	-0.889648
C	3.473510	-0.339093	-1.692242
H	3.209152	-0.291132	-2.757752
H	3.636968	0.692135	-1.348312
H	4.427125	-0.877991	-1.608574
C	2.127876	-2.459429	-1.486362

H	1.811773	-2.393286	-2.537173
H	3.047298	-3.059634	-1.451937
H	1.348407	-3.001663	-0.930582
H	0.206142	-0.867906	-0.616237

M$^{+\bullet}$ G781.071219

C	3.282549	0.933296	0.487403
C	3.721720	-0.043049	-0.625871
C	2.564014	-0.819721	-1.212083
C	0.264799	3.165980	1.157108
C	1.447332	2.501499	1.222055
C	2.183065	1.910484	0.096805
H	2.935303	0.355724	1.357527
H	4.235516	0.505731	-1.427158
C	1.866676	-1.809063	-0.580228
C	-1.376546	-0.937241	1.363749
H	1.898687	2.354380	2.208698
C	-1.069123	-1.223358	0.036383
C	0.734617	-2.502833	-1.283838
H	1.000908	-3.560606	-1.450058
C	1.930719	2.253085	-1.195866
H	2.505990	1.847791	-2.024792
H	1.172001	2.991552	-1.453171
H	4.153971	1.508630	0.846197
H	4.469685	-0.729238	-0.199024
C	2.143861	-2.242914	0.833026
H	1.976964	-3.321722	0.961354
H	3.160900	-2.008795	1.166341
H	1.443433	-1.734997	1.520423
H	2.269550	-0.587649	-2.238776
H	0.563341	-2.056858	-2.272481
H	-0.190955	3.579734	2.055719
H	-0.261810	3.336625	0.218310
C	-0.617411	-2.552311	-0.468034
H	-1.371070	-2.928026	-1.182048
H	-0.541665	-3.297379	0.331999
C	-1.295928	-1.903305	2.507048
H	-0.795436	-2.846537	2.270162
H	-0.783487	-1.440176	3.364321
H	-2.314765	-2.135842	2.860652
C	-1.811676	0.462226	1.673049
H	-2.195942	0.540589	2.699211
H	-0.876931	1.075179	1.656890
C	-2.799109	1.095646	0.659330
H	-3.826514	0.883204	0.988296
H	-2.681281	2.187334	0.712112
C	-1.225007	-0.098962	-0.949778
H	-1.093149	-0.457689	-1.979858
C	-2.612369	0.602671	-0.810563
C	-3.720073	-0.412938	-1.170637
H	-3.745389	-1.254889	-0.463525
H	-4.706196	0.070224	-1.149201
H	-3.569519	-0.821678	-2.180662
C	-2.669557	1.790343	-1.789557

H	-2.573414	1.452925	-2.831294
H	-3.626867	2.321933	-1.697702
H	-1.863458	2.512315	-1.591353
H	-0.423714	0.641007	-0.774121

M$^{+\bullet}$-O$^{+\bullet}$-TS G781.009982, T-316

C	2.785815	1.356352	0.893139
C	3.733104	0.468245	0.054499
C	2.986529	-0.518574	-0.809421
C	-0.341503	3.447024	0.489674
C	0.818245	2.932209	0.946555
C	1.891616	2.322035	0.127566
H	2.148027	0.704128	1.513642
H	4.381023	1.099905	-0.569866
C	2.428239	-1.680965	-0.385804
C	-1.233505	-1.262940	0.918860
H	0.998221	2.939374	2.027745
C	-0.438326	-1.302394	-0.283788
C	1.574123	-2.512081	-1.319958
H	1.976731	-3.533901	-1.410673
C	2.079584	2.652336	-1.168387
H	2.865529	2.206110	-1.774396
H	1.473032	3.419412	-1.649536
H	3.385945	1.948215	1.607425
H	4.399044	-0.059826	0.752955
C	2.514989	-2.173563	1.037939
H	2.622666	-3.267604	1.070958
H	3.348976	-1.730828	1.593554
H	1.592332	-1.929011	1.593653
H	2.859150	-0.260316	-1.864439
H	1.583772	-2.079247	-2.330404
H	-1.054129	3.917482	1.167363
H	-0.586939	3.460499	-0.573707
C	0.115366	-2.631528	-0.789367
H	-0.530172	-3.032858	-1.587369
H	0.093902	-3.376638	0.014881
C	-1.681146	-2.546125	1.578702
H	-0.829242	-3.083664	2.023751
H	-2.399739	-2.350839	2.382924
H	-2.144216	-3.237280	0.860301
C	-1.674224	-0.033253	1.491648
H	-1.944688	-0.079402	2.548453
H	-1.194180	0.902552	1.204698
C	-3.586705	0.385259	0.954090
H	-4.056229	-0.498960	1.389246
H	-3.666067	1.292773	1.555436
C	-0.136650	-0.147049	-0.977093
H	0.389934	-0.188526	-1.925661
C	-3.613596	0.519635	-0.445513
C	-3.802034	-0.694210	-1.296649
H	-2.872052	-1.292217	-1.256416
H	-4.602534	-1.342203	-0.913823
H	-3.996146	-0.449647	-2.346978
C	-3.382326	1.839514	-1.108005

H	-2.647122	1.754236	-1.922133
H	-4.320557	2.169583	-1.587890
H	-3.065367	2.618202	-0.406062
H	-0.434475	0.840942	-0.639751

**O+•**	G781.006668		
C	2.733652	1.421364	0.899454
C	3.721032	0.553881	0.081414
C	3.022425	-0.463146	-0.781613
C	-0.418639	3.454980	0.450781
C	0.750328	2.975074	0.922370
C	1.833710	2.366670	0.117036
H	2.098890	0.758009	1.511279
H	4.356401	1.201031	-0.539621
C	2.505934	-1.651188	-0.357230
C	-1.154374	-1.268944	0.923316
H	0.928339	3.009815	2.003105
C	-0.343179	-1.307792	-0.292408
C	1.682109	-2.508719	-1.295643
H	2.105900	-3.522836	-1.369699
C	2.023019	2.680573	-1.184442
H	2.814628	2.233739	-1.782699
H	1.410328	3.434704	-1.677626
H	3.310775	2.026065	1.621329
H	4.390100	0.053133	0.796598
C	2.612121	-2.139879	1.064747
H	2.758253	-3.229169	1.095479
H	3.428959	-1.666626	1.620226
H	1.681068	-1.930368	1.620190
H	2.893213	-0.216473	-1.838868
H	1.694690	-2.085127	-2.309673
H	-1.145457	3.920204	1.116394
H	-0.666197	3.432043	-0.611713
C	0.224493	-2.639513	-0.770646
H	-0.418050	-3.061762	-1.560461
H	0.208769	-3.369689	0.047230
C	-1.673440	-2.562967	1.510125
H	-0.861166	-3.160787	1.950939
H	-2.405819	-2.370475	2.301954
H	-2.148403	-3.190896	0.743826
C	-1.470926	-0.076443	1.548419
H	-1.970943	-0.096018	2.515297
H	-1.091515	0.888089	1.217807
C	-3.963154	0.553615	0.925804
H	-4.318073	-0.301829	1.499983
H	-3.905475	1.512875	1.440505
C	-0.060750	-0.163363	-1.002512
H	0.467333	-0.210787	-1.950324
C	-3.747964	0.455720	-0.418892
C	-3.856633	-0.858404	-1.135046
H	-2.859505	-1.158900	-1.502201
H	-4.247428	-1.657382	-0.494856
H	-4.496195	-0.770340	-2.025967
C	-3.377086	1.652244	-1.250602

H	-2.487665	1.443547	-1.865293
H	-4.188136	1.877104	-1.961873
H	-3.193250	2.546086	-0.644344
H	-0.400435	0.820435	-0.693516

References

[1] J. S. Dickschat, K. A. K. Pahirulzaman, P. Rabe, T. A. Klapschinski, *ChemBioChem* **2014**, *15*, 810-814.

[2] R. D. Gietz, R. H. Schiestl, *Nat. Protoc.* **2007**, *2*, 31-34.

[3] M. M. Bradford, *Anal. Biochem.* **1976**, *72*, 248-254.

[4] G. R. Fulmer, A. J. M. Miller, N. H. Sherden, H. E. Gottlieb, A. Nudelman, B. M. Stoltz, J. E. Bercaw, K. I. Goldberg, *Organometallics* **2010**, *29*, 2176-2179.

[5] R. P. Adams, *Identification of Essential Oil Components by Gas Chromatography/ Mass Spectrometry*, 4th Edition, Allured Business Media, Carol Stream, Illinois, USA, **2009**.

[6] J. Rinkel, J. S. Dickschat, *Org. Lett.* **2019**, *21*, 2426-2429.

[7] F. M. Hahn, A. P. Hurlburt, C. D. Poulter, *J. Bacteriol.* **1999**, *181*, 4499-4504.

[8] P. Rabe, J. Rinkel, E. Dolja, T. Schmitz, B. Nubbemeyer, T. H. Luu, J. S. Dickschat, *Angew. Chem. Int. Ed.* **2017**, *56*, 2776-2779; *Angew. Chem.* **2017**, *129*, 2820-2823.

[9] V. M. Dixit, F. M. Laskovics, W. I. Noall, C. D. Poulter, *J. Org. Chem.* **1981**, *46*, 1967-1969.

[10] L. Lauterbach, J. Rinkel, J. S. Dickschat, *Angew. Chem. Int. Ed.* **2018**, *57*, 8280-8283; *Angew. Chem.* **2018**, *130*, 8412-8415.

[11] J. Rinkel, L. Lauterbach, J. S. Dickschat, *Angew. Chem. Int. Ed.* **2019**, *58*, 452-455; *Angew. Chem.* **2019**, *131*, 461-465.

[12] P. Rabe, L. Barra, J. Rinkel, R. Riclea, C. A. Citron, T. A. Klapschinski, A. Janusko, J. S. Dickschat, *Angew. Chem. Int. Ed.* **2015**, *54*, 13448-13451; *Angew. Chem.* **2015**, *127*, 13649-13653.

[13] T. Lou, A. Li, H. Xu, J. Pan, B. Xing, R. Wu, J. S. Dickschat, D. Yang, M. Ma, *J. Am. Chem. Soc.* **2023**, *145*, 8474-8485.

[14] G. Bian, J. Rinkel, Z. Wang, L. Lauterbach, A. Hou, Y. Yuan, Z. Deng, T. Liu, J. S. Dickschat, *Angew. Chem. Int. Ed.* **2018**, *57*, 15887-15890; *Angew. Chem.* **2018**, *130*, 16113-16117.

[15] A. Hou, J. S. Dickschat, *Angew. Chem. Int. Ed.* **2020**, *59*, 19961-19965; *Angew. Chem.* **2020**, *132*, 20135-20140.

[16] H. Li, J. S. Dickschat, *Org. Chem. Front.* **2022**, *9*, 795-801.

[17] S. Grimme, S. Ehrlich, L. Goerigk, *J. Comp. Chem.* **2011**, *32*, 1456-1465.

[18] Gaussian 16, Revision C.01, M. J. Frisch, G. W. Trucks, H. B. Schlegel, G. E. Scuseria, M. A. Robb, J. R. Cheeseman, G. Scalmani, V. Barone, G. A. Petersson, H. Nakatsuji, X. Li, M. Caricato, A. V. Marenich, J. Bloino, B. G. Janesko, R. Gomperts, B. Mennucci, H. P. Hratchian, J. V. Ortiz, A. F. Izmaylov, J. L. Sonnenberg, D. Williams-Young, F. Ding, F. Lipparini, F. Egidi, J. Goings, B. Peng, A. Petrone, T. Henderson, D. Ranasinghe, V. G. Zakrzewski, J. Gao, N. Rega, G. Zheng, W. Liang, M. Hada, M. Ehara, K. Toyota, R. Fukuda, J. Hasegawa, M. Ishida, T. Nakajima, Y. Honda, O. Kitao, H. Nakai, T. Vreven, K. Throssell, J. A. Montgomery, Jr., J. E. Peralta, F. Ogliaro, M. J. Bearpark, J. J. Heyd, E. N. Brothers, K. N. Kudin, V. N. Staroverov, T. A. Keith, R. Kobayashi, J. Normand, K. Raghavachari, A. P. Rendell, J. C. Burant, S. S. Iyengar, J. Tomasi, M. Cossi, J. M. Millam, M. Klene, C. Adamo, R. Cammi, J. W. Ochterski, R. L. Martin, K. Morokuma, O. Farkas, J. B. Foresman, and D. J. Fox, Gaussian, Inc., Wallingford CT, 2019.

[19] S. Grimme, *Chem. Eur. J.* **2012**, *18*, 9955-9964.

[20] GoodVibes v3.0.1, G. Luchini, J. V. Alegre-Requena, Y. Guan, I. Funes-Ardoiz, R. S. Paton, 2019.

[21] C. Adamo, V. Barone, *J. Chem. Phys.* **1998**, *108*, 664.

[22] S. P. T. Matsuda, W. K. Wilson, Q. Xiong, *Org. Biomol. Chem.* **2006**, *4*, 530-543.

[23] Y. J. Hong, D. J. Tantillo, *J. Org. Chem.* **2018**, *83*, 3780-3793.

[24] L. Lauterbach, B. Goldfuss, J. S. Dickschat, *Angew. Chem. Int. Ed.* **2020**, *59*, 11943-11947; *Angew. Chem.* **2020**, *132*, 12041-12045.

[25] H. Xu, B. Goldfuss, J. S. Dickschat, *Chem. Eur. J.* **2021**, *27*, 9758-9762.

[26] P. Pracht, F. Bohle, S. Grimme, *Phys. Chem. Chem. Phys.* **2020**, *22*, 7169-7192.
[27] S. Grimme, *J. Chem. Theory Comput.* **2019**, *155*, 2847-2862.
[28] P. Pracht, S. Grimme, *Chem. Sci.* **2021**, *12*, 6551-6568.
[29] P. Pracht, C.A. Bauer, S. Grimme, *J. Comput. Chem.* **2017**, *38*, 2618-2631.
[30] S. Spicher, C. Plett, P. Pracht, A. Hansen, S. Grimme, *J. Chem. Theory Comput.* **2022**, *18*, 3174-3189.
[31] R. Higuchi, B. Krummel, R. Saiki, *Nucleic Acids Res.* **1988**, *16*, 7351.
[32] J. Rinkel, L. Lauterbach, P. Rabe, J. S. Dickschat, *Angew*. *Chem*. *Int*. *Ed*. **2018**, *57*, 3238-3241; *Angew. Chem.* **2018**, *130*, 3292-3296.
